# Global burden of ischemic stroke in adults aged 60 years and older from 1990 to 2021: Population-based study

**DOI:** 10.1371/journal.pone.0322606

**Published:** 2025-05-05

**Authors:** Xiuen Chen, Lizhi Lu, Chao Xiao, Yining Lan, Songxin Zhong, Chao Qin, Yanyan Tang

**Affiliations:** 1 Department of Neurology, Liuzhou People’s Hospital Affiliated to Guangxi Medical University, Liuzhou, Guangxi Province, China; 2 Department of Neurology, The First Affiliated Hospital of Guangxi Medical University, Nanning, Guangxi Province, China; 3 The Reproduction Hospital of Guangxi Zhuang Autonomous Region & The Reproductive Health Research Center of Guangxi Zhuang Autonomous Region, Nanning, Guangxi Province, China; 4 Department of Neurology, The First People’s Hospital of Yulin Affiliated to Guangxi Medical University, Yulin, Guangxi Province, China; Cedars-Sinai Medical Center, UNITED STATES OF AMERICA

## Abstract

**Background:**

Ischemic stroke is an important public health problem. However, comprehensive data on its burden in aging populations is limited. The aim of this study is to provide an up-to-date assessment of the prevalence, incidence, mortality, disability-adjusted life years, and risk factors for ischemic stroke globally in adults aged 60 years and older from 1990 to 2021 based on population changes.

**Methods:**

The Global Burden of Diseases, Injuries, and Risk Factors Study 2021 served as the data source for this study. Average annual percentage changes were estimated over the study period to quantify temporal patterns and assess trends in age-standardized rates of the prevalence, incidence, mortality, and disability-adjusted life-years of ischemic stroke.

**Results:**

The significant increase in the prevalence and incidence of ischemic stroke is mainly related to population ageing and the significant increase in the number of people over 60 years of age, with the significant increase in the population over 60 years of age being the main driving force, while epidemiological changes have had the opposite effect. Critically, using the entire age population for calculations will prompt us to underestimate the burden of ischemic stroke. The burden of ischemic stroke disease is highest in older men than in older women, and the age-standardized prevalence rates, incidence rates, mortality rates, and disability-adjusted life-years rates are 26–35% higher in men than in women. High-middle sociodemographic index and Sub-Saharan Africa regions suffer the heaviest burden. Ischemic stroke health inequities widen, with less developed regions bearing a heavier ischemic stroke burden and the disparity in that burden becoming more pronounced over time.

**Conclusion:**

Population aging is the primary driver of the growing burden of ischemic stroke. Our findings indicate that prevention and control of this disease remain critical public health challenges. Targeted interventions addressing modifiable risk factors could significantly reduce the global burden of ischemic stroke.

## Introduction

Stroke is recognized as a globally significant public health problem, a fatal and disabling vascular condition with profound impact on quality of life, healthcare systems, and economic stability. According to the 2019 Global Burden of Disease(GBD) study, stroke ranks as the second leading cause of death globally (11.6% [95% UI 10.8–12.2]))and the third leading cause of disability globally (5.7% of total all-cause disability-adjusted life-years(DALYs) [95% UI 5.1–6.2] [1]. The resulting disease burden of up to 123 million DALYs makes stroke a major cause of increased human suffering. Of these, ischemic stroke(ischemic stroke) is the most prevalent type of stroke, making up 62.4% of all stroke cases[1]. Acute ischemic stroke is the most common type of stroke, and its clinical management centers on three pillars: early diagnosis and treatment(intravenous thrombolysis and mechanical thrombectomy), early secondary prevention(targeting modifiable risk factors) and early rehabilitation. In a recent update, the lancet published a Seminar Series on acute ischemic stroke,reviewing recent advances in acute interventions, secondary prevention strategies, and emerging therapeutic frontiers[2]. Ischemic stroke causes neurological damage leading to disability and even death, imposing a severe health and economic burden on individuals and society. Consequently, developing targeted prevention strategies is essential to mitigate this burden.

Notably, ischemic stroke is generally considered to be a disease highly associated with aging and is often regarded as a disease of the elderly[3]. However, existing burden estimates often rely on analyses of the entire age range population [4–8], it may obscure the disproportionate impact on older adults. Critically, we posit that such broad age group analyses systematically underestimate ischemic stroke’s true burden in aging population. To address this gap, our study focus specifically on adults age-range in ≥ 60 years.

To this end, we comprehensively analyzed the burden of disease for ischemic stroke, including incidence, prevalence, mortality, and DALYs, among adults ≥ 60 years, globally, in 21 regions, and at the country level in 204 countries from 1990 to 2021 based on population changes, using the most recent GBD data available for the year 2021. At the same time, we used the population attributable fraction (PAF) to quantify the effect of specific risk factors on ischemic stroke-related DALYs.

## Materials and methods

### Data sources and study population

The ischemic stroke data analyzed in this study were derived from GBD2021, which provides the latest estimates of epidemiological data on the burden of 371 diseases and injuries across 21 GBD regions and 204 countries and territories from 1990 to 2021. All this data is available for free access through the Global Health Data Exchange (https://ghdx.healthdata.org/gbd-2021/sources).

The study population included patients with ischemic stroke aged 60 years and older, including patients diagnosed with ischemic stroke before age of 60 years. We extracted information on ischemic stroke in people aged 60 years and older from the Global Burden of Disease Study 2021: location-, age-, and sex-specific prevalence, incidence, mortality, numbers and rates for DALYs; and DALYs attributable to each risk factor (with corresponding 95% uncertainty intervals (UIs)).

To analyze the attributable burden of ischemic stroke to 23 risk factors currently available for such analysis in GBD 2021, we calculated population attributable fractions (PAFs) of DALYs, using the exposure level for each risk factor and theoretical minimum risk exposure level (TMREL) that minimizes risk for each individual in the population as the reference variable. Adjustments for mediation were applied to account for relationships involving risk factors that act indirectly on outcomes via intermediate risks, as described elsewhere[9]. Relative risk data were pooled using meta-regression of cohort, case–control, or intervention studies. From the prevalence and relative risk results, PAFs were estimated relative to the TMREL. The PAF represents a proportion of the stroke DALYs that would be decreased if the exposure to the risk factor in the past had been at the counterfactual level of the TMREL.

The 23 risks included in the analysis were Ambient particulate matter pollution; Kidney dysfunction; Household air pollution from solid fuels; Lead exposure; Secondhand smoke; Smoking; Alcohol use; High systolic blood pressure; High body mass index; Diet low in whole grains; Diet high in red meat; Diet low in fiber; Diet low in vegetables; Diet low in polyunsaturated fatty acids; Diet high in sugar-sweetened beverages; High fasting plasma glucose; Low temperature; Low physical activity; Diet low in fruits; High temperature; High LDL cholesterol; Diet high in processed meat; Diet high in sodium. The calculation formula of PAF is as follows:


$$PAF_{joasgt}=\;\frac{\sum_{x=1}^{u}RR_{joast}(x)P_{jasgt}(x)-RR_{joasg}(TMRE_{jas})}{\sum_{x=1}^{u}RR_{joas}(x)P_{jasgt}(x)}$$

### Case definition

In GBD2021, ischemic stroke are characterized by occlusion of blood flow to part of the brain due to hypoperfusion, most commonly due to a thrombus or embolism. It is described as an episode of neurological dysfunction caused by focal cerebral, spinal, or retinal infarction. Cases of transient ischaemic attack (TIA) were not listed. In GBD 2021, We did not differentiate between acute and chronic ischemic stroke because most epidemiological studies only report overall ischemic stroke only. According to the International Classification of Diseases(ICD), 9th and 10th editions, ischemic stroke is represented by codes 433–435.9, 437.0–437.1, 437.5–437.8 and I63-I63.9, I65-I66.9, I67.2-I67.3, I67.5-I67.6, I69.3, respectively[10].

### Burden evaluation indicators

We evaluated the burden of disease in ischemic stroke using four indicators: incidence, prevalence, mortality, and disability-adjusted life-years (DALYs). We also used age-standardized rates to eradicate the effects of differences in age distribution across countries, and calculated weighted averages by using the composition of the “standard population” to obtain a single, generalized indicator for comparison. DALYs is a standard comprehensive metric for quantifying the burden of disease, which is the sum of years lived with disability (YLDs) and years of life lost (YLLs). YLLs is a metric calculated by multiplying the estimated number of deaths by the prevailing life expectancy of the age of death, emphasizing premature deaths caused by certain factors. YLDs are equal to the number of years of health loss due to illness or injury. The ASR was computed per 100,000 individuals utilizing the subsequent formula:


$$ASR\;=\;\frac{\sum_{i=1}^{A}a_{i}w_{i}}{\sum_{i=1}^{A}w_{i}}\;\times 100,000$$

Where a_i_ and w_i_ denote age-specific rates and the number of persons (or weight) in the same age subgroup of the chosen reference standard population (where i denotes the age class), respectively.

### Age group and sociodemographic index

In this study, we analyzed ischemic stroke data from 21 GBD regions from geographically adjacent countries with similar epidemiological characteristics, including men and women in eight age groups (60–64 years, 65–69 years, 70–74 years, 75–79 years, 80–84 years, 85–89 years, 90–94 years, and ≥ 95 years). And, we age-standardized this population using the corresponding population numbers. We also calculated the sociodemographic index(SDI) for each country to estimate the relationship between ischemic stroke burden and socioeconomic development. The SDI is a composite indicator of social and economic conditions that affect health outcomes in each region. The SDI ranges from 0 to 1. In this study, 1 stands for highest education level, the highest per capita income, and the lowest fertility rate. The sociodemographic index is subdivided into quintiles: low, low-middle, middle, high-middle, and high SDI.

### BAPC forecasting model

The Bayesian age-period-cohort (BAPC) is a method for analyzing and predicting trends in disease burden by applying Bayesian formulas to calculate hypothetical probability distributions based on 3 factors: age, period, and cohort and combining a priori and sample information to derive posterior information. Compared with methods that estimate the overall parameters from sample statistics only, BAPC is more flexible in the choice of parameters and prior probability distributions, and the predictions are more robust and reliable. We used the absolute percentage deviation (APD) to evaluate the performance of the BAPC model[11,12]. The calculation formula of BAPC is as follows:


$$\log\left(u_{a,p,c}\right)=a+\theta_{a}+\gamma_{p}+\delta_{c}+\in_{a,p,c}$$

a, p and c represent age (Age), period (Period) and queue (Cohort) respectively. $\theta_{a}$ represents age fixed effect, capturing systematic age-specific impacts on utility. $\gamma_{p}$ represents individual random effect, capturing unobserved heterogeneity across individuals. $\delta_{c}$ represents context fixed effect, capturing impacts of environmental factors (e.g., region, time, policy) on utility. $\in_{a,p,c}$ represents Random error term and unexplained stochastic disturbances.

### Statistical analysis

Descriptive analyses were performed to characterize the burden of ischemic stroke in adults ages ≥60 years on a global scale. We compared age-standardized prevalence rates(ASPR, per 100 000 population), age-standardized mortality rates(ASMR, per 100 000 population), age-standardized DALYs rates(ASDR, per 100 000 population) and age-standardized incidence rates(ASIR, per 100 000 population) for ischemic stroke across age groups, sexes, regions, and countries. And are used to represent the average increase or rate of change of a specific variable over a specified period. In this study, it is the annual change percentage transformed from the weighted average of the slope coefficients of the underlying join point regression model from 1990 to 2021. The AAPC value denotes the percentage annual change (increase, decrease, or no change). If yearly percentage change estimates and 95% CIs were both >0 (or both <0), we considered the corresponding rate to be in an upward (or downward) trend. Based on the data on ischemic stroke and its associated risk factors obtained from the Global Burden of Disease Study, we further calculated age-standardized prevalence rates and corresponding 95% confidence intervals (CIs) for the world’s standardized population in 2021 reported by the Global Burden of Disease Study for comparisons across regions, and further estimated the AAPC by joint-point regression to measure the time trend. The calculation formula of AAPC is as follows:


$$AAPC\;=\;\left(e^{\left(\frac{\sum_{i-1}^{k}b_{i}w_{i}}{\sum_{i-1}^{k}w_{i}}\right)}-1\right)\times 100$$

b_i_ is the slope coefficient of the i^-th^ segment (i.e., the Annual Percentage Change, APC). w_i_ is the length of time for each segment (usually measured in years).“k” is the total number of segments.” ∑ represents the summation.

We performed a decomposition analysis of the burden of ischemic stroke from 1990 to 2021, decomposing the causes of changes in the burden due to population growth, population age structure, and epidemiological changes. Our decomposition analyses draw from methods developed by Das Gupta[13] to provide a computationally tractable solution for isolating drivers of burden changes whereby all combinations of possible pathways are averaged across factors.

## Results

### Global trends

**Fig 1:**
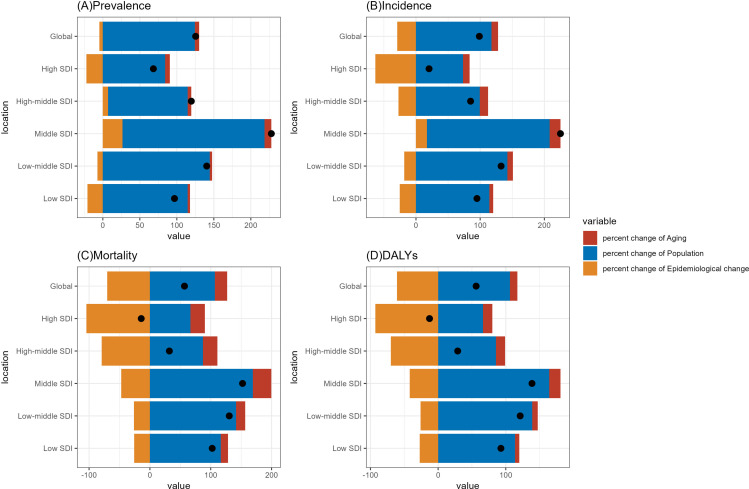
Decomposition of percentage change in the number of ischemic stroke cases, incidence, DALYs, and deaths between 1990 and 2021. DALYs: Disability-Adjusted Life-Years.

**Fig 2:**
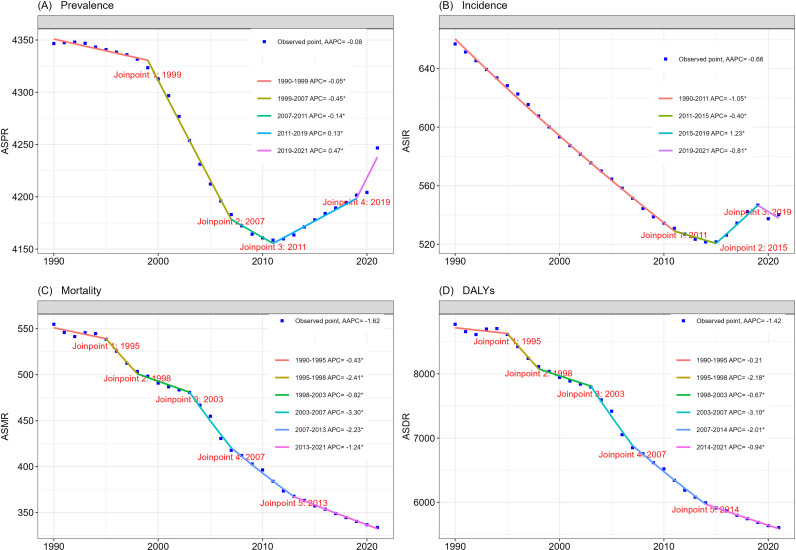
Join-point regression analysis of age-standardized prevalence (A), incidence (B), mortality (C), and DALYs(D) of ischemic stroke worldwide in people aged 60 years and older from 1990 to 2021. DALYs: Disability-Adjusted Life-Years.

Globally, the burden of ischemic stroke in adults ≥ 60 years is considerable in 2021. The number of ischemic stroke survivors in adults ≥ 60 years has risen by 125%, from 20.1 million to 45.52 million, and the number of incidence cases has increased from 2.87 million in 1990 to 5.71 million in 2021, an increase of 98.9%. The significant increase in the prevalence and incidence of ischemic stroke is mainly related to population ageing and the significant increase in the number of people over 60 years of age, with the significant increase in the population over 60 years of age being the main driving force, while epidemiological changes have had the opposite effect (Fig 1, S1, S2 and S5 Tables), this is similar with the description of the global population pyramid (https://ourworldindata.org/age-structure). Although the number of prevalence and incidence is a substantial increase, the number of ASPR and ASIR are decreasing overall, with the ASPR decreasing from 4347 per 100,000 population in 1990–4246 per 100,000 population in 2021, but instead of a continual downward trend, ASPR is showing a downward and then an upward trend, manifesting itself in the form of a decrease from 4347 per 100,000 population in 1990–4158 per 100,000 population in 2011, and then increasing year by year to 4246 per 100,000 population in 2021, especially the AAPC of 2019–2021 has the greatest increase (AAPC:0.47) (Fig 2; S1 and S2 Tables).

Over the same period, the ASIR of ischemic stroke among adults ≥ 60 years declined from 590.7 per 100,000 population in 1990 to 523.5 per 100,000 population in 2021. Similar to the ASPR, the ASIR has continued to decrease from 657 per 100,000 population 1990 to a low of 529 per 100,000 population in 2011, and then has risen annually to 540 per 100,000 population in 2021 (Fig 2). Unlike ASPR and ASIR, ASMR and ASDR showed a decreasing trend from year to year over the period 1990–2021, with AAPC -1.62 and -1.42 going down, respectively. ASMR and ASDR reducing by 39.3% and 36.1%, respectively, over the 32-year period 1990–2021 (Fig 2; S3 Table). Although the number of deaths and DALYs have increased substantially, they have benefited from significant epidemiological changes (Fig 1, S5 Table).

In addition, compared to patients with all stroke conditions, the percentage of Prevalence, Incidence, and DALYs for ischemic stroke generally continued to increase between 1990 and 2021, with the prevalence increasing from 81.67% in 1990 to 83.48% in 2021, and the percentage of Incidence increasing from a low of 64.% in 1998 to The prevalence rate has risen from 81.67% in 1990 to 83.48% in 2021, and the incidence rate has been reduced from the lowest point of 64.6% in 1998 to 69.6%. The mortality rate is placed on a downward and then upward trend (S1 Fig).

Ischemic stroke accounted for all-cause Deaths, Incidence, DALYs, Provance in general into a downward trend, where the all-cause share of Deaths declined from a high of 10.33% in 1990 to 7.4% in 2021, and DALYs declined from 7.79% to 5.72%, and we can see that these two indicators in 2020 and 2021 have the most significant declines (S2 Fig).

### Global trends by sex

From 1990 to 2021, the prevalence and incidence of ischemic stroke increased significantly in both men and women aged ≥60 years worldwide. Number of prevalence: men: from 9.84 million to 22.3 million, an increase of 135.9%; women: from 10.3million to 22.3 million, an increase of 115.4%. Number of incidences: males increased from 1.33 million in 1990 to 2.88 million in 2021, an increase of 115.8%, and females increased from 1.54 million to 2.83 million, an increase of 83.90%(S1 and S2 Tables). Overall, the burden of ischemic stroke disease is higher in men than in women, with ASMR and ASDR indicators 50–65% higher in men than in women(S4 Table).

**Fig 3:**
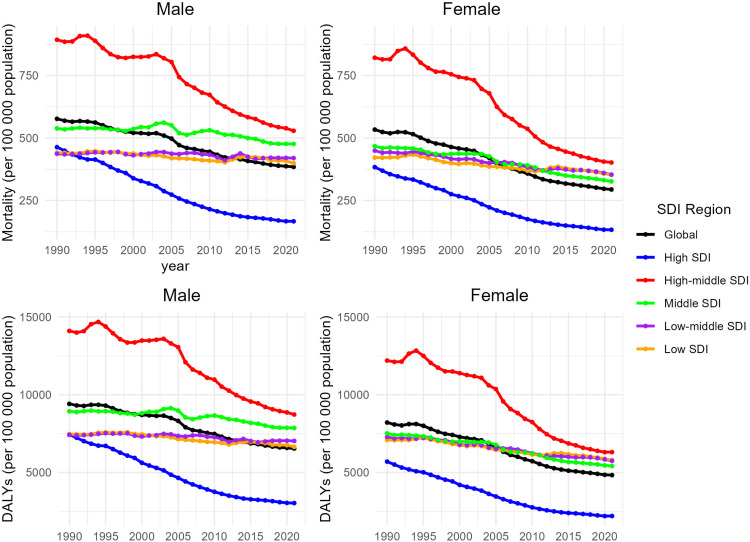
Temporal trend of age-standardized disability-adjusted life years and mortality of ischemic stroke in elderly people from 1990 to 2021 at Sociodemographic Index levels by sex.

**Fig 4:**
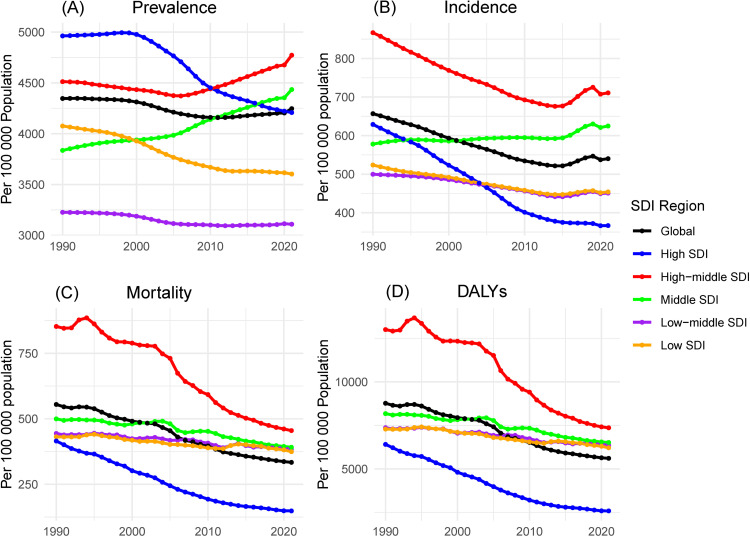
The age-specific number of prevalence (A), incidence (B), mortality (C), and DALYs (D) of ischemic stroke by SDI region and sex in 2021. DALYs: Disability-Adjusted Life-Years.

From 1990 to 2021, ASPR, ASIR, ASDR, and ASMR all decreased among adults ≥ 60 years (Figs 3, 4 and S3), but all decreased less for men than for women. And this trend was not linked with differences in geographic and SDI. In 2021, the burden of ischemic stroke was generally higher for males than for females, except for Sub-Saharan Africa region (S4 Table). Depending on age groups, the ischemic stroke burden of men aged 60–85 years is heavier than that of women, while the opposite is true for those aged 85 years and above(S4 Table).

### Global trends by sociodemographic index

From 1990 to 2021, the burden of ischemic stroke raised the most in the middle SDI. The reason is perhaps that its population over 60 years old is growing the fastest (Fig 1, S5 Table). According to the SDI stratification, in 2021, the highest ASPR in the 60+ population was in High-middle SDI, which was 52% higher than the lowest ASPR in low-middle SDI (4772.83 vs 3109.2) (Fig 4, S1 Table). Between 1990-2021, the ASPR of High-middle SDI and Middle SDI subgroups showed an increasing trend, while the AAPCs of other subgroups were 0.14 and 0.43, respectively. High-middle SDI and Middle SDI subgroups showed an increasing trend in ASPR, with AAPCs of 0.14 and 0.43, respectively, whereas all other subgroups showed varying degrees of decreasing ASPR, with the largest decrease in the High SDI region (-0.53).

**Fig 5:**
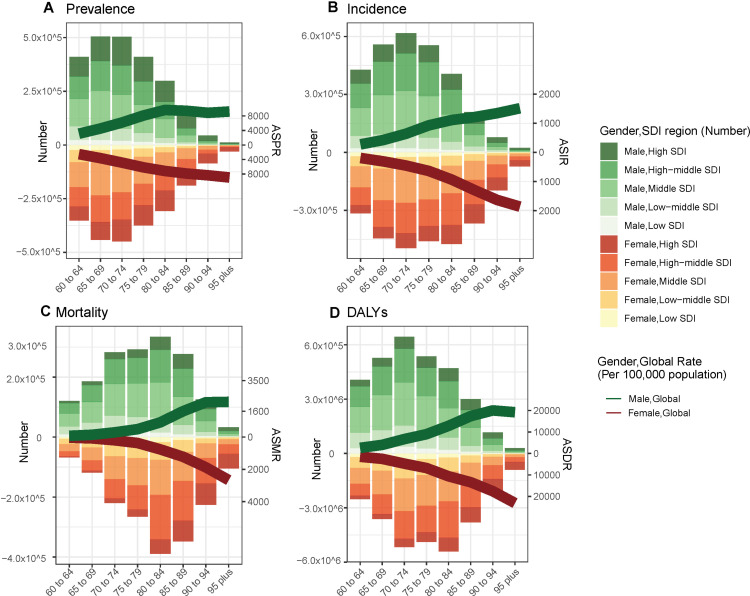
Temporal trend of age-standardized prevalence (A), incidence (B), mortality (C), and DALYs(D) of ischemic stroke in in adults ≥ 60 years from 1990 to 2021 at socio-demographic index levels. DALYs: Disability-Adjusted Life-Years.

The highest ASIR was again in High-middle SDI (Fig 5, S2 Table), which was 97% higher than the lowest High SDI (621 vs 316). Except for Middle SDI, which increased by 0.23% AAPC, all other SDI strata had varying decreases in ASIR, with the largest decrease in High SDI (-1.72%), and all other subgroups had varying decreases ranging from -0.34 to -0.65% (S4 Fig, S3 Table). This trend suggests the adoption of effective prevention and management strategies in High SDI, whereas the burden of ischemic stroke is increasing in older adults in high-middle SDI and middle SDI.

During 1990-2021, ASMR and ASDR in people over 60 years of age showed a decline in all subgroups of SDI, and the trend of decline was dominant the higher the SDI and the lower the ASMR and ASDR (S3 Fig, S3 Table). The largest decreases were observed in the High SDI region with -2.91 and -3.31, respectively, while the smallest decreases were observed in the Low SDI and Low-middle SDI, which ranged from about -0.4 to -0.5. In 2021, ASMR and ASDR are highest for High-middle SDI, at 455 per 100,000 population and 7,361 per 100,000 population, respectively, and lowest for High SDI at 149 per 100,000 population, which was only one-third that of High-middle SDI (S4 Fig, S3 Table). This stark contrast highlights the differences in ischemic stroke rescue measures and secondary prevention. In particular, the sizable difference in DALYs reflects the large differences in quality of life after stroke across SDI regions.

### GBD regional trends

**Fig 6:**
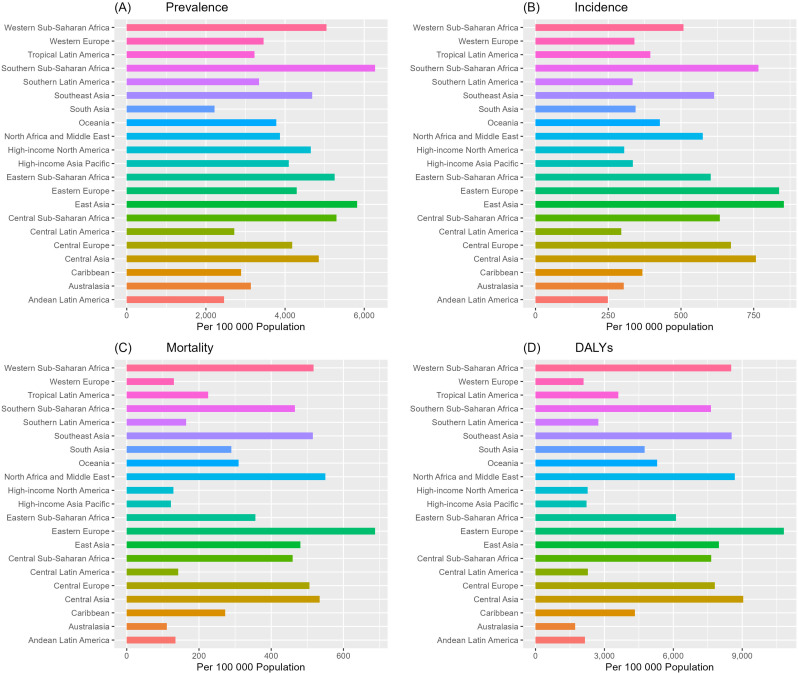
Age-standardized(per 100 000 population) of prevalence (A), incidence (B), mortality (C), and DALYs(D) of 21 GBD regions in 2021. DALYs: Disability-Adjusted Life-Years.

Our study showed that among the 21 GBD regions, the highest prevalence of ischemic stroke among people aged 60 years or older was found in Sub-Saharan Africa and East Asia, respectively Southern Sub-Saharan Africa (6277 per 100,000 population), East Asia (5824 per 100,000 population),Central Sub-Saharan Africa (5298 per 100,000 population), Eastern Sub-Saharan Africa (5255 per 100,000 population), and Western Sub-Saharan Africa(5046 per 100,000 population) (Fig 6, S1 Table). ASPR in these regions are 1.9-2.8 times higher than in South Asia, the lowest region. From 1990-2021, of the 21 GBD regions, only East Asia had an increase in ASPR(AAPC 1.09%), with little change in Southeast Asia and North Africa and Middle East. The ASPR of the remaining 18 regions are decreasing at rates ranging from 0.11% to 1.02% per year, with four regions, High-income Asia Pacific, Southern Latin America, Australasia, and Tropical Latin America, experiencing the fastest decreases of 1% (S5 Fig, S1 Table).

Over the period 1990-2021, the ASDR for adults ≥ 60 years declined at varying rates in most regions (AAPC: 0.20 - 3.9%), with the highest rates of decline in High-income Asia Pacific (AAPC:-3.85) and Western Europe (AAPC:-3.85) and Australasia had the largest decreases in AAPC. Over the same period only Southern Sub-Saharan Africa is up by an AAPC of 0.68. The regions with the highest ASDR in 2021 Eastern Europe, Central Asia, North Africa and Middle East, Southeast Asia, Western Sub-Saharan Africa, Western Europe (AAPC:-3.85) and Australasia (AAPC:-3.85), range from 10819 - 8516 per 100,000 population (Figs 6 and S5, S3 Table).

### National trends

At the level of 204 countries and territories, 16 of the 20 countries with the highest ASPR in 2021 are from the Sub-Saharan Africa region. Botswana(7711.8 per 100,000 population), Ghana(7485.7 per 100,000 population), Madagascar(7029.2 per 100,000 population), Sao Tome and Principe(6689.4 per 100,000 population) and Djibouti(6622.5 per 100,000 population) are among the five countries with the highest ASPR, and are also from the Sub-Saharan Africa region(S1 Table).This finding is consistent with our regional analysis, which identified Sub- Saharan Africa as the region with the highest burden of ischaemic stroke prevalence among older people globally.

**Fig 7:**
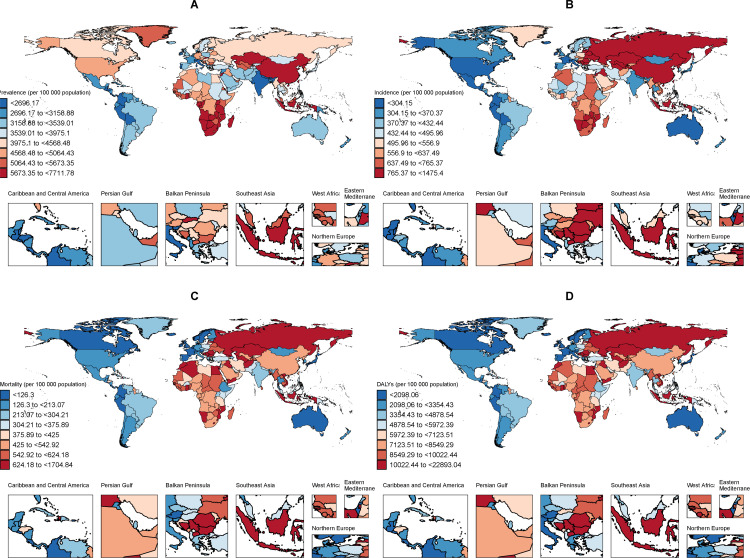
The global disease burden of ischemic stroke for both sexes in 204 countries and territories. Age-standardized rate(per 100 000 population) of prevalence(A), incidence(B), Mortality(C) and DALYs(D). DALYs: Disability-Adjusted Life-Years.

China denotes the country with the largest increase in ASPR and ASIP for ischemic stroke among adults ≥ 60 years, with both AAPC exceeding 1%, as well as the highest number of prevalence and morbidity(S1 and S2 Tables). The main reasons are the aging population and the sharp increase in the number of people over 60 years old (Fig 7, S5 Table). Ischemic stroke is usually regarded as a geriatric disease, and China is now entering an aging society, and the burden of ischemic stroke will inevitably increase in the future, which will require us to pay more attention to the national health care system and the policy preferences for prevention strategies.

As for ASDR, Singapore had the largest drop in ischemic stroke over 60 (AAPC: -5.70%), followed by Portugal (AAPC: -5.60%) and Luxembourg (AAPC: -5.40%). At the same time, we note that the vast majority of the 20 countries with the lowest number of ASDRs and the largest decreases in ASDRs are High SDI regions and High-middle SDI regions. This also corroborates our results on regional burden tendencies. Lesotho, Montenegro and Zimbabwe were the ones with the largest increases in ASDR, with increases of 1.57%, 1.45% and 1.10%, respectively(S3 Table).

We carried out locally weighted linear regression between the corresponding SDI of each country and the four disease burden observation indicators. It was found that when the SDI value exceeded 0.75, the ASIR, ASMR and ASDR values decreased rapidly and significantly, reflecting the huge difference between the ischemic stroke disease burden and different levels of social development, and indirectly reflecting the health inequity trend of the ischemic stroke disease burden (S6 Fig). At the same time, we also use the slope index suggested by the World Health Organization to quantify the trend of ischemic stroke health inequity over time. According to the results of slope index, compared with 1990, the inequities of the four indicators of ASPR, ASIR, ASMR and ASDR in 2021 are significantly expanded, and the underdeveloped regions bear a heavier ischemic stroke burden, and the gap in this burden is more significant over time (S7 Fig).

### Contribution of risk factors to ischemic stroke-related DALYs

We used the PAF to quantify the effect of specific risk factors on ischemic stroke-related DALYs. The study collected information on ischemic stroke-related deaths and DALYs attributable to 23 risk factors. The most notable of these is High systolic blood pressure, with a PAF of up to 60%, far exceeding additional risk factors (S8 and S9 Figs). This is closely followed by High LDL cholesterol and High fasting plasma glucose. This is what we are usually most familiar with the “3High”. This result proposes that we can do much to control blood pressure, lipids, and blood glucose, even though there are an endless variety of medications available today.

We found the risk factor “Household air pollution from solid fuels” to be quite distinctive. This risk factor declines globally from 17 per cent in 1990 to 8.7 per cent in 2021, but we found that the Low-middle SDI and Low SDI and Sub-Saharan Africa remain high at 30–40%. Over the same period, High-middle SDI and Middle SDI decline significantly. “Household air pollution from solid fuels” may be one of the important reasons for our earlier finding that the burden of disease for ischemic stroke is much higher in Sub-Saharan Africa than in other regions, when other risk factors do not differ significantly between regions.

In addition, stratified by gender, we found that, except for the “3H”, other risk factors had different impacts on ischemic stroke-related DALYs. For example, “Alcohol use”, “Smoking”, “Diet high in sodium”, and “Diet high in sodium”.(S9 Fig).

### Projections of future trends

According to the results of the BAPC prediction model, the burden of ischemic stroke will continue to increase considerably in people over 60 years of age (S10 Fig). By 2050, the prevalence of ischemic stroke in people aged 60 years and older will reach 115.7 million, double the 2021 figure of 45.5 million. Similar to the prevalence, the number of incidences, mortality and DALYs in 2050 were 2.29 times, 2.45 times and 2.28 times higher than in 2021, respectively. At the level of age-standardized data, the prevalence among women is gradually increasing, approaching or even overtaking that of men. In all four measures, the burden of ischemic stroke is consistently higher in men compared to women, despite the fact that the gap between men and women appears to be narrowing over time, particularly for ASPR.

## Discussion

Complex differences and trends across regions, SDIs and gender were revealed in our comprehensive assessment of the global burden of ischemic stroke for the period 1990 to 2021. Worldwide incidence and prevalence of Ischemic stroke doubled between 1990 and 2021, and the proportion of Ischemic stroke in stroke and overall prevalence is increasing. Because of the COVID-19 pandemic, stroke has fallen from the third to the fourth leading cause of disability[14].While the number of incidence and prevalence of ischemic stroke in adult ≥ 60 years has increased substantially globally, ASPR and ASIR have shown a downward trend, indicating that the burden of ischaemic stroke has benefited from population growth. However, the accelerating ageing of the population requires our attention and policy support. During the same period, both mortality rates and DALYs for ischemic stroke-related ASR declined dramatically, suggesting that thanks to more than 30 years of advances in health care, including the improvement of the emergency stroke care system[15], measures of revascularization, and the ability to provide resuscitative care in acute and critical care, countless lives have been saved and the quality of their post-stroke survival has improved[16–22]^.^

Furthermore, we found that using the entire age population for calculations did prompt us to underestimate the burden of ischemic stroke. The ASPR, ASIR, ASMR and ASDR for ischemic stroke in the 60+ age-group were five, 5.8, 8.7 and 6.7 times higher than in the all-age population group, respectively (S11 Fig, S1–S3 Tables). Ischemic stroke is an illness highly associated with aging, and using an all-age population to quantify the disease burden would lead us to underestimate the disease burden of ischemic stroke.

While ASPR and ASIR generally declined between 1990 and 2021, we found that both metrics continued to rise after 2011, which had never been mentioned in previous studies. Previous studies mention that the ASPR and ASIR are generally declining from 1990-2021, but do not find an upward trend after 2011[1,4,5,23]. In particular, the prevalence of ischemic stroke increased from 81.67% in 1990 to 83.48% in 2021, with accelerated growth after 2004, powered by an ageing population and rapid lifestyle changes. The incidence increased from 64.43% to 69.64%, with critical turning points in 2004 and 2011, suggesting changing environmental exposures or access to healthcare. Mortality rates followed a fluctuating trajectory, starting at 56.52% in 1990, peaking in 1994, then declining to a historic low in 2004, before recovering to 55.62% in 2021, reflecting a balance between medical developments and population ageing. DALYs fell from 64.3% to 53.64%, with steep declines after 1994 and after 2004, underscoring the success of the health system in reducing stroke-related disability and premature mortality. These turning points are such as to coincide with China’s socio-economic changes, lifestyle modernization and fluctuating environmental risks. Critically, future studies should priorities disentangling these factors particularly the interplay between environmental and socioeconomic factors and conduct region and population specific analyses to refine targeted interventions.

Stratified by sex, we found that the stroke burden was consistently higher in men than in women, and even when ASIR incidence, ASDR, and ASMR were all on a downward trend, the downward trend was lower in men than in women, and this trend is likely to increase further in the future. This is the same as the results of prior studies[24,25]. This tendency is perhaps more relevant to differences in risk factors. It can be seen that smoking and alcohol are more common in men, and the resulting chronic inflammation of the blood vessels can make ischemic stroke more likely to appear in men[25,26].

Stratified by SDI, the highest ASPR, ASIR, ASDR, and ASMR were found in the high-middle SDI and middle SDI regions, whereas the lowest rates were found in the abnormal SDI regions. ischemic stroke burden shows a trend of increasing and then decreasing with the growth of SDI, and ischemic stroke burden decreases sharply after SDI exceeds 0.75. This finding highlights the persistent SDI disparities in the global burden of infrastructure services, and is analogous to previous studies by income disparity[1,4,5]. This trend reflects the wide disparities in the quality of health-care and preventive measures between regions with different levels of development, with the higher the level of societal development, the better the quality of health-care and preventive policies. Surveillance for cardiovascular disease risk factors is largely established in developed regions, and interventions and care are more readily obtainable, so high SDI regions bear the least burden of ischemic stroke[27,28]^.^ In contrast, the higher burden in high-middle SDI and middle SDI regions may be due to multiple factors, including inadequate access to health-care, poor management of risk factors, and inadequate secondary prevention strategies[29,30].

Sub-Saharan Africa has the highest prevalence and mortality rates and bears the highest burden of ischemic stroke. This result is explained by a combination of factors, including genetic susceptibility, risk factors, dietary habits and limitations of the healthcare system[6,31]. Heritable factors, such as the high prevalence of sickle cell trait in some African populations, may also contribute to an elevated risk of stroke[32]. And Sub-Saharan Africa faces significant pressures on health care accessibility and quality, with limited resources for prevention and acute stroke care[33].

In addition, we observed that China is the one with the highest burden of ischemic stroke. China holds the largest increase in ischemic stroke ASPR and ASIP in adults ≥60 years, with an AAPC of more than 1% in both cases, and the highest prevalence and incidence of the disease. Several factors contribute to this unrelenting challenge. Primarily, the rapid urbanization and lifestyle changes accompanying economic development have led to an increased prevalence of risk factors such as obesity, hypertension, and diabetes, all of which contribute to atherosclerosis[23,34]. While treatment options have improved, access to healthcare varies widely across regions and socioeconomic groups, potentially leading to disparities in disease management[35]^.^ Public awareness of cardiovascular health is also relatively low, leading to late-stage diagnoses and poorer health outcomes[23]. As China’s population ages, the burden of ischemic stroke could increase unless targeted measures are taken in order to address the issue. Dealing with the burden of ischemic stroke in China requires a combination of approaches that address the complex relationship between lifestyle, healthcare accessibility, and socioeconomic factors.

We used the PAF to quantify the effect of specific risk factors on ischemic stroke-related DALYs. The study collected data on ischemic stroke-related deaths and DALYs attributable to 23 risk factors. The most noteworthy of these is High systolic blood pressure, with a PAF of up to 60%, far exceeding other risk factors(S8 and S9 Figs). This is intimately followed by High LDL cholesterol and High fasting plasma glucose. This is what we are usually most familiar with the “3H”. This result suggests that we can do much to control blood pressure, lipids, and blood glucose, despite the fact that there are an endless variety of medications available today. In addition, the “3H” affects men and women differently. According to pertinent studies, men have a higher rate of unsatisfactory blood pressure control than women, and men have higher LDL levels compared to women[25]. In addition, it has been shown that atherosclerotic plaques in men are still more prone to rupture than those in women[36], and that men have greater plaque number, plaque area, and plaque size compared with age-, body-mass index-, blood pressure-, cigarette-smoking-, and diabetes-matched women, which is largely attributed to the complex relationship between sex hormones and atherosclerosis and vascular remodeling [37]. Ambient particulate pollution, with a PAF of up to 17%, is the fourth highest risk factor attributable to ischemic stroke-related DALYs. Inhaled nitrogen dioxides (NO_2_) and particulate matter (PM) in air pollution induce oxidative stress in the body, generating reactive oxygen species (ROS) and other pro-inflammatory oxidants. This cascade of oxidative stress subsequently triggers systemic inflammation, damaging vascular endothelial cells, accelerating atherosclerosis and promoting thrombus formation mechanisms that collectively increase the risk of stroke. Notably, epidemiological studies in China have provided support for these links, showing that both chronic exposure to air pollution and acute episodes of high pollution dramatically increase the incidence of ischemic stroke[38,39]. Improving air quality through stricter emissions standards, promoting clean energy sources, and enhancing municipal transportation systems could address the issue of air pollution. Tobacco control policies, such as increasing tobacco taxes, implementing comprehensive smoke-free laws, and expanding cessation services.

We found the risk factor “Household air pollution from solid fuels” to be quite idiosyncratic. This risk factor declines globally from 17% in 1990 to 8.7% in 2021, but we found that the Low-middle SDI and Low SDI and Sub-Saharan Africa remain superior at 30–40%. Over the same period, High-middle SDI and Middle SDI decline markedly. “Household air pollution from solid fuels” may be one of the principal reasons for our earlier finding that the burden of disease for ischemic stroke is much higher in Sub-Saharan Africa than in other regions, when other risk factors do not differ significantly between regions. Ambient particulate matter pollution. Smoking and alcohol use are significant risk factors in ischemic stroke burden in men. Enhancing public education campaigns about the risks of excessive alcohol and tobacco use and their link to stroke could also be operative. Increasing tobacco taxes, implementing comprehensive smoke-free laws, and expanding cessation services may also be helpful measures.

The number of prevalence, morbidities and deaths of ischemic stroke among people over 60 years of age is projected to double by 2050 compared to 2021. In order to decrease the burden of ischaemic stroke, a combination of approaches that address the complex relationship between lifestyle, health care accessibility and socioeconomic factors is needed. First deal with the health-care, public health interventions could be to reduce the prevalence of risk factors. This could include programs promoting physical activity, healthy eating, and smoking cessation, as well as early detection and management of conditions like hypertension and diabetes[24]. Second, free or discounted screening and physical exams for ischemic stroke risk factors can be provided to older adults. Finally, public health education campaigns can help increase awareness of ischemic stroke, encouraging early screening and treatment. Such comprehensive efforts may help lessen the burden of ischemic stroke across various socioeconomic groups.

To summarize, despite declining age-standardized rates, the absolute burden of ischemic stroke remains high, showing considerable variation between regions, countries, and SDI regions. The findings emphasize the need for targeted prevention and treatment strategies that address the specific needs of diverse populations, particularly in high-middle SDI and middle SDI regions. Especially now that ischemic stroke health inequities continue to widen, strengthening health-care systems, promoting healthy lifestyles, and reducing socioeconomic disparities are indispensable steps toward reducing the global burden of ischemic stroke.

This study has some limitations that should be taken into account when interpreting the results. First, the accuracy of the estimates may be influenced by the quality and availability of data sources across countries and regions. The potential for misdiagnosis of ischemic stroke disease is unsatisfactory, most likely due to the limited medical services and epidemiological infrastructure in developing countries. In some low- and middle-income countries, the lack of reliable epidemiological data and the under reporting of stroke cases may lead to an underestimation of the true burden. Second, the GBD methodology relies on numerous assumptions and modeling techniques, which may introduce some uncertainty in the estimates. While the GBD study employs rigorous statistical methods to address these uncertainties, the results should be interpreted as the best available estimates based on the current evidence.

## Supporting information

S1 Fig The changes in the proportion of prevalence cases among ischemic stroke patients aged over 60 years to the overall stroke patients from 1990 to 2021. (TIF)

S2 Fig The age-standardized prevalence, incidence, mortality and DALYs proportion of ischemic stroke to all-cause in patients aged above 60 years from 1990 to 2021. (TIF)

S3 Fig Temporal trend of age-standardized prevalence and Incidence of ischemic stroke in elderly people from 1990 to 2021 at socio-demographic index levels by sex. (TIF)

S4 Fig Average annual percent changes of prevalence(A), incidence(B) mortality(C), and DALYs (D) of ischemic stroke in elderly people aged over 60 years from 1990 to 2021 at global and socio-demographic index levels. (TIF)

S5 Fig Average annual percent changes of Prevalence(A), incidence(B), mortality (C) and DALYs (D) rate of ischemic stroke in elderly people from 204 countries according to the socio-demographic index in 2021. (TIF)

S6 Fig Prevalence (A), incidence (B), mortality (C) and DALYs (D) of ischemic stroke in adults ≥60 years in 21 GBD regions in 2021, according to sociodemographic indices. (TIF)

S7 Fig Slope index of prevalence (A), incidence (B), mortality (C) and DALYs (D) of ischemic stroke among the elderly in 204 countries in 2021 according to socio-demographic index. (TIF)

S8 Fig Population attributable fraction of DALYs Attributable to identifiable risk factors, 1990(A) and 2021(B). (TIF)

S9 Fig Population attributable fraction of DALYs Attributable to definite risk factors, Male(A) and Female(B). (TIF)

S10 Fig BAPC prediction model for prevalence (A), morbidity (B), mortality (C), and disability-adjusted life years (D). (TIF)

S11 Fig Comparison of prevalence (A), morbidity (B), mortality (C) and DALYs in the adult group adults ≥60 years with the all-age group. (TIF)

S1 Table The prevalence cases and ASPR of ischemic stroke in 1990 and 2021 and its trends. (DOCX)

S2 Table The incidence cases and ASIR of ischemic stroke in 1990 and 2021 and its trends. (DOCX)

S3 Table The ASDR and ASMR of ischemic stroke in 1990 and 2021 and its trends. (DOCX)

S4 Table ASPR, ASIP, ASDR and ASMR of Ischemic stroke in 2021 by sex, global, regional, and national, 2021. (DOCX)

S5 Table Global and regional differences in morbidity, mortality, DALYs and demographic and epidemiological changes. (DOCX)

## References

[1] GBD 2019 Stroke Collaborators. Global, regional, and national burden of stroke and its risk factors, 1990-2019: a systematic analysis for the Global Burden of Disease Study 2019. Lancet Neurol. 2021;20(10):795–820. 34487721 10.1016/S1474-4422(21)00252-0 PMC8443449

[2] Hilkens NA, Casolla B, Leung TW, de Leeuw FE. Stroke. Lancet Lond Engl; 2024;403(10446):2820–36. 10.1016/S0140-6736(24)00642-1 38759664

[3] Bengtsson VW, Persson GR, Berglund J, Renvert S. Carotid calcifications in panoramic radiographs are associated with future stroke or ischemic heart diseases: a long-term follow-up study. Clin Oral Investig. 2019;23(3):1171–9. 10.1007/s00784-018-2533-8 29967974

[4] Feigin VL, Krishnamurthi R, Parmar P, Norrving B, Mensah GA, Bennett DA, et al. Update on the global burden of ischaemic and haemorrhagic stroke in 1990–2013: the GBD 2013 study. Neuroepidemiology. 2015;45(3):161–76. 26505981 10.1159/000441085 PMC4633282

[5] GBD 2016 Neurology Collaborators. Global, regional, and national burden of stroke, 1990–2016: a systematic analysis for the Global Burden of Disease Study 2016. Lancet Neurol. 2019;18(5):439–58. 30871944 10.1016/S1474-4422(19)30034-1 PMC6494974

[6] Ding Q, Liu S, Yao Y, Liu H, Cai T, Han L. Global, regional, and national burden of ischemic stroke, 1990-2019. Neurology. 2022;98(3):e279–90. 34911748 10.1212/WNL.0000000000013115

[7] Cheng Z, Lv J, Guo H, Liu X. Global, regional, and national burden of ischemic stroke, 1990–2021: an analysis of data from the global burden of disease study 2021. eClinicalMedicine. 2024;75:102758. 39157811 10.1016/j.eclinm.2024.102758 PMC11327951

[8] Feigin VL, Abate MD, Abate YH, Abd ElHafeez S, Abd-Allah F, Abdelalim A, et al. Global, regional, and national burden of stroke and its risk factors, 1990–2021: a systematic analysis for the Global Burden of Disease Study 2021. Lancet Neurol. 2024;23(10):973–1003. 39304265 10.1016/S1474-4422(24)00369-7 PMC12254192

[9] Collaborators G. Global burden and strength of evidence for 88 risk factors in 204 countries and 811 subnational locations, 1990–2021: a systematic analysis for the Global Burden of Disease Study 2021. Lancet Lond Engl. 2024;403(10440):2162. 10.1016/S0140-6736(24)00933-4 PMC11120204 38762324

[10] GBD 2021 Diseases and Injuries Collaborators. Global incidence, prevalence, years lived with disability (YLDs), disability-adjusted life-years (DALYs), and healthy life expectancy (HALE) for 371 diseases and injuries in 204 countries and territories and 811 subnational locations, 1990-2021: a systematic analysis for the global burden of disease study 2021. Lancet Lond Engl. 2024;403(10440):2133–61. 10.1016/S0140-6736(24)00757-8 PMC11122111 38642570

[11] Lee TCK, Dean CB, Semenciw R. Short-term cancer mortality projections: a comparative study of prediction methods. Stat Med. 2011;30(29):3387–402. 21965149 10.1002/sim.4373

[12] Jürgens V, Ess S, Cerny T, Vounatsou P. A bayesian generalized age-period-cohort power model for cancer projections. Stat Med. 2014;33(26):4627–36. 24996118 10.1002/sim.6248

[13] Das Gupta P. Standardization and decomposition of rates from cross-classified data. Genus. 1994;50(3–4):171–96. 12319256

[14] GBD 2021 Diseases and Injuries Collaborators. Global incidence, prevalence, years lived with disability (YLDs), disability-adjusted life-years (DALYs), and healthy life expectancy (HALE) for 371 diseases and injuries in 204 countries and territories and 811 subnational locations, 1990-2021: a systematic analysis for the global burden of disease study 2021. Lancet Lond Engl. 2024;403(10440):2133–61. 10.1016/S0140-6736(24)00757-8 PMC11122111 38642570

[15] Hubert GJ, Hubert ND, Maegerlein C, Kraus F, Wiestler H, Müller-Barna P, et al. Association between use of a flying intervention team vs patient interhospital transfer and time to endovascular thrombectomy among patients with acute ischemic stroke in Nonurban Germany. JAMA. 2022;327(18):1795–805. 35510389 10.1001/jama.2022.5948 PMC9092197

[16] Tsivgoulis G, Katsanos AH, Sandset EC, Turc G, Nguyen TN, Bivard A, et al. Thrombolysis for acute ischaemic stroke: current status and future perspectives. Lancet Neurol. 2023;22(5):418–29. 36907201 10.1016/S1474-4422(22)00519-1

[17] Widimsky P, Snyder K, Sulzenko J, Hopkins LN, Stetkarova I. Acute ischaemic stroke: recent advances in reperfusion treatment. Eur Heart J. 2022;44(14):1205–15. 10.1093/eurheartj/ehac684 PMC10079392 36477996

[18] Thomalla G, Simonsen CZ, Boutitie F, Andersen G, Berthezene Y, Cheng B, et al. MRI-guided thrombolysis for stroke with unknown time of onset. N Engl J Med. 2018;379(7):611–22. 29766770 10.1056/NEJMoa1804355

[19] Campbell BCV, Ma H, Ringleb PA, Parsons MW, Churilov L, Bendszus M, et al. Extending thrombolysis to 4·5-9 h and wake-up stroke using perfusion imaging: a systematic review and meta-analysis of individual patient data. Lancet. 2019;394(10193):139–47. 31128925 10.1016/S0140-6736(19)31053-0

[20] Thomalla G, Boutitie F, Ma H, Koga M, Ringleb P, Schwamm LH, et al. Intravenous alteplase for unknown time of onset stroke guided by advanced imaging: a systematic review and meta-analysis of individual patient data. Lancet Lond Engl. 2020;396(10262):1574–84. 10.1016/S0140-6736(20)32163-2 PMC7734592 33176180

[21] Ye S, Hu S, Lei Z, Li Z, Li W, Sui Y, et al. Shenzhen stroke emergency map improves access to rt-PA for patients with acute ischaemic stroke. Stroke Vasc Neurol. 2019;4(3):115–22. 31709116 10.1136/svn-2018-000212 PMC6812643

[22] Ma H, Campbell BCV, Parsons MW, Churilov L, Levi CR, Hsu C, et al. Thrombolysis guided by perfusion imaging up to 9 hours after onset of stroke. N Engl J Med. 2019;380(19):1795–803. 31067369 10.1056/NEJMoa1813046

[23] Chen W, Li Z, Zhao Y, Chen Y, Huang R. Global and national burden of atherosclerosis from 1990 to 2019: trend analysis based on the Global Burden of Disease Study 2019. Chin Med J (Engl). 2023;136(20):2442–50. 37677929 10.1097/CM9.0000000000002839 PMC10586830

[24] Truyen TTTT, Vo NLY, Vo QP, Phan TC, Le PNB, Nguyen HDT, et al. Burden and risk factors of stroke in vietnam from 1990 to 2021 - a systematic analysis from global burden disease 2021. J Stroke Cerebrovasc Dis Off J Natl Stroke Assoc. 2025;34(3):108241. 10.1016/j.jstrokecerebrovasdis.2025.108241 39826583

[25] Man JJ, Beckman JA, Jaffe IZ. Sex as a biological variable in atherosclerosis. Circ Res. 2020;126(9):1297–319. 32324497 10.1161/CIRCRESAHA.120.315930 PMC7185045

[26] Libby P, Loscalzo J, Ridker P, Farkouh ME, Hsue PY, Fuster V, et al. Inflammation, immunity, and infection in atherothrombosis: JACC review topic of the week. J Am Coll Cardiol. 2018;72(17):2071–81. 30336831 10.1016/j.jacc.2018.08.1043 PMC6196735

[27] Use of secondary prevention drugs for cardiovascular disease in the community in high-income, middle-income, and low-income countries (the PURE Study): a prospective epidemiological survey. Lancet. 2011;378(9798):1231–43. 21872920 10.1016/S0140-6736(11)61215-4

[28] Norrving B, Kissela B. The global burden of stroke and need for a continuum of care. Neurology. 2013;80(3 Suppl 2):S5–12. 23319486 10.1212/WNL.0b013e3182762397 PMC12443346

[29] Chimatiro GL, Rhoda AJ. Scoping review of acute stroke care management and rehabilitation in low and middle-income countries. BMC Health Serv Res. 2019;19:789. 31684935 10.1186/s12913-019-4654-4 PMC6829977

[30] Pandian JD, Gall SL, Kate MP, Silva GS, Akinyemi RO, Ovbiagele BI, et al. Prevention of stroke: a global perspective. Lancet. 2018;392(10154):1269–78. 30319114 10.1016/S0140-6736(18)31269-8

[31] Abissegue G, Yakubu SI, Ajay AS, Niyi-Odumosu F. A systematic review of the epidemiology and the public health implications of stroke in Sub-Saharan Africa. J Stroke Cerebrovasc Dis. 2024;33(8):107733. 38663647 10.1016/j.jstrokecerebrovasdis.2024.107733

[32] Caughey MC, Derebail VK, Key NS, Reiner AP, Gottesman RF, Kshirsagar AV, et al. Thirty-year risk of ischemic stroke in individuals with sickle cell trait and modification by chronic kidney disease: the atherosclerosis risk in communities (ARIC) study. Am J Hematol. 2019;94(12):1306–13. 31429114 10.1002/ajh.25615 PMC6858511

[33] Catley D, Puoane T, Tsolekile L, Resnicow K, Fleming K, Hurley EA, et al. Adapting the diabetes prevention program for low and middle-income countries: protocol for a cluster randomised trial to evaluate ‘Lifestyle Africa.’ BMJ Open. 2019;9(11):e031400. 10.1136/bmjopen-2019-031400 PMC6858109 31719084

[34] Yusuf S, Reddy S, Ounpuu S, Anand S. Global burden of cardiovascular diseases: part I: general considerations, the epidemiologic transition, risk factors, and impact of urbanization. Circulation. 2001;104(22):2746–53. 11723030 10.1161/hc4601.099487

[35] Yip W, Hsiao W. Harnessing the privatisation of China’s fragmented health-care delivery. Lancet Lond Engl. 2014;384(9945):805–18. 10.1016/S0140-6736(14)61120-X PMC7159287 25176551

[36] Yahagi K, Davis HR, Arbustini E, Virmani R. Sex differences in coronary artery disease: pathological observations. Atherosclerosis. 2015;239(1):260–7. 25634157 10.1016/j.atherosclerosis.2015.01.017

[37] Halvorsen DS, Johnsen SH, Mathiesen EB, Njølstad I. The association between inflammatory markers and carotid atherosclerosis is sex dependent: the Tromsø Study. Cerebrovasc Dis. 2009;27(4):392–7. 19276622 10.1159/000207443

[38] Cai M, Lin X, Wang X, Zhang S, Qian ZM, McMillin SE, et al. Ambient particulate matter pollution of different sizes associated with recurrent stroke hospitalization in China: a cohort study of 1.07 million stroke patients. Sci Total Environ. 2023;856 (Pt 2):159104. 36208745 10.1016/j.scitotenv.2022.159104

[39] Jiang D, Wang L, Han X, Pan Z, Wang Z, Wang Y, et al. Short-term effects of ambient oxidation, and its interaction with fine particles on first-ever stroke: a national case-crossover study in China. Sci Total Environ. 2024;907:168017. 37879462 10.1016/j.scitotenv.2023.168017

