# Supplementary figures and images for: Global burden of ischemic stroke in adults aged 60 years and older from 1990 to 2021: Population-based study
Source: PLoS One. 2025 May 5;20(5):e0322606. doi: 10.1371/journal.pone.0322606 (PMC12052125; doi:10.1371/journal.pone.0322606)

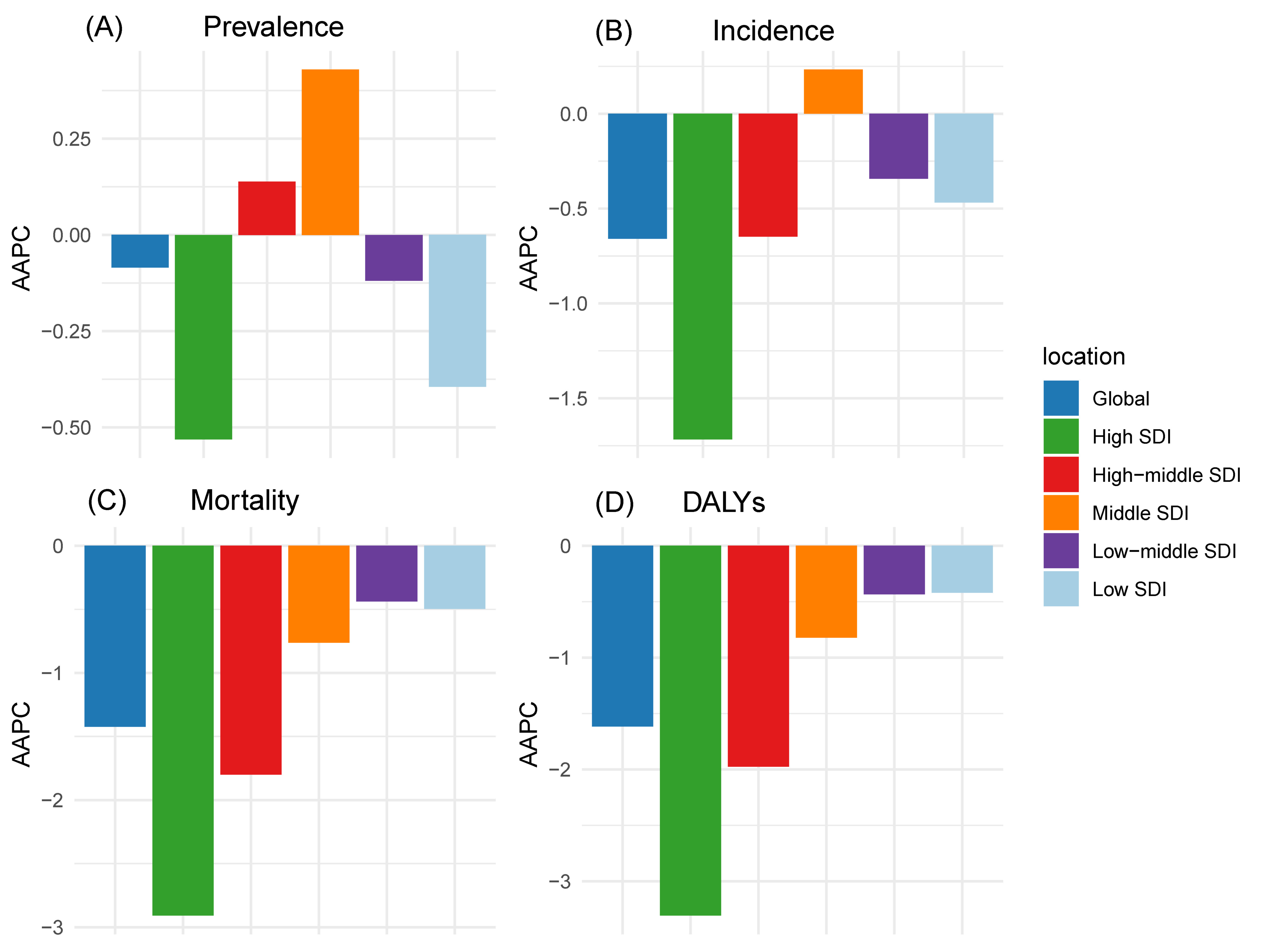

Supplement: S4 Fig — (TIF) [file pone.0322606.s004.tif]

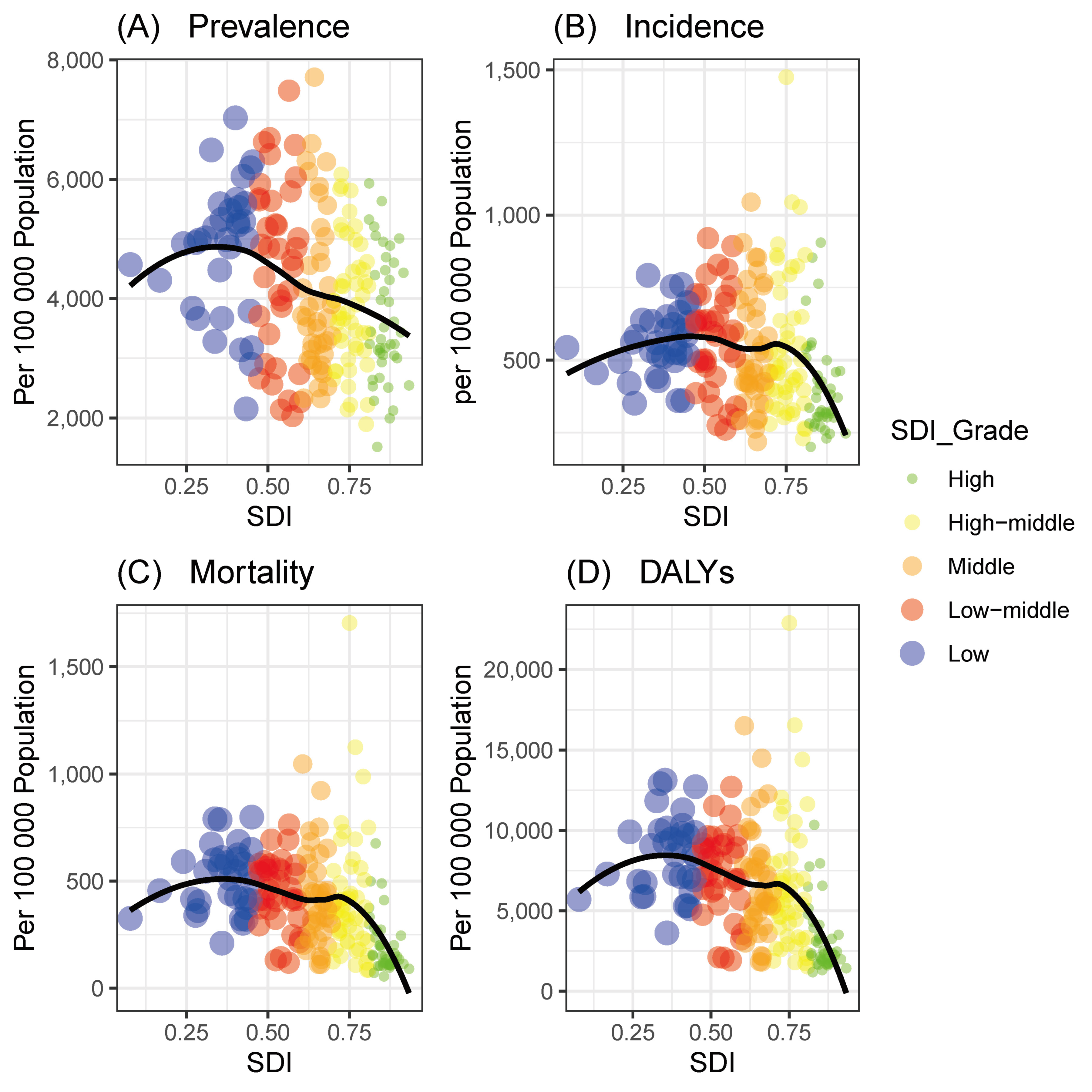

Supplement: S6 Fig — (TIF) [file pone.0322606.s006.tif]

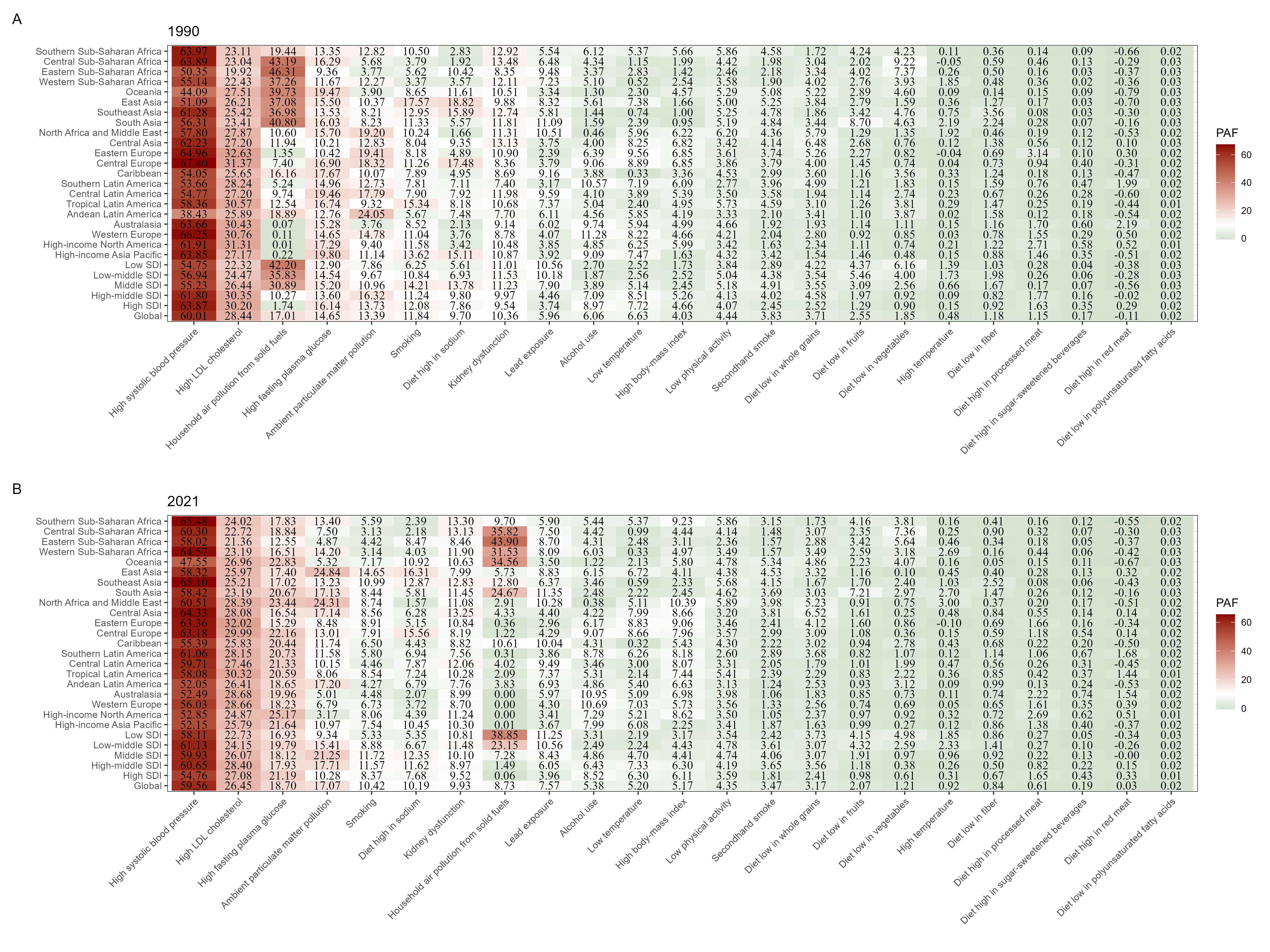

Supplement: S8 Fig — (TIF) [file pone.0322606.s008.tif]

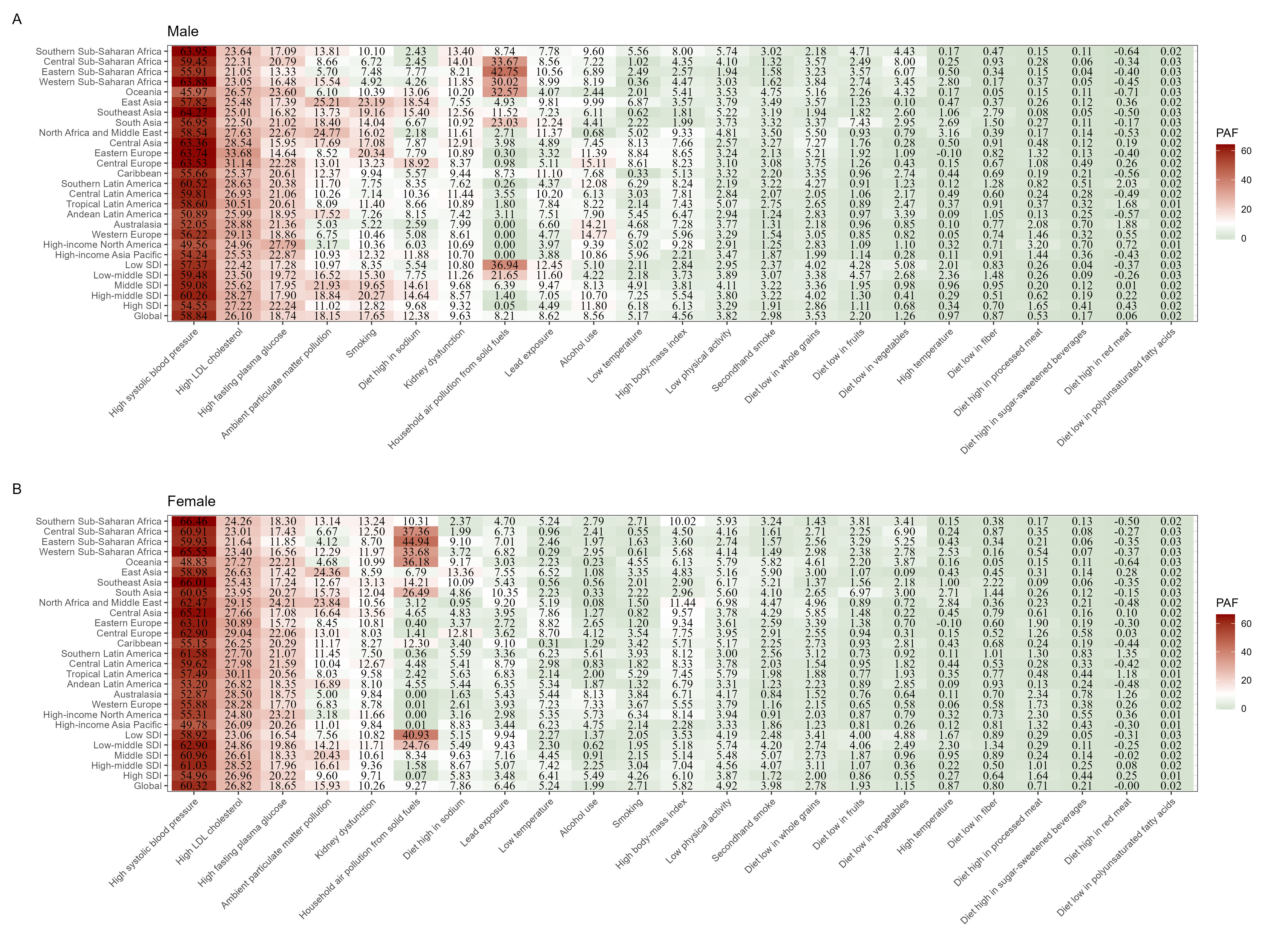

Supplement: S9 Fig — (TIF) [file pone.0322606.s009.tif]

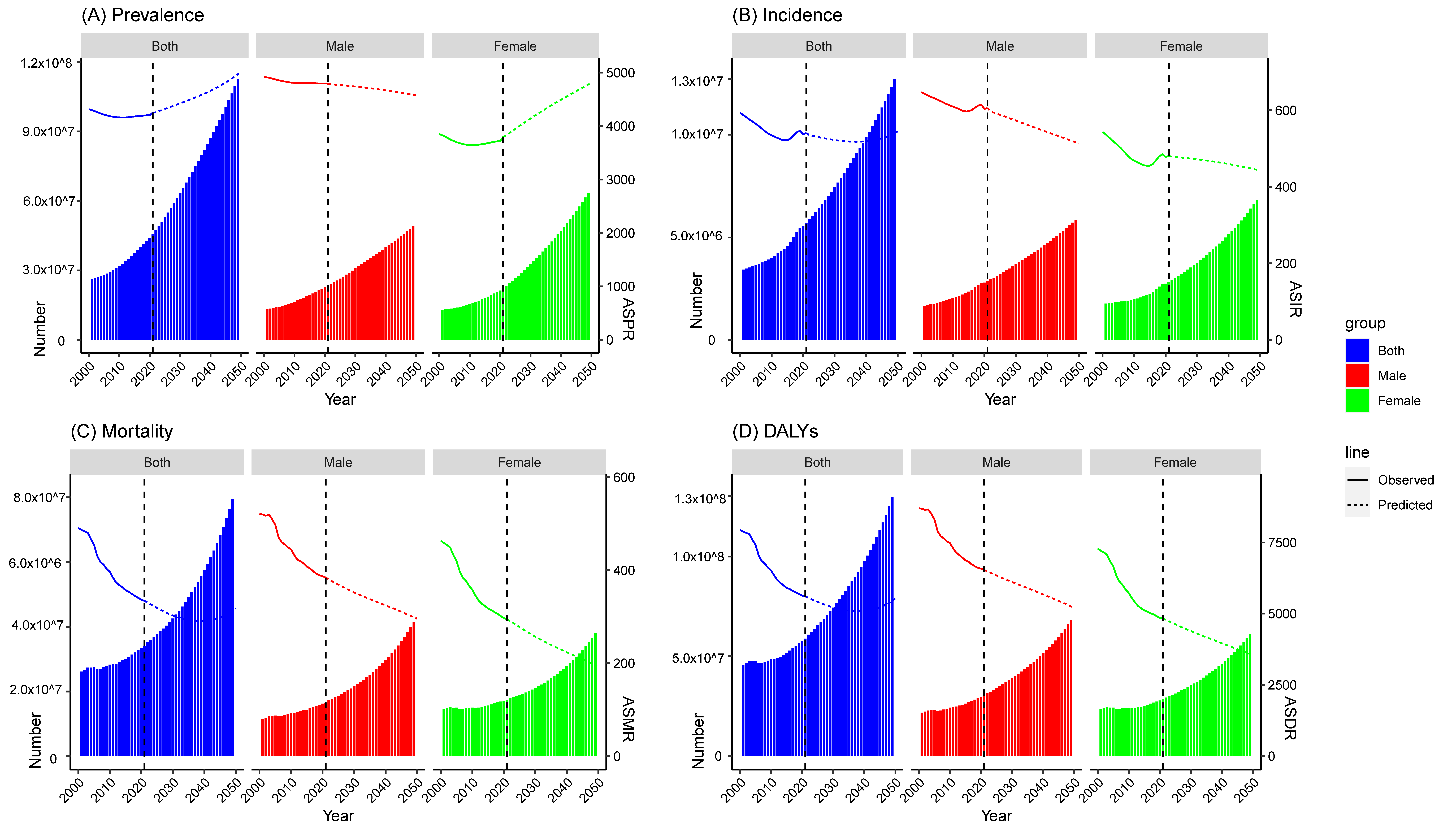

Supplement: S10 Fig — (TIF) [file pone.0322606.s010.tif]

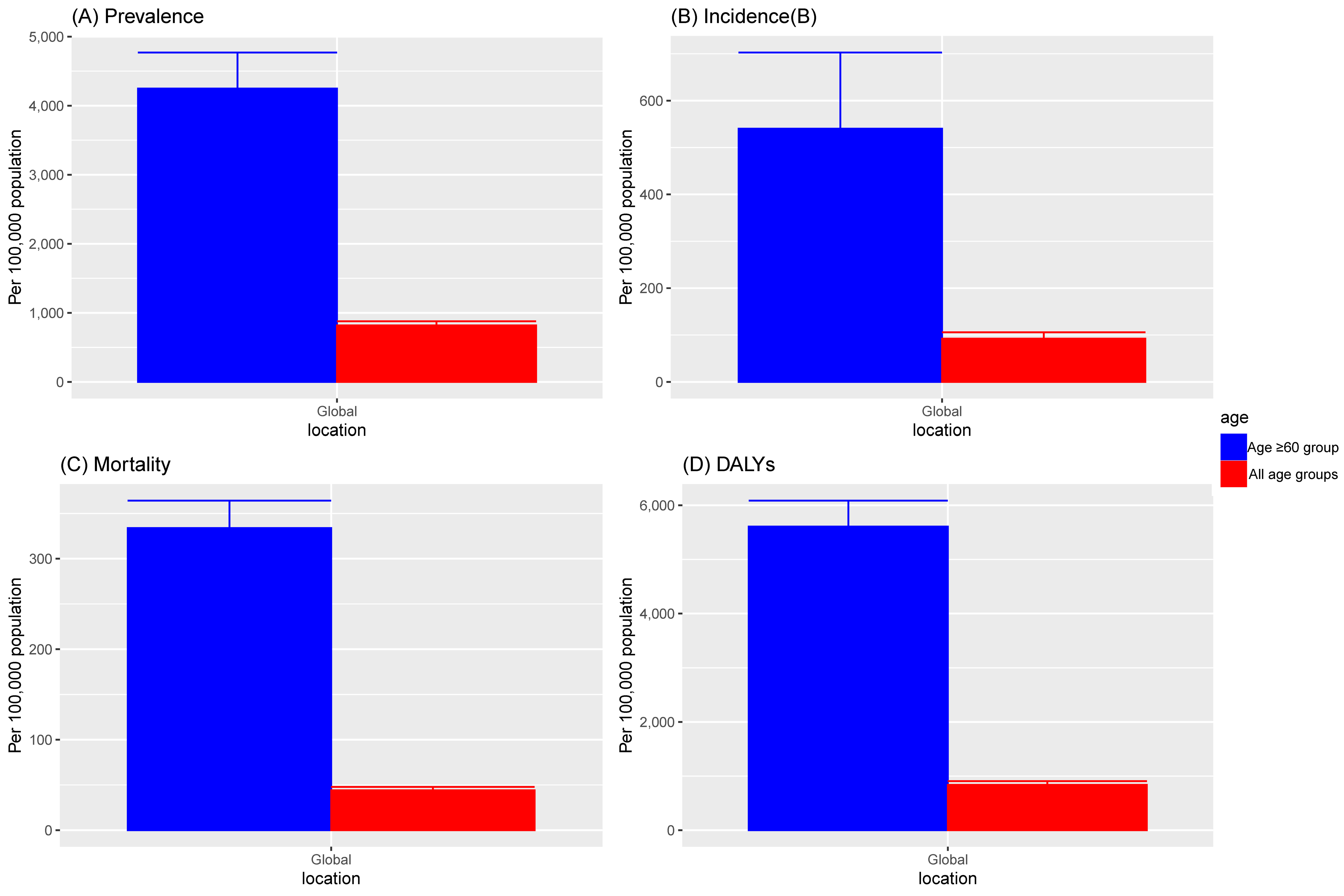

Supplement: S11 Fig — (TIF) [file pone.0322606.s011.tif]
