# Supplementary material for: Global burden of ischemic stroke in adults aged 60 years and older from 1990 to 2021: Population-based study
Source: PLoS One. 2025 May 5;20(5):e0322606. doi: 10.1371/journal.pone.0322606 (PMC12052125; doi:10.1371/journal.pone.0322606)
Supplement: S1 Table — (DOCX) [file pone.0322606.s012.docx]

Table 1. The prevalence cases and ASPR of ischemic stroke in 1990 and 2021 and its trends.

|  | Prevalence | | | | |
| --- | --- | --- | --- | --- | --- |
|  | 1990 counts  （95%UI） | 2021 counts  （95%UI） | 1990 ASR  （95%UI） | 2021ASR  （95%UI） | AAPC（95%CI） |
| Global | | | | | |
| Age ≥60 group | 20198734 (17923234.4-22757196.9) | 45524895.8 (40453360.5-51105265.9) | 4246.6 (3850.6-4904.9) | 4246.7 (3773-4768.7) | -0.08 (-0.1, -0.07) |
| All age groups | 34668041.37 (32153636.82-37171587.51) | 69944884.82 (64788695.13-75009602.78) | 849.49 (785.92-913.25) | 819.47 (760.26-878.71) | -0.12 (-0.14, -0.11) |
| Sex | | | | | |
| Male | 9840865.4 (8710515.7-11111803.9) | 23212809.4 (20720868.5-25985058.4) | 4881.9 (4313.9-5523) | 4792 (4280.2-5363.1) | -0.06 (-0.08, -0.05) |
| Female | 10357868.6 (9177798.6-11681817.4) | 22312086.4 (19707058.2-25226719.6) | 3953.2 (3496.7-4464.8) | 3795.1 (3352.7-4290.1) | -0.15 (-0.17, -0.13) |
| SDI quintiles | | | | | |
| High SDI | 3735372.3 (3346862.2-4155961.9) | 5750733.5 (5209434.1-6335899) | 4232.5 (3789-4712.6) | 3511 (3185.7-3861.8) | -0.53 (-0.55, -0.52) |
| High-middle SDI | 5537561.8 (4864178.7-6278898.1) | 12158882 (10718361.6-13765892.9) | 4512.5 (3956-5132.8) | 4772.8 (4206.6-5405.1) | 0.14 (0.12, 0.16) |
| Middle SDI | 4313423.9 (3661197.6-5024633.5) | 14136145 (12294017.6-16241670.4) | 3835.6 (3242.8-4489.2) | 4434.9 (3850.7-5105.2) | 0.43 (0.39, 0.47) |
| Low-middle SDI | 2141160.2 (1826917.2-2475217.1) | 5146637.7 (4488027.4-5835297.5) | 3226.3 (2736.9-3756.5) | 3109.2 (2698.9-3545.2) | -0.12 (-0.14, -0.1) |
| Low SDI | 963380.4 (851913.4-1081860.2) | 1896184.2 (1707559.7-2093393.5) | 4075.8 (3580.6-4610) | 3603.3 (3223-4005.6) | -0.39 (-0.41, -0.38) |
| Age group | | | | | |
| 60-64years | 4152754.6 (3668690.3-4690080.2) | 7623259.5 (6782072.5-8525689) | 2585.6 (2284.2-2920.2) | 2381.9 (2119.1-2663.9) | -0.27 (-0.31 to -0.24) |
| 65-69years | 4486383.8 (3985567.3-5005852.6) | 9472508.3 (8431885.4-10591034.8) | 3629.5 (3224.3-4049.7) | 3434 (3056.8-3839.5) | -0.2 (-0.24 to -0.15) |
| 70-74years | 4105132.9 (3620756.4-4661894.9) | 9534459.1 (8414541.2-10750308.4) | 4848.9 (4276.8-5506.5) | 4632 (4087.9-5222.7) | -0.16 (-0.19 to -0.13) |
| 75-79years | 3712316.5 (3342488.9-4161601.5) | 7855746.2 (7038306.9-8772089.7) | 6030.8 (5430-6760.7) | 5956.5 (5336.7-6651.3) | -0.05 (-0.08 to -0.02) |
| 80-84years | 2371833.6 (2126345.7-2662637.8) | 6070884.2 (5459064.7-6785463.4) | 6704.7 (6010.7-7526.7) | 6931.6 (6233-7747.5) | 0.09 (0.04 to 0.15) |
| 85-90years | 1010931.9 (878631.9-1149011.7) | 3264541.4 (2880183.9-3698780) | 6690 (5814.5-7603.8) | 7140 (6299.4-8089.8) | 0.19 (0.12 to 0.26) |
| 90-94years | 289239.6 (243595.5-340844.5) | 1287955.8 (1097929-1489309.8) | 6749.8 (5684.6-7954) | 7199.6 (6137.3-8325.1) | 0.18 (0.11 to 0.26) |
| 95+years | 70141.1 (57158.4-85273.6) | 415541.5 (349376.8-492590.6) | 6889.5 (5614.3-8375.9) | 7624.2 (6410.2-9037.8) | 0.3 (0.24 to 0.36) |
| GBD Region | | | | | |
| Andean Latin America | 66497.4 (61238.7-71738) | 174578.6 (162697.6-186739.7) | 2892.5 (2657.9-3127.5) | 2456.9 (2288.7-2629.5) | -0.53 (-0.54, -0.52) |
| Australasia | 131853.3 (124340-140176.7) | 230198.5 (216621.4-244146.6) | 4278.1 (4027.9-4553.5) | 3133.6 (2950.8-3321.5) | -1 (-1.05, -0.94) |
| Caribbean | 96888 (89465.3-105129.6) | 194326 (180109-209030.8) | 3032.4 (2792.9-3298.4) | 2886.8 (2677-3103.8) | -0.16 (-0.17, -0.14) |
| Central Asia | 278155.2 (254553.4-302325.9) | 455040.8 (423737-488579.5) | 5019.8 (4582-5474.9) | 4854 (4495.6-5244.1) | -0.11 (-0.12, -0.09) |
| Central Europe | 962597 (864873-1070728.8) | 1280160.5 (1161539.3-1406675.7) | 5039.9 (4512.9-5626.3) | 4180.7 (3795.1-4593) | -0.59 (-0.62, -0.56) |
| Central Latin America | 324847.1 (290564.1-361623.9) | 816842 (735119.8-906714.7) | 3563.8 (3181.6-3973.8) | 2716.1 (2444.5-3015.3) | -0.88 (-0.9, -0.86) |
| Central Sub-Saharan Africa | 126490.3 (115577.4-137789.8) | 267615.7 (247982.7-287627.2) | 5873 (5302.9-6461.8) | 5298 (4861.6-5742.8) | -0.33 (-0.35, -0.32) |
| East Asia | 3865573.9 (3206183.9-4610280.1) | 15717508.9 (13445296.8-18253632.4) | 4089.3 (3377.8-4906.2) | 5823.7 (4976.5-6773) | 1.09 (1.04, 1.15) |
| Eastern Europe | 1714390.9 (1453012.6-1989998.9) | 2061210.9 (1764370.3-2378273.9) | 4642.7 (3928.9-5396.2) | 4294.6 (3673.1-4958.6) | -0.25 (-0.3, -0.2) |
| Eastern Sub-Saharan Africa | 417692.8 (372929.9-464542.5) | 870516.8 (790985.6-955628.7) | 5578.8 (4948.5-6246.9) | 5254.9 (4746.6-5807.2) | -0.19 (-0.21, -0.18) |
| High-income Asia Pacific | 1397386.1 (1231248.6-1572713.3) | 2676401.5 (2386616.4-2995735.2) | 5646.5 (4963.1-6367.6) | 4094.1 (3663-4567.2) | -1.02 (-1.06, -0.99) |
| High-income North America | 2374979.8 (2042405.6-2725452) | 4169625.4 (3692403.2-4664781.3) | 5009.7 (4305.5-5752.3) | 4652 (4121.5-5203.8) | -0.25 (-0.35, -0.16) |
| North Africa and Middle East | 732987 (654810.7-812950.5) | 1950456.2 (1787824.6-2119418.1) | 3865.1 (3430.5-4317.2) | 3868.3 (3529-4227.6) | 0.01 (-0.01, 0.03) |
| Oceania | 10916 (10027.1-11808.5) | 25875.2 (24087.5-27670.2) | 4081.5 (3718.3-4454.8) | 3779.1 (3501.8-4061) | -0.25 (-0.26, -0.24) |
| South Asia | 1496967.8 (1206691-1808746.5) | 3834365 (3180653.4-4539312.8) | 2414.9 (1932.4-2945.5) | 2220.5 (1829.8-2646.7) | -0.26 (-0.3, -0.21) |
| Southeast Asia | 1266298.4 (1103535.9-1438281.2) | 3507249.3 (3117323.3-3917277.3) | 4607.4 (3995.8-5264.3) | 4685.2 (4154.5-5248.9) | 0.05 (0.05, 0.06) |
| Southern Latin America | 265870.4 (247023.1-285856) | 380833.2 (357226.2-405572.7) | 4581.2 (4247.2-4935.2) | 3342.9 (3136.2-3559.4) | -1 (-1.03, -0.98) |
| Southern Sub-Saharan Africa | 199385.7 (169232.3-232707.3) | 384464.9 (332640.6-442216.7) | 6796.3 (5753.2-7959) | 6277 (5405.8-7242.3) | -0.26 (-0.28, -0.24) |
| Tropical Latin America | 458642.6 (385747.9-541191.2) | 1020663.2 (868404.1-1190045.7) | 4381 (3674.8-5188.8) | 3227.8 (2744-3766.4) | -0.98 (-1.01, -0.94) |
| Western Europe | 3506596.7 (3208248.2-3821670.2) | 4512389.8 (4226119.3-4812944.6) | 4468.3 (4083.3-4876.3) | 3458.6 (3241.4-3685.7) | -0.8 (-0.83, -0.76) |
| Western Sub-Saharan Africa | 503717.9 (444107.8-568852.6) | 994573.3 (896388.7-1100509.1) | 5326.3 (4667.7-6052.8) | 5046.9 (4522.4-5619.6) | -0.17 (-0.18, -0.17) |
| 204 countries and regions | | | | | |
| Afghanistan | 29190.2 (25237.6-33240.8) | 27471.1 (24366.1-30766) | 3410.2 (2923.1-3915.8) | 3282.6 (2909.3-3683.1) | -0.12 (-0.15, -0.1) |
| Albania | 7259.2 (6621.7-7886.1) | 16483.9 (15061.1-17919.6) | 3139.9 (2849.5-3427.1) | 2784.6 (2534.3-3038.3) | -0.36 (-0.4, -0.32) |
| Algeria | 69427.5 (62512-76414.3) | 196080.2 (180894.3-211994.8) | 4732.1 (4224.1-5263.4) | 4792.2 (4397-5207.7) | 0.05 (0.03, 0.07) |
| American Samoa | 109.7 (100.9-118.6) | 217.7 (203-233.1) | 5092.5 (4657.9-5531.6) | 4213.3 (3912.6-4531.1) | -0.61 (-0.62, -0.6) |
| Andorra | 237.6 (216.7-259.5) | 495.5 (454.4-537.4) | 3364.3 (3055.7-3685.6) | 2509.4 (2305.8-2716.4) | -0.94 (-0.98, -0.89) |
| Angola | 23212.6 (21319.9-25197.9) | 66048.3 (61339.8-70759) | 6671.6 (6065.6-7310.6) | 6293.1 (5804.7-6795.4) | -0.19 (-0.19, -0.18) |
| Antigua and Barbuda | 234.5 (213.6-257.4) | 360.4 (333.3-388.3) | 3268 (2983.4-3579.1) | 2799 (2577.8-3027.8) | -0.5 (-0.51, -0.48) |
| Argentina | 184261.7 (170099.3-199453.5) | 248582.2 (232693.8-265379.9) | 4519.2 (4161.9-4901.8) | 3392.2 (3175.5-3621.5) | -0.91 (-0.94, -0.88) |
| Armenia | 14144.3 (12891.2-15441) | 23126 (21567.1-24755.5) | 4465.8 (4051.5-4890.4) | 3970.5 (3700.1-4256.3) | -0.37 (-0.41, -0.32) |
| Australia | 111528.7 (105940-117391.6) | 196612.9 (186015.2-207216.8) | 4346.4 (4121.9-4580.1) | 3171.3 (3003.1-3339.7) | -1.01 (-1.06, -0.96) |
| Austria | 66710.4 (62524.5-70951.8) | 125761.4 (118119.4-133660.4) | 4056.1 (3795.9-4320.5) | 4953.2 (4655.2-5264.1) | 0.7 (0.59, 0.8) |
| Azerbaijan | 19890.5 (17832.7-22118.4) | 43653 (40036.1-47376.7) | 3589 (3199.9-4009.6) | 3902.2 (3550.3-4266.5) | 0.27 (0.24, 0.3) |
| Bahamas | 568.3 (521.4-617.7) | 1344.6 (1248-1446.1) | 3275.1 (2995.6-3569.9) | 2891.4 (2674.9-3120) | -0.39 (-0.41, -0.38) |
| Bahrain | 449.4 (409-494.4) | 1916 (1776.2-2071.3) | 2632.9 (2355-2941.9) | 2220.6 (2032.1-2432.6) | -0.52 (-0.56, -0.48) |
| Bangladesh | 154997.5 (137456-172586.3) | 482675.3 (434346.6-532380.3) | 2972.2 (2620.9-3328.6) | 2903.2 (2596.1-3222.2) | -0.09 (-0.14, -0.04) |
| Barbados | 1377.1 (1235.9-1525.8) | 2063.9 (1904.9-2236) | 3393.6 (3045.7-3758.7) | 2981.5 (2748.8-3233.6) | -0.41 (-0.42, -0.4) |
| Belarus | 88560.6 (76025.7-101363.9) | 106052.6 (96823.1-116191.4) | 5267.2 (4504.2-6048.2) | 4913.2 (4479.8-5387.3) | -0.21 (-0.31, -0.1) |
| Belgium | 73942.1 (65866.5-82946.1) | 99645.1 (93925.2-105439.1) | 3546.3 (3156.7-3981.9) | 3056.6 (2887-3229.5) | -0.47 (-0.5, -0.44) |
| Belize | 280.3 (254.3-307.5) | 766.4 (705.5-829.5) | 2472.7 (2242.9-2712.6) | 2332.4 (2140.9-2530.7) | -0.18 (-0.23, -0.14) |
| Benin | 12975.4 (11928.9-14115.7) | 26249.7 (24414.7-28187.8) | 5724.7 (5243-6252.5) | 4986.8 (4618.1-5372.1) | -0.45 (-0.46, -0.43) |
| Bermuda | 267.6 (245.2-291.1) | 492.5 (455-532) | 3493.6 (3193-3812.3) | 2638.7 (2441.5-2845.9) | -0.89 (-0.92, -0.86) |
| Bhutan | 657.9 (583.4-730.8) | 1894.6 (1706.3-2081.4) | 2654.6 (2316.8-2992.5) | 2663.7 (2389.2-2937.4) | 0 (-0.01, 0.02) |
| Bolivia (Plurinational State of) | 9837 (8830.4-10874.1) | 23955.3 (21807.5-26142.6) | 2763.8 (2459.2-3081.8) | 2266.5 (2045.6-2494) | -0.64 (-0.66, -0.63) |
| Bosnia and Herzegovina | 24931.5 (22617.5-27348.5) | 44397 (40724.6-48274.9) | 5345.9 (4817.8-5895.9) | 5239.3 (4802.7-5700.7) | -0.06 (-0.07, -0.05) |
| Botswana | 4162.9 (3768.8-4536.7) | 10913.1 (10084-11781.7) | 7471.7 (6701.9-8209.5) | 7711.8 (7086.8-8373.9) | 0.1 (0.09, 0.11) |
| Brazil | 448741.2 (376414.7-530858.6) | 998353.2 (846873.6-1166938.7) | 4393.7 (3675-5218.2) | 3227.3 (2735.4-3775.3) | -0.99 (-1.02, -0.95) |
| Brunei Darussalam | 970.4 (904.6-1039.8) | 1900.4 (1783.7-2020.1) | 9366.4 (8716.9-10053.6) | 5326.1 (4981.9-5681.4) | -1.78 (-1.87, -1.69) |
| Bulgaria | 91393.2 (79615.6-105182.8) | 104397.1 (89864.2-119679.1) | 5526.7 (4776.9-6405.3) | 5214.4 (4493.1-5974.9) | -0.19 (-0.2, -0.18) |
| Burkina Faso | 18475.8 (17035.2-19939.8) | 35615.1 (33270.3-38000.4) | 3938.2 (3609.2-4274.6) | 3665.4 (3408-3927.9) | -0.23 (-0.24, -0.22) |
| Burundi | 16835.1 (15121.7-18476.2) | 23632.1 (21693.6-25529.4) | 6639.4 (5912.8-7345.3) | 4981.8 (4527.1-5423.7) | -0.92 (-0.95, -0.89) |
| Cabo Verde | 1413.7 (1305.7-1521.2) | 2461 (2296.8-2628) | 4671.9 (4311.9-5031.4) | 4837.3 (4514.8-5165.7) | 0.12 (0.11, 0.12) |
| Cambodia | 17660.6 (15902.6-19425) | 51170.5 (46833-55726.2) | 3660.6 (3263.4-4072) | 3706.2 (3360-4077) | 0.03 (0.01, 0.04) |
| Cameroon | 22839.7 (20923.6-24845.4) | 61179.1 (56806.7-65489.6) | 4988.4 (4542.7-5459.5) | 4904.3 (4523-5280.9) | -0.05 (-0.07, -0.03) |
| Canada | 210619.6 (197979.4-223214.5) | 427604.7 (411240.7-443937.5) | 4973 (4670.4-5274.9) | 4313.1 (4150.6-4475.3) | -0.44 (-0.47, -0.42) |
| Central African Republic | 5757.5 (5215.5-6308.3) | 9451.9 (8642.4-10267.9) | 5512.1 (4916.5-6115.8) | 5018.7 (4518-5527) | -0.3 (-0.32, -0.29) |
| Chad | 16807.2 (15378.5-18250) | 28755.6 (26627.3-30797) | 5171.8 (4708.6-5646.2) | 4921.8 (4525.8-5303.2) | -0.16 (-0.17, -0.15) |
| Chile | 50391.7 (46607.3-54473.2) | 104895.1 (97603.6-112587.3) | 4188.8 (3865.4-4538.1) | 3163.2 (2944-3394.5) | -0.91 (-0.93, -0.88) |
| China | 3656009.8 (3006526.9-4392985.9) | 15217282.8 (12967025.7-17730954.2) | 3990.6 (3264.3-4828.9) | 5836 (4965.7-6812.6) | 1.18 (1.12, 1.24) |
| Colombia | 73648.6 (68538.7-79327.3) | 175036.5 (163890.2-187781.8) | 3840.6 (3564.4-4144.9) | 2505.5 (2349.5-2683.4) | -1.37 (-1.43, -1.32) |
| Comoros | 1391.7 (1275.8-1508) | 3058.1 (2839.7-3283.1) | 6974.2 (6348.7-7599.6) | 5921.4 (5478.8-6377.9) | -0.52 (-0.53, -0.51) |
| Congo | 8223.8 (7542.5-8917.1) | 16167.6 (15016-17347.4) | 7580 (6882.5-8293.6) | 6575.1 (6060.3-7110.3) | -0.46 (-0.47, -0.45) |
| Cook Islands | 59.9 (55.2-64.8) | 150.7 (140.8-160.5) | 4445.6 (4073.7-4827.4) | 4523 (4221.8-4820.5) | 0.04 (0.03, 0.05) |
| Costa Rica | 6579 (6095-7109.7) | 18336.3 (17057.3-19714.7) | 3220.5 (2980.8-3482.7) | 2644.5 (2463.9-2841) | -0.64 (-0.65, -0.63) |
| Coted'Ivoire | 22717.8 (20907.2-24528.4) | 56032.7 (52043.2-59951.4) | 6195.1 (5662.9-6729.3) | 5293.3 (4893.5-5691.7) | -0.51 (-0.52, -0.5) |
| Croatia | 36143.2 (31752.5-40913.7) | 53787 (51000.5-56552) | 4859.7 (4245.2-5530.8) | 4277.8 (4060.9-4493.3) | -0.39 (-0.42, -0.35) |
| Cuba | 38459.7 (35233.8-41783.2) | 68252.3 (62936.2-73762.1) | 2959.9 (2706.8-3220.7) | 2745.9 (2537.4-2961.9) | -0.24 (-0.26, -0.22) |
| Cyprus | 3384.3 (3045.7-3750.7) | 4158.9 (3659.8-4772.1) | 3192.3 (2847.1-3567.6) | 1515.8 (1322.9-1754.3) | -2.37 (-2.49, -2.25) |
| Czechia | 105049.5 (93376.4-117831.6) | 131730.7 (123333.9-140535.1) | 5710.9 (5063.8-6424.2) | 4394.5 (4113.7-4688.7) | -0.8 (-0.88, -0.72) |
| Democratic People's Republic of Korea | 95100 (85961.5-104403) | 225734.3 (206261.5-246386.9) | 5789.3 (5193.7-6396.7) | 5796.6 (5270-6357) | 0.01 (0, 0.02) |
| Democratic Republic of the Congo | 83305.5 (75632.8-91243.8) | 166508.4 (153137.4-180869.2) | 5465.8 (4890.1-6063.4) | 4862.9 (4420-5336.9) | -0.37 (-0.4, -0.35) |
| Denmark | 57521.2 (53692.6-61536.4) | 49287.7 (45902.3-52852.6) | 5143.3 (4796.6-5505.7) | 3001.8 (2794.9-3219.4) | -1.67 (-1.75, -1.59) |
| Djibouti | 787.5 (725-851.5) | 3711.1 (3452.4-3975.7) | 6938.6 (6339.1-7546.5) | 6622.5 (6124.7-7138) | -0.15 (-0.16, -0.14) |
| Dominica | 217.6 (196.9-240.5) | 264.6 (241.4-288) | 2757.2 (2489.8-3054.5) | 2523.9 (2293.3-2757.6) | -0.28 (-0.3, -0.27) |
| Dominican Republic | 12929.4 (11829.2-14121.9) | 42489.9 (38853.6-46284.4) | 3062.1 (2787.6-3360.8) | 3532.2 (3228.2-3850.1) | 0.47 (0.45, 0.48) |
| Ecuador | 20249.6 (18666.4-21814.1) | 56023.5 (52049.2-60139.9) | 3368.6 (3098.6-3636.9) | 2796.5 (2593.8-3006.1) | -0.59 (-0.62, -0.57) |
| Egypt | 97881.6 (84505.7-111768.9) | 303180 (272568.5-333867.6) | 3237.2 (2759.2-3741.9) | 4177.9 (3699.2-4663.5) | 0.82 (0.81, 0.84) |
| El Salvador | 9618.4 (8819.9-10459.5) | 18345.5 (16940.8-19863.3) | 2761.3 (2532-3003) | 2290.4 (2121.9-2472.2) | -0.6 (-0.63, -0.56) |
| Equatorial Guinea | 1204.1 (1098.1-1315.1) | 2657.9 (2472.8-2854.3) | 6362.3 (5736.3-7002.6) | 5884 (5452.8-6340.8) | -0.24 (-0.27, -0.21) |
| Eritrea | 5389.6 (4912.6-5866.2) | 14018.2 (12991.4-15069.5) | 6129.8 (5511.5-6755.3) | 5664.4 (5191.3-6149) | -0.26 (-0.26, -0.25) |
| Estonia | 11083.5 (9592.5-12634.9) | 11222 (10123.2-12376.1) | 4145 (3586.5-4726.9) | 3092.5 (2809.3-3385.1) | -0.9 (-0.93, -0.87) |
| Eswatini | 1623.8 (1467.1-1786) | 3222.8 (2953.8-3486.8) | 5913.4 (5299.4-6557.2) | 6031.8 (5475-6587.7) | 0.06 (0.06, 0.07) |
| Ethiopia | 83541.6 (67176.8-101440.4) | 162792.8 (134071-193303.4) | 4381.7 (3491.3-5380) | 3670.2 (3006.8-4385) | -0.57 (-0.6, -0.53) |
| Fiji | 1854.5 (1700.6-2015.9) | 4016.5 (3738.4-4294.3) | 5669.7 (5167.1-6193) | 5039.6 (4663.2-5421.1) | -0.38 (-0.39, -0.37) |
| Finland | 59854.5 (56248.6-63459.4) | 81614.9 (76794.4-86762) | 6263.3 (5878.8-6647.9) | 4476.3 (4215.8-4755.8) | -0.98 (-1.06, -0.9) |
| France | 353796.6 (325651.5-384138.9) | 610664.8 (577722.1-644393.8) | 3122.5 (2875.9-3391.5) | 3136.4 (2973-3304.9) | 0.03 (-0.02, 0.08) |
| Gabon | 4786.8 (4410.4-5172.2) | 6781.7 (6319.5-7261.5) | 7510.6 (6893.6-8146.5) | 6596.6 (6122.3-7090.7) | -0.42 (-0.43, -0.4) |
| Gambia | 2138.8 (1974.7-2307.5) | 5702.6 (5322.6-6093.9) | 5955.8 (5469.9-6460.3) | 5528.4 (5137-5935) | -0.23 (-0.25, -0.22) |
| Georgia | 30044.2 (27072.2-33338.8) | 34368.8 (31203.1-37800.6) | 3828 (3433.9-4265.6) | 4296.8 (3907.2-4718.1) | 0.36 (0.33, 0.39) |
| Germany | 1044178.1 (979771.6-1112462.1) | 1377491.1 (1303893.3-1452462.7) | 6128.5 (5743.6-6536.5) | 5008.6 (4736.9-5284.5) | -0.64 (-0.65, -0.62) |
| Ghana | 46576.5 (43060.6-50084.3) | 130071 (121761.9-138633.9) | 7220.6 (6638.2-7806.2) | 7485.7 (6970.3-8020.3) | 0.11 (0.09, 0.13) |
| Greece | 92012.3 (82975.3-102063) | 117474.5 (108442.6-127121.8) | 4567 (4107-5080.8) | 3428.4 (3171.9-3703.5) | -0.91 (-0.99, -0.84) |
| Greenland | 285 (261-312.4) | 407.8 (381.3-434.1) | 9033.7 (8203.2-9986.2) | 5207.2 (4846.5-5567.7) | -1.76 (-1.82, -1.69) |
| Grenada | 376.6 (323.1-436.6) | 442.3 (387.5-504.6) | 3903.1 (3362.2-4504.2) | 3255.8 (2832.7-3744.1) | -0.6 (-0.61, -0.59) |
| Guam | 365.1 (338.6-393.8) | 1257.6 (1179.2-1339) | 5033.2 (4648.6-5451.6) | 4617.4 (4338.8-4907.6) | -0.28 (-0.29, -0.27) |
| Guatemala | 9339.3 (8595.3-10107.4) | 27669.4 (25628.9-29771) | 2598.7 (2378-2829.1) | 2137.7 (1974.5-2305.5) | -0.64 (-0.65, -0.62) |
| Guinea | 20343.5 (18585.6-22028.1) | 31684.5 (29376.8-33994.9) | 5264.6 (4782.8-5730.8) | 5198.9 (4799.7-5603.3) | -0.04 (-0.05, -0.04) |
| Guinea-Bissau | 2482.2 (2266.6-2689.2) | 3841.8 (3571.1-4111.4) | 5997.8 (5438.7-6548) | 5590.3 (5156.5-6026.8) | -0.23 (-0.24, -0.21) |
| Guyana | 1913.8 (1701.9-2147.8) | 2632.3 (2414.9-2873.9) | 4406.6 (3897-4974.4) | 3500.2 (3186-3850.4) | -0.75 (-0.78, -0.73) |
| Haiti | 11644.6 (10311.9-13056.6) | 22522.5 (20169.7-24913) | 3218.2 (2815.6-3646.1) | 2900.6 (2569.5-3244.8) | -0.34 (-0.35, -0.32) |
| Honduras | 5928.6 (5344-6503.6) | 18211 (16592-19863.7) | 2656.6 (2378.3-2934.8) | 2562.9 (2315.1-2821) | -0.12 (-0.13, -0.1) |
| Hungary | 128984.6 (118214.1-140639.6) | 110232.9 (101834.4-119392.1) | 6563.6 (5996.1-7178.1) | 4058.3 (3753.4-4390.9) | -1.53 (-1.6, -1.45) |
| Iceland | 1886.3 (1737.2-2044.4) | 2551.1 (2378.5-2727.2) | 4954.9 (4563.6-5369.7) | 3232.5 (3017.7-3452.8) | -1.32 (-1.37, -1.27) |
| India | 1091868.2 (864228.4-1336872.5) | 2852087.6 (2316264.1-3429284.5) | 2199.1 (1726.1-2721.2) | 2032.2 (1639.1-2460.1) | -0.25 (-0.28, -0.21) |
| Indonesia | 542675.8 (441090.2-653141.3) | 1490563.6 (1233753.3-1763684.9) | 5319.4 (4300.3-6449.8) | 5770.9 (4753.5-6879.6) | 0.26 (0.25, 0.27) |
| Iran (Islamic Republic of) | 105111.1 (84937.1-127461.3) | 300356.4 (254115.6-352334.5) | 3482 (2805.5-4247.4) | 3374.4 (2849.1-3967.4) | -0.09 (-0.11, -0.08) |
| Iraq | 38541.5 (34217.2-42986.5) | 118118.9 (107893.4-128871.7) | 4294.3 (3803.7-4798.6) | 4606.4 (4173.7-5066) | 0.23 (0.22, 0.24) |
| Ireland | 22833.5 (20564.5-25152.3) | 20926.8 (19248.5-22765.7) | 4132.4 (3713.8-4567) | 1993.5 (1833.3-2168.7) | -2.33 (-2.37, -2.29) |
| Israel | 33633.3 (31531.3-35880.9) | 56731.3 (53051.5-60644.5) | 5259.1 (4920.5-5618.8) | 3420.8 (3199.8-3655.2) | -1.27 (-1.34, -1.19) |
| Italy | 378005.4 (318686.5-444750.3) | 457833.9 (384136.1-535372.4) | 3158.7 (2656-3725.5) | 2218.9 (1871-2585.2) | -1.13 (-1.16, -1.1) |
| Jamaica | 6719.3 (6068.8-7424.2) | 9741.5 (8877.8-10746.6) | 2848.9 (2574.5-3146.4) | 2524.9 (2308-2776.5) | -0.39 (-0.41, -0.37) |
| Japan | 1074154.8 (919265-1233242.4) | 2070323.6 (1799554.9-2367655.9) | 5033.7 (4300.6-5787.6) | 3945.9 (3430-4509.7) | -0.78 (-0.8, -0.76) |
| Jordan | 7273.4 (6566.1-8015.3) | 45337.9 (42266.8-48559.9) | 5389.7 (4820.5-5993.6) | 6082.7 (5629.6-6557.3) | 0.42 (0.34, 0.51) |
| Kazakhstan | 103442.8 (94236.7-113135.1) | 124650.8 (115067.8-135276.5) | 6879.1 (6245.1-7557.3) | 5884.1 (5391-6428.2) | -0.5 (-0.54, -0.46) |
| Kenya | 46741.8 (38133.2-56250.4) | 116847.3 (97253.4-137907.2) | 5483.5 (4453.1-6634.8) | 5245.1 (4342.3-6236.1) | -0.15 (-0.15, -0.14) |
| Kiribati | 215.7 (197.5-234.7) | 375.1 (347.2-404.8) | 5764.3 (5232.1-6322.8) | 5227.9 (4789.8-5693.6) | -0.32 (-0.33, -0.31) |
| Kuwait | 2275 (2103.6-2446.7) | 10304.4 (9632.4-11045.1) | 4289.8 (3949-4631.6) | 3979.7 (3716.8-4271.9) | -0.23 (-0.29, -0.18) |
| Kyrgyzstan | 18553.6 (16790.2-20468.9) | 20156.6 (18537.9-21917.4) | 5135.8 (4630.7-5687.2) | 3718.6 (3392.8-4071.5) | -1.03 (-1.06, -1) |
| Lao People's Democratic Republic | 10370 (9358.1-11434.7) | 21392.1 (19591.9-23258.4) | 4481.7 (4000.8-4999.2) | 4358.9 (3961.5-4777.7) | -0.09 (-0.1, -0.09) |
| Latvia | 22875.5 (19431.5-26347.9) | 27542.1 (25068.7-30117.5) | 4917.1 (4172-5668.6) | 5054.7 (4629.9-5498.4) | 0.12 (-0.08, 0.31) |
| Lebanon | 8934.1 (8037.3-9860.6) | 28455.6 (25709.9-31370.4) | 3504.9 (3127.2-3896.2) | 3773.1 (3424.2-4140.4) | 0.25 (0.21, 0.29) |
| Lesotho | 4243.7 (3808.6-4696.9) | 6298.9 (5718-6884.1) | 4521.5 (4028.7-5040.3) | 5639.9 (5059.7-6248.1) | 0.71 (0.68, 0.75) |
| Liberia | 6982.6 (6417.4-7560.3) | 8964.1 (8321.3-9612.1) | 5260.7 (4804.2-5728.5) | 4480 (4138.5-4824.7) | -0.51 (-0.53, -0.5) |
| Libya | 6258.5 (5638-6900.2) | 17696.2 (16176.6-19302.8) | 2971.2 (2663.7-3291) | 3290.1 (2990.8-3605.1) | 0.35 (0.31, 0.4) |
| Lithuania | 23540.6 (19593.2-27410.8) | 31364.4 (25448-38022.1) | 4071.5 (3385.1-4739.4) | 4121.2 (3384.6-4935.4) | 0.2 (-0.55, 0.97) |
| Luxembourg | 2720.2 (2285.5-3194.7) | 2937.2 (2744.4-3132.7) | 3779.3 (3168.7-4448.2) | 2123.7 (1988.2-2261.5) | -1.85 (-1.9, -1.79) |
| Madagascar | 38326.5 (35211.8-41497.8) | 71800.3 (66694.8-77028.1) | 7203.7 (6573.6-7842.1) | 7029.2 (6474.4-7609.3) | -0.08 (-0.08, -0.07) |
| Malawi | 23101.2 (21069.8-25059.2) | 40567.9 (37502.6-43552.3) | 5864 (5304.6-6407.8) | 5476.8 (5024.2-5922) | -0.22 (-0.23, -0.21) |
| Malaysia | 52805.7 (48060.6-57698.6) | 167408.9 (155664.1-179340.6) | 5273.7 (4787.3-5777.8) | 5093.6 (4718.5-5479.4) | -0.11 (-0.12, -0.1) |
| Maldives | 497.2 (455.5-538.2) | 1207.5 (1114.9-1306.2) | 5649.6 (5120.8-6183.5) | 3610.2 (3326.8-3912) | -1.43 (-1.49, -1.36) |
| Mali | 18074.2 (16577.7-19620.4) | 34488.5 (32086.6-36908.2) | 4410.7 (4006.7-4834.7) | 3839.7 (3542.5-4136.2) | -0.45 (-0.47, -0.43) |
| Malta | 1883.9 (1676.5-2116) | 2618 (2402.3-2844.4) | 3458.5 (3068.2-3895.1) | 1896.5 (1741.1-2059) | -1.93 (-1.97, -1.88) |
| Marshall Islands | 84.2 (76-92.3) | 151.3 (139.3-163.7) | 4948.3 (4432-5468.8) | 4651 (4239.5-5091) | -0.2 (-0.21, -0.19) |
| Mauritania | 7476.7 (6901.6-8069.7) | 12369.5 (11532.9-13252.6) | 6694.4 (6150.8-7253.7) | 5187.2 (4820.5-5574.5) | -0.81 (-0.83, -0.8) |
| Mauritius | 5103.9 (4692.1-5561.5) | 8638.8 (8016.5-9290.2) | 6060.3 (5531.2-6650) | 3760.3 (3479.9-4053.8) | -1.53 (-1.6, -1.45) |
| Mexico | 171743.2 (145869.4-200244.5) | 429215.9 (366301.6-497575.3) | 3692.3 (3124.5-4314) | 2884.1 (2458.4-3347.5) | -0.8 (-0.82, -0.78) |
| Micronesia (Federated States of) | 286.9 (259.7-313.5) | 357.4 (329-385.7) | 5249.8 (4723.3-5774.1) | 4847 (4424.5-5276.7) | -0.26 (-0.28, -0.24) |
| Monaco | 517.2 (470.5-565) | 472 (433.1-511.1) | 5104.4 (4643.7-5574.6) | 3433.5 (3158.3-3711.9) | -1.27 (-1.3, -1.24) |
| Mongolia | 3718.9 (3357.2-4091.2) | 8512.6 (7852.3-9172.8) | 3111 (2783.2-3451.6) | 3689 (3372.8-4010.2) | 0.56 (0.49, 0.64) |
| Montenegro | 2358.8 (2135.5-2588.3) | 4075.1 (3730.9-4430) | 3170.1 (2861-3486.7) | 3134.8 (2853-3427.2) | -0.01 (-0.05, 0.03) |
| Morocco | 63219.3 (56323.8-70645.5) | 171392.4 (155642.6-188018.8) | 3725.4 (3303.6-4184.1) | 4126.2 (3726.2-4554.1) | 0.33 (0.3, 0.35) |
| Mozambique | 35743 (32395.7-39243.2) | 72146.7 (66231.1-78124.6) | 5797.2 (5198-6420.3) | 6491.4 (5907.6-7090.6) | 0.35 (0.31, 0.38) |
| Myanmar | 113290.7 (101631.3-124865.5) | 225297.1 (206899.9-243968.3) | 4361.2 (3871.5-4857.5) | 4060.7 (3706.6-4423.7) | -0.22 (-0.23, -0.22) |
| Namibia | 4748.6 (4335-5158.6) | 8647.6 (7955.9-9341.7) | 7165.1 (6479.8-7859.2) | 6311.1 (5766.2-6859.3) | -0.41 (-0.42, -0.39) |
| Nauru | 28.5 (26.2-30.9) | 34.7 (32.5-36.9) | 6761.1 (6170.5-7370) | 6132 (5741.1-6533.1) | -0.32 (-0.33, -0.31) |
| Nepal | 23472.2 (20731.9-26294.5) | 59608.3 (53070.8-66006.9) | 2343.1 (2036.7-2665.9) | 2152.2 (1895.8-2409) | -0.27 (-0.29, -0.25) |
| Netherlands | 151730 (141045.3-162673.9) | 156383.5 (145044.9-168164.1) | 5704.2 (5297.8-6120.6) | 3237 (3001.9-3480.4) | -1.82 (-1.93, -1.71) |
| New Zealand | 20324.6 (16848.6-24194) | 33585.6 (28178.2-39780.5) | 3942.6 (3261.4-4706.4) | 2935.2 (2465-3475) | -0.93 (-0.97, -0.9) |
| Nicaragua | 5932.8 (5431.4-6471.2) | 15698.2 (14433.2-17030) | 3570.6 (3258.8-3907.5) | 2830.7 (2597-3077.2) | -0.74 (-0.76, -0.71) |
| Niger | 14051.2 (12876.7-15231.6) | 36224.8 (33569-38861.9) | 5094.6 (4623.2-5574.8) | 4307.7 (3962-4663.4) | -0.54 (-0.56, -0.52) |
| Nigeria | 247020.4 (200998.8-299530.3) | 435474.1 (361904.9-514763.4) | 5123 (4147.6-6241.9) | 4858.7 (4019.2-5776) | -0.17 (-0.18, -0.16) |
| Niue | 16.9 (15.4-18.4) | 13.8 (12.9-14.8) | 5725.1 (5220.2-6229.8) | 5146.6 (4772.3-5532.9) | -0.34 (-0.35, -0.33) |
| North Macedonia | 14219.3 (12054-16696.3) | 23233 (19694.7-27172.7) | 6494.5 (5482.2-7662.4) | 5459.8 (4590.4-6447.9) | -0.56 (-0.58, -0.54) |
| Northern Mariana Islands | 56.8 (52.5-61.2) | 213.6 (199.4-227.9) | 4762.9 (4387.2-5152.9) | 4244.4 (3954.2-4541.5) | -0.37 (-0.4, -0.34) |
| Norway | 59594.4 (50474.2-69630.9) | 61937.2 (52799.1-71557.1) | 6226.6 (5269.9-7280.1) | 4433.3 (3780.2-5122.4) | -1.09 (-1.12, -1.07) |
| Oman | 2727.7 (2423.3-3032) | 7284.6 (6721.4-7907.9) | 3838.1 (3385-4296.4) | 3900.9 (3565.4-4276) | 0.05 (0.02, 0.08) |
| Pakistan | 225972 (179973.1-276663.5) | 438099.2 (356033.1-525960) | 3480 (2758.9-4287.6) | 3403.9 (2745.5-4122.6) | -0.07 (-0.08, -0.07) |
| Palau | 63.7 (58.3-69.3) | 136.7 (126.7-146.2) | 5833.3 (5299.9-6386) | 5812.3 (5356.9-6259.1) | -0.01 (-0.02, -0.01) |
| Palestine | 3255.1 (2902.4-3644.8) | 8201.1 (7444.8-8995.2) | 3130.7 (2775.3-3525) | 2984.8 (2681.5-3303.3) | -0.17 (-0.23, -0.12) |
| Panama | 5717.1 (5253-6228.7) | 15453.7 (14308.5-16651) | 3309.8 (3035.9-3612.2) | 2780.9 (2578.7-2992.7) | -0.56 (-0.58, -0.54) |
| Papua New Guinea | 5236 (4789.1-5720) | 13756 (12701.8-14813.3) | 3278.6 (2963.7-3620.9) | 3140.9 (2879.1-3405.2) | -0.14 (-0.16, -0.13) |
| Paraguay | 9901.3 (9029-10856.5) | 22310 (20535.3-24130.7) | 3839.5 (3494.1-4218.1) | 3255.2 (2991.3-3525.2) | -0.53 (-0.55, -0.51) |
| Peru | 36410.8 (33262.4-39683.4) | 94599.8 (87788.9-101466.9) | 2703.8 (2466.1-2950.2) | 2323.9 (2158.8-2490.7) | -0.49 (-0.51, -0.48) |
| Philippines | 108490.4 (89404-129674.7) | 358948.8 (300830.3-422673) | 3528.1 (2891.8-4245.6) | 3961.1 (3306-4690.3) | 0.38 (0.36, 0.39) |
| Poland | 182168.5 (152170.3-216504.1) | 320760.9 (267158.3-380912.1) | 3232.1 (2693.6-3850.3) | 3239 (2700.9-3844.3) | -0.01 (-0.05, 0.04) |
| Portugal | 106343.8 (91267.9-123325.7) | 72936.8 (66596.1-79538) | 5715.6 (4883.1-6652.1) | 2140.7 (1962.2-2326.2) | -3.11 (-3.21, -3.01) |
| Puerto Rico | 11803.4 (10830.2-12867) | 23521.7 (21818.4-25209.6) | 2541.7 (2325-2776.4) | 2287.4 (2127.7-2446.4) | -0.33 (-0.36, -0.31) |
| Qatar | 460.1 (426.4-493.7) | 2168.5 (2012-2330.6) | 5924.6 (5447.8-6418.3) | 3319.3 (3040.5-3613.3) | -1.68 (-1.94, -1.42) |
| Republic of Korea | 302986.5 (282983.5-325594) | 566118.5 (532847.3-602205.1) | 9595.5 (8919.6-10351.8) | 4602.7 (4329.8-4897.9) | -2.34 (-2.44, -2.24) |
| Republic of Moldova | 19954.1 (17850.2-21972.3) | 29166.2 (26535.6-31942.3) | 3531.3 (3138.8-3915) | 3660.3 (3322.8-4016) | 0.12 (0.09, 0.15) |
| Romania | 200437.4 (176693.4-226603.5) | 257958.3 (237784.2-279123) | 5671.6 (4972.5-6441.3) | 4986.4 (4603.2-5388.7) | -0.41 (-0.44, -0.38) |
| Russian Federation | 1023114.2 (859249.6-1197730.5) | 1384032.6 (1175000.1-1609067) | 4287.1 (3596-5021.8) | 4213.8 (3573.1-4904.7) | -0.09 (-0.16, -0.01) |
| Rwanda | 19201.2 (17245-21170.2) | 31400.3 (28891.3-34009.4) | 6621.2 (5875.6-7374.2) | 5003.2 (4556.4-5467.5) | -0.89 (-0.92, -0.87) |
| Saint Kitts and Nevis | 289.6 (251.1-333.9) | 294.2 (270.5-320.6) | 5307.9 (4586.8-6142.9) | 3602 (3281.7-3953.6) | -1.22 (-1.29, -1.15) |
| Saint Lucia | 463.8 (408.9-524.1) | 912.6 (842.8-990.3) | 4118.4 (3612.1-4679.5) | 3066.8 (2829.8-3330.7) | -0.94 (-0.96, -0.92) |
| Saint Vincent and the Grenadines | 331.6 (295.8-374) | 524.9 (481.4-568.6) | 3538 (3146.1-4003.4) | 2880.2 (2633.8-3128.9) | -0.66 (-0.7, -0.63) |
| Samoa | 479.9 (436.6-522.7) | 792.5 (736.6-849.9) | 5221.2 (4720.7-5719.3) | 5011.3 (4643.1-5391.7) | -0.13 (-0.14, -0.12) |
| San Marino | 207.9 (190.2-226) | 314.3 (287.9-342.4) | 4364.2 (3990.1-4746.7) | 3044.8 (2803.9-3300.7) | -1.15 (-1.18, -1.12) |
| Sao Tome and Principe | 516.5 (476.4-557.5) | 766.4 (714-819.9) | 6705.8 (6161.5-7265.7) | 6689.4 (6207.1-7184.1) | -0.01 (-0.02, 0) |
| Saudi Arabia | 19948.6 (17739.4-22287.8) | 54904.7 (50308.6-59707.5) | 3280.6 (2897.1-3690.4) | 3444.1 (3110.4-3796.7) | 0.16 (0.14, 0.17) |
| Senegal | 21484.3 (19792.9-23151.5) | 44106.7 (41094.7-47042.5) | 6095.8 (5591-6594.4) | 5284.7 (4903.5-5659.9) | -0.45 (-0.46, -0.44) |
| Serbia | 78356.9 (66770-91470.7) | 102464.4 (87867-117995.4) | 5703 (4827.9-6698.6) | 4485.3 (3841.4-5173.7) | -0.78 (-0.8, -0.75) |
| Seychelles | 350.6 (320.7-382.6) | 561.1 (520.6-602.6) | 4842.9 (4425.8-5291.1) | 4204.4 (3887.8-4530.7) | -0.45 (-0.46, -0.44) |
| Sierra Leone | 13957.5 (12879.7-15141.1) | 21187.5 (19679.9-22751.3) | 5898 (5417.1-6426.1) | 5328.4 (4923.7-5750.2) | -0.33 (-0.34, -0.31) |
| Singapore | 19274.5 (18032.4-20695.4) | 38058.9 (35603.2-40628.5) | 7947.3 (7421.8-8548.5) | 3520 (3295.6-3755.2) | -2.57 (-2.64, -2.5) |
| Slovakia | 64321.9 (60388.4-68482.1) | 75587.9 (70278.2-80776.9) | 8371.9 (7838.7-8934.9) | 5931.3 (5508.3-6345.1) | -0.97 (-1.16, -0.78) |
| Slovenia | 11579.1 (9892.1-13364.6) | 16419.1 (15267.1-17613.7) | 3721.4 (3171-4302.2) | 2671.4 (2494.4-2854.4) | -1.04 (-1.17, -0.91) |
| Solomon Islands | 726.3 (653.7-798.2) | 1823.8 (1661.5-1978.7) | 5680 (5055.3-6313.9) | 5594.4 (5060.9-6122.2) | -0.05 (-0.06, -0.05) |
| Somalia | 10279.5 (9223.4-11354.3) | 26063.3 (23773.3-28279.6) | 5126.4 (4539.5-5737.1) | 4570.4 (4086.2-5040.5) | -0.36 (-0.38, -0.33) |
| South Africa | 163088.2 (135002.9-194454.1) | 316757.2 (267990.3-371369.8) | 7156.1 (5916.8-8547.3) | 6292.7 (5307.5-7387.6) | -0.42 (-0.45, -0.38) |
| South Sudan | 15709.2 (14347.4-17119.8) | 17147 (15809.2-18449.9) | 5668.8 (5153.9-6204.8) | 4939.3 (4527.5-5344.6) | -0.41 (-0.44, -0.39) |
| Spain | 297525.1 (258442.1-335839.6) | 462173.7 (439332.9-485179.7) | 4055.2 (3517.5-4583.2) | 3465.5 (3296.8-3635.1) | -0.48 (-0.57, -0.39) |
| Sri Lanka | 52615.9 (48015.6-57335.7) | 135458.4 (125060.7-145518.7) | 4461.6 (4044.4-4896) | 3959.9 (3635.1-4279.2) | -0.39 (-0.41, -0.37) |
| Sudan | 38067 (33426.5-43065.8) | 81018 (72870.4-89206.4) | 3440.4 (3003.8-3915.9) | 3865.1 (3454.2-4284.6) | 0.38 (0.36, 0.4) |
| Suriname | 990.8 (898.5-1087.6) | 2390.6 (2184.6-2593) | 3431.6 (3105.3-3775.3) | 3146 (2867.9-3420.5) | -0.28 (-0.28, -0.27) |
| Sweden | 105380.7 (88054.1-126897.2) | 113000.4 (94725.6-132465.2) | 4934.1 (4113.4-5944.6) | 3613.5 (3035.8-4226.7) | -1.01 (-1.05, -0.97) |
| Switzerland | 45011.9 (41244.2-49641.1) | 63366.2 (59168.4-67952.9) | 3160.4 (2894-3485) | 2545.6 (2380.3-2727.8) | -0.68 (-0.71, -0.65) |
| Syrian Arab Republic | 20052.6 (18031.8-22134.4) | 46203.2 (42035.7-50633.4) | 3407.8 (3039.3-3789.2) | 2966.3 (2671.6-3282.7) | -0.44 (-0.5, -0.39) |
| Taiwan (Province of China) | 114464 (106868.2-122406.5) | 274491.9 (258150.8-291173.7) | 6927.2 (6460.7-7418.5) | 4888.7 (4600.3-5183.2) | -1.11 (-1.17, -1.05) |
| Tajikistan | 12077.3 (10837.5-13363.4) | 24006.4 (21627.1-26408.1) | 3836.4 (3430.8-4258.7) | 3967.4 (3527.6-4415.7) | 0.1 (0.05, 0.14) |
| Thailand | 163797.3 (150139.3-177542.7) | 510526.7 (476344.5-545768.2) | 4413.7 (4021.8-4809.6) | 3675.2 (3429.7-3929.4) | -0.58 (-0.61, -0.54) |
| Timor-Leste | 870.9 (781.9-955.9) | 4005.8 (3658.9-4352.3) | 3572.8 (3176.2-3957) | 3790 (3432.9-4153.1) | 0.19 (0.17, 0.21) |
| Togo | 7367.2 (6765.9-7973.6) | 19387.1 (18047.7-20759.5) | 5929.9 (5415.3-6454) | 5231.3 (4834.3-5638.7) | -0.4 (-0.42, -0.39) |
| Tokelau | 7.6 (6.9-8.3) | 8.1 (7.5-8.7) | 4360.9 (3937.9-4803.8) | 4216.2 (3909.9-4516.1) | -0.1 (-0.12, -0.08) |
| Tonga | 240.8 (220.6-261.3) | 366.1 (339.3-393.5) | 4040.6 (3688-4400.3) | 3864.3 (3578-4157) | -0.15 (-0.16, -0.13) |
| Trinidad and Tobago | 4499.5 (4114-4911.1) | 8076.3 (7454.6-8727.9) | 4312.3 (3925.8-4725) | 3220 (2964.1-3488.8) | -0.93 (-0.96, -0.9) |
| Tunisia | 18195.6 (16359.7-20117.2) | 54611.7 (49771-59840.3) | 3010.1 (2683.1-3357.4) | 3292 (2984.8-3626.7) | 0.29 (0.27, 0.32) |
| Turkey | 181786 (165253.5-199343.4) | 408077.4 (379143.3-439510) | 4718.5 (4262.7-5207.5) | 3586.5 (3321.2-3876.9) | -0.88 (-0.93, -0.83) |
| Turkmenistan | 10016.8 (9105.6-11040.3) | 25199.1 (22966.5-27526.1) | 4548.7 (4109.3-5050.9) | 5565.1 (5022.9-6132.3) | 0.67 (0.57, 0.78) |
| Tuvalu | 33.7 (30.7-36.7) | 54.8 (50.9-59.1) | 4609.4 (4157.1-5087.5) | 4533 (4177.6-4912.6) | -0.06 (-0.07, -0.05) |
| Uganda | 43380.5 (39554.7-47323.7) | 84587.9 (78262-91045.8) | 6541.3 (5920.6-7170.7) | 6050.7 (5577.3-6533.8) | -0.24 (-0.27, -0.22) |
| Ukraine | 525262.2 (433965.8-624809.6) | 471830.8 (392803.7-561727.9) | 5485.6 (4527.5-6533.8) | 4452.3 (3706.3-5303.5) | -0.63 (-0.71, -0.55) |
| United Arab Emirates | 2104.3 (1889.6-2323.2) | 15919 (14719.4-17140.6) | 6324.6 (5625.8-7039.2) | 5634.2 (5093.2-6204.3) | -0.37 (-0.39, -0.35) |
| United Kingdom | 544804.1 (477303-618058.3) | 567640.5 (503992.8-635481.2) | 4381 (3832-4976.2) | 3172.5 (2820.2-3547.2) | -1.03 (-1.08, -0.99) |
| United Republic of Tanzania | 60414.2 (55519.9-65721.8) | 160862.6 (148720.2-172874.2) | 5233.9 (4775.3-5731.3) | 6192.8 (5702-6682.6) | 0.51 (0.44, 0.58) |
| United States of America | 2164020.7 (1838385.2-2508908.4) | 3741547.5 (3270793.7-4233151.7) | 5013.5 (4256.3-5815.8) | 4694.2 (4105.7-5310.5) | -0.25 (-0.33, -0.16) |
| United States Virgin Islands | 239.9 (218.2-262.3) | 656 (602.2-712.6) | 2540.3 (2292.2-2799.1) | 2530.9 (2317.2-2758.8) | -0.01 (-0.02, 0.01) |
| Uruguay | 31204.3 (28819.1-33763.9) | 27334.9 (25385-29451) | 5993.2 (5528.9-6491.3) | 3673.8 (3420.9-3948.5) | -1.57 (-1.6, -1.54) |
| Uzbekistan | 66266.7 (58949.5-73813.8) | 151367.6 (139708.3-164109.2) | 4915.2 (4367.4-5483.6) | 5190 (4750.9-5670.1) | 0.18 (0.16, 0.19) |
| Vanuatu | 353.4 (322-385) | 986.9 (911.5-1059.3) | 5811.8 (5252-6388.5) | 5678.7 (5204.5-6144) | -0.08 (-0.09, -0.07) |
| Venezuela (Bolivarian Republic of) | 36340 (33436.3-39388.2) | 98875.5 (91180.6-106724.2) | 3430.9 (3150.2-3725.3) | 2737.5 (2522.5-2957.3) | -0.72 (-0.73, -0.7) |
| Viet Nam | 195937.7 (176113.8-215385.9) | 527178.1 (490262.8-565871.4) | 4035 (3608-4460.4) | 4554.9 (4212.8-4915.3) | 0.39 (0.36, 0.42) |
| Yemen | 17427.2 (15305.6-19528.2) | 49939.6 (44651.7-55304.9) | 3111.3 (2703.1-3522.1) | 3177.2 (2818-3545.7) | 0.09 (0.06, 0.11) |
| Zambia | 16551.4 (15153.6-18031.7) | 41123.3 (38047.3-44243.6) | 5936.3 (5393.8-6509.2) | 6420.9 (5899.1-6955) | 0.26 (0.24, 0.27) |
| Zimbabwe | 21518.5 (19729.1-23320.8) | 38625.3 (35661.6-41537.3) | 5117.9 (4650.3-5585.9) | 5647.5 (5164.6-6137) | 0.31 (0.3, 0.32) |

ASPR = age-standardized prevalence rate; AAPC=average annual percentage change; CI=confidence interval; SDI=sociodemographic index; UI=uncertainty interval.
