# Supplementary material for: Global burden of ischemic stroke in adults aged 60 years and older from 1990 to 2021: Population-based study
Source: PLoS One. 2025 May 5;20(5):e0322606. doi: 10.1371/journal.pone.0322606 (PMC12052125; doi:10.1371/journal.pone.0322606)
Supplement: S2 Table — (DOCX) [file pone.0322606.s013.docx]

Table 2. The incidence cases and ASIR of ischemic stroke in 1990 and 2021 and its trends.

|  | Incidence | | | | |
| --- | --- | --- | --- | --- | --- |
|  | 1990 counts  （95%UI） | 2021 counts  （95%UI） | 1990 ASR  （95%UI） | 2021ASR  （95%UI） | AAPC  （95%CI） |
| Global | | | | | |
| Age ≥60 group | 2871965.6 (2079466.5-3808063.4) | 5706997 (4269491.7-7444063.4) | 656.9 (480.5-863.9) | 540.2 (405.6-702.5) | -0.66 (-0.73, -0.59) |
| All age groups | 4151978.08 (3536772.36-4868149.6) | 7804449.4 (6719760.38-8943692.09) | 109.79 (93.56-127.62) | 92.39 (79.84-105.82) | -0.57 (-0.65, -0.48) |
| Sex | | | | | |
| Male | 1332205.7 (946621-1801231.6) | 2875384.1 (2107399.1-3805862.3) | 700.9 (504.3-936.3) | 606.1 (447-797.3) | -0.49 (-0.51, -0.46) |
| Female | 1539759.9 (1111659.4-2063736.5) | 2831612.9 (2091624.8-3731970.5) | 615.5 (446.9-820.7) | 480.4 (354.9-633.2) | -0.81 (-0.87, -0.76) |
| SDI quintiles | | | | | |
| High SDI | 496040.5 (369956-648928.4) | 554359.7 (429250.9-707825.5) | 553.8 (412.1-727.3) | 316 (242.6-407.3) | -1.72 (-1.78, -1.65) |
| High-middle SDI | 551115.6 (394713.2-744626.3) | 905735.4 (668781.8-1196468.1) | 817 (589.1-1096.5) | 621.1 (457.9-821.7) | -0.65 (-0.72, -0.58) |
| Middle SDI | 292540.7 (202448.2-404341.9) | 909285.6 (652681.8-1228655.4) | 537 (376.8-732.8) | 546.4 (394.1-735.2) | 0.23 (0.2, 0.26) |
| Low-middle SDI | 140586.6 (99636.6-191860) | 341926.8 (251156.7-451008.6) | 485.6 (348.5-655.2) | 424.2 (313.9-555.9) | -0.34 (-0.36, -0.32) |
| Low SDI | 57254.9 (40658.7-78058.1) | 117407.2 (86589.2-154599.7) | 540.1 (388.1-727.1) | 468.9 (348.4-612.5) | -0.47 (-0.49, -0.45) |
| Age group | | | | | |
| 60-64years | 464396.5 (335192.6-634072.7) | 744377.1 (560419.9-992843.8) | 289.1 (208.7-394.8) | 232.6 (175.1-310.2) | -0.7 (-0.76 to -0.64) |
| 65-69years | 534269.1 (359277.9-761472.2) | 1004680.4 (680518.9-1406095.9) | 432.2 (290.7-616) | 364.2 (246.7-509.7) | -0.56 (-0.78 to -0.34) |
| 70-74years | 526778.3 (386846.4-700737.3) | 1112622.1 (828150.5-1449691.2) | 622.2 (456.9-827.7) | 540.5 (402.3-704.3) | -0.46 (-0.49 to -0.44) |
| 75-79years | 544189.8 (374976.8-713154.6) | 1015086.6 (747221.3-1339906.6) | 884.1 (609.2-1158.6) | 769.7 (566.6-1016) | -0.46 (-0.59 to -0.33) |
| 80-84years | 433258.1 (328498.7-543745.8) | 881644.8 (675712-1105395.7) | 1224.7 (928.6-1537.1) | 1006.6 (771.5-1262.1) | -0.65 (-0.68 to -0.61) |
| 85-90years | 253968.8 (205880.1-307782.6) | 577064.3 (479293.2-687879.9) | 1680.7 (1362.4-2036.8) | 1262.1 (1048.3-1504.5) | -0.93 (-1.05 to -0.8) |
| 90-94years | 89941.2 (70631.5-113886.7) | 275306.1 (225121.2-340807.1) | 2098.9 (1648.3-2657.7) | 1538.9 (1258.4-1905.1) | -1.02 (-1.07 to -0.97) |
| 95+years | 25163.8 (18162.5-33211.5) | 96215.6 (73054.8-121443.2) | 2471.7 (1784-3262.1) | 1765.3 (1340.4-2228.2) | -1.1 (-1.15 to -1.04) |
| GBD Region | | | | | |
| Andean Latin America | 7764.7 (5802.5-10133.3) | 17445.3 (13295-22311.4) | 351.6 (264-457.5) | 757.8 (597.9-931.5) | -1.12 (-1.22, -1.03) |
| Australasia | 16926 (14068.7-19870) | 22963.4 (18115.9-28078.8) | 566.6 (472-664.5) | 334.2 (252-430.8) | -1.99 (-2.04, -1.94) |
| Caribbean | 12808.1 (9811.3-16291.6) | 25020.8 (19517.7-31317.9) | 428.1 (330.6-541.5) | 305.1 (221.9-411.2) | -0.5 (-0.51, -0.49) |
| Central Asia | 40317.5 (31047.8-50959.1) | 64377.3 (50507.7-79544.8) | 769.8 (593.8-971.7) | 671.7 (534.3-821.2) | -0.06 (-0.14, 0.02) |
| Central Europe | 173271.7 (134032.3-217052.9) | 206700.9 (164508.3-252585.4) | 977.9 (762.6-1217) | 508.6 (378.9-660.3) | -1.22 (-1.34, -1.09) |
| Central Latin America | 39527.2 (29135.9-52112.9) | 87197.1 (65768.9-112239.3) | 457.8 (340.5-599.5) | 766.4 (553.9-1024.9) | -1.42 (-1.49, -1.35) |
| Central Sub-Saharan Africa | 14249.6 (10271.1-19263.7) | 29906.4 (22350.3-39031.1) | 703.3 (514.6-933.7) | 394.9 (280.9-530.7) | -0.35 (-0.38, -0.32) |
| East Asia | 554892.7 (377896.4-772056.4) | 2269396.6 (1623584.2-3059350.9) | 631.6 (435.5-867.7) | 249 (190.3-317.7) | 0.95 (0.88, 1.03) |
| Eastern Europe | 385824.2 (269338.5-529287.2) | 392636.5 (287892.6-521391.9) | 1179.4 (829.9-1609) | 614.1 (465.8-786.3) | -1.11 (-1.27, -0.96) |
| Eastern Sub-Saharan Africa | 44602.5 (31851.3-60368.8) | 95126.1 (70552.7-124230.3) | 624 (451.6-833.1) | 633.5 (477.8-819.2) | -0.12 (-0.2, -0.03) |
| High-income Asia Pacific | 166916.7 (115899-231662.6) | 230625 (175667.1-294410.8) | 701.3 (489.5-969.6) | 303.5 (238.4-372.4) | -2.4 (-2.77, -2.03) |
| High-income North America | 249568.7 (172518.8-345171.2) | 276674.6 (201541.5-372601.7) | 526 (364-728.1) | 427 (326.6-544) | -1.73 (-1.77, -1.68) |
| North Africa and Middle East | 107368.1 (80663.5-139894.9) | 264264.6 (203693.9-334278) | 654.7 (498.5-841.9) | 602.2 (449.9-780.5) | -0.43 (-0.47, -0.39) |
| Oceania | 1203.5 (871.9-1622.8) | 2840.9 (2148.1-3658) | 475.3 (350.3-630.8) | 294.9 (223.4-378.4) | -0.36 (-0.38, -0.34) |
| South Asia | 221433.3 (151957.6-308080.3) | 526716.8 (376735.8-708103.5) | 417.9 (292.1-573.2) | 343.6 (249.1-457.6) | -0.64 (-0.68, -0.61) |
| Southeast Asia | 155265.7 (111966.2-207757.3) | 430328.2 (323398.8-555833) | 617.9 (450.5-818.1) | 367.3 (286.5-459.7) | -0.03 (-0.11, 0.05) |
| Southern Latin America | 30865.1 (23704.9-39307.2) | 38323.9 (29849.8-47394.9) | 552.2 (426.6-699.8) | 854 (615.3-1144.1) | -1.62 (-1.69, -1.56) |
| Southern Sub-Saharan Africa | 20391.9 (14160.8-28010.5) | 45295.6 (32498.6-61026.4) | 713.2 (497.6-974.7) | 333.9 (259.8-413.5) | 0.24 (-0.05, 0.53) |
| Tropical Latin America | 63993.5 (43105.8-90383.7) | 122976.5 (87229-165687.5) | 670.5 (457.3-937.5) | 574.6 (446.9-721) | -1.69 (-1.73, -1.66) |
| Western Europe | 515358.2 (405081-635990.1) | 464172.5 (382395-553198.4) | 662.1 (520.4-817.9) | 339.9 (278.2-407.7) | -2.13 (-2.18, -2.09) |
| Western Sub-Saharan Africa | 49416.7 (34851.5-67368.1) | 94007.8 (69363.2-123118.2) | 554.3 (395.3-747.3) | 837.5 (613-1112.8) | -0.29 (-0.33, -0.25) |
| 204 countries and regions | | | | | |
| Afghanistan | 4860.5 (3585.5-6384.9) | 4795.9 (3686-6119.7) | 701.4 (527.7-905.5) | 633.3 (493.2-798) | -0.33 (-0.36, -0.31) |
| Albania | 950.4 (703.2-1243.7) | 2199 (1656.7-2841.7) | 431 (321.6-561.1) | 399.4 (303.4-513) | -0.24 (-0.35, -0.12) |
| Algeria | 9377.7 (7038.6-12203.4) | 25989.1 (19233.1-33445.1) | 785.1 (601.3-1004.1) | 682.1 (509.9-872.5) | -0.48 (-0.74, -0.22) |
| American Samoa | 11.4 (8.2-15.3) | 22.5 (16.8-29.2) | 553.9 (405.2-733.3) | 453.7 (342.2-585.2) | -0.66 (-0.73, -0.6) |
| Andorra | 26.4 (19-35.9) | 53.4 (39.8-69.8) | 385.3 (280-520.4) | 257.2 (190.6-338.2) | -1.29 (-1.37, -1.22) |
| Angola | 2527.8 (1846.3-3417.9) | 6903.9 (5035.3-9019.5) | 769.7 (569.6-1022.3) | 695.1 (514.6-900.5) | -0.34 (-0.39, -0.28) |
| Antigua and Barbuda | 35.6 (27.3-45.3) | 45.6 (35.3-58.2) | 484.1 (369.6-617.6) | 389 (303.7-493.2) | -0.7 (-0.75, -0.66) |
| Argentina | 21148.5 (16089.5-27295.4) | 24298.2 (18775-30420.9) | 538.8 (412.4-692.4) | 330.5 (255.1-414.1) | -1.58 (-1.63, -1.53) |
| Armenia | 2232 (1693.8-2835.2) | 2640.7 (2095.8-3225.2) | 761.3 (578.2-967.4) | 460 (365.3-561.5) | -1.62 (-1.71, -1.53) |
| Australia | 14140.7 (11943.7-16366.8) | 19420.5 (15265.7-23756.5) | 569.4 (481.7-658.7) | 303.5 (237.5-372.4) | -2 (-2.07, -1.93) |
| Austria | 10131.8 (8525.6-11866.3) | 10758.6 (8321.8-13484.9) | 622.1 (523.4-728.7) | 416.3 (321.4-524.1) | -1.34 (-1.56, -1.12) |
| Azerbaijan | 2505.7 (1870.2-3299.7) | 5775.2 (4319.5-7453.9) | 470.7 (350.8-618.6) | 582.9 (437.1-750.2) | 0.69 (0.61, 0.78) |
| Bahamas | 71.1 (53.3-92.2) | 152.6 (115.5-193.3) | 431.9 (326.2-557.4) | 350.4 (267-441.9) | -0.67 (-0.7, -0.65) |
| Bahrain | 55.4 (41.7-71.4) | 192.1 (143.7-246.1) | 448.8 (347.3-564.6) | 317.7 (247.6-396.6) | -1.13 (-1.2, -1.05) |
| Bangladesh | 23096.3 (16910.6-30319.7) | 73924.4 (55266.7-95652.7) | 493.4 (365.8-642.2) | 501.3 (379.8-642) | 0.03 (-0.07, 0.13) |
| Barbados | 215.9 (168.3-271.6) | 274.6 (213-345.9) | 535 (416.8-673.4) | 406.8 (316.4-511.3) | -0.88 (-0.94, -0.83) |
| Belarus | 16844 (12884.6-21551.7) | 17639 (13465.6-22457) | 1041.6 (797.3-1332.8) | 824.1 (627.9-1051) | -0.78 (-0.89, -0.68) |
| Belgium | 12575.7 (9622.2-16025.2) | 11064.7 (8956.5-13168.1) | 609.6 (467.2-776.9) | 320.1 (257.4-382.2) | -2.08 (-2.14, -2.02) |
| Belize | 37.8 (28.4-48.7) | 92 (69.8-117.4) | 333.4 (251.1-429.4) | 292.4 (222.8-371.9) | -0.43 (-0.5, -0.37) |
| Benin | 1324.9 (971.5-1747.6) | 2561.9 (1897.7-3350) | 602.4 (444.5-790.7) | 511.1 (381.5-663.9) | -0.53 (-0.65, -0.4) |
| Bermuda | 34.1 (26-43.7) | 54.4 (40.8-70) | 473.4 (361.9-603.4) | 281.3 (210.7-362.8) | -1.67 (-1.77, -1.58) |
| Bhutan | 89.1 (63.9-120.5) | 264.2 (200.2-340.3) | 415.6 (301.4-555.7) | 385.7 (293.2-495.6) | -0.25 (-0.37, -0.13) |
| Bolivia (Plurinational State of) | 1178.5 (841.8-1600.6) | 2794.6 (2013.8-3761.4) | 370.5 (269.2-496.1) | 295.6 (216.6-392.5) | -0.74 (-0.77, -0.7) |
| Bosnia and Herzegovina | 4111.3 (3137.1-5261.7) | 7071.2 (5496.8-8904.3) | 972.4 (747.3-1235.4) | 851.4 (663-1070.5) | -0.41 (-0.54, -0.29) |
| Botswana | 513.1 (373.9-694.9) | 1425.9 (1066.7-1871.1) | 965.6 (713.8-1287.8) | 1044.7 (789-1354.9) | 0.24 (0.18, 0.29) |
| Brazil | 62667.1 (42035.9-88789.5) | 120158.3 (85029.4-162394.4) | 674.6 (457.9-946.2) | 394.4 (279.8-531.7) | -1.72 (-1.75, -1.68) |
| Brunei Darussalam | 98.3 (74.3-126.3) | 178.9 (134.1-231.3) | 949 (719.8-1216.9) | 539.1 (411.7-686.7) | -1.83 (-1.91, -1.75) |
| Bulgaria | 15433.8 (11654.6-19708.6) | 20368.8 (16029.9-25300.7) | 1096.2 (849.4-1374) | 1044.3 (823.8-1294.1) | -0.16 (-0.19, -0.14) |
| Burkina Faso | 1550.4 (1096.8-2119.8) | 3163.3 (2324.8-4175.6) | 362.6 (261.5-487.7) | 351 (261.4-458.1) | -0.1 (-0.17, -0.04) |
| Burundi | 1903 (1375.8-2604.3) | 2574.3 (1890.8-3400.2) | 781.2 (569.5-1057.8) | 581.2 (432.7-758.4) | -0.95 (-1.03, -0.88) |
| Cabo Verde | 116 (84.3-155.8) | 222.3 (163.8-294.3) | 382.4 (277.4-514.5) | 442.3 (325.2-585.5) | 0.48 (0.38, 0.58) |
| Cambodia | 2449.8 (1802.6-3273.8) | 7443.7 (5561.9-9640.2) | 596 (446.6-781.6) | 621.2 (471.3-793.6) | 0.13 (0.1, 0.16) |
| Cameroon | 2034.8 (1435.6-2784.9) | 5686.1 (4246.5-7512.6) | 477 (340.2-644.4) | 493.5 (372.6-646.3) | 0.1 (0.02, 0.17) |
| Canada | 25276.3 (19195.9-32637.2) | 31799.9 (25768.5-38506.4) | 606 (461.4-781.7) | 315.1 (254.7-382.4) | -2.09 (-2.15, -2.03) |
| Central African Republic | 661.7 (468.5-901) | 1086.7 (797.7-1448) | 677.7 (487.7-907.8) | 637.8 (476.1-836.8) | -0.21 (-0.29, -0.13) |
| Chad | 1590.3 (1150-2135.3) | 2683.4 (1984.6-3550.6) | 513.3 (374.8-682.8) | 494.5 (370.4-647.5) | -0.12 (-0.15, -0.09) |
| Chile | 6213 (4754.3-7925.6) | 10896.5 (8307.6-13774) | 533.6 (410.4-678.4) | 327.1 (249.2-413.6) | -1.58 (-1.63, -1.53) |
| China | 527692.4 (356178.9-739435.6) | 2210259.5 (1574847.9-2987286.9) | 623.9 (426.4-863.3) | 862.3 (618.6-1158.3) | 1.02 (0.94, 1.11) |
| Colombia | 8878.9 (6759.8-11368.6) | 18732.1 (14300.1-23921.5) | 482 (369.1-614.3) | 262.3 (200.4-334.7) | -1.98 (-2.04, -1.92) |
| Comoros | 143.2 (102.5-195.7) | 318.7 (235.6-418.9) | 746.3 (541.5-1006.1) | 637.7 (475.6-831.6) | -0.51 (-0.58, -0.45) |
| Congo | 895.6 (653.3-1204) | 1746.2 (1292-2307.1) | 863.6 (638.5-1145.7) | 754.7 (565-984.2) | -0.44 (-0.5, -0.37) |
| Cook Islands | 6.3 (4.6-8.3) | 14.3 (10.7-18.6) | 475.1 (350.2-626.7) | 431.3 (323.8-562.9) | -0.32 (-0.33, -0.3) |
| Costa Rica | 813.7 (618.5-1055.8) | 1965.5 (1500.3-2546.4) | 406.2 (309.5-525.7) | 279.3 (213.4-361.6) | -1.2 (-1.27, -1.14) |
| Coted'Ivoire | 2026.5 (1480.1-2732) | 5160.3 (3797.7-6745.6) | 597.7 (441.3-797.8) | 523.8 (388.9-679) | -0.44 (-0.48, -0.41) |
| Croatia | 7380.8 (6209-8574.8) | 8260.1 (7218.7-9354.6) | 1058.4 (892-1230) | 648.9 (566.1-736.2) | -1.57 (-1.61, -1.53) |
| Cuba | 4984.7 (3816-6384) | 9029 (6967.2-11401) | 400.6 (307.6-512.1) | 349.9 (269.3-442.1) | -0.44 (-0.53, -0.35) |
| Cyprus | 568.5 (431.8-733.3) | 659.1 (525.9-811.5) | 569.1 (439.2-725.7) | 270.3 (216.6-331.1) | -2.34 (-2.74, -1.94) |
| Czechia | 21286.7 (16294.4-26869) | 16852.7 (13187-21027.8) | 1208.5 (932.2-1519.9) | 560.4 (439-699) | -2.49 (-2.64, -2.34) |
| Democratic People's Republic of Korea | 12630.7 (9344.5-16741.9) | 31196.7 (23619.3-39558.3) | 795.2 (593.2-1046.6) | 813 (616-1031.6) | 0.07 (0.03, 0.1) |
| Democratic Republic of the Congo | 9532.1 (6784.6-12995) | 19171.3 (14087.9-25338.9) | 671.4 (485-898.7) | 602.6 (446.9-788.2) | -0.36 (-0.41, -0.31) |
| Denmark | 7326.3 (5882.2-8885) | 5077.1 (3909.7-6469.1) | 658.2 (528.4-799.1) | 301.8 (231.2-386.5) | -2.52 (-2.59, -2.45) |
| Djibouti | 79.6 (57.4-109.4) | 385.2 (285.6-505) | 728.5 (529.1-983.7) | 725.4 (543.9-939.9) | -0.02 (-0.14, 0.09) |
| Dominica | 29.4 (22-38.4) | 36.4 (27.3-46.4) | 393.9 (296.9-512.1) | 369.5 (279.3-470.3) | -0.19 (-0.21, -0.18) |
| Dominican Republic | 1334.6 (992.5-1740) | 5071.3 (3859.1-6529.4) | 342.3 (257.1-443.3) | 424.3 (322.9-546) | 0.69 (0.66, 0.73) |
| Ecuador | 2117.6 (1587.3-2739.6) | 5621.5 (4276.2-7200.8) | 368.1 (277.3-474.5) | 291.7 (222.8-372.5) | -0.77 (-0.84, -0.71) |
| Egypt | 15151.3 (11208.5-19923.4) | 41908 (31570.5-54201.9) | 639.4 (483.9-823.3) | 707.6 (543.8-900.8) | 0.33 (0.24, 0.41) |
| El Salvador | 1207.8 (907-1563.9) | 2201.7 (1670.9-2832.8) | 347.9 (261.7-449.4) | 260.1 (196.4-336.3) | -0.93 (-0.98, -0.88) |
| Equatorial Guinea | 139.5 (102-190) | 283.5 (208.3-378.9) | 773.4 (571.3-1037) | 641.2 (476.8-848.6) | -0.6 (-0.65, -0.55) |
| Eritrea | 583.2 (415.3-808.6) | 1420.2 (1029.1-1921.4) | 744.6 (541.1-1004.9) | 622.7 (456.7-827.8) | -0.57 (-0.66, -0.47) |
| Estonia | 2526.5 (2042.8-3079.1) | 1554.1 (1160.9-1999.3) | 988.4 (799.8-1204.3) | 395.9 (293.9-511.5) | -2.96 (-3.05, -2.86) |
| Eswatini | 206.9 (150.3-282.1) | 428.4 (319.7-561.6) | 784.8 (575.9-1055.2) | 892.7 (678-1154) | 0.4 (0.37, 0.44) |
| Ethiopia | 8996 (6004.2-12784.9) | 18137.3 (12768.9-24857.3) | 508.5 (346.8-711.5) | 428.4 (304.2-583.1) | -0.55 (-0.63, -0.46) |
| Fiji | 200.9 (148.2-267.7) | 417.8 (309.7-544.6) | 642.7 (478-849) | 551.3 (413.1-710.1) | -0.52 (-0.57, -0.47) |
| Finland | 7631.6 (6239.4-9187.1) | 8046.9 (6351-10000.3) | 804.7 (658-968.6) | 425.4 (334.4-530.1) | -2.05 (-2.09, -2.01) |
| France | 45774.2 (38649.9-53628.6) | 58529.4 (48719.1-69079.5) | 402.5 (339.4-471.6) | 280.8 (233.1-332.2) | -1.18 (-1.25, -1.1) |
| Gabon | 492.9 (355.2-659.4) | 714.9 (529.3-929.2) | 779.4 (565.7-1036.5) | 719.1 (537.4-926.2) | -0.27 (-0.31, -0.23) |
| Gambia | 192.4 (138-260.4) | 540.3 (405.1-710.6) | 573.2 (415.4-766.8) | 552.8 (417.6-721.7) | -0.12 (-0.15, -0.09) |
| Georgia | 5208.6 (3968.5-6737.1) | 6102.9 (4767.5-7610.8) | 717.8 (549.3-924.9) | 731.8 (570.3-913.8) | 0.07 (0.03, 0.11) |
| Germany | 133942.3 (109351.6-160230.2) | 127384.8 (103426.7-151597.5) | 791.3 (644.8-946.4) | 458.1 (369.9-548.2) | -1.74 (-1.82, -1.66) |
| Ghana | 4053 (2946.6-5389.8) | 11541.7 (8551.2-14883.9) | 689.2 (508.5-904.5) | 721.9 (542.4-923.8) | 0.13 (0.06, 0.21) |
| Greece | 16142.4 (12743.6-19964.3) | 17317 (13932.4-21226) | 845.6 (670.2-1041.5) | 445.7 (350.9-555.8) | -1.98 (-2.23, -1.73) |
| Greenland | 32.1 (24-42) | 37.8 (28.6-48.3) | 1207.7 (921.2-1543.7) | 550 (426.8-690.5) | -2.55 (-2.61, -2.49) |
| Grenada | 63.9 (50.1-80.1) | 60.6 (47.3-76) | 624.6 (487.3-787.4) | 529.4 (419.8-655.1) | -0.53 (-0.57, -0.49) |
| Guam | 38.8 (28.8-51.2) | 109.4 (82.8-141.7) | 550.4 (412.4-718.7) | 396.1 (300.5-511.7) | -1.05 (-1.13, -0.97) |
| Guatemala | 1109.4 (823.1-1440.5) | 3332.2 (2557.5-4202.1) | 347 (261.9-444.6) | 275.1 (212.6-345.4) | -0.75 (-0.78, -0.72) |
| Guinea | 1947.1 (1406.6-2611.7) | 3051.1 (2284.1-4011.3) | 533.5 (389.2-708.8) | 530.8 (401-692.8) | -0.03 (-0.12, 0.06) |
| Guinea-Bissau | 222.3 (157.9-303) | 345.2 (252-459.1) | 584.1 (421.7-782) | 559.7 (416.4-733) | -0.15 (-0.21, -0.1) |
| Guyana | 306.8 (234.8-389.2) | 382.1 (291.9-484) | 774.5 (598.8-973.7) | 573.6 (444.8-719.9) | -0.97 (-1.14, -0.8) |
| Haiti | 1694.6 (1242.2-2244.8) | 3289.4 (2477.6-4301.8) | 578.9 (435.4-750.1) | 517.7 (397.7-662.6) | -0.37 (-0.42, -0.33) |
| Honduras | 702 (503.9-942.5) | 2429 (1808.4-3163.9) | 349.5 (254.8-464.2) | 389.8 (295.1-502.3) | 0.37 (0.32, 0.41) |
| Hungary | 19750.2 (15489.3-24336) | 15362.6 (11780.1-19649.4) | 1066.4 (841.4-1309.6) | 557.5 (427.5-712.6) | -2.1 (-2.13, -2.07) |
| Iceland | 252.7 (191-325.7) | 247.3 (189.6-318.3) | 659.7 (497.8-851.8) | 302.5 (230.9-390.4) | -2.51 (-2.59, -2.42) |
| India | 165083.6 (111468.8-232678.9) | 389326.9 (273367.4-531306.6) | 393.8 (270.2-547.8) | 312.1 (221.8-422.3) | -0.76 (-0.81, -0.72) |
| Indonesia | 62941.2 (42435-88535.9) | 193866.9 (136829.5-265303.2) | 684.4 (466.3-952) | 854.9 (610.1-1154) | 0.73 (0.69, 0.76) |
| Iran (Islamic Republic of) | 15156.3 (10231.1-21367.2) | 37913.8 (26972.1-51515.7) | 640.7 (440.8-885.1) | 454.9 (324.7-615.1) | -1.11 (-1.22, -0.99) |
| Iraq | 6527.4 (4939.7-8382.1) | 18006.2 (13693.6-23156.7) | 743.7 (563.6-953.7) | 809.7 (624.8-1027.8) | 0.28 (0.23, 0.32) |
| Ireland | 3600.4 (2770.3-4661.3) | 2459.5 (1908.1-3126.6) | 677.6 (523.6-873.8) | 229.6 (177.3-293.3) | -3.47 (-3.78, -3.15) |
| Israel | 3705.5 (2771-4838.8) | 4425.6 (3322.2-5839.8) | 597 (449.5-776.7) | 261 (194.7-346.4) | -2.74 (-2.94, -2.54) |
| Italy | 75021.9 (49804.6-106766.9) | 59764.2 (45982.1-75207.1) | 640.9 (428-910) | 264.6 (201.5-335.9) | -2.81 (-2.87, -2.74) |
| Jamaica | 1205.3 (926.2-1545.4) | 1762 (1357.9-2234.8) | 508.4 (390.8-652.3) | 425.6 (326.3-539.5) | -0.59 (-0.66, -0.52) |
| Japan | 129882.7 (85987.6-185509.7) | 179032.6 (133754.5-231751.9) | 630.3 (420.2-896.7) | 319.7 (236.1-419) | -2.21 (-2.45, -1.96) |
| Jordan | 1122.1 (838-1455.3) | 5448.8 (4197.2-6816.4) | 935.8 (704.4-1203.9) | 781.6 (606.6-973.8) | -0.61 (-0.7, -0.53) |
| Kazakhstan | 14761.8 (11167.9-18889) | 16864.5 (12893.8-21268.5) | 1057.6 (803-1348.7) | 899.4 (694-1127.7) | -0.54 (-0.59, -0.48) |
| Kenya | 4819.8 (3251.5-6790.1) | 12439.9 (8706.8-17041.3) | 582.7 (396.7-814.6) | 597.6 (422.9-810.3) | 0.08 (0.04, 0.13) |
| Kiribati | 24 (17.6-31.8) | 41.4 (30.8-53.8) | 667.5 (494.7-876.6) | 632.4 (476.2-812.2) | -0.19 (-0.26, -0.12) |
| Kuwait | 234.7 (177-301.3) | 1089.9 (831.6-1390.1) | 471.9 (358.6-600.9) | 430.5 (329.3-545.5) | -0.31 (-0.59, -0.04) |
| Kyrgyzstan | 3066.7 (2313.2-3929.5) | 2964.3 (2248.4-3794.3) | 898.6 (678.2-1151.8) | 596 (452.6-758.9) | -1.33 (-1.41, -1.25) |
| Lao People's Democratic Republic | 1352.6 (992.7-1810.6) | 2830.7 (2108.8-3667.8) | 676.6 (504.7-889.7) | 636.1 (478.9-816.7) | -0.2 (-0.21, -0.18) |
| Latvia | 5667.3 (4392.9-7158.6) | 4944.8 (3914.9-6013.7) | 1257.4 (975.8-1584.3) | 853.3 (671.9-1041.3) | -1.26 (-1.35, -1.17) |
| Lebanon | 1389.4 (1030.9-1826.1) | 3898.6 (2954.2-5058.5) | 619.3 (463.5-807.3) | 493.9 (373.4-642.8) | -0.75 (-0.77, -0.73) |
| Lesotho | 578.2 (421.1-774.1) | 936.6 (686.7-1222.1) | 636.5 (466-846.7) | 920.4 (686.9-1183) | 1.21 (1.14, 1.27) |
| Liberia | 677.2 (487.7-912.7) | 823.8 (611.2-1088.6) | 544.5 (396.7-725.3) | 442.1 (331.7-579.3) | -0.68 (-0.7, -0.66) |
| Libya | 823.5 (622.4-1064.1) | 2289.6 (1726.9-2970.7) | 408.7 (309.5-525.9) | 455.5 (345-588.9) | 0.36 (0.34, 0.37) |
| Lithuania | 6944.6 (5383.3-8467.8) | 7470.9 (5799.2-9373.7) | 1210.5 (938.9-1477.1) | 904.8 (698.5-1141.5) | -0.97 (-1.27, -0.66) |
| Luxembourg | 431.2 (339.3-533.8) | 357.2 (291.8-425.5) | 619.9 (489.5-765.2) | 245.4 (199.6-293.4) | -2.97 (-3.04, -2.9) |
| Madagascar | 3800.4 (2774.8-5079.8) | 7140.2 (5324.3-9326.2) | 748.4 (551.8-991.6) | 753.7 (567.9-975.2) | 0.04 (-0.06, 0.13) |
| Malawi | 2490.9 (1809-3340.1) | 4491.2 (3335.5-5859.1) | 675.5 (497.1-894.7) | 649.8 (489.7-841.3) | -0.12 (-0.18, -0.07) |
| Malaysia | 6936.8 (5145.3-9128.2) | 17222.2 (12932.3-22470.6) | 707.3 (525.6-928.1) | 547.8 (415.7-709) | -0.82 (-0.87, -0.78) |
| Maldives | 54.8 (39.8-73.9) | 146.9 (111.5-188.5) | 698.7 (516.8-927) | 455.8 (346.2-584.1) | -1.38 (-1.42, -1.35) |
| Mali | 1812.9 (1306.3-2437) | 3279.9 (2425.8-4295.8) | 491.9 (360.5-649.2) | 419.8 (317.2-540.6) | -0.52 (-0.55, -0.48) |
| Malta | 316.3 (241.6-402.1) | 328.5 (257-412.7) | 601.1 (462.1-760.1) | 229.3 (178.7-289.3) | -3.1 (-3.48, -2.73) |
| Marshall Islands | 9.8 (7.1-13.2) | 16.6 (12.3-21.9) | 620.7 (457.5-823.4) | 585.1 (441.3-757.9) | -0.21 (-0.33, -0.1) |
| Mauritania | 692.7 (499.9-936.1) | 1114.6 (823.1-1464.9) | 647.3 (472-866.6) | 492.7 (367.1-642.4) | -0.88 (-0.91, -0.84) |
| Mauritius | 713.6 (539.4-926.9) | 1063.3 (804.2-1350.6) | 929 (709.7-1195.1) | 480.7 (366.5-607.3) | -2.15 (-2.3, -2) |
| Mexico | 20655.4 (14037.5-28920.7) | 42805.6 (30343.9-58564.8) | 473.2 (323.9-659.8) | 296.9 (211.5-404.6) | -1.5 (-1.59, -1.42) |
| Micronesia (Federated States of) | 33.3 (24.1-44.7) | 40 (29.8-52.2) | 648.3 (474.8-859) | 601.8 (452.9-774.1) | -0.25 (-0.29, -0.22) |
| Monaco | 73.4 (55.5-95.1) | 54.2 (41.1-70.3) | 707.1 (531.8-921.7) | 370.5 (278.9-483.7) | -2.09 (-2.17, -2.01) |
| Mongolia | 341.1 (243.6-461.3) | 761.8 (554-1006) | 303.2 (219.2-406.8) | 351.3 (255.9-462) | 0.46 (0.37, 0.56) |
| Montenegro | 339.3 (250-442) | 591.4 (453.9-754.3) | 471.9 (349.1-612.2) | 501.1 (388.5-634.7) | 0.22 (0.14, 0.3) |
| Morocco | 9992.2 (7562.4-13046.8) | 26113.9 (20105.3-33250.2) | 647.8 (494.4-838.8) | 697.1 (541.7-881.5) | 0.23 (0.17, 0.29) |
| Mozambique | 4183.9 (3041.6-5578.6) | 8365 (6212.6-10979.4) | 713.8 (524.3-943.2) | 792.2 (593.3-1032.1) | 0.34 (0.22, 0.45) |
| Myanmar | 14963.8 (10971.9-19960.2) | 30091.7 (22958.5-38564.9) | 652.5 (484.1-857.2) | 584.2 (448.4-743.2) | -0.36 (-0.43, -0.3) |
| Namibia | 607.3 (440-806.4) | 1194.6 (894.7-1537.8) | 957.7 (702-1256.2) | 904.7 (683.5-1156) | -0.19 (-0.26, -0.13) |
| Nauru | 2.7 (1.9-3.7) | 2.7 (2-3.5) | 706.8 (516.7-948.9) | 469.5 (351.2-611.9) | -1.3 (-1.38, -1.22) |
| Nepal | 3284.6 (2336.1-4517.5) | 8718.7 (6442.5-11424) | 380.1 (274.9-513.2) | 359.9 (269.5-466.3) | -0.18 (-0.2, -0.16) |
| Netherlands | 17993.7 (14192.8-22317.7) | 15994.1 (12163.5-20649.4) | 679.3 (535.4-843.5) | 324 (245.1-420.4) | -2.39 (-2.5, -2.29) |
| New Zealand | 2785.2 (1922.9-3896.5) | 3542.9 (2614.3-4619.6) | 552.3 (383.1-770) | 304.5 (224.4-397.8) | -1.9 (-1.93, -1.88) |
| Nicaragua | 790.9 (585.3-1041.4) | 1860.1 (1410.4-2379.1) | 497.7 (370.5-651.6) | 342.9 (260.5-437.8) | -1.21 (-1.26, -1.16) |
| Niger | 1352 (966-1837.6) | 3463.3 (2539-4570.9) | 536.7 (389.5-718.6) | 455.7 (338.9-594.3) | -0.53 (-0.57, -0.48) |
| Nigeria | 25845.5 (17416.4-36500.7) | 42701.1 (29967-58355.5) | 562.6 (383.6-786.9) | 501.7 (354.5-680) | -0.38 (-0.4, -0.36) |
| Niue | 1.9 (1.4-2.5) | 1.4 (1.1-1.8) | 626.7 (460.9-836.6) | 529.9 (401.3-684.5) | -0.55 (-0.63, -0.47) |
| North Macedonia | 2962.8 (2309.9-3727.4) | 4824.5 (3763-5969.3) | 1472.6 (1157.4-1836.9) | 1475.4 (1181.1-1791.1) | 0.01 (-0.09, 0.11) |
| Northern Mariana Islands | 5.6 (4-7.6) | 21.9 (16.1-28.6) | 476 (348.1-642.1) | 440.3 (328-568.6) | -0.25 (-0.32, -0.18) |
| Norway | 7892.9 (5239.1-11214.8) | 6574.7 (4766.6-8757.1) | 819 (543.9-1168.2) | 462 (334-617.3) | -1.85 (-2.06, -1.64) |
| Oman | 395.4 (293.3-518.7) | 989.5 (752-1253.8) | 635.3 (477-825.8) | 643.7 (497.2-805.8) | 0.03 (-0.06, 0.12) |
| Pakistan | 29879.7 (20098.2-42257.1) | 54482.6 (38147.2-74657.9) | 506.7 (345.5-710.2) | 477.7 (337.8-648.4) | -0.19 (-0.23, -0.16) |
| Palau | 6.7 (4.8-8.9) | 13.3 (9.9-17.4) | 640.7 (468.2-849.9) | 599 (453.8-777.5) | -0.24 (-0.29, -0.19) |
| Palestine | 668.6 (509.3-856.8) | 1665.8 (1309.4-2060.6) | 757 (583.9-958.2) | 778.4 (623.6-948.8) | 0.06 (-0.17, 0.29) |
| Panama | 816.9 (621.9-1039.5) | 1926.4 (1473.3-2447.2) | 486.8 (372.6-616.9) | 338.7 (258.8-430.6) | -1.17 (-1.2, -1.13) |
| Papua New Guinea | 580.1 (409.1-796.7) | 1552.6 (1156.5-2035.1) | 382.3 (275-517.4) | 360.3 (271.3-467.3) | -0.2 (-0.22, -0.17) |
| Paraguay | 1326.4 (1001.2-1720.8) | 2818.1 (2128-3629.4) | 529.2 (401.5-683.5) | 419.3 (317.8-537.8) | -0.75 (-0.83, -0.67) |
| Peru | 4468.5 (3315.3-5895) | 9029.2 (6778.1-11771.6) | 339.9 (252.8-447.5) | 218.6 (163.9-285) | -1.43 (-1.53, -1.34) |
| Philippines | 11261.8 (7609-15804.8) | 39961.2 (28131.8-54669.8) | 396.7 (271-551.8) | 470.6 (333.7-638.4) | 0.55 (0.49, 0.61) |
| Poland | 35336.7 (24080.5-49456.8) | 50726 (36455.3-68258.5) | 671.2 (460.6-934.5) | 507.5 (363.6-684) | -0.92 (-1.01, -0.82) |
| Portugal | 18237.7 (14241.6-22554.5) | 11623.8 (9447.7-14033.5) | 1065.5 (841-1307.9) | 301.9 (242.4-368.5) | -4.03 (-4.21, -3.84) |
| Puerto Rico | 1423.1 (1075.8-1855.1) | 2289 (1744.2-2947.7) | 327.9 (249.8-425.1) | 200.5 (150.9-261.5) | -1.59 (-1.65, -1.53) |
| Qatar | 44.7 (33.9-58.3) | 206.2 (153.1-272) | 645.7 (495.7-825.3) | 446.5 (341.4-573.4) | -1.21 (-1.5, -0.91) |
| Republic of Korea | 34889.1 (26300.1-44934.9) | 48509.2 (37219.4-61512.9) | 1180.4 (901.8-1502.1) | 401.1 (308.6-507.7) | -3.46 (-3.73, -3.2) |
| Republic of Moldova | 3761 (2847-4853.4) | 5020.3 (3836.6-6313.7) | 785.1 (602.5-999.6) | 636.1 (487-798.8) | -0.7 (-0.77, -0.63) |
| Romania | 37323.2 (28733.6-47646.7) | 43203.6 (34107.5-53090) | 1181.8 (923.3-1487.4) | 820.8 (646.8-1010.9) | -1.18 (-1.22, -1.15) |
| Russian Federation | 242495.3 (165490.1-337996.7) | 273013.1 (197046.4-366682.8) | 1181.2 (814.7-1633.6) | 862.5 (620.6-1159.4) | -1.01 (-1.18, -0.84) |
| Rwanda | 2169.3 (1528-3000.6) | 3530.2 (2525.3-4724.7) | 797.2 (569.6-1085.7) | 602.1 (436-799.6) | -0.91 (-1, -0.82) |
| Saint Kitts and Nevis | 41.4 (31.8-53.1) | 39.4 (30.2-50.2) | 847.7 (659.4-1073.1) | 575.3 (449.7-721.3) | -1.25 (-1.29, -1.21) |
| Saint Lucia | 69.5 (52.6-89.3) | 122.6 (95-154) | 708 (545.1-896.4) | 421.1 (326.9-528.2) | -1.67 (-1.79, -1.56) |
| Saint Vincent and the Grenadines | 48.1 (36.9-61.4) | 70.2 (54.4-88.7) | 560.7 (433.2-710.6) | 412.4 (321.6-519.5) | -1.01 (-1.09, -0.94) |
| Samoa | 52.2 (37.9-71.1) | 79.6 (60-102.4) | 586.9 (430.5-791) | 510.2 (385.9-654.2) | -0.47 (-0.49, -0.44) |
| San Marino | 26.5 (19.7-34.8) | 35.3 (26.7-45.9) | 553.3 (412.9-728.4) | 315.8 (235.2-416.7) | -1.79 (-1.84, -1.74) |
| Sao Tome and Principe | 45.8 (33.4-60.6) | 68.9 (51.1-90.3) | 620.2 (455.8-817.9) | 637.4 (477.8-830.2) | 0.08 (-0.05, 0.2) |
| Saudi Arabia | 3448.5 (2609.5-4468.3) | 7421 (5727.1-9378.8) | 657.5 (504.4-841.4) | 546.3 (428.6-680.5) | -0.64 (-0.7, -0.58) |
| Senegal | 1927 (1378.1-2601.8) | 3914.8 (2917.6-5122.6) | 575.5 (416.3-768.9) | 497.4 (374.3-645.5) | -0.47 (-0.55, -0.4) |
| Serbia | 14846.9 (11391.1-18775.7) | 22885.9 (18423.5-27800.8) | 1240.8 (962.9-1555) | 1027.7 (830.4-1244.8) | -0.62 (-0.68, -0.56) |
| Seychelles | 42.1 (31.7-55.5) | 60.3 (45.9-77.5) | 590.5 (445.4-776.7) | 476.5 (364.1-607.9) | -0.7 (-0.73, -0.67) |
| Sierra Leone | 1320.3 (950.2-1768) | 1920.8 (1428-2503.9) | 584.3 (424.6-776.2) | 516.2 (388.5-668) | -0.41 (-0.52, -0.3) |
| Singapore | 2046.6 (1544.8-2624.3) | 2904.3 (2167.3-3851) | 855 (650-1091.4) | 269.3 (202-355.2) | -3.73 (-3.85, -3.62) |
| Slovakia | 8084.9 (6144.9-10245.8) | 8937.8 (6825.5-11274) | 1058.7 (809.1-1338.8) | 704.8 (539.8-887.1) | -1.33 (-1.38, -1.28) |
| Slovenia | 2693.9 (2052.5-3446.6) | 2408.7 (1912.4-2953.1) | 885.6 (677.9-1128.5) | 369.9 (292.6-455) | -2.8 (-2.85, -2.74) |
| Solomon Islands | 86.9 (62.3-117.8) | 231 (171.6-300.8) | 744.5 (543.4-991.6) | 760.1 (571.6-982) | 0.06 (0.03, 0.08) |
| Somalia | 1115.6 (781.7-1549.5) | 2742.7 (2014.9-3694) | 595.1 (422.8-814.8) | 543.6 (406.6-719.9) | -0.28 (-0.33, -0.24) |
| South Africa | 16044.7 (10848-22519.9) | 36888.3 (25995.6-50568.6) | 719.4 (488.1-1005.7) | 755.6 (535.9-1029) | 0.15 (0.01, 0.29) |
| South Sudan | 1633.5 (1175.5-2245.5) | 1853.9 (1368.2-2449.6) | 609.5 (443.8-827.1) | 561.3 (418.5-732.2) | -0.26 (-0.3, -0.22) |
| Spain | 51280.1 (39175.7-65551.2) | 43411.6 (37060.8-49868.9) | 719.5 (552.2-916.5) | 313.5 (267.1-361.4) | -2.67 (-2.89, -2.45) |
| Sri Lanka | 6755.5 (5014.7-8791.7) | 16415.7 (12500.2-21128.8) | 653.4 (493.1-839.2) | 528.1 (407.9-673.8) | -0.72 (-0.76, -0.68) |
| Sudan | 6075.3 (4513.9-7933) | 12272 (9335.5-15705.3) | 646.2 (488.8-833.1) | 651.1 (500.6-825.6) | 0.02 (-0.08, 0.11) |
| Suriname | 137 (104-176) | 326.6 (248.8-412.6) | 488.8 (371.7-626.8) | 445.1 (340.4-561.2) | -0.31 (-0.36, -0.25) |
| Sweden | 13957.1 (9703.2-19120.4) | 12529.2 (8919.9-17058.9) | 645.2 (447.6-888.5) | 380.5 (269.7-520.8) | -1.7 (-1.77, -1.64) |
| Switzerland | 6680.4 (5271.7-8348.8) | 6499 (5089.8-8194.1) | 462.5 (363.6-579.9) | 246.9 (191.7-313.7) | -2.01 (-2.03, -1.98) |
| Syrian Arab Republic | 2943.4 (2223.7-3796.5) | 6170 (4718.1-7882.4) | 562.4 (429.4-718) | 488.4 (382.1-612.7) | -0.45 (-0.49, -0.41) |
| Taiwan (Province of China) | 14569.6 (11128.4-18499.6) | 27940.4 (20922.1-36481.5) | 863 (662.7-1088.9) | 499.1 (373.8-649.7) | -1.77 (-1.83, -1.72) |
| Tajikistan | 1841.8 (1382.9-2403.5) | 4266.8 (3222.2-5432.3) | 607.2 (456.5-791.3) | 828.5 (631.7-1051.6) | 1.01 (0.89, 1.13) |
| Thailand | 18035 (13253.1-23872.4) | 48224.8 (36214.3-62306.6) | 505.9 (374.1-666) | 347.3 (261-448.2) | -1.23 (-1.3, -1.16) |
| Timor-Leste | 112.9 (82.2-151.4) | 554.2 (415-716.6) | 513.2 (378.1-679.7) | 584.4 (443.7-746) | 0.42 (0.38, 0.47) |
| Togo | 683.9 (495.3-919.7) | 1764.1 (1301.6-2319.8) | 591.2 (433.2-786.5) | 524.8 (393.5-681.3) | -0.39 (-0.43, -0.34) |
| Tokelau | 0.9 (0.6-1.2) | 0.8 (0.6-1) | 517.5 (380.3-693.1) | 408.4 (306.9-528.4) | -0.77 (-0.82, -0.73) |
| Tonga | 24.9 (18.1-33.4) | 39.7 (29.9-51.5) | 428.4 (314.1-570.2) | 421.6 (317.7-546) | -0.06 (-0.09, -0.03) |
| Trinidad and Tobago | 613 (463.4-787.1) | 993.9 (755.7-1275.3) | 645 (493.6-821.3) | 413.9 (316.6-528.3) | -1.45 (-1.48, -1.43) |
| Tunisia | 2696.6 (2003.1-3525.2) | 8002.3 (6019.2-10352.5) | 535.4 (404.4-689.7) | 521.8 (395.8-670.5) | -0.1 (-0.27, 0.07) |
| Turkey | 23213.6 (17237.1-30788.2) | 49871.2 (37261.9-64899.2) | 647.8 (483.6-854) | 458.9 (344.7-594.8) | -1.14 (-1.2, -1.08) |
| Turkmenistan | 1426.6 (1077.7-1835.4) | 3293.1 (2495.7-4226.9) | 703.4 (533.9-900.8) | 766.6 (582.4-980) | 0.29 (0.04, 0.55) |
| Tuvalu | 3.7 (2.7-5.1) | 6.1 (4.6-7.9) | 556 (405.7-738.7) | 528.8 (400.8-682.2) | -0.17 (-0.2, -0.14) |
| Uganda | 4471.8 (3176.6-6128.6) | 9083.9 (6654.6-12020.8) | 690.6 (495.3-936.5) | 664.7 (489.6-874) | -0.12 (-0.18, -0.05) |
| Ukraine | 107585.4 (72598.2-151606.4) | 82994.4 (59345.6-112732.7) | 1238.2 (842.2-1735.2) | 794.8 (567.8-1081) | -1.44 (-1.5, -1.37) |
| United Arab Emirates | 280.5 (210.7-370.6) | 1469.9 (1078.6-1959.8) | 1032.5 (792.4-1336.2) | 763.3 (588.6-974.1) | -0.98 (-1.12, -0.84) |
| United Kingdom | 81345.8 (58279.9-108596.9) | 60568.8 (46932.6-77611.9) | 655.6 (469.8-877.4) | 326.4 (252-420.5) | -2.23 (-2.31, -2.16) |
| United Republic of Tanzania | 6416.4 (4685.3-8590.8) | 17701.4 (13072.8-23100.4) | 577.6 (425.2-765.8) | 698.5 (519-905.9) | 0.6 (0.49, 0.72) |
| United States of America | 224254.6 (152071.8-314437.1) | 244832.5 (173422.4-335870.9) | 518.3 (352-727.5) | 303.9 (215.1-417.1) | -1.7 (-1.74, -1.66) |
| United States Virgin Islands | 28.5 (20.8-37.8) | 82.4 (63-105.9) | 341.4 (253-449.4) | 335 (257.3-429.7) | -0.07 (-0.12, -0.03) |
| Uruguay | 3502.1 (2700.3-4431.8) | 3127.2 (2426.3-3934.2) | 691.5 (535.4-873.1) | 392.4 (301.8-498) | -1.85 (-1.93, -1.76) |
| Uzbekistan | 8933.1 (6790.5-11390.1) | 21708 (16683.9-27499.9) | 676.2 (513-863.7) | 853.2 (661.5-1075.6) | 0.74 (0.61, 0.87) |
| Vanuatu | 36.6 (25.9-49.8) | 102.4 (76.5-133.9) | 650 (468.2-868.9) | 631.6 (479-814.9) | -0.1 (-0.19, -0.02) |
| Venezuela (Bolivarian Republic of) | 4552.1 (3416.4-5935.6) | 11944.4 (9116.8-15215.8) | 447.2 (337.1-580) | 339.6 (260.8-430.3) | -0.86 (-0.92, -0.8) |
| Viet Nam | 29421.1 (22071-38493.7) | 71846.4 (57734.6-87872.9) | 651.6 (492.1-847.3) | 664.4 (536.2-811.9) | 0.03 (-0.06, 0.12) |
| Yemen | 2852.1 (2111.4-3738.1) | 8304.3 (6338.8-10492.5) | 636.4 (479.1-820.4) | 629.6 (487.1-786.5) | -0.03 (-0.05, -0.01) |
| Zambia | 1764.1 (1283.3-2394.7) | 4859.2 (3593.6-6299.4) | 663.5 (487.2-892.3) | 795.6 (594.2-1024.9) | 0.58 (0.52, 0.64) |
| Zimbabwe | 2441.7 (1813.5-3176.7) | 4421.9 (3227-5797.1) | 622.2 (467.5-802.5) | 727.9 (546.3-933.9) | 0.51 (0.44, 0.59) |

ASIR = age-standardized incidence rate; AAPC=average annual percentage change; CI=confidence interval; SDI=sociodemographic index; UI=uncertainty interval.
