# Supplementary material for: Global burden of ischemic stroke in adults aged 60 years and older from 1990 to 2021: Population-based study
Source: PLoS One. 2025 May 5;20(5):e0322606. doi: 10.1371/journal.pone.0322606 (PMC12052125; doi:10.1371/journal.pone.0322606)
Supplement: S3 Table — (DOCX) [file pone.0322606.s014.docx]

Table 3. The ASDR and ASMR of ischemic stroke in 1990 and 2021 and its trends.

|  | ASDR | | | ASMR | | |
| --- | --- | --- | --- | --- | --- | --- |
|  | 1990 year | 2021 year | AAPC（95%CI） | 1990 year | 2021 year | AAPC（95%CI） |
| Global | | | | | | |
| Age ≥60 group | 8771.6 (8030.6-9425.3) | 5607.6 (5033.4-6086.4) | -1.42 (-1.61, -1.23) | 554.8 (499.3-595.2) | 333.8 (293.5-364.2) | -1.62 (-1.82, -1.41) |
| All age groups | 1286.31 (1195.19-1376.06) | 837.36 (763.73-904.98) | -1.37 (-1.53, -1.2) | 73.15 (66.36-77.94) | 44.18 (39.29-47.81) | -1.6 (-1.81, -1.39) |
| Sex | | | | | | |
| Male | 9414.8 (8559.5-10311) | 6541.9 (5886.7-7192.2) | -1.16 (-1.32, -1) | 576.8 (521.3-629.2) | 384.3 (341.6-425.2) | -1.16 (-1.32, -1) |
| Female | 8214.8 (7424.8-8823) | 4830.9 (4217.2-5360.4) | -1.68 (-1.88, -1.48) | 533.7 (473.6-572.6) | 293.9 (250.3-327.2) | -1.68 (-1.88, -1.48) |
| SDI quintiles | | | | | | |
| High SDI | 6408 (5814.7-6812.5) | 2584.4 (2235.4-2860.3) | -2.91 (-3.08, -2.74) | 415.3 (367.3-439.6) | 148.5 (122.9-162.7) | -3.31 (-3.54, -3.07) |
| High-middle SDI | 13007.5 (12029.1-13719.3) | 7361.9 (6557.6-8086.6) | -1.8 (-2.12, -1.48) | 852.5 (777.8-898) | 455 (396.7-502.3) | -1.98 (-2.35, -1.6) |
| Middle SDI | 8175.7 (7294.8-9213) | 6518.6 (5762-7211.2) | -0.76 (-0.95, -0.57) | 499.8 (441.1-564.7) | 391.4 (339.5-435.9) | -0.82 (-1.06, -0.58) |
| Low-middle SDI | 7368.3 (6432-8530.4) | 6348.9 (5621.1-7215.2) | -0.44 (-0.59, -0.29) | 444.2 (382-515.3) | 383.9 (336.1-435.8) | -0.43 (-0.57, -0.3) |
| Low SDI | 7289.1 (6066-9167.7) | 6212.6 (5257.9-7618.3) | -0.5 (-0.59, -0.4) | 431.4 (354.2-545.3) | 374.5 (312.5-460.1) | -0.42 (-0.56, -0.28) |
| Age group | | | | | | |
| 60-64years | 3133.4 (2910.4-3412.6) | 2061.9 (1878.7-2245.1) | -1.31 (-1.49 to -1.14) | 94.8 (87.6-104.4) | 58.7 (54-64.4) | -1.5 (-1.73 to -1.28) |
| 65-69years | 4686.8 (4354.6-5135.2) | 3226.7 (2936.6-3509.3) | -1.13 (-1.32 to -0.93) | 169.6 (157.8-186.4) | 110.2 (100.9-121.1) | -1.33 (-1.57 to -1.09) |
| 70-74years | 8486.3 (7940.8-9196.8) | 5652 (5208.1-6102.4) | -1.29 (-1.51 to -1.06) | 385.8 (360.6-417.9) | 244.2 (225.3-265) | -1.44 (-1.69 to -1.19) |
| 75-79years | 12697.6 (11962.3-13486.9) | 7781.8 (7125.1-8404.2) | -1.59 (-1.71 to -1.47) | 732.8 (685.7-779) | 423.3 (387.4-459.8) | -1.78 (-1.92 to -1.65) |
| 80-84years | 18501.8 (16984.8-19563.6) | 11566.4 (10395.3-12475.3) | -1.49 (-1.8 to -1.18) | 1384.6 (1270.2-1466.4) | 826.5 (734-890.2) | -1.63 (-1.91 to -1.36) |
| 85-90years | 23996.3 (21189-25525.9) | 14895.5 (12996.4-16201.4) | -1.53 (-1.68 to -1.39) | 2290.4 (2004.7-2430.7) | 1366.8 (1173.6-1490.2) | -1.64 (-1.8 to -1.49) |
| 90-94years | 30170.3 (25599.2-32556.2) | 18373.6 (15207.5-20247.8) | -1.58 (-1.84 to -1.32) | 3347.8 (2818.3-3611.8) | 1967.1 (1597.9-2173.6) | -1.69 (-1.95 to -1.42) |
| 95+years | 36417.3 (28864.1-40144.2) | 22000.7 (16899.7-24936.5) | -1.55 (-1.72 to -1.38) | 4299.7 (3352.6-4748.6) | 2530 (1872.2-2879) | -1.69 (-1.91 to -1.46) |
| GBD Region | | | | | | |
| Andean Latin America | 3782.4 (3227.3-4370.7) | 2149.0 (1748.8-2617.8) | -1.78 (-2.3, -1.26) | 238.3 (200.4-278.1) | 134.8 (108-166.8) | -1.8 (-2.34, -1.25) |
| Australasia | 5228.2 (4634.1-5710.9) | 1726.5 (1441.3-1956.4) | -3.58 (-3.77, -3.39) | 358.5 (309.3-394.1) | 110.8 (88.9-125.3) | -3.78 (-4.01, -3.55) |
| Caribbean | 6044.5 (5471.3-6562.3) | 4328.9 (3745.5-4952.4) | -1.05 (-1.48, -0.63) | 398.5 (358.9-430.4) | 272.5 (234-311.6) | -1.18 (-1.74, -0.62) |
| Central Asia | 10437.1 (9536.1-11153.3) | 9042.3 (8130.7-9880.8) | -0.38 (-0.69, -0.08) | 604.1 (546-647.2) | 534.5 (474.4-585.5) | -0.25 (-0.83, 0.32) |
| Central Europe | 16421.4 (15462.9-17111.6) | 7802.8 (7060.1-8417.9) | -2.39 (-2.62, -2.16) | 1085.1 (1009.6-1130.9) | 505.9 (450-546.7) | -2.49 (-2.7, -2.27) |
| Central Latin America | 4345.3 (4058.3-4567) | 2274.9 (2019.5-2533.2) | -2.15 (-2.43, -1.86) | 284.5 (262.3-298.7) | 142.3 (123.7-159.3) | -2.23 (-2.56, -1.9) |
| Central Sub-Saharan Africa | 8452 (6462.9-10929.6) | 7646.8 (5637.2-10315.1) | -0.31 (-0.37, -0.25) | 494.4 (368.5-650.4) | 459.6 (327.3-636.6) | -0.23 (-0.31, -0.15) |
| East Asia | 9200.7 (7864.8-10754.4) | 7983 (6705-9196.7) | -0.45 (-0.65, -0.25) | 557.3 (471.7-655.6) | 480.6 (394.5-561.7) | -0.46 (-0.73, -0.19) |
| Eastern Europe | 19582.2 (18458.1-20264.1) | 10818.7 (9785.1-11716.9) | -1.89 (-2.37, -1.4) | 1277.6 (1188.6-1322.6) | 687.9 (611.5-748.6) | -1.98 (-2.51, -1.44) |
| Eastern Sub-Saharan Africa | 6802.1 (5532.1-8474.2) | 6114.9 (5071.8-7275.2) | -0.34 (-0.41, -0.27) | 396.8 (316-503.9) | 356.2 (284.4-433.1) | -0.34 (-0.4, -0.28) |
| High-income Asia Pacific | 7417.9 (6568.3-8011.7) | 2225.2 (1856.3-2528.2) | -3.85 (-4.17, -3.54) | 487 (418.6-526.5) | 122.2 (95.9-138.4) | -4.41 (-4.87, -3.96) |
| High-income North America | 3761.1 (3324.4-4110.4) | 2268.6 (1936.2-2537.7) | -1.55 (-1.7, -1.4) | 229.1 (195.8-246.6) | 129.5 (105.1-142.2) | -1.79 (-2.34, -1.24) |
| North Africa and Middle East | 12553.6 (10764.9-14358.7) | 8676.4 (7458.2-9808.4) | -1.15 (-1.37, -0.93) | 791.4 (668.6-907.8) | 549.9 (467.5-623.1) | -1.14 (-1.33, -0.95) |
| Oceania | 6266.2 (4890.2-8094.9) | 5293.5 (4221.1-6840.6) | -0.56 (-0.6, -0.51) | 369.4 (282.4-485.2) | 310 (240.5-410.2) | -0.58 (-0.62, -0.53) |
| South Asia | 5540.9 (4568-6920) | 4752.9 (4085.9-5848.4) | -0.42 (-0.84, 0) | 328.3 (267.4-410.5) | 289.4 (246-352.2) | -0.37 (-0.88, 0.15) |
| Southeast Asia | 9089.9 (7890.1-10288.7) | 8539.5 (7241.5-9763.5) | -0.21 (-0.28, -0.13) | 548.4 (466.4-628.9) | 515.5 (433.2-591.6) | -0.2 (-0.27, -0.13) |
| Southern Latin America | 6947.4 (6266.9-7514.1) | 2736.9 (2428.3-3011) | -2.99 (-3.26, -2.71) | 445.5 (395.9-482.8) | 164.7 (142.6-181.4) | -3.18 (-3.51, -2.84) |
| Southern Sub-Saharan Africa | 6217.1 (5162.2-7109.5) | 7639.5 (6840.2-8435.2) | 0.68 (0.22, 1.13) | 361.1 (290.5-418.1) | 465.9 (410.7-517.7) | 0.9 (0.46, 1.33) |
| Tropical Latin America | 9377.8 (8599.6-9877.6) | 3606.6 (3211-3878) | -2.97 (-3.29, -2.65) | 606.2 (543-642.6) | 225.4 (193.6-244.2) | -3.08 (-3.26, -2.89) |
| Western Europe | 7034.5 (6360.4-7457.3) | 2086.8 (1781.8-2314.5) | -3.85 (-3.98, -3.72) | 490.3 (431.7-520.3) | 130.7 (106.9-143.7) | -4.19 (-4.35, -4.02) |
| Western Sub-Saharan Africa | 9589 (7621.7-12300.5) | 8515.9 (7136.4-10102.1) | -0.39 (-0.46, -0.32) | 577.7 (449.6-747) | 517.2 (429-617.4) | -0.35 (-0.43, -0.27) |
| 204 countries and regions | | | | | | |
| Afghanistan | 14307.3 (9831.8-20273.5) | 12914.3 (9067.6-17558.7) | -0.31 (-0.38, -0.25) | 860 (587.3-1216.4) | 791.1 (550.2-1072.7) | -0.26 (-0.31, -0.2) |
| Albania | 6618.8 (5241.7-8155.1) | 5008.7 (3700.2-6581.4) | -0.96 (-1.52, -0.4) | 423.9 (328.5-529.8) | 352.5 (252.6-471.4) | -0.66 (-1.3, -0.01) |
| Algeria | 12362.7 (9409.2-15656.8) | 8596.3 (6411.8-10969.2) | -1.14 (-1.27, -1) | 883.6 (670.7-1119.4) | 627.4 (461.7-804.7) | -1.08 (-1.23, -0.93) |
| American Samoa | 6110.2 (4914.3-7516.6) | 4631.6 (3605.6-5849) | -0.87 (-0.99, -0.75) | 360.3 (277.9-455.2) | 271.6 (201-355.6) | -0.88 (-1.02, -0.73) |
| Andorra | 3383.3 (2433.8-4541.1) | 1680.4 (1217.8-2207.9) | -2.32 (-2.87, -1.78) | 224.6 (153.2-309) | 108.4 (72.1-150.1) | -2.45 (-2.89, -2.01) |
| Angola | 8743.8 (6383.6-11556.8) | 8292 (6149.1-10837.3) | -0.17 (-0.24, -0.1) | 498.5 (348.5-669.5) | 490.6 (351.6-656.3) | -0.05 (-0.12, 0.03) |
| Antigua and Barbuda | 6550.8 (5789.8-7256.7) | 4243.6 (3737.2-4786.6) | -1.38 (-2.14, -0.62) | 441.5 (387.7-489.2) | 290.2 (254.2-328.1) | -1.24 (-1.84, -0.64) |
| Argentina | 6653.8 (5875-7336.2) | 2597.7 (2280.1-2911.4) | -2.96 (-3.47, -2.46) | 426.3 (371.4-470.7) | 149.7 (128.2-168.9) | -3.3 (-3.92, -2.67) |
| Armenia | 9530.5 (8715.9-10219.3) | 6353.3 (5620.6-7080.2) | -1.12 (-2.09, -0.14) | 566.1 (510.6-608.1) | 384.3 (332.8-431.1) | -1.04 (-2.17, 0.11) |
| Australia | 5215.8 (4589.6-5743.6) | 1653.2 (1370.2-1886.7) | -3.71 (-3.93, -3.49) | 359.2 (307.8-398.4) | 104.2 (83.2-119.1) | -3.96 (-4.24, -3.68) |
| Austria | 7441.6 (6651.2-8103.1) | 2013.9 (1703.9-2300.8) | -4.05 (-4.44, -3.66) | 524.2 (459.5-572.8) | 103.6 (83.9-118.1) | -5.03 (-5.74, -4.31) |
| Azerbaijan | 5918.6 (4756.6-7200.1) | 5300.1 (4185.5-6647.1) | -0.34 (-0.77, 0.1) | 336.3 (262.3-418.4) | 300.8 (229.6-385.4) | -0.33 (-0.95, 0.3) |
| Bahamas | 4880.2 (4179.4-5557.2) | 3326.2 (2771-3954.5) | -1.25 (-1.88, -0.63) | 309.9 (263-352.5) | 219.5 (180.5-261.9) | -1.09 (-1.95, -0.23) |
| Bahrain | 11182.6 (9093.8-13329) | 6278.1 (4999.6-7745.3) | -1.94 (-2.54, -1.34) | 747 (605.2-888.9) | 442.7 (351-547.1) | -1.72 (-2.43, -1) |
| Bangladesh | 9452.7 (7013.5-12684.2) | 8261.6 (6124.1-11017.4) | -0.3 (-0.97, 0.38) | 596.1 (438.5-796.2) | 565.9 (419.2-747.7) | -0.03 (-0.81, 0.75) |
| Barbados | 7840.5 (7025.8-8548.4) | 4871.7 (3948.7-5900.1) | -1.58 (-2.37, -0.78) | 562.8 (503.2-612.5) | 343.7 (277-413.9) | -1.52 (-2.4, -0.63) |
| Belarus | 13116.7 (11866.9-14219.8) | 9367.2 (7869.4-10964.4) | -1.12 (-1.56, -0.67) | 772.3 (692-838.5) | 561.6 (465-658.7) | -1.04 (-1.52, -0.55) |
| Belgium | 6311.2 (5548.2-6922.2) | 1921.3 (1605.7-2177.4) | -3.68 (-3.79, -3.58) | 449.4 (387.1-495.6) | 117.3 (92.8-134.2) | -4.08 (-4.24, -3.91) |
| Belize | 3502 (3056-3922.6) | 3003.5 (2557.4-3453.4) | -0.28 (-1.22, 0.67) | 225.7 (194.4-253.9) | 200.2 (168.2-230.2) | -0.19 (-1.09, 0.71) |
| Benin | 10949.8 (8293.3-14053.7) | 9475.4 (7178-12228.2) | -0.45 (-0.63, -0.27) | 661.6 (492.9-858) | 586.2 (436.2-765.5) | -0.39 (-0.55, -0.23) |
| Bermuda | 5799.7 (5077-6492.9) | 2354.6 (1955.9-2846.7) | -2.94 (-3.42, -2.45) | 388.6 (340.2-435.1) | 156 (126-191.1) | -2.98 (-3.6, -2.35) |
| Bhutan | 5768.3 (3614.8-8185.5) | 4758.4 (3456.8-6224.2) | -0.59 (-0.67, -0.52) | 342.1 (200.2-499.6) | 293.2 (208.3-389.8) | -0.48 (-0.57, -0.4) |
| Bolivia (Plurinational State of) | 5873.8 (4025-8008.1) | 3565.3 (2387.3-5098.9) | -1.59 (-1.69, -1.5) | 375.1 (256.3-511) | 234.4 (154.7-335.7) | -1.51 (-1.6, -1.42) |
| Bosnia and Herzegovina | 15436.3 (13165.4-17632.4) | 11048.7 (8805.8-13184.9) | -1.07 (-1.38, -0.76) | 946.3 (799.9-1082.7) | 696.9 (544.7-840.9) | -0.93 (-1.18, -0.68) |
| Botswana | 10919.1 (8208.7-14003.6) | 7735.3 (5985.6-10000) | -1.15 (-2.01, -0.28) | 652.8 (477.7-850.8) | 442.9 (325.8-590.2) | -1.33 (-2.33, -0.32) |
| Brazil | 9452.4 (8667-9954.9) | 3585.7 (3192-3853.8) | -3.01 (-3.33, -2.69) | 611 (547.4-647.9) | 223.8 (192.2-242.6) | -3.13 (-3.32, -2.94) |
| Brunei Darussalam | 10014.8 (8075.5-12228) | 4781.7 (3826.2-5800.8) | -2.46 (-3.07, -1.85) | 587.1 (460.2-729.9) | 298.6 (228.7-371.5) | -2.32 (-2.75, -1.88) |
| Bulgaria | 20298.8 (19006.5-21540) | 16556.8 (14485.1-18718.9) | -0.67 (-1.33, 0) | 1400.6 (1307.7-1483.8) | 1125.7 (987.4-1266.5) | -0.71 (-1.56, 0.14) |
| Burkina Faso | 5907.7 (4103-8157.1) | 5898.5 (4249.4-8031) | 0.01 (-0.11, 0.13) | 356.8 (241.6-500.3) | 362.1 (256.6-496.7) | 0.08 (-0.05, 0.21) |
| Burundi | 10917.5 (7600.8-15005.4) | 6758.6 (4806.3-9376.1) | -1.57 (-1.75, -1.39) | 635.6 (427.7-886.9) | 403.9 (276.9-574.6) | -1.49 (-1.67, -1.31) |
| Cabo Verde | 6169.9 (4735.6-7854.7) | 8015 (6113.6-10192.7) | 0.83 (0.33, 1.33) | 370 (274.6-479) | 499.9 (371.8-644.7) | 1.03 (0.57, 1.5) |
| Cambodia | 9432.6 (7185.9-12061.2) | 8885.3 (6778.7-11294.1) | -0.18 (-0.27, -0.1) | 582.9 (436.2-754.8) | 581 (436-744.5) | 0 (-0.06, 0.07) |
| Cameroon | 8065.4 (5802.8-10819.9) | 8876.2 (6398.3-12235.9) | 0.31 (0.16, 0.46) | 490.7 (345.5-662.8) | 539.4 (383.3-751) | 0.32 (0.2, 0.44) |
| Canada | 4100.2 (3587.8-4514.9) | 1753.6 (1473.2-1996.1) | -2.63 (-2.9, -2.37) | 264.9 (226.6-293.4) | 95.6 (76.4-108.7) | -3.14 (-3.52, -2.76) |
| Central African Republic | 10097.3 (6867-13998.3) | 9069.3 (6060.9-13077.3) | -0.35 (-0.42, -0.27) | 595 (392.1-839.6) | 546 (350.4-804.6) | -0.27 (-0.38, -0.16) |
| Chad | 8692.7 (6162.8-12249.4) | 9920.1 (7215.4-13410) | 0.43 (0.29, 0.57) | 514.7 (353.7-737.1) | 591.3 (424.3-806.5) | 0.46 (0.32, 0.59) |
| Chile | 7062.2 (6375.4-7705) | 2743.1 (2390.1-3071.7) | -3.07 (-3.58, -2.56) | 446.2 (397.4-488.7) | 174 (147.2-195.5) | -3.04 (-3.64, -2.43) |
| China | 9236.2 (7853.2-10837.9) | 8107.3 (6786.9-9364.8) | -0.42 (-0.61, -0.22) | 562 (474.4-663.1) | 491 (401.9-575) | -0.42 (-0.69, -0.15) |
| Colombia | 4716.8 (4215.2-5184.6) | 1841 (1527.2-2174.8) | -3.1 (-3.61, -2.58) | 290.5 (254.5-320.3) | 110.7 (88.9-132.2) | -3.18 (-3.73, -2.62) |
| Comoros | 8741 (6376.7-11537.7) | 6360.2 (4600-8549.5) | -1.05 (-1.11, -0.98) | 505.6 (358.1-682.8) | 372.7 (257.2-513.5) | -0.99 (-1.05, -0.92) |
| Congo | 10846.8 (7937.6-14215.8) | 8943.1 (6635.9-11530.1) | -0.62 (-0.77, -0.47) | 636.7 (453.9-850.7) | 534.3 (383.7-705.3) | -0.56 (-0.71, -0.4) |
| Cook Islands | 5658.4 (4370.7-7219.5) | 3363.8 (2586-4298.4) | -1.67 (-1.73, -1.6) | 334 (248.2-434) | 181.1 (128.1-243.2) | -1.96 (-2.03, -1.89) |
| Costa Rica | 3152 (2774.2-3491.3) | 1895.6 (1601-2156.7) | -1.46 (-2.12, -0.79) | 204.3 (176-227.2) | 123.7 (101.3-142.4) | -1.41 (-2.15, -0.66) |
| Coted'Ivoire | 10590.1 (7948.8-13647.6) | 9686.6 (7119.7-12815.5) | -0.27 (-0.41, -0.14) | 623.2 (462.8-804.1) | 580.3 (423.6-766.8) | -0.22 (-0.32, -0.12) |
| Croatia | 16423.4 (15138.5-17566.5) | 5714.5 (4973-6441.7) | -3.43 (-3.65, -3.21) | 1094.3 (1002.6-1170.7) | 377.7 (322.6-427.8) | -3.45 (-3.68, -3.23) |
| Cuba | 4950.8 (4434.5-5450.9) | 4029.4 (3451.6-4630.7) | -0.69 (-0.8, -0.57) | 331.2 (295.3-364.1) | 260.9 (222-300.2) | -0.83 (-0.96, -0.69) |
| Cyprus | 12160.9 (9631.2-15224.2) | 3112.7 (2372.8-3917.4) | -4.28 (-5.05, -3.5) | 994.6 (781.6-1249) | 265.8 (198.9-338.6) | -4.13 (-4.93, -3.32) |
| Czechia | 18601.7 (17147.8-19867.3) | 3951.5 (3414.5-4449.4) | -4.91 (-5.4, -4.42) | 1220.7 (1113.9-1302.2) | 236.6 (200-268.3) | -5.19 (-5.75, -4.63) |
| Democratic People's Republic of Korea | 9163.6 (6722-12005.4) | 8596.4 (6428.6-11412.1) | -0.2 (-0.26, -0.15) | 509.3 (349.2-686.7) | 466.8 (328.6-641.9) | -0.29 (-0.33, -0.24) |
| Democratic Republic of the Congo | 8073.8 (5716.1-11113.4) | 7288.7 (4761.1-10692.2) | -0.34 (-0.48, -0.21) | 475.7 (324-667.7) | 441.5 (275.9-666.1) | -0.25 (-0.47, -0.03) |
| Denmark | 5831.7 (5183.2-6413.5) | 2254.7 (1915.2-2527) | -3.07 (-3.41, -2.73) | 377 (327.9-415.1) | 147.3 (121.1-166.1) | -3.06 (-3.39, -2.74) |
| Djibouti | 7474 (5273.9-10625.4) | 7162.1 (5191.1-9720.5) | -0.14 (-0.24, -0.04) | 426.9 (286-624.3) | 412.4 (285.6-574.1) | -0.11 (-0.21, 0) |
| Dominica | 7365.7 (5971.1-8889) | 6000 (4694.3-7474.2) | -0.65 (-0.74, -0.56) | 512.9 (413.7-618.9) | 417.5 (324.8-517.4) | -0.66 (-0.73, -0.58) |
| Dominican Republic | 4608.8 (3673-5694.8) | 4168.5 (3088.3-5507.4) | -0.29 (-0.83, 0.24) | 311 (246.5-384.8) | 256.7 (186.1-342.1) | -0.55 (-1.09, -0.02) |
| Ecuador | 4229.8 (3723.6-4685.7) | 2213.5 (1819.7-2647.7) | -2.14 (-2.91, -1.37) | 269.6 (237.5-298.6) | 148.1 (120.7-178) | -1.98 (-2.87, -1.09) |
| Egypt | 20884.2 (15150.2-28132.4) | 16518.3 (12664.9-20931.8) | -0.69 (-1.29, -0.08) | 1375.1 (998.7-1839.4) | 1047.6 (801.6-1323.3) | -0.87 (-1.14, -0.59) |
| El Salvador | 3092.9 (2504.5-3756) | 1917.9 (1466.8-2446) | -1.5 (-2.24, -0.76) | 185.6 (147.4-226.9) | 118.8 (87.8-154.7) | -1.43 (-2.23, -0.62) |
| Equatorial Guinea | 9415.4 (6633.3-13153.9) | 7401.3 (5091.7-10385.5) | -0.71 (-0.88, -0.54) | 543.7 (368.9-780.9) | 441.4 (291-633.9) | -0.6 (-0.78, -0.41) |
| Eritrea | 8205.7 (5469.6-12006) | 7123.9 (5027.1-9713) | -0.46 (-0.53, -0.39) | 468.8 (299.4-698.4) | 425.1 (287.8-591.1) | -0.32 (-0.39, -0.24) |
| Estonia | 16533.7 (15182-17805) | 3407.7 (2901.1-3887) | -4.94 (-6.06, -3.82) | 1040.9 (948.6-1123.4) | 201.3 (167.1-231.7) | -5.19 (-6.07, -4.3) |
| Eswatini | 9402.6 (6904.4-12400) | 9702.3 (6663.9-13618.1) | 0.11 (-0.04, 0.27) | 570.4 (404.8-767.3) | 575.7 (385.7-818.1) | 0.07 (-0.1, 0.23) |
| Ethiopia | 4727.5 (3244.3-6997.9) | 3626.7 (2808.4-4765.4) | -0.86 (-0.95, -0.77) | 263.7 (173.2-399.8) | 210.1 (155.7-285.3) | -0.72 (-0.81, -0.62) |
| Fiji | 6247.3 (4900.5-7875.9) | 5611.8 (4283.3-7184.9) | -0.33 (-0.54, -0.13) | 347.4 (260.4-450.4) | 330 (240.8-436.8) | -0.17 (-0.64, 0.3) |
| Finland | 7233.8 (6398-7925.2) | 2519.4 (2129.9-2845.7) | -3.38 (-3.59, -3.16) | 451.2 (387-498.3) | 148.8 (119.1-170.2) | -3.58 (-3.97, -3.19) |
| France | 4771.5 (4216.6-5224.6) | 1698.8 (1419.9-1929.7) | -3.28 (-3.54, -3.01) | 349.1 (302.7-383.2) | 103.6 (82.7-117.8) | -3.83 (-4.16, -3.5) |
| Gabon | 8424.9 (6192.6-11107.9) | 7570.8 (5520.9-9933.2) | -0.32 (-0.49, -0.14) | 488.2 (341-660.6) | 450.1 (313.3-607.5) | -0.22 (-0.41, -0.04) |
| Gambia | 10274.2 (7416.9-13760) | 11295 (7993.6-15307.8) | 0.38 (0.03, 0.74) | 600.5 (424.5-815.1) | 685.5 (476.7-939.1) | 0.5 (0.21, 0.8) |
| Georgia | 9461.8 (7768.6-10896.2) | 11493.6 (10015.4-12834.1) | 0.69 (-0.65, 2.04) | 597.3 (493.3-687.8) | 731.6 (630.9-821.2) | 0.79 (-0.83, 2.44) |
| Germany | 7824.5 (6897.3-8588.1) | 2471.1 (2098.5-2797.1) | -3.64 (-4.01, -3.27) | 524.4 (451.3-578.5) | 133.6 (106.6-153) | -4.3 (-4.56, -4.04) |
| Ghana | 12193.6 (9227.7-15872.4) | 12717.2 (9511.8-16627.1) | 0.14 (0.08, 0.2) | 721.9 (537.1-946.4) | 764.3 (562.5-1012.5) | 0.18 (0.12, 0.25) |
| Greece | 10782.5 (9642.9-11621) | 3216.9 (2725.9-3586.4) | -3.96 (-4.3, -3.62) | 815.9 (720-880.2) | 232.4 (190.9-259.8) | -4.13 (-4.44, -3.82) |
| Greenland | 12006.1 (9861.3-14378.2) | 4326.6 (3391.1-5460.5) | -3.15 (-3.41, -2.9) | 790.9 (638.8-956.6) | 279.7 (212.2-360.9) | -3.19 (-3.47, -2.92) |
| Grenada | 10914.8 (9518.8-12237.9) | 5916.8 (5084.9-6680.4) | -2.23 (-2.75, -1.7) | 683 (595.6-763.9) | 401.6 (342.1-454.2) | -1.98 (-2.54, -1.42) |
| Guam | 5665.2 (4645.1-6779.4) | 2332.8 (1892.2-2824) | -3.03 (-3.77, -2.28) | 335.3 (265.8-410.9) | 85.1 (62.8-109.2) | -4.65 (-5.95, -3.33) |
| Guatemala | 3530.1 (3065.9-3986.1) | 2089.2 (1769-2421.5) | -1.59 (-2.42, -0.75) | 239.4 (207.8-269.7) | 144.1 (120.7-167.2) | -1.45 (-2.46, -0.43) |
| Guinea | 8886.6 (6148.8-12001.2) | 10002.1 (7255.7-13474.6) | 0.4 (0.31, 0.49) | 531.6 (355.8-725.5) | 599.7 (428.5-814.2) | 0.41 (0.34, 0.48) |
| Guinea-Bissau | 13759.6 (9908.6-18567.4) | 13129.4 (9698.7-17266.2) | -0.15 (-0.24, -0.05) | 788.9 (563.8-1063.4) | 787.3 (569.4-1043.6) | -0.01 (-0.09, 0.07) |
| Guyana | 14313.3 (12656.3-16107.8) | 8392.4 (6612.8-10351.6) | -1.65 (-2.23, -1.06) | 850.8 (752.6-956.3) | 542.2 (426.4-668.8) | -1.33 (-2, -0.66) |
| Haiti | 12887.8 (9423.4-16511.3) | 9876.6 (6877.4-13826.8) | -0.86 (-0.9, -0.81) | 840.8 (615.1-1072.2) | 649 (453.5-902.6) | -0.83 (-0.87, -0.78) |
| Honduras | 4811.4 (3568.3-6249.9) | 6332 (4509.2-8451.6) | 0.96 (0.66, 1.27) | 309.6 (226.7-404.6) | 421.8 (300.5-563.3) | 1.08 (0.74, 1.42) |
| Hungary | 15879.7 (14626.5-17038.7) | 5396.5 (4590.2-6165.7) | -3.4 (-3.92, -2.88) | 1004.5 (916.6-1079.6) | 319.8 (266.8-370.3) | -3.55 (-4.07, -3.02) |
| Iceland | 4834.2 (4236.9-5354.7) | 1700.4 (1404-1958.1) | -3.35 (-4.02, -2.66) | 307.5 (263.8-342.2) | 105.5 (81.8-122.6) | -3.35 (-4.13, -2.57) |
| India | 4898.7 (3984-6158.3) | 4184.5 (3560.3-5251.3) | -0.45 (-0.9, 0) | 281.7 (226.8-355) | 247.6 (209-307.9) | -0.44 (-1.06, 0.18) |
| Indonesia | 9186.9 (7371.6-11009) | 11982.5 (9250.3-14704.3) | 0.86 (0.81, 0.91) | 542 (424.6-663.5) | 751.8 (576.5-927.9) | 1.06 (1.01, 1.11) |
| Iran (Islamic Republic of) | 11536 (10241-12656.4) | 6198.2 (5492.2-6829.8) | -2 (-2.14, -1.86) | 734.2 (641.6-808.2) | 396.3 (343.3-439.6) | -1.99 (-2.12, -1.87) |
| Iraq | 15397.9 (12258.2-18769.8) | 14502.3 (11128.7-18072) | -0.12 (-0.34, 0.1) | 902.8 (711.3-1103.9) | 922.8 (713.3-1144) | 0.16 (-0.09, 0.42) |
| Ireland | 7050.3 (6315.9-7683.5) | 1609.9 (1315.1-1837.7) | -4.68 (-5.01, -4.35) | 488.1 (429.3-534) | 113.7 (89.3-130.6) | -4.62 (-4.99, -4.25) |
| Israel | 4479.3 (3941.7-4952) | 1535.4 (1288.5-1763.4) | -3.47 (-4.35, -2.58) | 279.5 (241.3-311.2) | 85.1 (68.5-98) | -3.97 (-4.83, -3.1) |
| Italy | 7093.2 (6277.1-7577.3) | 2061.7 (1703.7-2303.1) | -3.9 (-4.24, -3.56) | 516.9 (444.4-554.7) | 151.2 (118.9-169.4) | -3.88 (-4.28, -3.48) |
| Jamaica | 7555 (6682-8357.7) | 5337.8 (4214.4-6618.3) | -0.89 (-1.49, -0.28) | 512.2 (450.2-565.5) | 365.9 (287.6-453.9) | -0.9 (-1.47, -0.33) |
| Japan | 6511.1 (5755.3-7028.3) | 2031.9 (1693.7-2324.1) | -3.74 (-4.1, -3.37) | 439.6 (376.4-472.5) | 109.9 (86.9-123.7) | -4.38 (-4.84, -3.92) |
| Jordan | 13339.9 (10721.2-16299.7) | 6721.2 (5341.9-8233.1) | -2.34 (-2.79, -1.89) | 818.9 (650.4-1005.5) | 411.6 (319-512.8) | -2.34 (-3.06, -1.61) |
| Kazakhstan | 14981.1 (13199.7-16528.9) | 12052.7 (10539.2-13571.4) | -0.61 (-1.25, 0.03) | 886.5 (776.3-977.9) | 771.8 (672.6-867.8) | -0.34 (-1.16, 0.49) |
| Kenya | 4906.7 (3763.5-6302.8) | 5389.2 (4156.5-6766.1) | 0.3 (0.25, 0.35) | 285.2 (205.2-378.6) | 323.8 (234.9-421.2) | 0.41 (0.33, 0.5) |
| Kiribati | 7011.5 (5214.6-9006.6) | 7062.3 (5309-9061.5) | 0.02 (-0.01, 0.06) | 380.6 (263.1-507.3) | 406.7 (295.4-536.3) | 0.22 (0.18, 0.26) |
| Kuwait | 4668 (4025.6-5262.9) | 3272.9 (2667.8-3926.2) | -1.12 (-2.99, 0.78) | 263.4 (219.5-299.8) | 192.7 (151.5-238.1) | -0.86 (-2.99, 1.32) |
| Kyrgyzstan | 14021.5 (12447.1-15481.8) | 7849.6 (6622.5-9115.2) | -1.84 (-2.43, -1.26) | 850.7 (746.9-942.5) | 424 (350.5-495.9) | -2.22 (-2.65, -1.8) |
| Lao People's Democratic Republic | 13474.1 (10162.5-17427.4) | 9645.9 (7273.9-12582.4) | -1.08 (-1.14, -1.03) | 796.7 (592.5-1040.6) | 592.7 (439.5-779.3) | -0.96 (-1.01, -0.9) |
| Latvia | 17475.9 (16079.6-18736.6) | 10350.8 (8985.9-11552.2) | -1.45 (-2.39, -0.49) | 1130.4 (1029.8-1213.9) | 676.2 (576.6-756.4) | -1.38 (-2.36, -0.38) |
| Lebanon | 8751.3 (6527.7-11583) | 3419.5 (2682.2-4263.5) | -2.97 (-3.19, -2.76) | 561.8 (413.7-749.5) | 214.2 (162.1-273.7) | -3.05 (-3.3, -2.81) |
| Lesotho | 7343.3 (5301.3-9995.1) | 11529.4 (8189.1-15838.4) | 1.57 (1.17, 1.97) | 449.8 (311-630.1) | 693.6 (482.2-953.5) | 1.49 (1.03, 1.94) |
| Liberia | 9707.4 (7467.1-12439.7) | 9223.4 (6684.4-12501.3) | -0.15 (-0.33, 0.02) | 590.8 (447.2-758.9) | 569 (407-775.9) | -0.11 (-0.28, 0.06) |
| Libya | 6335.7 (4567.4-8684.9) | 6875.4 (4894.2-9426.1) | 0.31 (-0.14, 0.77) | 386.6 (268.4-535.7) | 418.4 (290.2-580.9) | 0.29 (-0.22, 0.8) |
| Lithuania | 9640.7 (8766.8-10497.1) | 6565 (5667.2-7420.7) | -1.17 (-1.69, -0.65) | 568.6 (513-618.2) | 411.1 (350.1-464.2) | -0.98 (-1.45, -0.51) |
| Luxembourg | 9970 (9068.7-10716) | 1806.4 (1536.3-2037.5) | -5.4 (-6, -4.79) | 731.2 (660.6-786.7) | 123.9 (101.7-140.7) | -5.56 (-6.25, -4.87) |
| Madagascar | 9915.3 (7509.9-12650.3) | 9239.8 (6471.9-12396.9) | -0.22 (-0.34, -0.1) | 581.9 (427.7-754.8) | 545.8 (368.6-748.8) | -0.19 (-0.31, -0.07) |
| Malawi | 7763.7 (5796-10039.1) | 8485.2 (6254-11251.8) | 0.31 (0.19, 0.44) | 457.7 (328.7-603) | 510.8 (363.4-688.1) | 0.41 (0.2, 0.61) |
| Malaysia | 7352.2 (5925.5-8960.3) | 5670.1 (4539.9-6892.8) | -0.73 (-0.86, -0.6) | 392.2 (303.7-486) | 320.4 (246.7-403.4) | -0.61 (-0.97, -0.26) |
| Maldives | 10128.4 (7918.7-12600.8) | 4865.7 (3838.2-5963.3) | -2.51 (-2.68, -2.34) | 594.3 (446.4-749.9) | 310.7 (237.4-385.8) | -2.26 (-2.43, -2.09) |
| Mali | 7289.7 (5106.2-10364.9) | 6846.7 (4824-9657.5) | -0.17 (-0.34, 0) | 440.9 (299.3-635.1) | 416.4 (288-591.5) | -0.16 (-0.36, 0.04) |
| Malta | 7098.9 (6325.9-7785) | 1733.5 (1437.5-1985.8) | -4.58 (-5.08, -4.09) | 475.6 (415.5-523.7) | 118.7 (95-137.6) | -4.8 (-5.21, -4.39) |
| Marshall Islands | 8029.2 (5900.1-10716.5) | 6843.6 (5011.3-9224.8) | -0.53 (-0.63, -0.43) | 477.4 (338.6-646.2) | 409.4 (290.7-564.5) | -0.51 (-0.62, -0.4) |
| Mauritania | 11274 (8064.9-15382.3) | 8922.5 (6321.3-12651.5) | -0.76 (-0.9, -0.62) | 668.4 (464.6-924.4) | 553.1 (382-792) | -0.6 (-0.76, -0.45) |
| Mauritius | 13126 (11868.6-14310) | 4634.6 (4111.3-5159.4) | -3.26 (-4.09, -2.43) | 746.4 (665.1-816.9) | 266.5 (231.6-299) | -3.25 (-4.29, -2.2) |
| Mexico | 4598.8 (4352.7-4800) | 2155 (1916.4-2409.8) | -2.51 (-3.02, -2) | 316.1 (297.2-328.5) | 134.1 (117.7-149.2) | -2.8 (-3.43, -2.17) |
| Micronesia (Federated States of) | 8400.5 (6059.5-11221.5) | 6982.4 (5085.2-9473.4) | -0.59 (-0.62, -0.57) | 493.1 (340.4-671) | 411.8 (291.2-572.7) | -0.58 (-0.61, -0.54) |
| Monaco | 8537.9 (6422.1-10545.4) | 3511.2 (2659.1-4450.1) | -2.83 (-2.94, -2.73) | 606.8 (440.7-755.5) | 245.3 (176-318.7) | -2.92 (-3.05, -2.79) |
| Mongolia | 3316.1 (2486.9-4286.6) | 3368.6 (2480.4-4357.3) | -0.1 (-0.44, 0.24) | 173.4 (123.3-236) | 175.4 (119.6-237.6) | -0.03 (-0.81, 0.76) |
| Montenegro | 4733.9 (3681.6-5948.6) | 6994.9 (5371.1-8899.9) | 1.45 (1, 1.9) | 302.1 (225.1-384.8) | 506 (381.6-652.4) | 1.82 (1, 2.64) |
| Morocco | 11434.6 (8299.7-15187.9) | 10925.4 (8151.2-14063.1) | -0.16 (-0.28, -0.04) | 707.7 (507.7-946.9) | 693.3 (514.9-897) | -0.07 (-0.18, 0.04) |
| Mozambique | 9697.7 (7302.3-12645.3) | 11845.6 (8284.5-15878.1) | 0.68 (0.61, 0.76) | 558.9 (405.5-738.8) | 673.8 (457.8-919.6) | 0.66 (0.56, 0.77) |
| Myanmar | 12527.4 (9446-16155.2) | 9056.4 (6838.4-11876.3) | -1.05 (-1.1, -1) | 739.6 (554.3-960.8) | 558.4 (414.7-744.8) | -0.91 (-0.96, -0.86) |
| Namibia | 11441.4 (8799.1-14352.3) | 10199.4 (7651.4-13102.8) | -0.31 (-0.55, -0.06) | 688 (516.2-875.9) | 625.1 (454.7-812.7) | -0.25 (-0.46, -0.05) |
| Nauru | 11363.1 (8282.7-14869.3) | 10034.6 (7399.1-13163.3) | -0.41 (-0.46, -0.35) | 653 (460.4-867.5) | 581.7 (415.7-776.1) | -0.38 (-0.43, -0.32) |
| Nepal | 6876.1 (4669.1-9450.2) | 5126.3 (3638-7229.8) | -0.95 (-1, -0.89) | 411.9 (275.9-566.8) | 321.4 (225.6-454.5) | -0.8 (-0.88, -0.72) |
| Netherlands | 5612.7 (4922.5-6201.1) | 2385.7 (2001.6-2695.7) | -2.79 (-3.19, -2.39) | 367.8 (313.6-407.9) | 162.1 (130.7-184) | -2.71 (-3.14, -2.29) |
| New Zealand | 5287.3 (4634.9-5806.1) | 2142.6 (1791.7-2432.5) | -2.91 (-3.12, -2.7) | 354.4 (302.3-391.3) | 148.3 (119.4-167.9) | -2.82 (-3.08, -2.55) |
| Nicaragua | 3559 (2861.5-4309.5) | 2112.7 (1628.1-2688.9) | -1.77 (-2.2, -1.35) | 224.7 (176.3-274.9) | 130.3 (96.7-169.6) | -1.8 (-2.19, -1.4) |
| Niger | 7521.1 (4983.7-11199.1) | 7296.4 (4948.9-10693.9) | -0.08 (-0.17, 0.01) | 448.7 (282-683.7) | 455.4 (302.5-676.8) | 0.06 (-0.04, 0.16) |
| Nigeria | 9781 (7146.6-13292.2) | 7524.9 (6019.4-9315.9) | -0.85 (-0.97, -0.73) | 593.7 (424.8-808.3) | 459.8 (364.6-572.3) | -0.83 (-0.96, -0.69) |
| Niue | 7572.4 (5907.6-9686.4) | 6172.6 (4779.1-7809.4) | -0.65 (-0.72, -0.59) | 445.7 (332.5-584.1) | 362.4 (269-471.5) | -0.66 (-0.74, -0.57) |
| North Macedonia | 23690 (20781.3-26627) | 22893 (19025.2-26976.2) | -0.06 (-0.54, 0.41) | 1505.4 (1309.3-1694.1) | 1704.8 (1429.8-1997.9) | 0.52 (-0.1, 1.15) |
| Northern Mariana Islands | 6382.1 (4996.7-8046.3) | 4455.7 (3491.3-5498.3) | -1.2 (-1.57, -0.82) | 384.9 (291.2-496.1) | 259.6 (196.1-330.9) | -1.37 (-2.11, -0.61) |
| Norway | 6684.8 (6022.7-7212.9) | 1991.4 (1686.7-2269.4) | -3.86 (-4.16, -3.55) | 434.7 (382.6-464.6) | 113.1 (93.1-124.8) | -4.26 (-4.63, -3.88) |
| Oman | 10098.9 (7338.3-13463.3) | 6870.1 (5271.4-8699.2) | -1.22 (-2.06, -0.38) | 573.2 (410.6-766.7) | 423.1 (319-542.3) | -0.91 (-1.74, -0.07) |
| Pakistan | 6080.6 (4438.1-8097.8) | 6200.4 (4789.4-8038.8) | 0.09 (0.01, 0.16) | 360.1 (254.9-486.6) | 368.3 (278.2-479.7) | 0.1 (0.03, 0.17) |
| Palau | 8518.7 (6549.6-10873.5) | 7225.8 (5544.3-9223.1) | -0.57 (-0.66, -0.48) | 494.6 (364.8-644.9) | 428.6 (319.6-560.5) | -0.52 (-0.65, -0.38) |
| Palestine | 16083.5 (12522.3-20156.4) | 9951.1 (8057-12094.9) | -1.58 (-1.87, -1.29) | 1090.4 (852.8-1355.4) | 683.6 (550-831.8) | -1.53 (-1.67, -1.39) |
| Panama | 4330.3 (3762.3-4825.7) | 2657.8 (2080.5-3185.4) | -1.46 (-2.05, -0.86) | 283.3 (242.8-316.3) | 177.9 (134.8-215.3) | -1.4 (-2.1, -0.69) |
| Papua New Guinea | 5976.9 (4062.8-8600.9) | 5305.7 (3770.8-7500.5) | -0.39 (-0.43, -0.36) | 358.5 (234.3-527.8) | 322.5 (219.8-469.2) | -0.35 (-0.38, -0.31) |
| Paraguay | 6657.3 (5405.7-7914) | 4579.6 (3413.5-5855) | -1.09 (-1.28, -0.89) | 445 (356.5-529.1) | 299.5 (218.6-384.5) | -1.17 (-1.75, -0.6) |
| Peru | 3098 (2416.1-3871.3) | 1820.4 (1343.8-2401.4) | -1.57 (-2.71, -0.42) | 196.7 (151.2-248.5) | 111.2 (78.7-151.5) | -1.68 (-3.15, -0.2) |
| Philippines | 6516.1 (5698.2-7266.3) | 5944.8 (5126.9-6783.5) | -0.28 (-0.62, 0.06) | 426.8 (371.1-477.3) | 343.2 (292.1-394.1) | -0.71 (-1.12, -0.3) |
| Poland | 14242.1 (13322.6-14874.3) | 5164.6 (4554.1-5693.2) | -3.29 (-3.67, -2.9) | 960 (885.1-1004) | 332.6 (286.4-366.8) | -3.5 (-4.08, -2.92) |
| Portugal | 17356 (15770.4-18541.2) | 2932.9 (2476.4-3278.1) | -5.6 (-5.89, -5.32) | 1286.1 (1153.4-1372.5) | 219.5 (178.9-247.2) | -5.63 (-6.18, -5.08) |
| Puerto Rico | 3344.6 (3000.7-3675.8) | 1184.6 (974.2-1385.7) | -3.47 (-4.01, -2.92) | 242 (215.3-266) | 71.3 (56-85.1) | -3.94 (-4.82, -3.05) |
| Qatar | 10453.8 (8027.6-13233.2) | 4125.6 (3057.9-5338.9) | -2.95 (-4.2, -1.68) | 674.8 (510.1-864.3) | 272.9 (197.3-360) | -2.9 (-4.34, -1.44) |
| Republic of Korea | 14335.9 (12090.4-16599.8) | 3262.1 (2634.2-3889) | -4.73 (-5.05, -4.41) | 906.4 (747.4-1064.6) | 200.3 (151.3-246.1) | -4.9 (-5.34, -4.47) |
| Republic of Moldova | 10086.7 (8873.4-11196) | 7137 (6307.9-8002.1) | -1.13 (-2.1, -0.15) | 673 (590.9-744.5) | 389.2 (337.8-438.8) | -1.81 (-2.85, -0.76) |
| Romania | 18115.1 (16394.8-19596.8) | 10537.2 (9201.2-11800.8) | -1.82 (-2.28, -1.36) | 1242.8 (1126.3-1340) | 695.3 (599.4-782.8) | -2.03 (-2.49, -1.56) |
| Russian Federation | 21375.6 (20190.1-22060.3) | 11643.5 (10549-12562.5) | -1.93 (-2.58, -1.28) | 1411.3 (1314.3-1457.4) | 750.4 (668.3-810.9) | -1.96 (-2.69, -1.23) |
| Rwanda | 11122.2 (8085.5-14868.4) | 6184.1 (4224.5-8544.4) | -1.9 (-2.06, -1.75) | 655.6 (463.6-892.5) | 376.5 (243.7-535.8) | -1.79 (-1.94, -1.64) |
| Saint Kitts and Nevis | 15033.6 (13296.6-16641.3) | 8237.3 (6959.7-9420.1) | -1.89 (-2.84, -0.94) | 1039.7 (925-1144.9) | 566.8 (480.6-642.2) | -1.86 (-2.75, -0.97) |
| Saint Lucia | 12513.4 (11323.7-13661.6) | 5577.5 (4616.6-6593.6) | -2.72 (-3.58, -1.87) | 927.6 (842.4-1007.8) | 409.7 (338.7-483.6) | -2.92 (-3.38, -2.45) |
| Saint Vincent and the Grenadines | 8668 (7664.5-9664.9) | 5287.9 (4603.9-5992.8) | -1.52 (-2.32, -0.71) | 601.5 (530.2-669) | 378.8 (328.9-428.6) | -1.37 (-1.97, -0.77) |
| Samoa | 6830.4 (5198.1-8796.1) | 5705.8 (4424.1-7232.1) | -0.57 (-0.61, -0.53) | 402.3 (290.1-531.1) | 331 (244-434.9) | -0.62 (-0.67, -0.57) |
| San Marino | 5471.3 (4322.2-6659.2) | 1892.8 (1354.8-2555.9) | -3.58 (-4.03, -3.14) | 384.1 (294.9-475.8) | 115.2 (73.2-165.3) | -4.11 (-4.8, -3.41) |
| Sao Tome and Principe | 8555.5 (6652.9-10727.5) | 9617.5 (7586.5-12150.2) | 0.38 (0.19, 0.58) | 509.9 (385.7-651.9) | 578 (448.2-738.5) | 0.4 (0.21, 0.6) |
| Saudi Arabia | 11619.8 (8648.2-14984.2) | 7759.9 (6046.7-9726.6) | -1.33 (-1.46, -1.2) | 738.8 (546.2-952.7) | 499.1 (384.2-629.8) | -1.29 (-1.44, -1.15) |
| Senegal | 10659.2 (8070.7-13769.5) | 9821.6 (7235.7-13240.8) | -0.25 (-0.46, -0.04) | 628.8 (466.6-821.6) | 603.7 (437.3-823.4) | -0.11 (-0.32, 0.1) |
| Serbia | 26408.6 (22884.8-29960.5) | 14419.2 (11859-17177.4) | -1.9 (-2.16, -1.64) | 1843.9 (1598.6-2089.3) | 988.4 (810.7-1180.8) | -1.96 (-2.21, -1.7) |
| Seychelles | 7974.8 (6354-9662.6) | 5447.3 (4283-6638.5) | -1.21 (-1.76, -0.65) | 465.2 (362.7-568.8) | 326.8 (249.3-405.1) | -1.11 (-1.7, -0.51) |
| Sierra Leone | 10997.7 (8278-14335.1) | 10155.1 (7361.7-13580.7) | -0.27 (-0.44, -0.09) | 652.8 (484.1-856.2) | 610 (437.5-816) | -0.23 (-0.43, -0.02) |
| Singapore | 7996.7 (7207.4-8728.4) | 1317 (1092.5-1524.1) | -5.7 (-6.83, -4.57) | 430.5 (378.9-472.4) | 54.4 (43.4-63.2) | -6.53 (-7.59, -5.46) |
| Slovakia | 12741.6 (10892.1-14650.5) | 6507.2 (5278.9-7773.6) | -2.16 (-2.51, -1.8) | 749.4 (628-870.2) | 375.2 (295.8-459) | -2.21 (-2.64, -1.78) |
| Slovenia | 10250.1 (9265.4-11123.2) | 3007.7 (2547.6-3444.9) | -3.94 (-4.58, -3.29) | 647.4 (576.8-705.6) | 198.6 (163.3-228) | -3.92 (-4.73, -3.09) |
| Solomon Islands | 7634.2 (5559.5-10519.1) | 7117.8 (5101.9-9894.2) | -0.2 (-0.31, -0.09) | 445.1 (308.6-633.6) | 418 (286.6-600.5) | -0.19 (-0.28, -0.09) |
| Somalia | 6952.7 (4377.9-10562.9) | 5714.3 (3589.6-8650.1) | -0.61 (-0.7, -0.51) | 399.9 (240.9-618.9) | 326 (191.6-509) | -0.62 (-0.73, -0.52) |
| South Africa | 5799.8 (4614.8-6734.5) | 7241.8 (6403.9-8088.5) | 0.72 (0.12, 1.32) | 333.5 (254.5-393.2) | 445.1 (386.5-499.9) | 0.95 (0.32, 1.59) |
| South Sudan | 6970.2 (4884.5-9619.5) | 5866.5 (4094.5-8239.2) | -0.55 (-0.63, -0.46) | 403.1 (272.5-569.3) | 339.5 (228.3-484) | -0.54 (-0.64, -0.44) |
| Spain | 7468.6 (6579-8119.3) | 1792 (1495.1-2038.5) | -4.45 (-4.6, -4.3) | 550.3 (476-599.1) | 107.5 (85.3-122.9) | -5.06 (-5.24, -4.89) |
| Sri Lanka | 11628.9 (9905.5-13429.1) | 7957.6 (5745.4-10384.6) | -1.22 (-1.73, -0.71) | 803.2 (683.3-926.8) | 548.4 (387.7-722.6) | -1.23 (-1.79, -0.67) |
| Sudan | 12394.5 (8671.7-16504.3) | 9679.2 (6822.6-13013.4) | -0.8 (-0.86, -0.74) | 761.3 (525.2-1014.3) | 598.1 (416.4-803.5) | -0.78 (-0.84, -0.72) |
| Suriname | 6900.5 (5616.1-8284.8) | 4991 (3640.4-6593.6) | -0.89 (-1.46, -0.31) | 434.3 (349.8-523.4) | 317.6 (225.6-424.5) | -0.56 (-1.28, 0.15) |
| Sweden | 5483.5 (4851.1-5994.8) | 1991.8 (1660.3-2303.1) | -3.33 (-3.76, -2.9) | 366.2 (315.9-400.8) | 123.5 (99.2-142) | -3.55 (-4.07, -3.04) |
| Switzerland | 4681.9 (4055.8-5159.7) | 1432.8 (1185.3-1636.1) | -3.76 (-3.91, -3.61) | 335.3 (283.7-371.2) | 90.4 (70.5-104) | -4.2 (-4.35, -4.06) |
| Syrian Arab Republic | 9560.3 (7435.7-11954) | 7691.6 (5816.3-9930.9) | -0.67 (-0.92, -0.42) | 607 (469.3-761.9) | 506.6 (382.3-650.1) | -0.55 (-0.82, -0.28) |
| Taiwan (Province of China) | 7981.6 (7190.1-8743.4) | 2444.8 (2075.1-2800.6) | -3.72 (-4.23, -3.2) | 439.8 (389.7-482) | 108.2 (89.4-124.1) | -4.37 (-5.23, -3.49) |
| Tajikistan | 10532.5 (8242-12757) | 9166.1 (7257.4-11344.7) | -0.49 (-0.91, -0.07) | 614.5 (465.4-753.2) | 548.8 (427.1-685.3) | -0.38 (-0.81, 0.06) |
| Thailand | 5929 (4542.2-7472) | 3598.1 (2715.3-4615) | -1.6 (-1.96, -1.23) | 332.6 (241-430.6) | 195.4 (136.6-260.6) | -1.71 (-2.17, -1.25) |
| Timor-Leste | 7866 (5982.8-9916.1) | 8533.6 (6026.9-11421.7) | 0.27 (0.15, 0.39) | 484.7 (362.6-615.2) | 536.1 (372.6-727.1) | 0.34 (0.21, 0.47) |
| Togo | 10509 (7911.2-13445.7) | 10328.7 (7402.8-13811.3) | -0.06 (-0.15, 0.03) | 623.2 (461.4-810.7) | 622.7 (436.6-842.6) | -0.02 (-0.12, 0.08) |
| Tokelau | 7819.4 (5747.1-10263.3) | 5473 (4037.2-7181.5) | -1.15 (-1.18, -1.12) | 475.4 (335.9-635.4) | 325.8 (227.4-439.8) | -1.21 (-1.24, -1.19) |
| Tonga | 4327.5 (3332.1-5584.3) | 4016.5 (3034.9-5164.4) | -0.22 (-0.51, 0.08) | 242.8 (174.4-326.4) | 231.6 (163.5-310.9) | -0.12 (-0.37, 0.14) |
| Trinidad and Tobago | 9668.6 (8767-10525.6) | 4926.2 (3880-6088.9) | -2.05 (-2.39, -1.71) | 654.8 (592.2-711.4) | 326.1 (253.5-403.1) | -2.25 (-2.48, -2.02) |
| Tunisia | 9247.7 (6963.8-12012.2) | 6923.5 (4727.2-9566.3) | -1 (-1.12, -0.87) | 612.9 (455-802) | 456.2 (302.1-635.7) | -1.03 (-1.36, -0.71) |
| Turkey | 9869.2 (7783.7-12189.1) | 5443.3 (4240.2-6812.9) | -1.87 (-2.19, -1.54) | 626.4 (486.3-781.5) | 364.5 (275.6-461.8) | -1.62 (-2.03, -1.21) |
| Turkmenistan | 9993.2 (8452.5-11387.4) | 12277.4 (9692.3-15144.7) | 0.8 (-0.01, 1.62) | 544.4 (456.4-620.9) | 653.6 (508.7-810.8) | 0.77 (-0.14, 1.7) |
| Tuvalu | 8889.2 (6700.3-11592) | 6989.1 (5308.6-8990.6) | -0.78 (-0.82, -0.74) | 530.9 (388-703.6) | 419.6 (308.5-554.8) | -0.76 (-0.79, -0.73) |
| Uganda | 6853.2 (4904.6-9258.9) | 5330.1 (3914.8-7215.3) | -0.81 (-0.9, -0.72) | 398.1 (268.1-554.8) | 303.3 (209.9-425.1) | -0.86 (-0.98, -0.75) |
| Ukraine | 18106.3 (16617.7-19300.8) | 9696.1 (7550.1-12126.4) | -1.98 (-2.4, -1.55) | 1179.5 (1075.2-1257.2) | 602.7 (461.7-764.6) | -2.12 (-2.78, -1.46) |
| United Arab Emirates | 11736.2 (8905.4-15307.1) | 7955.4 (6126-10077.9) | -1.26 (-3.77, 1.32) | 670.2 (499.4-880.7) | 489 (366.9-630.6) | -1.02 (-4.17, 2.23) |
| United Kingdom | 7059.5 (6478.1-7438.5) | 1885.2 (1629.6-2086.8) | -4.21 (-4.58, -3.84) | 478.8 (430.3-503.4) | 119.4 (100.4-129.7) | -4.4 (-4.86, -3.94) |
| United Republic of Tanzania | 5548.1 (4035.4-7573.8) | 6923.5 (4978.4-9361.8) | 0.7 (0.51, 0.9) | 321.1 (221.8-456.4) | 409.3 (282-565.3) | 0.79 (0.6, 0.97) |
| United States of America | 3731.5 (3293.1-4083.2) | 2333.6 (1991.2-2613.9) | -1.43 (-1.58, -1.27) | 225.9 (192.7-243.4) | 133.9 (108.6-147.2) | -1.63 (-1.95, -1.3) |
| United States Virgin Islands | 4897.2 (3758.4-6225.5) | 2219.7 (1655.3-2879.4) | -2.59 (-2.92, -2.27) | 339.2 (259-433.1) | 151.3 (110.3-198.9) | -2.65 (-2.94, -2.36) |
| Uruguay | 8841.3 (7913.6-9654.4) | 3971 (3465.1-4402.2) | -2.61 (-2.89, -2.32) | 576.6 (508.5-629.6) | 256.1 (217.3-285.6) | -2.72 (-3.1, -2.34) |
| Uzbekistan | 8115.6 (7281.6-8888.6) | 8220.8 (7097.1-9494.3) | 0.02 (-0.72, 0.77) | 425.5 (373.4-470.8) | 455.2 (385.7-529.1) | 0.22 (-0.65, 1.09) |
| Vanuatu | 8662.5 (6447.9-11376.3) | 7120.6 (5303.3-9184) | -0.64 (-0.74, -0.54) | 504 (358.9-675.4) | 411 (289.7-547) | -0.66 (-0.74, -0.58) |
| Venezuela (Bolivarian Republic of) | 3958.9 (3387.6-4471) | 3224 (2484.3-4091.3) | -0.64 (-0.94, -0.34) | 254.3 (214.5-287.8) | 214.1 (160.6-273.4) | -0.47 (-0.78, -0.16) |
| Viet Nam | 10877.8 (8361.6-14113.5) | 11486.8 (8759.4-14363) | 0.19 (0.12, 0.26) | 689.3 (517.4-908.7) | 734 (555-920.7) | 0.22 (0.15, 0.3) |
| Yemen | 13833.3 (9638.5-19139.1) | 12724.6 (8866.7-17619.1) | -0.26 (-0.34, -0.17) | 844.5 (585.3-1164.8) | 799.1 (552.3-1111.9) | -0.17 (-0.26, -0.08) |
| Zambia | 7152.1 (5076.7-10129.9) | 8344.6 (5992.2-11293.8) | 0.51 (0.43, 0.59) | 426.1 (289.5-617.2) | 501 (347.9-692.2) | 0.53 (0.41, 0.64) |
| Zimbabwe | 6725.5 (5158.2-8429.5) | 9193.3 (6966.8-11861.5) | 1.1 (0.46, 1.74) | 411.7 (306.3-526.2) | 545.1 (404.5-709.8) | 1.16 (0.7, 1.61) |

ASDR = Age-standardized DALYs rate; ASMR = Age-standardized mortality rate; DALYs =Disability-Adjusted Life Years;

AAPC = average annual percentage change; CI=confidence interval; SDI=sociodemographic index; UI=uncertainty interval.
