# Supplementary material for: Global burden of ischemic stroke in adults aged 60 years and older from 1990 to 2021: Population-based study
Source: PLoS One. 2025 May 5;20(5):e0322606. doi: 10.1371/journal.pone.0322606 (PMC12052125; doi:10.1371/journal.pone.0322606)
Supplement: S4 Table — (DOCX) [file pone.0322606.s015.docx]

Table 4. ASPR, ASIP, ASDR and ASMR of Ischemic stroke in 2021 by sex, global, regional, and national, 2021

|  | Male | | | | Female | | | |
| --- | --- | --- | --- | --- | --- | --- | --- | --- |
|  | Prevalence | Incidence | DALYs | Deaths | Prevalence | Incidence | DALYs | Deaths |
| Global | 4791.95 (4280.19-5363.09) | 606.09 (447.05-797.26) | 6541.94 (5886.73-7192.18) | 384.3 (341.59-425.16) | 3795.06 (3352.74-4290.13) | 480.38 (354.85-633.22) | 4830.91 (4217.17-5360.44) | 293.89 (250.25-327.22) |
| SDI quintiles | | | | | | | | |
| High SDI | 5052.2 (4606.33-5516.54) | 423.31 (318.65-547.41) | 3033.62 (2705.49-3328.96) | 166.51 (146.05-179.64) | 3511.02 (3185.65-3861.78) | 316.03 (242.64-407.32) | 2200.29 (1840.59-2469.87) | 132.72 (104.1-148.97) |
| High-middle SDI | 5532.23 (4906.3-6229.48) | 814.11 (598.94-1075.96) | 8729.01 (7681.89-9881.3) | 528.88 (459.06-602.86) | 4193.55 (3646.67-4797.78) | 621.12 (457.93-821.71) | 6310.89 (5476.93-7034.45) | 401.91 (340.66-450.49) |
| Middle SDI | 4798.97 (4183.13-5493.22) | 710.61 (515.15-943.72) | 7863.89 (6850.47-8940.69) | 476.31 (410.75-547.01) | 4112.81 (3531.89-4780.34) | 546.41 (394.05-735.24) | 5416.47 (4636.07-6202.35) | 326.44 (272.69-377.53) |
| Low-middle SDI | 3455.23 (2982.71-3960.44) | 479.99 (353.63-628.72) | 7029.97 (6028.04-8226.11) | 419.71 (357.62-490.88) | 2807.91 (2450.48-3200.15) | 424.2 (313.95-555.92) | 5749.24 (4905.16-6723.47) | 353.58 (298.69-414.75) |
| Low SDI | 3469.79 (3066.09-3902.68) | 436.85 (320.92-573.85) | 6649.03 (5580.38-8102.3) | 399.04 (332.06-484.41) | 3718.51 (3351.35-4099.14) | 468.86 (348.41-612.52) | 5802.94 (4511.7-7453.61) | 352.26 (268.05-456.76) |
| Age group | | | | | | | | |
| 60-64years | 2637.56 (2344.24-2953.79) | 275.22 (204.78-369.7) | 2614.89 (2363.68-2886.99) | 77.33 (69.69-86.64) | 2140.21 (1907.99-2392.7) | 192.27 (143.18-261.32) | 1539.09 (1350.71-1753.23) | 41.03 (36.44-47.14) |
| 65-69years | 3833.67 (3407.3-4286.97) | 423.91 (287.76-604.49) | 4001.6 (3606.78-4456.92) | 140.91 (125.96-160.64) | 3068.19 (2719.86-3430.11) | 309.58 (211.16-440.85) | 2517.34 (2237.96-2824.28) | 82.15 (73.12-92.7) |
| 70-74years | 5229.77 (4594-5931.89) | 639.95 (467.24-840.3) | 6694.31 (6052.76-7419.19) | 293.18 (264.22-326.73) | 4105.53 (3625.84-4650.42) | 452.97 (326.2-596.23) | 4733.99 (4280.04-5196.44) | 201.14 (181.5-221.65) |
| 75-79years | 6859.17 (6223.22-7621.23) | 928.19 (673.82-1220.6) | 8980.02 (8111.79-9884.01) | 489.54 (438.16-543.73) | 5208.04 (4624.06-5869.42) | 638.23 (455.09-862.19) | 6788.1 (6116.28-7457.65) | 368.34 (330.82-404.68) |
| 80-84years | 8151.69 (7424.25-9004.07) | 1109.29 (841.76-1404.39) | 12858.95 (11687.14-14032.53) | 912.85 (821.16-1004.6) | 6053.53 (5382.45-6887.61) | 932.77 (701.5-1184.18) | 10636.3 (9333.01-11659.11) | 764.37 (662.19-839.95) |
| 85-90years | 7954.31 (7132.03-8867.48) | 1210.22 (979.76-1461.64) | 17378.03 (15550.6-18756.19) | 1604.01 (1424.5-1749.81) | 6646.54 (5777.62-7622.56) | 1293.58 (1047.44-1577.27) | 13391.02 (11204.61-14893.21) | 1223.09 (1011.45-1366.02) |
| 90-94years | 7493.14 (6558.26-8540.46) | 1345.36 (1055.29-1669.68) | 20032.01 (17536.27-21818.85) | 2155.7 (1867.53-2358.66) | 7057.68 (5929.91-8250.16) | 1632.48 (1297.54-2043.73) | 17572.14 (14037.8-19736.01) | 1875.98 (1462.67-2115.84) |
| 95+years | 7780.73 (6616.49-9063.28) | 1513.21 (1140.01-1981.55) | 19197.14 (15663-21186.41) | 2179.94 (1724.39-2413.29) | 7564.08 (6290.7-9046.02) | 1862.12 (1393.41-2396.65) | 23077.08 (17293.94-26430.52) | 2664.41 (1930.3-3065.27) |
| GBD Region | | | | | | | | |
| Andean Latin America | 2547.69 (2346.38-2746.37) | 267.51 (198.5-347.37) | 2304.81 (1768.12-2915.29) | 142.25 (107.79-182.98) | 2380.26 (2196.45-2571.91) | 232.48 (174.79-304.55) | 2005.09 (1602.77-2539.93) | 127.9 (98.87-165.12) |
| Australasia | 4026.91 (3778.93-4295.67) | 359.51 (272.76-452.36) | 1878.43 (1610.51-2143.34) | 108.46 (91.14-123.56) | 2362.18 (2181.34-2545.19) | 252.02 (194.31-321.92) | 1571.94 (1266.34-1816.19) | 109.98 (84.14-126.69) |
| Caribbean | 3089.07 (2838.69-3348.26) | 400.48 (304.6-511.83) | 4552.13 (3879.14-5299.29) | 279.5 (237.21-326.36) | 2722.45 (2514.09-2941.73) | 337.26 (259.38-431.25) | 4121.43 (3485.47-4856.06) | 265.29 (223.1-309.47) |
| Central Asia | 4916.22 (4500.89-5377.61) | 810.95 (627.31-1021.59) | 10602.9 (9546.63-11623.91) | 620.58 (555.59-679.54) | 4783.6 (4409.49-5173.68) | 713.33 (547.21-897.87) | 7952.9 (7091.43-8734.98) | 478.46 (420.33-526.68) |
| Central Europe | 5109.73 (4620.78-5646.98) | 731.29 (568.87-916.13) | 9104.18 (8285.64-9836.69) | 559.41 (504.21-605.46) | 3520.05 (3181.2-3880.33) | 613.89 (483.74-760.53) | 6800.45 (6057.57-7375.13) | 464.43 (406.57-505.42) |
| Central Latin America | 2818.32 (2518.36-3143.01) | 320.6 (237.6-416.3) | 2526.02 (2219.54-2848.21) | 154.16 (133.73-174.59) | 2637.76 (2374.12-2930.47) | 273.15 (204.25-357.75) | 2061.33 (1793.69-2323.92) | 132.02 (111.78-150.04) |
| Central Sub-Saharan Africa | 4285.2 (3867.03-4713.38) | 542.17 (398.72-709.09) | 7484.16 (5356.89-10598.29) | 451.36 (317.02-647.84) | 5961.83 (5460.45-6474.44) | 694.26 (513.9-914.59) | 7684.37 (5167.2-11184.8) | 461.22 (293.46-693.39) |
| East Asia | 6320.69 (5452.88-7280.59) | 1003.93 (715.1-1356.87) | 10308.04 (8403.9-12406.95) | 653.16 (527.45-793.81) | 5350.51 (4464.95-6343.83) | 712.34 (501.48-976.9) | 6253.71 (5023.48-7570.12) | 366.64 (283.85-454.86) |
| Eastern Europe | 4959.13 (4175.37-5790.68) | 892.94 (639.49-1195.66) | 12849.15 (11498.58-14147.18) | 754.2 (667.52-831.43) | 3907.07 (3369.09-4497.14) | 786.07 (568.73-1055.77) | 9492.92 (8381.71-10463.6) | 638.57 (555.7-708.18) |
| Eastern Sub-Saharan Africa | 4684.84 (4182.42-5233.22) | 545.85 (399.95-719.09) | 6234.06 (5062.11-7749.61) | 364.43 (291.55-456.75) | 5711.52 (5158.11-6311.06) | 646.56 (479.36-847) | 5984.76 (4547.74-7720.83) | 348.22 (250.51-463.28) |
| High-income Asia Pacific | 5592.95 (4983.74-6239.02) | 435.86 (323.69-570.64) | 3075.44 (2680.92-3443.82) | 162.57 (139.78-177.86) | 2839.62 (2525.36-3191.93) | 248.11 (184.6-325.25) | 1546.58 (1198.6-1818.62) | 92.16 (63.89-110.05) |
| High-income North America | 5193.64 (4604.43-5816.19) | 323.68 (230.22-437.45) | 2324.36 (2040.62-2581.12) | 124.57 (107.15-134.72) | 4202.1 (3700.32-4730.65) | 286.25 (204.86-388.48) | 2190.94 (1827.1-2475.86) | 130.28 (101.86-145.39) |
| North Africa and Middle East | 4371.84 (3978.77-4788.87) | 602.01 (459.42-770.43) | 8606 (7356.12-9914.61) | 528.14 (447.3-611.8) | 3374.86 (3047.27-3720.73) | 546.78 (418.11-701.25) | 8735.76 (7275.96-10181.9) | 570.8 (467.59-668.28) |
| Oceania | 3890.49 (3584.23-4208.42) | 451 (335.42-587.73) | 4605.99 (3423.16-6389.1) | 261.51 (184.21-373.91) | 3665.3 (3375.36-3957.5) | 399.6 (298.07-521.48) | 5980.8 (4549.27-7960.97) | 356.58 (263.17-485.43) |
| South Asia | 2682.36 (2208.44-3196.1) | 366.5 (260.9-494.38) | 5240.15 (4299.07-6869.05) | 316.7 (257.65-410.16) | 1802.05 (1481.64-2157.01) | 321.65 (232.13-433.38) | 4320.82 (3492.12-5424.07) | 266.68 (213.25-334.77) |
| Southeast Asia | 5076.05 (4470-5727.85) | 667.89 (499.72-864.46) | 9970.53 (8026.1-11729.32) | 581.53 (465.53-688.51) | 4385.64 (3886.48-4914.31) | 566.04 (422.67-738.28) | 7357.06 (6126.18-8671.7) | 461.69 (376.57-548.76) |
| Southern Latin America | 4076.53 (3789.51-4365.45) | 388.58 (291.06-497.86) | 3326.34 (2970.23-3693.03) | 191.22 (168.04-212.93) | 2786.24 (2586.19-2990.65) | 289.39 (221.39-367.67) | 2285.08 (1960.57-2566.93) | 144.77 (119.99-163.47) |
| Southern Sub-Saharan Africa | 5126.41 (4349.73-6034.98) | 658.3 (469.01-886.99) | 7516.44 (6666.31-8420.58) | 445.5 (392.77-500.46) | 6928.38 (5952.55-7952.15) | 822.02 (585.87-1112.83) | 7566.4 (6598.55-8516.85) | 467.76 (398.94-529.81) |
| Tropical Latin America | 3973.38 (3341.69-4673.69) | 493.23 (343.96-671.26) | 4510.28 (4097.86-4833.45) | 274.04 (242.92-295.36) | 2667.15 (2256.48-3118.44) | 317.23 (223.91-432.81) | 2909.74 (2490.6-3191.26) | 189.4 (155.65-210.17) |
| Western Europe | 4244.82 (3933.63-4563.89) | 388.42 (308-479.4) | 2391.13 (2118.75-2629.43) | 140.46 (121.94-151.46) | 2807.17 (2601.84-3018.28) | 295.11 (239.14-356.12) | 1821 (1496.06-2048.13) | 121.38 (94.06-136.63) |
| Western Sub-Saharan Africa | 4678.26 (4157.36-5232.88) | 485.06 (355.28-638.65) | 10312.31 (8608.55-12394.56) | 617.44 (512.72-744.95) | 5372.83 (4811.33-5987.05) | 530.08 (390.03-693.77) | 6925.39 (5181.01-9001.42) | 429.22 (313.43-564.78) |
| 204 countries and regions | | | | | | | | |
| Afghanistan | 4061.5 (3566.5-4580.9) | 680.3 (519.1-889.9) | 13673.9 (9030.7-19615.4) | 832.8 (542.8-1192) | 2644.4 (2285-3032.2) | 601.1 (456.1-774.9) | 12254.3 (7605.6-17979.6) | 752.2 (463.1-1104.4) |
| Albania | 3037.5 (2735.6-3358.5) | 396.3 (292.5-522) | 5248.5 (3651.6-7242.7) | 362.3 (244.1-506.2) | 2551.6 (2295.1-2810) | 394.2 (295.1-519.1) | 4784.2 (3376.5-6568.3) | 343.3 (234.3-480.9) |
| Algeria | 5097.8 (4645.9-5604.6) | 666.5 (496.1-868.1) | 7618 (5403.1-10267.2) | 530.7 (367-722.3) | 4447.5 (4011.8-4886.6) | 698.2 (497-940.7) | 10257.1 (7011-14086.5) | 800.6 (540.1-1097.7) |
| American Samoa | 4154.6 (3816.2-4506.8) | 438 (319.6-580.4) | 4219.7 (3055.6-5720.4) | 237.7 (161.6-335.2) | 4272.9 (3920.8-4639.6) | 465.2 (342.7-617) | 5002 (3584.6-6803.2) | 301.3 (199.4-425) |
| Andorra | 2813.1 (2560.4-3089.2) | 267.7 (191.8-360.9) | 1565.4 (1078.1-2174.4) | 92.3 (56.8-135.9) | 2196.6 (1980.1-2420) | 243.3 (177.9-330.4) | 1747.4 (1218.3-2396.2) | 119.2 (76.7-171) |
| Angola | 5129.3 (4643.8-5623.8) | 598.2 (430.2-799.7) | 8456.2 (5767.8-11806.4) | 504.1 (336.8-713.7) | 7087.4 (6489.8-7723.1) | 762.9 (551.3-1019.6) | 8119.5 (5586-11367.2) | 478.8 (310.5-694) |
| Antigua and Barbuda | 2797.7 (2540.6-3072.2) | 381 (282.4-506.1) | 4385.3 (3670.5-5208.6) | 288.1 (241.5-340.3) | 2823.8 (2564.7-3089.1) | 393.1 (298.5-509.1) | 4040 (3500.4-4591.5) | 283.6 (244.1-323.1) |
| Argentina | 4166.7 (3852.6-4492.4) | 383.4 (282.9-501.8) | 3177.5 (2760.2-3642.9) | 175.5 (149.6-203.7) | 2808.5 (2588.8-3033.6) | 287 (216.6-369.6) | 2154.1 (1832.6-2480) | 130.3 (106.9-151.2) |
| Armenia | 4906.7 (4536.4-5302.3) | 543.2 (417.8-688.6) | 7192 (6383.7-8044.4) | 400.7 (348-448.6) | 3331.9 (3059.1-3632.6) | 401.1 (306.9-507.4) | 5720.7 (5009.8-6407) | 369.9 (318-416.1) |
| Australia | 4121.8 (3883.3-4364.2) | 363.1 (274.1-458.8) | 1819.8 (1545.3-2098.4) | 102.6 (85.1-118.6) | 2351.4 (2182.1-2519.5) | 249 (191.3-319.6) | 1487.1 (1187.8-1732.6) | 103 (78.1-120.2) |
| Austria | 5892.2 (5446.9-6358.6) | 471.1 (357-608.6) | 2339.8 (1986.9-2681.3) | 115.7 (95.9-133.2) | 4183.7 (3873.1-4517.4) | 365.5 (269.2-481.7) | 1747.2 (1426.2-2056.1) | 93.7 (71.9-111.2) |
| Azerbaijan | 3960.1 (3554.9-4403.2) | 591.5 (427.3-779.7) | 5689.6 (4236.4-7496.8) | 326.7 (239.7-433.6) | 3874.6 (3487.6-4284) | 570.4 (420.5-756.2) | 5042.2 (3675.9-6575.4) | 286 (199.4-387.2) |
| Bahamas | 3068.1 (2793.3-3354.8) | 361.8 (266.3-477.4) | 3102.5 (2487-3821.7) | 199.7 (158.1-246.8) | 2770 (2531.6-3030.8) | 339.9 (249.4-443) | 3476.3 (2834.9-4157.3) | 232.8 (187.7-280.5) |
| Bahrain | 2506.9 (2284.9-2753.4) | 303.2 (228.2-393.8) | 6359.7 (4775.1-8128) | 448.9 (332.8-576.3) | 1963.1 (1749.7-2199.7) | 326.1 (247.6-420.4) | 6273.5 (4686.9-8208.9) | 440.3 (326.6-576.1) |
| Bangladesh | 3185.2 (2829.3-3569) | 488.6 (354-648.3) | 8023.4 (5353.3-11715.8) | 534.8 (355.1-775) | 2594.8 (2301.4-2919.7) | 516.4 (385.6-671.1) | 8545.4 (5947.5-11828.6) | 601.9 (416.6-832.3) |
| Barbados | 3197.4 (2901.1-3519.1) | 411.4 (304.5-534.3) | 4693.3 (3670.4-5837.5) | 318.4 (248.6-393.4) | 2818.3 (2568-3101.7) | 399.7 (302.4-514.6) | 4969.9 (4007.3-6044.2) | 359.3 (289.3-435.6) |
| Belarus | 6179 (5560.6-6894.8) | 939.1 (698.3-1228.6) | 12011.3 (9947.3-14251.4) | 676.3 (556.8-803.7) | 4224.4 (3789.9-4683.5) | 739.1 (548.5-979.5) | 7787.9 (6540.4-9118.7) | 495.3 (408.2-583.8) |
| Belgium | 3760.6 (3505.9-4023.5) | 372.3 (282.7-458.6) | 2223.9 (1886.5-2543.4) | 128.5 (105.7-148.8) | 2507.6 (2326.4-2697.4) | 274.1 (217.3-338.1) | 1672.1 (1329.5-1945.6) | 107.9 (80-127.6) |
| Belize | 2530.6 (2290.1-2787.3) | 317.5 (232.7-419.8) | 2989.5 (2457.5-3584.4) | 190.7 (155.1-228.4) | 2128.7 (1936.1-2335.4) | 264.7 (196.4-347.9) | 2997.3 (2506.1-3495.5) | 207.9 (171.8-243.9) |
| Benin | 4576.2 (4174-4990) | 483.2 (348.1-642.3) | 10780.4 (7840.6-14534.4) | 649.8 (466.6-878.6) | 5306.6 (4867.9-5765.9) | 533.5 (386.4-723.2) | 8321.9 (5656.3-11789.4) | 531.5 (351.4-760.4) |
| Bermuda | 2909.1 (2651.4-3182.8) | 339.6 (245.6-454.5) | 2997 (2459.7-3630.6) | 194.5 (158.3-236.8) | 2438.4 (2233.8-2651.2) | 235.1 (170.1-316.2) | 1864.3 (1496.4-2322.6) | 127.7 (98.5-162.5) |
| Bhutan | 3012.3 (2678.5-3352.5) | 409.6 (299.8-544.7) | 4996.1 (3280.4-7145.7) | 306.4 (195.4-446.1) | 2313.2 (2048.5-2587.7) | 361.3 (264.7-480.6) | 4524.7 (2948.9-6677.1) | 280.7 (174.9-424.4) |
| Bolivia (Plurinational State of) | 2315.5 (2072.7-2569.2) | 308.6 (221.3-416.7) | 3562.7 (2184-5443.4) | 226.7 (136.5-349) | 2229.9 (1991.2-2480.5) | 284.2 (202.5-388.6) | 3545.1 (2203.7-5375.5) | 239 (145.2-363.6) |
| Bosnia and Herzegovina | 6150.9 (5577-6777.5) | 883 (660.7-1151.6) | 11652.2 (8844.9-14476.4) | 696 (516.5-872.3) | 4543.5 (4102.4-5000.5) | 815.2 (615.1-1047.2) | 10474.6 (8213.7-12849.9) | 688.9 (526.4-853.6) |
| Botswana | 6468.4 (5839.2-7152.5) | 948 (699.6-1249.8) | 8410.4 (5968.1-11523) | 481 (331.9-672.3) | 8383.3 (7651.3-9144.9) | 1092.9 (801.6-1465) | 7157.2 (5255.5-9670.5) | 411.6 (280.5-581.4) |
| Brazil | 3973.3 (3331.2-4685.6) | 492.7 (342.5-672.6) | 4486.5 (4068.1-4813) | 272.2 (240.8-293.5) | 2667.4 (2250.7-3124.9) | 316.8 (222.7-433.2) | 2892.3 (2479.7-3174.9) | 188.1 (154.5-208.9) |
| Brunei Darussalam | 6889.3 (6416.3-7391.3) | 599.1 (431.9-798.4) | 5594.6 (4280.5-7158.1) | 349.1 (254.7-465.2) | 4047.8 (3712.9-4395.3) | 454.2 (340.8-589.8) | 4343.9 (3233.1-5600.4) | 282.5 (200.5-373.9) |
| Bulgaria | 6175.9 (5154.9-7265.3) | 1137.9 (862.4-1471.7) | 19931.5 (17300.1-22788.4) | 1289.1 (1129.4-1464.3) | 4524.7 (3901-5219) | 961.4 (748.2-1214.9) | 14116.4 (12278.8-15935.3) | 1012.5 (879.4-1138.6) |
| Burkina Faso | 3499 (3196.7-3808.4) | 350.8 (253-473.3) | 7996.6 (5276.8-11673.1) | 492.5 (322.3-718.8) | 3798.2 (3486.6-4109.9) | 350.6 (254-470.8) | 4132.2 (2629.3-6203.2) | 258.4 (153.3-401) |
| Burundi | 4390.2 (3922.3-4863.5) | 526.2 (378.5-709.9) | 6305.4 (4114.2-9405.1) | 378.3 (237.4-574.2) | 5572.5 (5048-6100.3) | 634.2 (461.5-840.9) | 7188.6 (4715.4-10814.6) | 427.1 (262.1-663.7) |
| Cabo Verde | 4831.3 (4420.3-5247.9) | 485.9 (348.5-646.5) | 11761.6 (8482.6-16084.5) | 724.5 (514.2-993.5) | 4816.8 (4464.5-5193.3) | 415.9 (297.3-573.2) | 5624.4 (3967.7-7645.9) | 367 (246.3-513.7) |
| Cambodia | 4017.6 (3574.6-4497.1) | 670 (496.5-879.4) | 9683.6 (6797.1-13298.6) | 622.5 (426.4-866.1) | 3526.7 (3153.1-3916.5) | 587.3 (428.2-782.4) | 8395.3 (6002.9-11204.6) | 556.9 (389.2-752.5) |
| Cameroon | 4678.3 (4260-5097.6) | 486.9 (358.1-645.1) | 10915.6 (7282.3-15788.8) | 655.6 (433.2-950.2) | 5086.6 (4638.4-5531.2) | 500.3 (367.2-674.5) | 7059.9 (4577.6-10515.3) | 441.7 (274.2-665.5) |
| Canada | 5281.5 (5039.9-5528.9) | 367.4 (285.3-463.1) | 1910.9 (1622.9-2196.4) | 95.4 (79.2-110.4) | 3509.4 (3339.4-3681) | 268.4 (213.6-331.2) | 1604.4 (1302.5-1863.4) | 93.5 (71.1-109.4) |
| Central African Republic | 3960 (3476.3-4436) | 534 (389.5-715.2) | 8767.2 (5436-13648.6) | 512.3 (304.7-806) | 5642.4 (5073.1-6235.2) | 696.8 (507.1-932.2) | 9076.7 (5346.3-14112.9) | 555.4 (309.2-878.9) |
| Chad | 4524.5 (4095.7-4955.5) | 470.6 (342.2-634.5) | 11599.8 (7962-16244.8) | 687.5 (462.8-961.7) | 5398.8 (4926.1-5879.8) | 525.3 (385-697.9) | 7847.8 (4843.6-11881) | 477.4 (280.9-738.7) |
| Chile | 3827.9 (3531.6-4157.2) | 391.2 (285.3-513.9) | 3335.4 (2907.8-3770.7) | 201.3 (172.5-229.1) | 2646.7 (2424.2-2881) | 275.8 (206.5-364.3) | 2302.9 (1917.5-2644.8) | 155.6 (124.8-180.2) |
| China | 6300.9 (5412.1-7284.4) | 1011.2 (717.4-1370.9) | 10512.2 (8526.7-12690.6) | 672.6 (541.4-819.6) | 5370 (4455.3-6393.4) | 719.2 (504.3-990.1) | 6331.7 (5062.2-7695.4) | 372.8 (287.1-464) |
| Colombia | 2584.4 (2388-2793.4) | 281.9 (207.2-366.2) | 2118.5 (1705.9-2591.5) | 126.3 (100-156) | 2447.9 (2261.4-2654.7) | 246.2 (179.3-329.4) | 1619.2 (1310.9-1936.1) | 98.4 (76.2-120.1) |
| Comoros | 4903.3 (4448.1-5355.3) | 544.7 (398.8-723.6) | 6187 (3951-9118.9) | 372.7 (228.3-564.7) | 6725.3 (6177.9-7310.9) | 711 (511.5-954.7) | 6490.5 (4224.4-9411.6) | 372.5 (222-561.4) |
| Congo | 5162.1 (4672.3-5681) | 621.6 (451.2-821.8) | 8023.7 (5335.7-11329.5) | 479.1 (308.1-686.7) | 7738 (7052.9-8454.7) | 863.1 (630.5-1153.8) | 9644.8 (6453.1-13453.8) | 575.1 (364.4-828) |
| Cook Islands | 4792.6 (4427.2-5169) | 458.2 (330.4-616.5) | 3580 (2535.1-4889.7) | 189.4 (120.8-279) | 4273.6 (3942.9-4615) | 402.2 (292.2-544.3) | 3154.3 (2222.1-4344.1) | 173 (107.3-258.4) |
| Costa Rica | 2681.5 (2468.9-2911.5) | 299.4 (220.9-397.2) | 2021.7 (1687.6-2359.6) | 130 (106.4-153) | 2618.6 (2404.6-2838.3) | 261.9 (190.9-356) | 1785.8 (1470.8-2074.8) | 117.9 (93.3-138.8) |
| Coted'Ivoire | 5107.7 (4632.6-5583.3) | 518 (379-688.4) | 11972 (8280.5-16671.9) | 710.4 (491.7-990.2) | 5446.9 (5012.4-5916.5) | 530.1 (383-709.4) | 7324.8 (4947.6-10385.7) | 455.5 (294.8-655.5) |
| Croatia | 5780 (5470.4-6095.2) | 754 (640.4-876.3) | 6632.6 (5648.6-7623.2) | 402.6 (334.7-468.5) | 3203.1 (2980.9-3429.7) | 559.2 (462-660.5) | 4968.8 (4289.3-5632.3) | 353.8 (300.3-402.4) |
| Cuba | 3044.5 (2759.4-3336.5) | 389.3 (289-508.3) | 4601.9 (3821.2-5450.2) | 287.9 (237.6-340.6) | 2498.9 (2284.1-2732.6) | 315.1 (235.3-410.4) | 3529.3 (2952.3-4156.4) | 237.5 (197.3-281.2) |
| Cyprus | 1690.6 (1497.4-1911.8) | 263.9 (203.1-335.8) | 2976.5 (2152.8-3982.9) | 239.4 (170.8-321.7) | 1342.2 (1122.7-1633.6) | 266.2 (210.2-331.7) | 3063.1 (2225.3-4001.2) | 270 (193.2-355.5) |
| Czechia | 5412.2 (5011.3-5828.2) | 616.2 (461.4-796) | 4629.4 (3990.9-5271.9) | 259.6 (220.3-300) | 3624.9 (3351.2-3911.9) | 505.2 (384.7-643.2) | 3393.9 (2890.8-3867.7) | 216.3 (178.6-249) |
| Democratic People's Republic of Korea | 6615.2 (5855.3-7384.1) | 1011.8 (739.1-1325) | 10607.9 (7524.5-14484) | 576.4 (388.7-807.2) | 5361.3 (4839.1-5887.8) | 699.8 (519.6-918.6) | 7450.8 (5098.6-10488.5) | 411.8 (257.9-604.7) |
| Democratic Republic of the Congo | 3894.7 (3443.3-4358.1) | 514 (369.9-684.3) | 7046.1 (4259.5-11373.4) | 428.5 (252.2-697.4) | 5479.5 (4953.8-6029.6) | 659.9 (480-883.3) | 7374.3 (4250.8-11837.3) | 445.8 (236.5-741.2) |
| Denmark | 3602.2 (3303.6-3917.3) | 352.2 (259.5-465.6) | 2608.2 (2238.4-2965.9) | 162.5 (136.8-186.4) | 2481.1 (2271.5-2696.6) | 254.8 (189.4-338.4) | 1937.3 (1592.1-2225.4) | 133.3 (105.1-154.7) |
| Djibouti | 5932.7 (5421.4-6486.3) | 676.7 (491.2-898.5) | 8036.7 (5339.5-11577.9) | 477.8 (307.3-699.4) | 7308.4 (6713.3-7932.7) | 776 (567.9-1035.8) | 6242.9 (4176.1-8963.5) | 351.5 (211.4-530.4) |
| Dominica | 2574.1 (2298.8-2855.4) | 376.7 (272.8-497.7) | 5659.2 (4064.9-7644.9) | 374.4 (267.2-506.2) | 2533 (2291.6-2797.4) | 356.9 (266.3-468.1) | 5999.9 (4488.6-7713.9) | 427.7 (319.2-548.1) |
| Dominican Republic | 3776.9 (3380.4-4203.3) | 470.2 (342.4-622.6) | 4509.1 (3012-6510.4) | 266.7 (173.9-389.4) | 3323.1 (3019.4-3648.9) | 381.4 (281.4-505.2) | 3839.9 (2686.9-5274.9) | 246 (167.3-342.6) |
| Ecuador | 2910.5 (2660.7-3184.2) | 313.6 (231.1-419) | 2468 (1970.4-3069.9) | 163.2 (129.7-202.4) | 2700.9 (2476.2-2936.3) | 271.2 (201-356.7) | 1988.3 (1614.9-2412.4) | 134.9 (107.8-164.4) |
| Egypt | 4561.7 (4027.1-5117.9) | 699.5 (510.5-933.7) | 13991.1 (10039.2-19019.1) | 836.2 (595.3-1136.6) | 3658 (3153-4173) | 778.8 (595.1-993.7) | 22658.5 (16512.8-29579.5) | 1631.6 (1199.3-2110.3) |
| El Salvador | 2300 (2103.7-2510.4) | 279.7 (207.2-371.7) | 2129.2 (1514.1-2900.6) | 130.3 (90.5-179.8) | 2283.9 (2088.7-2486.3) | 245.6 (178.1-328.1) | 1764.5 (1267.9-2389.3) | 110.5 (75.7-153.4) |
| Equatorial Guinea | 4706.4 (4279-5136.1) | 527.8 (375-720.1) | 7187.2 (4522.7-10659.5) | 429.8 (258-647.9) | 6672.9 (6131.6-7257.1) | 719.1 (518.8-973.7) | 7524.2 (4819.2-11476.1) | 447.8 (266.4-705.3) |
| Eritrea | 4695.6 (4222.8-5169.8) | 513.5 (365.6-696.8) | 7206.9 (4661.3-10570.8) | 435 (273.9-645.8) | 6194.7 (5646.7-6757.7) | 680 (490.8-930.2) | 7037.3 (4360.1-10402.8) | 418.4 (242.5-637.2) |
| Estonia | 3994.7 (3586.2-4417.9) | 464.6 (337.4-614.1) | 4908.6 (4159.2-5691.9) | 284.9 (237.5-333.3) | 2625.4 (2358.6-2914.5) | 344.8 (245.6-459.6) | 2573.8 (2133.3-2995.2) | 160.6 (130-188.3) |
| Eswatini | 5200.7 (4633.6-5790.9) | 797.1 (591.1-1059.4) | 9374.6 (5911.9-14308.5) | 514.9 (311.3-802.8) | 6389.6 (5782.2-7015.5) | 922 (682.1-1213.3) | 9558.5 (6133.4-13850) | 584.7 (362.1-859.2) |
| Ethiopia | 3188.9 (2597.8-3842.8) | 386.2 (268.2-527.6) | 3313.2 (2356.2-4611.8) | 196.1 (133.1-278.6) | 4168.5 (3408.2-5011.9) | 472 (328.2-649) | 3964.9 (2811.2-5560.6) | 225.4 (149.7-327.4) |
| Fiji | 5162.4 (4716.3-5632.6) | 601.1 (434.9-795.3) | 6224.2 (4416.3-8447.5) | 385.5 (259-537.3) | 4941.3 (4521.7-5362.6) | 515.7 (373.9-680.1) | 5319 (3795.7-7244.6) | 307.3 (203.9-438.2) |
| Finland | 5367.5 (4980-5787.9) | 483 (368.3-615) | 2876.1 (2453.6-3285.1) | 158.3 (131.2-183.2) | 3747.9 (3469.1-4042.2) | 371.2 (282.3-483.5) | 2194.2 (1784.4-2541.7) | 138 (105.5-162) |
| France | 4020.1 (3762.2-4294.5) | 333.1 (269.5-403.7) | 2055.5 (1738-2360.7) | 116.9 (96.1-135.5) | 2425.8 (2254.6-2601.6) | 235 (186.6-289.4) | 1406.8 (1125.3-1651) | 92.5 (69.7-108.9) |
| Gabon | 5639.5 (5117.2-6178.5) | 638.5 (464.6-841.6) | 7783.9 (5219.9-10808.7) | 465.2 (300-659.4) | 7221.2 (6663.2-7815.2) | 771.8 (560.7-1024.4) | 7278.3 (4707.8-10386.4) | 433.8 (260.3-639.2) |
| Gambia | 5096.1 (4666.6-5555.6) | 524.3 (381.9-700.7) | 13747.4 (9391.2-19004.7) | 821.1 (552.8-1138.8) | 5879 (5398.2-6388.1) | 575.7 (418.4-773.1) | 9097.9 (5636.6-14014) | 572.2 (338.1-893) |
| Georgia | 4385 (3860-4955.1) | 800.9 (606-1031) | 14524.8 (12829.2-16170.6) | 936.5 (824.8-1041) | 4214.2 (3819.9-4651.2) | 687.1 (522.4-880.4) | 9787.2 (8420.2-11022) | 629.4 (532.7-713.6) |
| Germany | 5791.5 (5369.6-6233.8) | 499 (374.6-639.6) | 2876.8 (2469.6-3297.7) | 150.4 (124.7-174.4) | 4341.7 (4059.8-4630.5) | 417.1 (337.8-502.4) | 2123.7 (1736.2-2457.3) | 119.5 (89.7-141.2) |
| Ghana | 7044.9 (6461.6-7650.1) | 683.4 (496.7-902.1) | 16238.3 (11854.1-21400.5) | 960.7 (695.2-1268.9) | 7780.2 (7171-8402.2) | 748.1 (544.4-983.4) | 10146.5 (6763.8-14753.5) | 630.7 (405.8-925.8) |
| Greece | 4214 (3845.2-4598.8) | 461.2 (342.9-596.7) | 3215 (2739.8-3658.6) | 207.3 (172.8-238.1) | 2773.5 (2506.9-3050.2) | 427.1 (328.1-550.8) | 3259.5 (2675.7-3712.5) | 259.3 (206-294.7) |
| Greenland | 6335 (5849.7-6830.9) | 667.7 (505.9-858) | 4947.7 (3737.9-6406.6) | 308.4 (223.3-410.8) | 4037.5 (3704.8-4383) | 434.5 (329.4-566.9) | 3672.3 (2746.5-4821.1) | 250.9 (179-336.9) |
| Grenada | 3596.4 (2964.2-4306.8) | 666.3 (515.7-835.7) | 6648.1 (5522.5-7820.5) | 442.1 (364-525.1) | 3185.8 (2817.8-3647.3) | 508.7 (394.7-644.9) | 5460 (4622.2-6265.5) | 374.1 (315-429.3) |
| Guam | 4798.1 (4460.7-5155.2) | 431.8 (318.6-574.5) | 2658.6 (2078.7-3364.7) | 107.5 (76.8-145.4) | 4482.2 (4159.9-4802.8) | 364.7 (265.9-492.8) | 2086.7 (1620.9-2633.1) | 68.8 (45.7-96.7) |
| Guatemala | 2120.1 (1929.6-2315.5) | 293.5 (219.5-378.4) | 2218.1 (1807.6-2654.5) | 153.4 (125-183.2) | 2156.8 (1971-2349.8) | 259.7 (193.7-339.4) | 1977.9 (1639.2-2342.3) | 136 (110.8-161.4) |
| Guinea | 4938 (4484.8-5396.1) | 513.5 (373.3-685) | 11564 (7990.7-16305.4) | 681.1 (466.4-957.5) | 5468.6 (5009.6-5937.8) | 550.2 (402.7-735.8) | 8302.4 (5266.2-12237.7) | 513.6 (314.7-766.6) |
| Guinea-Bissau | 5147.2 (4662.5-5628.9) | 538.4 (390.6-723.8) | 16443 (11819.1-22021.5) | 958 (681-1287.6) | 5923 (5422.8-6437) | 574 (414.8-769.9) | 10416.4 (6533.8-15318.1) | 654.1 (395.8-973) |
| Guyana | 3740.4 (3340-4165.4) | 626.1 (460.5-814.3) | 8674.4 (6520.6-11187.2) | 529.4 (395.9-679.4) | 3319 (2978.9-3690.9) | 528.5 (396-685.1) | 8098.2 (6337.8-10084.7) | 549.4 (425.6-684.1) |
| Haiti | 2955.3 (2593.4-3345.2) | 507.7 (374.6-672.6) | 8171.8 (5053.8-12315.3) | 527.9 (326.7-792.4) | 2844.9 (2489.7-3221.1) | 529.2 (399-692.7) | 11477.7 (7339.3-16996.3) | 764.5 (492.8-1118.4) |
| Honduras | 2684.4 (2399.6-2993.9) | 418.5 (305.7-557.1) | 6840.3 (4356.3-9679.6) | 442.2 (279-626.4) | 2461 (2209.2-2730.4) | 364.8 (267.6-480.8) | 5888.6 (3814.5-8597.7) | 403.8 (258.6-587.7) |
| Hungary | 4904.4 (4459.1-5388.7) | 620.5 (456.5-818.5) | 6665 (5626.2-7766) | 372.4 (310.5-439.8) | 3494.6 (3188.2-3804.1) | 503.4 (375.4-657.3) | 4492.9 (3821.3-5128.4) | 282.5 (233.1-326.6) |
| Iceland | 3975.3 (3661-4311.7) | 361 (267.7-481.9) | 1780.1 (1489.5-2079.2) | 98.4 (78.8-115.9) | 2554.7 (2343.7-2769.2) | 245.4 (182.9-323.2) | 1593.5 (1261.8-1880.1) | 108 (80.1-128.9) |
| India | 2527.5 (2038.4-3065.2) | 338.7 (236.9-465.6) | 4746.9 (3894.9-6323.4) | 279.4 (228.1-365.4) | 1605.6 (1293.5-1952.2) | 288 (203.5-394.6) | 3706.7 (2930.2-4764.4) | 222.3 (173.6-284.2) |
| Indonesia | 5837.4 (4741.6-7066.4) | 877.4 (611.7-1200.7) | 12875.3 (9075.8-16579.7) | 785.3 (548.3-1011.1) | 5737.5 (4735.2-6821.4) | 827 (580.1-1131.7) | 11110.9 (8254.7-14207.8) | 716.8 (521.4-923.8) |
| Iran (Islamic Republic of) | 3963 (3323.4-4692.6) | 475.3 (331.1-646.8) | 6160.4 (5416.2-6888.4) | 375.4 (323-422.6) | 2786.9 (2341.8-3288.3) | 438.6 (311-596.8) | 6288.3 (5397.4-7064.2) | 422.9 (353.6-479.1) |
| Iraq | 5268 (4703.9-5867.9) | 903.5 (676-1177.9) | 17043.6 (12321.3-22148.1) | 1068.7 (776.7-1384.4) | 4036.8 (3581.4-4511.5) | 723.2 (541.8-945) | 12294.7 (9243.7-15827.4) | 802 (600.5-1030.3) |
| Ireland | 2299.4 (2081.7-2528.6) | 244.6 (181.9-322.3) | 1785.6 (1487.6-2069.6) | 120 (97.5-141) | 1714.9 (1544.4-1899.9) | 212.1 (159.2-280.6) | 1434.1 (1122.9-1678.6) | 106.1 (78.9-125.6) |
| Israel | 4346.8 (4001.7-4726.6) | 302.8 (220-412.1) | 1726.8 (1450.8-2003.7) | 84.1 (68.4-98.7) | 2659.7 (2445.2-2881.2) | 223.7 (162.4-303.3) | 1361.3 (1096.2-1598.6) | 84.3 (64-99.6) |
| Italy | 2842.2 (2386.3-3338.5) | 309.6 (232.1-401.6) | 2357.6 (2053-2597.5) | 164 (139.2-178.3) | 1712.4 (1432.7-2018) | 224.6 (169.1-288.5) | 1821.8 (1433-2077.3) | 140.9 (104.8-162) |
| Jamaica | 2733.1 (2453.2-3053.1) | 445.5 (323.2-581.8) | 5310.2 (4042.9-6750.3) | 353.9 (268.7-448.5) | 2359.3 (2121.2-2609.9) | 402.4 (301.4-527.8) | 5312 (4185.8-6583.3) | 370.9 (289.6-461.3) |
| Japan | 5452.6 (4724.1-6230.3) | 427 (311.2-567.4) | 2887.6 (2516-3239) | 151 (131.2-162.8) | 2673.2 (2305.4-3079.5) | 228.5 (166.5-304.4) | 1344.1 (1037.4-1592.2) | 79 (54.8-94.1) |
| Jordan | 7017.6 (6427.4-7618.7) | 814 (608.1-1059.5) | 6003.5 (4618.2-7693) | 336.9 (247.6-443.5) | 5029.9 (4570.1-5532.5) | 746.4 (562.6-963.7) | 7624.5 (5787.2-9668.7) | 510.1 (378.5-653.4) |
| Kazakhstan | 5797.7 (5203.3-6484.7) | 968.5 (728.8-1257.4) | 14320.9 (12519.6-16202.3) | 872.4 (761.4-987) | 5864.7 (5305.1-6441.2) | 849.8 (631.2-1101.3) | 10617.4 (9259.9-11958.2) | 706.9 (611.3-798.1) |
| Kenya | 4557.9 (3740.6-5479.1) | 546.2 (382.2-745) | 5286 (4091.9-6900.5) | 321.4 (240.9-426.1) | 5707.9 (4743.1-6782.8) | 628.3 (439.4-866.6) | 5424.8 (3852.2-7537.1) | 324.1 (212.3-466.6) |
| Kiribati | 5912.7 (5331.3-6540.3) | 790.4 (575-1045.4) | 10124.4 (6816.1-14072.7) | 598.9 (382.4-847.5) | 4817.4 (4376.8-5291.2) | 537.9 (393.7-711.3) | 5264.9 (3688-7295.8) | 302.2 (194.3-441.9) |
| Kuwait | 4700.4 (4339.2-5086.6) | 496.7 (366.9-647.6) | 4037.8 (3230.2-4953.3) | 240.2 (186.7-302.3) | 2946.5 (2689.8-3223.7) | 333.7 (251.1-433.7) | 2185.6 (1743.7-2608.8) | 126.1 (93.3-155.4) |
| Kyrgyzstan | 3747.9 (3322-4229.3) | 664.6 (499.7-877.5) | 10406.7 (8714.9-12177.1) | 573.1 (475.2-671.5) | 3657.7 (3309.1-4028.6) | 546.8 (390.6-728.6) | 6188.9 (5204.5-7191.5) | 337.9 (276.3-398) |
| Lao People's Democratic Republic | 4888.4 (4375-5427.7) | 708.2 (517.8-925.7) | 11128.2 (7592.4-15488.7) | 674.3 (447.8-942.9) | 3911.1 (3518.4-4329.9) | 569.3 (416.2-755.6) | 8364.3 (5967.1-11363.4) | 526.5 (366.2-724.3) |
| Latvia | 7157.2 (6515.9-7808.1) | 980.4 (745.5-1237.3) | 12684.4 (10959.3-14432.7) | 754 (645.9-861.7) | 3963.8 (3541.6-4403.2) | 761.3 (583.7-969.9) | 8880.2 (7597.8-10010.6) | 620.1 (521.7-700.3) |
| Lebanon | 4616.7 (4150-5116.8) | 571.9 (418.2-764.1) | 3817 (2782.6-5104.7) | 229 (158-314.8) | 3063.6 (2736.9-3420.8) | 428.1 (313.2-572.9) | 3088.7 (2278.3-4048) | 202 (141.5-272.4) |
| Lesotho | 4047.1 (3546.7-4558.5) | 707.7 (518.6-923) | 10810.9 (6464.9-16721.1) | 599.2 (350.1-930.8) | 6324.7 (5640.8-7042.5) | 997 (724.1-1309.1) | 11389.6 (7715.6-16157.5) | 702.7 (464.8-1001) |
| Liberia | 4019.8 (3668.5-4372.5) | 406.7 (296-548.6) | 10051.8 (6943.6-14116.3) | 611 (420.6-861) | 4957 (4537.1-5388.9) | 478.3 (348.1-639.4) | 8331.6 (5298.3-12606) | 524.9 (322.5-802.3) |
| Libya | 3273.4 (2948.3-3616.1) | 441 (324.9-578.6) | 6477.9 (4287.1-9435.8) | 396.3 (257.1-582.7) | 3306.8 (2949.5-3701.6) | 469.3 (342.7-620.7) | 7257 (4697.9-10692.3) | 439.2 (274.3-652.4) |
| Lithuania | 5245.1 (3975.3-7028.6) | 1043.8 (770.4-1369.8) | 8564.7 (7371.3-9813.7) | 499.4 (425.4-570.4) | 3487.9 (2845.1-4221.3) | 806.9 (605.7-1045.1) | 5349.9 (4530.1-6132.7) | 359.2 (301.2-410.9) |
| Luxembourg | 2683.5 (2487.3-2887.7) | 290.6 (226.8-361.6) | 2293.5 (1966.6-2616.5) | 152.8 (127.9-176.2) | 1648.2 (1512.2-1789.5) | 204.4 (162.1-254.2) | 1423.4 (1163.4-1649.3) | 103.4 (81.6-120.3) |
| Madagascar | 6005.6 (5442.4-6579.3) | 645 (463.5-866.1) | 9403.3 (5978-13763.1) | 569.5 (346.1-844.7) | 7924.8 (7272-8625.1) | 847.2 (616.3-1130.2) | 9094.6 (5856.9-13365.4) | 526.5 (317.2-797.9) |
| Malawi | 5284.1 (4769.8-5818.1) | 641.3 (468.1-847.4) | 9246 (6028.8-13460) | 544.2 (347.6-797.4) | 5566.9 (5060.9-6078.7) | 650.9 (478.1-871) | 7859.5 (5231.1-11191.8) | 482.3 (308.6-701.3) |
| Malaysia | 5382.8 (4938.4-5859.5) | 543.8 (394-721.1) | 5514.5 (4142.6-7196.2) | 285 (199.7-388) | 4815.5 (4397.4-5240.6) | 556.5 (409.4-742.1) | 5889.9 (4429.9-7655.8) | 363.4 (258.2-489) |
| Maldives | 4050.6 (3693.7-4434.6) | 512.8 (375.1-673) | 5373.2 (4058.8-6888.1) | 341.3 (248.7-444.7) | 3132.8 (2858.2-3437.1) | 393.6 (292.1-517.9) | 4308.6 (3237.6-5511.5) | 277.1 (198.1-365.2) |
| Mali | 3526.1 (3199.5-3842.6) | 422.4 (311.5-550.4) | 6865.9 (4610.9-9792.8) | 413.3 (271.3-595.3) | 4176.8 (3824.2-4552.1) | 416.6 (302.8-561.8) | 6823.8 (4053.2-10736.2) | 419.6 (234.7-667.4) |
| Malta | 2231.6 (2025-2454.3) | 252 (188.6-326.9) | 1800.7 (1507.9-2095.9) | 117.2 (95.1-136.9) | 1609.1 (1453.1-1776.5) | 207.6 (157.6-269.2) | 1656.6 (1334.7-1939.6) | 117.9 (90.8-140.1) |
| Marshall Islands | 4513.7 (4067.6-4992.5) | 577.4 (427.8-772.4) | 6583.9 (4189.3-10017.5) | 388 (232.4-606.8) | 4791.2 (4310.6-5295.5) | 593.2 (433.6-787.5) | 7130.2 (4740.3-10428.4) | 432.6 (273.3-648.1) |
| Mauritania | 4469 (4098.7-4837.9) | 429.3 (308-579.8) | 9366 (6223.2-13595.3) | 577.4 (375-844.5) | 5927.9 (5452.1-6432.9) | 558.4 (407.3-739.4) | 8476.9 (5175.1-13104.6) | 528.6 (307.4-828.5) |
| Mauritius | 4188.7 (3829.8-4576.7) | 547.6 (402.8-708.5) | 5586.6 (4859.2-6373.6) | 314.7 (270.4-361.2) | 3447.4 (3149.5-3744.2) | 425.1 (308.3-566.4) | 3890.4 (3346.1-4437.9) | 230.5 (193.5-266.3) |
| Mexico | 2993.3 (2532.5-3503.9) | 322.4 (225.4-443.7) | 2324.8 (1968.7-2685.3) | 138.9 (115.9-160.8) | 2795.6 (2378.8-3251.2) | 275 (193.1-379.8) | 2010.7 (1753-2280.8) | 130.2 (111.7-147.5) |
| Micronesia (Federated States of) | 4869.2 (4403.8-5369.3) | 605 (442.3-803.3) | 7159.8 (4661.5-10662.7) | 413.7 (257-631.1) | 4850.8 (4388.8-5326.7) | 594.1 (433.5-786.3) | 6769 (4591.8-9679.2) | 405.8 (258.7-598.6) |
| Monaco | 3951.9 (3585.4-4324.6) | 381.8 (277.3-510.4) | 3549.5 (2590.9-4707) | 235.8 (164.4-322.9) | 2982.7 (2694.3-3277) | 354.7 (260.1-476.4) | 3439.7 (2357.2-4702.3) | 249.5 (160.7-348.9) |
| Mongolia | 3738.6 (3355.6-4129.8) | 389.8 (278.8-519.8) | 4248.8 (2929.9-5857.4) | 227.3 (144.8-326.1) | 3645.6 (3315-3980.1) | 328.5 (230.6-449.8) | 2800.2 (2021.7-3710.8) | 144.3 (94.3-204.8) |
| Montenegro | 3631.4 (3276.4-4001.3) | 559.1 (417.5-739.1) | 7637.9 (5578.7-10152.8) | 534.4 (382.6-720.2) | 2774.2 (2495.5-3076.2) | 456.1 (339.6-592.3) | 6511.8 (4738.6-8691.4) | 486.7 (347.6-655.2) |
| Morocco | 4798.6 (4282.9-5358.1) | 783.3 (586.3-1021.6) | 10399.4 (7056.1-14241.4) | 652.8 (437.3-899.6) | 3492.5 (3099.8-3917.2) | 613.1 (460.5-805.8) | 11428.4 (7813-16214.2) | 730.3 (497.3-1037.5) |
| Mozambique | 6506 (5825.1-7266.9) | 790.2 (575.9-1036.4) | 15082.2 (9972.9-21165.3) | 843.2 (552-1189.9) | 6395.8 (5775.8-7032.2) | 781.4 (570.8-1043.7) | 9415.1 (5952.7-13884.4) | 558.3 (331.5-846.8) |
| Myanmar | 4830 (4345.1-5336.8) | 688.8 (504.3-898.9) | 12033.3 (8333.5-16445.2) | 740.9 (502.2-1022.9) | 3537.2 (3194.3-3915.7) | 508.9 (377.1-671.7) | 7071.7 (4950.8-9797.8) | 444.6 (298.8-633.5) |
| Namibia | 5579.4 (5003-6187.7) | 854.9 (629.4-1126.5) | 11420.5 (8223.8-15251.9) | 681.3 (483.9-917.1) | 6664 (6055.3-7265.6) | 923.5 (676.9-1226.4) | 9308 (6351.3-12747.8) | 583.5 (383.7-811.9) |
| Nauru | 6094.3 (5617.3-6574.5) | 521.8 (382.8-700.1) | 10379.3 (6903.7-14615.4) | 585.3 (376.1-842.3) | 6159.6 (5716.4-6622.8) | 434.3 (313.1-588.3) | 9767.3 (6455.5-13701.2) | 578.7 (360.4-831.2) |
| Nepal | 2511.2 (2182.2-2834.6) | 398.5 (289.6-530.4) | 5723.7 (3671.8-8615.4) | 354.2 (222.8-536.2) | 1830 (1604.4-2069.3) | 325.5 (237.8-433.4) | 4597.4 (2895.7-6929.1) | 292.8 (180.7-445.2) |
| Netherlands | 3781.3 (3444.2-4124.6) | 348.6 (253.8-469.3) | 2638.3 (2250-3013.4) | 171.7 (143-197.3) | 2749.6 (2504-3010.9) | 295.5 (218.9-392.7) | 2152.8 (1734.4-2494) | 152.9 (117.3-179.2) |
| New Zealand | 3513.1 (2879-4241.6) | 340.5 (246.3-452.8) | 2213.8 (1888.5-2529.5) | 142.6 (120.6-161.8) | 2427.3 (2022.4-2899.7) | 269.2 (194.9-357.7) | 2050.3 (1641.1-2380.1) | 149.9 (113.9-175) |
| Nicaragua | 2959.1 (2682.6-3251.6) | 379.3 (277.3-498) | 2328.9 (1651-3186.6) | 143.2 (98.2-200.4) | 2731.8 (2485-2999.4) | 311.6 (226.9-411.4) | 1939.1 (1371.8-2641.9) | 120 (81.1-168.1) |
| Niger | 3838.1 (3468.3-4211.6) | 425.7 (308.1-580.9) | 8204.7 (5343.1-12510.1) | 513.8 (327.6-782.7) | 4757.1 (4328.5-5183.9) | 484.2 (351.1-645.4) | 6424.7 (3569.4-10433.2) | 399.9 (209.5-667.3) |
| Nigeria | 4499.7 (3687-5412.5) | 478.4 (333.7-655.4) | 9323.8 (6961.6-12591.9) | 563.6 (420.1-763.5) | 5177.9 (4300.7-6154.5) | 522.7 (365.9-718) | 5982.7 (4365.2-8210.3) | 368 (259.1-509.6) |
| Niue | 5308.5 (4851.8-5783) | 583.3 (429.1-770.7) | 6827.3 (4888.8-9228.2) | 399.6 (270.8-557) | 5043.9 (4620.3-5465.3) | 487.4 (357-643.1) | 5682.1 (3975.6-7836.9) | 337 (219.5-485.3) |
| North Macedonia | 6147.1 (4990.6-7440.8) | 1406.9 (1073.7-1782) | 21449.6 (17074.1-26083.5) | 1505.8 (1207-1823.5) | 4873.4 (4031.2-5847.2) | 1489.4 (1175.6-1849.1) | 23474.4 (19201.6-28118.1) | 1808.4 (1483.7-2153.3) |
| Northern Mariana Islands | 4722.7 (4353.8-5132.2) | 518.1 (375.8-682.5) | 4805.6 (3479.4-6420.5) | 270.4 (182.6-377.4) | 3778.8 (3483.5-4083.6) | 360.8 (258.9-479.6) | 4056.1 (2972.3-5330.1) | 246 (170.2-335.9) |
| Norway | 5699.6 (4806.5-6631.4) | 572.7 (405.3-769.6) | 2283.3 (1970.7-2608.4) | 117.9 (101.7-128.7) | 3357.3 (2858.4-3913.9) | 363.2 (259.5-492.7) | 1724.1 (1426.9-1978.3) | 107 (84.4-120.6) |
| Oman | 4703.4 (4238.4-5222.3) | 740.8 (555-957.9) | 8642.1 (6274.6-11632.5) | 569.4 (407.7-773.8) | 3174.2 (2843.5-3519) | 553.9 (419.3-712.3) | 5616 (4015.3-7541.6) | 331.4 (229-454.9) |
| Pakistan | 3621.7 (2906.7-4429.3) | 462.4 (320.5-635.5) | 5911.1 (4170.4-8302.1) | 343.4 (236.3-487.9) | 3162.8 (2534.4-3888.5) | 494.2 (344.6-684.6) | 6518.7 (4601.3-9126.9) | 395.6 (271.6-557.5) |
| Palau | 5977 (5461.8-6509.2) | 645.7 (473.6-853.3) | 6858.9 (4975.4-9264.9) | 372.3 (255.2-518.8) | 5630.6 (5140.7-6111.5) | 554.6 (408-740.5) | 7760.5 (5552.3-10532.8) | 503 (345.5-698.2) |
| Palestine | 3230.1 (2843.3-3641.7) | 921.2 (723.9-1148.4) | 10692.2 (8050.1-13778.1) | 754 (564.7-970.2) | 2822.7 (2496.4-3180.3) | 710.6 (546.5-900.4) | 9597.4 (7345.3-12060) | 655.8 (496.8-827.1) |
| Panama | 2932.9 (2681.2-3204.6) | 377.7 (279-495.5) | 3115 (2388.7-3834.6) | 208.8 (157.5-258.2) | 2649 (2430.3-2876.9) | 303 (221.9-400.1) | 2254.8 (1759.6-2699.2) | 151.3 (113-184) |
| Papua New Guinea | 3391.5 (3079.8-3715.1) | 397.6 (291.5-530.7) | 4260.8 (2669.1-6680.5) | 246.8 (142.5-403.3) | 2848.2 (2585.5-3127.2) | 315.4 (225.5-423.5) | 6501.2 (4312.6-9587.6) | 406.8 (261.3-609.9) |
| Paraguay | 3982.9 (3614.9-4371.1) | 516.4 (377.1-687.5) | 5560.6 (3951.3-7437.6) | 356 (251-477.2) | 2660.1 (2406.6-2930.7) | 335.2 (248.5-443.2) | 3758.9 (2667.7-5021.7) | 255 (175.1-343.4) |
| Peru | 2414.3 (2211.8-2617) | 236.8 (170.5-314.9) | 1973.5 (1316.4-2794.7) | 119 (75.7-173.9) | 2245.5 (2057.8-2441.9) | 202.5 (148.6-273.7) | 1678.4 (1160.4-2343.8) | 103.9 (67.1-150.6) |
| Philippines | 3937.6 (3226-4725) | 488.6 (340.8-669) | 6628 (5482.8-7918) | 360.3 (293.1-434.9) | 3976.2 (3328.4-4677.4) | 454.9 (319.7-623.3) | 5427.8 (4494.4-6519.2) | 334 (273.4-401.3) |
| Poland | 3967.8 (3258.9-4755.9) | 554.5 (388.3-755.4) | 6143.7 (5444-6807.9) | 363.7 (318.6-402.4) | 2734 (2287.1-3248) | 459.4 (324.2-624.2) | 4363 (3744.7-4846.2) | 302.7 (252.5-338.6) |
| Portugal | 2752.6 (2484.4-3037.8) | 338.4 (260.6-433.2) | 3359.4 (2890-3799.2) | 235.9 (199-268.4) | 1661.4 (1488.6-1843.9) | 270.2 (210.9-335.1) | 2582.6 (2077.9-2969.3) | 205.1 (159.1-236.8) |
| Puerto Rico | 2222.6 (2042.5-2413.6) | 214.4 (154.2-287) | 1315.4 (1059.7-1593.4) | 78.9 (61.5-96.9) | 2344.5 (2154.4-2528.8) | 189 (138.4-253.3) | 1077.5 (871.2-1292.9) | 65 (49.9-79.5) |
| Qatar | 3729.9 (3403.5-4085.2) | 409.5 (298.6-541.5) | 4436.9 (3116.3-6087.7) | 299.1 (203.8-417.9) | 2822.8 (2528.6-3133.8) | 470.9 (356.8-609.5) | 3876 (2703.4-5273.4) | 255.2 (171.8-355.7) |
| Republic of Korea | 6178.2 (5755-6609.1) | 486.7 (358.2-635) | 4245.8 (3410.7-5086.7) | 249.6 (190.7-310.2) | 3356.2 (3109.8-3620.6) | 328.5 (245.4-430.9) | 2543.2 (1899.1-3195.3) | 168.2 (115.9-218.5) |
| Republic of Moldova | 4771.8 (4263.3-5319.9) | 762.2 (565.8-987.4) | 8959 (7779.5-10225.1) | 465.2 (399.9-534.7) | 2987.4 (2689.8-3316) | 550.4 (406.2-712.5) | 5941.5 (5142.4-6768.9) | 340 (288.4-390.5) |
| Romania | 6214.9 (5663.3-6798.1) | 922.6 (708.4-1173) | 12458.6 (10814.2-14136.7) | 776.3 (667.7-885.7) | 4147.1 (3789.4-4538.8) | 739.4 (564-932.8) | 9133.8 (7844.1-10313.9) | 636.5 (539.3-720.9) |
| Russian Federation | 4748.4 (3959.8-5610.5) | 898.3 (629.7-1222.6) | 13676.2 (12183.5-15046.5) | 807 (713-887.4) | 3894 (3327.3-4505.9) | 819.9 (585.2-1111.2) | 10286.7 (9097.3-11196) | 702.9 (612.6-768.8) |
| Rwanda | 4396 (3955.8-4870.1) | 530.5 (376.6-714.2) | 6302.9 (3845.1-9795.2) | 391.6 (229.3-627.8) | 5368.4 (4854.1-5890) | 643.3 (461.1-866.7) | 6090.9 (3703.4-9094.2) | 367.4 (203.2-568.2) |
| Saint Kitts and Nevis | 3848.3 (3444.6-4273.9) | 588.5 (442.8-764.9) | 8293.1 (6650.4-10131.4) | 531.8 (424.7-647.3) | 3438.5 (3102-3836.5) | 554.3 (422.3-708.3) | 7907.8 (6628.2-9082.6) | 568.9 (476-647.3) |
| Saint Lucia | 3239.5 (2931.3-3565.9) | 427.1 (318.8-553.3) | 5751.1 (4613.5-6940.4) | 415.5 (333.5-500.1) | 2931.5 (2661.4-3224.5) | 408.5 (310-534.2) | 5397.8 (4445.8-6395.2) | 401.5 (328.8-477) |
| Saint Vincent and the Grenadines | 2875.2 (2591.7-3166.9) | 407.3 (303.2-528) | 5238.6 (4458.9-6078) | 369.3 (315.9-426.1) | 2903.3 (2625.1-3200.8) | 416.1 (313.7-542.4) | 5285.4 (4454.4-6154) | 382.2 (320.9-444.7) |
| Samoa | 4888.1 (4480.5-5320.1) | 521.5 (382.5-696.2) | 5726.3 (3967.5-7966.1) | 328.1 (212-472.4) | 5151.6 (4723.7-5591) | 498.4 (362-657.3) | 5659.4 (3948-7747.4) | 331.2 (213.7-475.4) |
| San Marino | 3647.9 (3321.6-3996.8) | 351.2 (252.6-473.8) | 2011.3 (1414.7-2773) | 112.5 (70.4-164.7) | 2491.1 (2241.1-2748.9) | 280.4 (203.6-382.8) | 1770.8 (1174.2-2481.2) | 116.3 (68.4-173.1) |
| Sao Tome and Principe | 5668.9 (5184.4-6165.3) | 542.7 (392.2-732) | 10316.8 (7351.7-14164.3) | 611.9 (430.3-845.6) | 7522 (6941.7-8136.1) | 711.6 (525.7-943.9) | 8938.2 (6571.5-11761.2) | 545.5 (388.5-734.2) |
| Saudi Arabia | 4062.4 (3634.9-4512.8) | 594.7 (445.6-770.1) | 7654.6 (5659.7-10102.5) | 479.9 (346.3-639.7) | 2521.8 (2200.6-2904.1) | 459.3 (354.5-587.6) | 7860.6 (5585.8-10660.9) | 520.9 (368.6-705.5) |
| Senegal | 4757.4 (4361-5156.1) | 455.5 (329-606.9) | 11667.4 (8052.7-16136.6) | 711.6 (483.1-990.9) | 5751.6 (5284.9-6234.8) | 534.8 (390.1-715.8) | 8146.5 (5230.1-11919) | 509.5 (313.7-760.8) |
| Serbia | 5419.6 (4606.5-6308.8) | 954.5 (722.4-1221.6) | 15058.6 (11840.6-18652.3) | 995.4 (779.1-1244.2) | 3747.6 (3103.4-4454) | 1033 (820.2-1270.6) | 13820.3 (11134.5-16724.7) | 977.3 (782-1183.6) |
| Seychelles | 4693.4 (4289.1-5132) | 508.3 (376-667.6) | 6382.5 (4741.4-8204.2) | 368.2 (262.4-482.2) | 3784 (3466.3-4118) | 437.7 (326.1-577.8) | 4596 (3439.5-5829.8) | 288.6 (208.3-373.2) |
| Sierra Leone | 4803.1 (4365.7-5260) | 482.9 (351.8-640) | 11219.8 (7408.4-16204.3) | 660.9 (435.6-952.2) | 5838.9 (5340.3-6363.1) | 548.1 (402.2-728) | 9031.8 (5863.1-13418.1) | 557.6 (352.1-834.8) |
| Singapore | 4323.4 (4020.9-4642.8) | 292.6 (210.8-397.1) | 1417.5 (1167.7-1667.7) | 50.5 (40.9-59.6) | 2786.2 (2577.4-3000.8) | 243.3 (179.4-326) | 1208.9 (980.9-1420.3) | 55.9 (43-66.7) |
| Slovakia | 7156.2 (6549.9-7742.3) | 791.6 (589.2-1034) | 7754.8 (6153.1-9608.4) | 425.4 (325.6-539.3) | 5067.1 (4677.9-5469.2) | 631.1 (471.6-808.2) | 5575.9 (4355.3-6873.9) | 338.1 (253.1-429.6) |
| Slovenia | 3314.3 (3075.4-3553.3) | 421.9 (329-528.6) | 3857.2 (3298.9-4416.3) | 249.2 (209.5-286.1) | 2157.3 (1972.1-2352.5) | 320.5 (244.7-409.8) | 2393.9 (1937.6-2797.2) | 166.4 (130.8-195.5) |
| Solomon Islands | 5132.6 (4595.4-5693.7) | 683.4 (494-909.3) | 5847.6 (3808.1-9061.1) | 328 (195.8-533.5) | 6059.8 (5428.4-6687.7) | 836.7 (616.1-1106.7) | 8371.2 (5727.2-11989.7) | 507.3 (329.3-752.5) |
| Somalia | 3966.8 (3492.1-4461.7) | 494.3 (356.7-665) | 5417.3 (2776.3-9743.7) | 305.1 (144.9-566.4) | 4926.9 (4387.7-5439.4) | 570.8 (419.6-766.3) | 5839.4 (3059.3-9471.6) | 334.9 (157.7-564.2) |
| South Africa | 5185.1 (4300.4-6210.5) | 653.4 (454.9-897.2) | 7101.8 (6215.1-8088.5) | 424 (369.8-484.2) | 6917.2 (5835-8068.1) | 807.8 (565.5-1109.6) | 7173.8 (6159.9-8183.1) | 447 (375.1-513.2) |
| South Sudan | 4549.6 (4116.1-4992.7) | 531 (384.7-713.3) | 5965.8 (3833.9-9227.8) | 347.6 (214-544.6) | 5377.8 (4897.1-5872.7) | 595.3 (427-801.3) | 5742.8 (3428.7-8722.1) | 329.9 (180.4-516.5) |
| Spain | 4941.8 (4635-5247) | 413.7 (343.1-490.6) | 2187.9 (1849.5-2516.5) | 116.2 (95.3-134.8) | 2310.5 (2159.2-2467.9) | 228.6 (187.3-272.9) | 1468.7 (1155.9-1719.4) | 99.4 (73.6-117.8) |
| Sri Lanka | 4320.9 (3925.7-4726.5) | 552.5 (413.5-722.7) | 9112.3 (6243.4-12342.4) | 621.3 (422.1-838.7) | 3687.1 (3349.5-4030.3) | 503 (373.9-661.8) | 7148.6 (5112.6-9539.4) | 501.7 (350.2-678.2) |
| Sudan | 4448.3 (3941.8-4987.1) | 689.4 (511.6-898.4) | 9283 (6131.1-13446.2) | 567.9 (369.2-829.2) | 3134.2 (2740.4-3550) | 601.9 (449.7-785.3) | 10171.6 (6236.7-15264.1) | 635.1 (384.3-945.5) |
| Suriname | 3479.8 (3118.7-3860) | 488.1 (355.2-644.8) | 5308.6 (3545.7-7467.8) | 319.4 (205.6-457.1) | 2905.1 (2626.9-3199.1) | 408.7 (306.4-527.4) | 4676.4 (3272.9-6415.7) | 311.4 (212-433.3) |
| Sweden | 4595.4 (3792.8-5466.9) | 452 (313.4-623.3) | 2248.6 (1883.1-2650.6) | 129.2 (105.9-152.7) | 2760.9 (2298.2-3285.5) | 314.1 (216.6-438.2) | 1755.7 (1398-2069.5) | 117.2 (88.2-139.1) |
| Switzerland | 3235.8 (2974.5-3512.3) | 284.9 (211.6-374) | 1670.9 (1414.3-1922.5) | 98.3 (80.3-114.1) | 1961.8 (1803-2128.1) | 211.9 (159.3-275.2) | 1226.4 (954.8-1450.4) | 83.2 (60.4-99.5) |
| Syrian Arab Republic | 3425.7 (3063.7-3816) | 479 (358.5-624.7) | 7496.6 (5385.2-10158.1) | 471.5 (337.1-637.3) | 2464.2 (2171-2792.6) | 548.4 (422.1-699.2) | 8368.4 (6094.2-11205.1) | 595.6 (431-793.6) |
| Taiwan (Province of China) | 5616.1 (5235.7-6005.5) | 597 (434-798.5) | 2975.6 (2525.5-3435.4) | 131.7 (110.4-154.9) | 4321.5 (3991.8-4658.7) | 417.7 (297-566.2) | 2012.9 (1641.2-2371.8) | 89.6 (69.4-105.7) |
| Tajikistan | 3796.4 (3298.6-4329) | 880.3 (654.1-1141.2) | 9469.9 (6776.1-12554.1) | 569.7 (399.1-762.6) | 4128.2 (3655.3-4628.8) | 788.2 (578.1-1034.1) | 8897.3 (6633.2-11585) | 530.9 (385.1-706.4) |
| Thailand | 3874.4 (3571.8-4194.4) | 361.3 (262.5-481.6) | 3988.6 (2857.1-5331.7) | 210 (139.5-295.3) | 3512.9 (3247.6-3804.8) | 332.6 (240.3-450) | 3260.8 (2336.9-4306.8) | 182.3 (117.9-255.8) |
| Timor-Leste | 4072.4 (3629-4511.6) | 612.3 (450.9-803.4) | 9300.9 (5486.4-14152.8) | 573.7 (325.9-883.4) | 3520.2 (3156.7-3909.2) | 558.2 (414.8-736.5) | 7815.1 (5325.8-10972.8) | 501.2 (331.5-716.3) |
| Togo | 5131.4 (4661-5625.2) | 531.2 (387.6-710.4) | 13851.5 (9617.4-19246.6) | 818.8 (564.7-1138.4) | 5274.9 (4833.5-5739) | 520 (376.1-698.1) | 8000.5 (4964.3-12030.9) | 502.6 (300.5-764) |
| Tokelau | 3679.3 (3373.3-3984.4) | 364.5 (259.9-490.3) | 5150.1 (3540.2-7120.9) | 308.2 (197.4-440) | 4686.1 (4301.7-5063.7) | 448.6 (329.4-593.7) | 5731.6 (3901.9-7986.4) | 340.1 (214.7-495) |
| Tonga | 3920.2 (3585.1-4269) | 442 (322.8-580.5) | 4107 (2900.4-5621.9) | 227.4 (147.9-328.1) | 3818 (3494.5-4153.3) | 398.6 (290.1-535.1) | 3888.1 (2746.6-5352.9) | 230.4 (147.4-334) |
| Trinidad and Tobago | 3722.8 (3373.7-4094.3) | 484.5 (356.8-641.7) | 5785.6 (4442.3-7364.9) | 367.2 (279.5-465) | 2811.4 (2548.9-3086.5) | 353.2 (257.9-463.9) | 4154.8 (3268.1-5139.5) | 289.6 (223.2-360.3) |
| Tunisia | 3944 (3524.2-4375.8) | 589.6 (434.3-772.7) | 7905.9 (5160.4-11488.7) | 524.2 (334.2-769.3) | 2710 (2412.3-3049.1) | 460.1 (338.5-615) | 6112.2 (3618.2-9136.7) | 404 (228.1-611.2) |
| Turkey | 3970.9 (3633.5-4332.2) | 465.3 (341.6-617.5) | 5644.7 (4073.6-7555.3) | 359.9 (251-490.3) | 3289.1 (2982.7-3624.1) | 449.7 (324.1-606.6) | 5238.5 (3880.7-6857.6) | 364.6 (262.4-486.2) |
| Turkmenistan | 5508.4 (4857.2-6183.8) | 826.9 (602.6-1087.3) | 14434.8 (10570.7-18076) | 779.9 (569.7-974.8) | 5573 (4993.7-6183) | 725.9 (531-959.6) | 10823.8 (8437.5-13596.9) | 577.5 (440.6-735.7) |
| Tuvalu | 4429.2 (4033.9-4861.9) | 530.6 (385.4-699) | 7021.6 (4870.6-9808.2) | 415.3 (272-591) | 4626.4 (4225.5-5063.9) | 526.5 (388-695) | 6929.2 (4783.5-9580.1) | 420.6 (276.3-598.7) |
| Uganda | 5320.4 (4815.6-5851.3) | 592.1 (427.7-796.3) | 5698.2 (3759.2-8321.4) | 334.2 (211.1-503.3) | 6462.4 (5935.4-6998.2) | 708.2 (505.6-957.2) | 5053.7 (3373-7304.1) | 283.4 (169.5-434.8) |
| Ukraine | 5208.9 (4140.3-6431.3) | 883.4 (612.5-1209.8) | 11797.9 (8586.6-15712.1) | 700.8 (507.3-931) | 4002.5 (3287.3-4819.5) | 729.6 (511.4-1006.2) | 8444.3 (6109.9-11355.2) | 547.7 (387.5-742.9) |
| United Arab Emirates | 5937.4 (5334.1-6575.9) | 786.4 (596-1018.3) | 4946.1 (3622.1-6575.1) | 298.7 (206.1-409.5) | 4391.3 (3926.6-4877.2) | 755.6 (580-978.5) | 21296.8 (15349.6-28132.4) | 1430.1 (1024.7-1894.3) |
| United Kingdom | 3620.9 (3195.8-4095.2) | 353.6 (267.8-459.4) | 2027.2 (1806.2-2225.6) | 121.5 (108-129.3) | 2775.6 (2451.5-3116.7) | 298.5 (227.5-386.9) | 1739.8 (1463.2-1953.9) | 115.5 (93.2-127.6) |
| United Republic of Tanzania | 5980.6 (5416.5-6574.9) | 665.5 (480.6-888.8) | 7706.5 (4851.2-11346.4) | 466.4 (283.2-699.6) | 6363.5 (5819.9-6916.6) | 725.8 (527.6-969.7) | 6264.3 (4082.3-9149.2) | 364.3 (213.9-557.8) |
| United States of America | 5181.3 (4530.7-5867.4) | 318.2 (220.8-439.2) | 2377.6 (2086.2-2641.5) | 128.4 (110.5-139) | 4287 (3733.5-4874) | 288.5 (201.6-398.2) | 2263.6 (1887.1-2559.7) | 134.9 (105.5-150.8) |
| United States Virgin Islands | 2778.3 (2504.6-3078.1) | 378.8 (280.7-500.9) | 2846 (1944.7-3996.9) | 198.8 (133.1-284.9) | 2337.4 (2123-2561.7) | 293.7 (217.4-383.2) | 1802.7 (1245.9-2458.9) | 123.9 (81.2-172.8) |
| Uruguay | 4372.1 (4018.8-4749.9) | 424.7 (310.9-557.3) | 4658.4 (4051.7-5267.9) | 283 (242.9-322.1) | 3162 (2903.9-3441.9) | 361.6 (271.5-467.2) | 3438.5 (2896.6-3914.9) | 234.4 (191.8-269.2) |
| Uzbekistan | 5289 (4736.2-5902.6) | 898.6 (678-1160.2) | 9900.5 (8361.1-11656.9) | 558.3 (467.8-661) | 5069.7 (4596.1-5581.4) | 797.5 (596.1-1046.2) | 6994.1 (6017.1-8086.2) | 387.4 (325.3-451.8) |
| Vanuatu | 5591.3 (5068.8-6135.7) | 651.1 (473.9-855.8) | 7583.2 (5232.4-10495.1) | 433.8 (284.6-620) | 5764.6 (5240.2-6296.1) | 611.7 (455.7-818.7) | 6670.3 (4531.4-9394.3) | 389 (243.5-572.8) |
| Venezuela (Bolivarian Republic of) | 2898.4 (2636.6-3185.3) | 376.7 (278.1-494.2) | 3739.5 (2831.9-4849.7) | 242.9 (183-316) | 2624.4 (2399.4-2860.8) | 309.3 (231.7-410) | 2810.3 (2092.2-3609.1) | 191.2 (137.7-247.9) |
| Viet Nam | 5998.7 (5482.8-6562.4) | 873.3 (689-1088.1) | 17005.5 (12091.9-22376.5) | 1063.6 (750.1-1401.7) | 3554.9 (3238.6-3873.8) | 521.7 (404.8-659) | 7983.4 (5703.5-10589.9) | 546.6 (381.1-732.1) |
| Yemen | 3634.3 (3185.8-4089.9) | 674.2 (499.5-872.3) | 13458 (8886.6-19470.8) | 842.6 (554-1221.8) | 2742 (2379.4-3122.6) | 585.6 (445.9-759.4) | 12041.1 (7570.8-18063.4) | 759.7 (473.4-1137.7) |
| Zambia | 5445.3 (4948.5-5986.9) | 670.1 (489.8-893.3) | 8110.6 (5384.8-11817.3) | 487.3 (314.3-718.9) | 7188.8 (6531.2-7866.8) | 891.2 (636.9-1178.9) | 8478.9 (5383-12287.4) | 508.2 (302.1-765.8) |
| Zimbabwe | 4277.2 (3856-4743.9) | 573.5 (419.5-754.2) | 8959.7 (6273.8-12298.1) | 517.8 (352.9-716.8) | 6528 (5917.2-7138.8) | 824.9 (607.9-1088.7) | 9324.3 (6569.3-12761.8) | 561 (385.8-777.9) |

ASPR = age-standardized prevalence rate; ASIR = age-standardized incidence rate;ASDR = Age-standardized DALYs rate; ASMR = Age-standardized mortality rate; DALYs =Disability-Adjusted Life Years; AAPC = average annual percentage change; CI=confidence interval; SDI=sociodemographic index; UI=uncertainty interval.
