# Supplementary material for: Global burden of ischemic stroke in adults aged 60 years and older from 1990 to 2021: Population-based study
Source: PLoS One. 2025 May 5;20(5):e0322606. doi: 10.1371/journal.pone.0322606 (PMC12052125; doi:10.1371/journal.pone.0322606)
Supplement: S5 Table — (DOCX) [file pone.0322606.s016.docx]

| Table 5. Global and regional differences in morbidity, mortality, DALYs and demographic and epidemiological changes. | | | | | | | | | | | | | | | | |
| --- | --- | --- | --- | --- | --- | --- | --- | --- | --- | --- | --- | --- | --- | --- | --- | --- |
|  | Prevalence | | | | Incidence | | | | Deaths | | | | DALYs | | | |
| location | Overll difference | Aging | Population change | Epidemiological change | Overll difference | Aging | Population change | Epidemiological change | Overll difference | Aging | Population change | Epidemiological change | Overll difference | Aging | Population change | Epidemiological change |
| Global | 25326161.76(125.38%) | 1120140.75 (5.55%) | 25140106.73 (124.46%) | -934085.72 (-4.62%) | 2835031.35(98.71%) | 295633.56 (10.29%) | 3375788.37 (117.54%) | -836390.58 (-29.12%) | 1228747.23(56.82%) | 432532.79 (20%) | 2311838.98 (106.9%) | -1515624.53 (-70.08%) | 21161869.7(56.19%) | 4128173.99 (10.96%) | 39945419.76 (106.06%) | -22911724.05 (-60.84%) |
| High SDI | 4930795.89(68.31%) | 461305.38 (6.39%) | 6081896.43 (84.26%) | -1612405.92 (-22.34%) | 184326(20.36%) | 91745.33 (10.13%) | 664889.86 (73.43%) | -572309.2 (-63.2%) | -82705(-14.27%) | 136769.08 (23.61%) | 386302.85 (66.68%) | -605776.93 (-104.56%) | -1167140.53(-12.7%) | 1265818.07 (13.78%) | 6081079.7 (66.19%) | -8514038.31 (-92.67%) |
| High-middle SDI | 6621320.25(119.57%) | 280100.27 (5.06%) | 5951162.78 (107.47%) | 390057.2 (7.04%) | 819345.21(85.05%) | 124051.35 (12.88%) | 958544.03 (99.5%) | -263250.16 (-27.33%) | 267006.79(31.98%) | 200176.83 (23.98%) | 727278.63 (87.11%) | -660448.67 (-79.11%) | 4108498.93(28.91%) | 1888615.2 (13.29%) | 12152082.06 (85.51%) | -9932198.33 (-69.89%) |
| Middle SDI | 9822721.06(227.72%) | 402607.63 (9.33%) | 8271092.53 (191.75%) | 1149020.91 (26.64%) | 1332533.48(225.11%) | 99713.08 (16.84%) | 1132254.34 (191.27%) | 100566.05 (16.99%) | 661353.76(152.29%) | 132399.09 (30.49%) | 734555.36 (169.15%) | -205600.68 (-47.34%) | 11436018.24(138.74%) | 1362814.48 (16.53%) | 13542317.09 (164.29%) | -3469113.34 (-42.09%) |
| Low-middle SDI | 3005477.57(140.37%) | 69175.92 (3.23%) | 3093442.72 (144.48%) | -157141.07 (-7.34%) | 390536.34(132.61%) | 25331.28 (8.6%) | 419002.72 (142.28%) | -53797.65 (-18.27%) | 303128.64(130.43%) | 34279.56 (14.75%) | 329520.95 (141.78%) | -60671.87 (-26.1%) | 5342312.11(121.29%) | 359692.64 (8.17%) | 6119618.04 (138.94%) | -1136998.57 (-25.81%) |
| Low SDI | 932803.84(96.83%) | 28569.18 (2.97%) | 1104837.69 (114.68%) | -200603.03 (-20.82%) | 106990.7(95%) | 6677.18 (5.93%) | 128626.8 (114.21%) | -28313.28 (-25.14%) | 79863.62(102.4%) | 9370.95 (12.01%) | 90741.78 (116.34%) | -20249.11 (-25.96%) | 1442469.66(92.83%) | 97454.67 (6.27%) | 1765925.54 (113.64%) | -420910.55 (-27.09%) |
| Andean Latin America | 108081.22(162.53%) | 2849.8 (4.29%) | 126153.36 (189.71%) | -20921.94 (-31.46%) | 9680.62(124.68%) | 671.84 (8.65%) | 13773.5 (177.39%) | -4764.71 (-61.36%) | 4267.93(85.2%) | 655.36 (13.08%) | 8260.39 (164.9%) | -4647.82 (-92.78%) | 66854.67(80.3%) | 6666.36 (8.01%) | 135521.87 (162.78%) | -75333.56 (-90.49%) |
| Australasia | 98345.22(74.59%) | 10979.29 (8.33%) | 146714.54 (111.27%) | -59348.61 (-45.01%) | 6037.47(35.67%) | 2579.23 (15.24%) | 17126.49 (101.18%) | -13668.25 (-80.75%) | -413.53(-4.25%) | 3481.78 (35.76%) | 9068.64 (93.14%) | -12963.96 (-133.15%) | -13705.05(-9.07%) | 33190.68 (21.97%) | 136296.45 (90.21%) | -183192.19 (-121.24%) |
| Caribbean | 97438.03(100.57%) | 1035.15 (1.07%) | 103787.55 (107.12%) | -7384.67 (-7.62%) | 12212.63(95.35%) | 1527.07 (11.92%) | 13608.99 (106.25%) | -2923.43 (-22.82%) | 7599.38(68.1%) | 2368.57 (21.23%) | 11184.18 (100.22%) | -5953.37 (-53.35%) | 112839.53(62.2%) | 17994.5 (9.92%) | 177609.77 (97.91%) | -82764.74 (-45.62%) |
| Central Asia | 176885.64(63.59%) | -5860.92 (-2.11%) | 200328.99 (72.02%) | -17582.44 (-6.32%) | 24059.82(59.68%) | -2865.88 (-7.11%) | 28713.1 (71.22%) | -1787.4 (-4.43%) | 11133.61(37.36%) | -3857.17 (-12.94%) | 19751.97 (66.28%) | -4761.19 (-15.98%) | 215228.07(39.47%) | -43161.18 (-7.92%) | 364089.49 (66.77%) | -105700.25 (-19.38%) |
| Central Europe | 317563.53(32.99%) | 60963.36 (6.33%) | 475251.98 (49.37%) | -218651.81 (-22.71%) | 33429.21(19.29%) | 26173.14 (15.11%) | 82023.05 (47.34%) | -74766.98 (-43.15%) | -17494.33(-10.03%) | 42703.91 (24.48%) | 75275.82 (43.15%) | -135474.06 (-77.66%) | -451236.25(-15.76%) | 428398.25 (14.97%) | 1193694.89 (41.7%) | -2073329.38 (-72.44%) |
| Central Latin America | 491994.92(151.45%) | 18550.98 (5.71%) | 642210.19 (197.7%) | -168766.26 (-51.95%) | 47669.9(120.6%) | 4724.84 (11.95%) | 74173.03 (187.65%) | -31227.97 (-79%) | 18486.74(82.85%) | 4439.64 (19.9%) | 39413.72 (176.63%) | -25366.63 (-113.68%) | 297600.41(80.6%) | 39704.81 (10.75%) | 644539.44 (174.56%) | -386643.83 (-104.71%) |
| Central Sub-Saharan Africa | 141125.41(111.57%) | 2962.46 (2.34%) | 160572.37 (126.94%) | -22409.42 (-17.72%) | 15656.8(109.88%) | 554.79 (3.89%) | 18014.68 (126.42%) | -2912.68 (-20.44%) | 9643.01(126.87%) | 1087.6 (14.31%) | 9974.4 (131.23%) | -1418.99 (-18.67%) | 178146.32(111.02%) | 8676 (5.41%) | 203314.26 (126.7%) | -33843.94 (-21.09%) |
| East Asia | 11851935.03(306.6%) | 789144.83 (20.41%) | 8115535.5 (209.94%) | 2947254.7 (76.24%) | 1714503.83(308.98%) | 149035.63 (26.86%) | 1171307.08 (211.09%) | 394161.11 (71.03%) | 741611.2(188.2%) | 197631.49 (50.15%) | 688210.12 (174.65%) | -144230.41 (-36.6%) | 12633226.91(161.8%) | 2130869.27 (27.29%) | 12964834.18 (166.05%) | -2462476.54 (-31.54%) |
| Eastern Europe | 346820.03(20.23%) | -2733.31 (-0.16%) | 515225.92 (30.05%) | -165672.59 (-9.66%) | 6812.34(1.77%) | 35557.6 (9.22%) | 108077.67 (28.01%) | -136822.93 (-35.46%) | -68106.9(-17.73%) | 54305.43 (14.13%) | 99689.4 (25.95%) | -222101.74 (-57.81%) | -1388844.94(-21.58%) | 445883.14 (6.93%) | 1627141.97 (25.28%) | -3461870.04 (-53.79%) |
| Eastern Sub-Saharan Africa | 452823.94(108.41%) | 10058.14 (2.41%) | 486815.62 (116.55%) | -44049.82 (-10.55%) | 50523.6(113.28%) | 1740.8 (3.9%) | 52557.98 (117.84%) | -3775.18 (-8.46%) | 25134(110.1%) | 2535.23 (11.11%) | 26738.98 (117.13%) | -4140.21 (-18.14%) | 473745.05(102.12%) | 21741.91 (4.69%) | 532938.91 (114.88%) | -80935.77 (-17.45%) |
| High-income Asia Pacific | 1279015.4(91.53%) | 293385.54 (21%) | 1652314.69 (118.24%) | -666684.83 (-47.71%) | 63708.32(38.17%) | 53119.21 (31.82%) | 177104.49 (106.1%) | -166515.39 (-99.76%) | 9606.8(9.41%) | 81308.25 (79.67%) | 109039.94 (106.85%) | -180741.39 (-177.11%) | 38940.92(2.31%) | 811420.66 (48.07%) | 1684008.59 (99.76%) | -2456488.34 (-145.52%) |
| High-income North America | 1794645.63(75.56%) | -24853.82 (-1.05%) | 2070922.82 (87.2%) | -251423.37 (-10.59%) | 27105.86(10.86%) | 183.11 (0.07%) | 179091.5 (71.76%) | -152168.75 (-60.97%) | 16129.14(14.95%) | 6936.88 (6.43%) | 78589.46 (72.83%) | -69397.21 (-64.31%) | 309741.02(17.33%) | 36551.99 (2.04%) | 1310059.46 (73.29%) | -1036870.42 (-58.01%) |
| North Africa and Middle East | 1217469.26(166.1%) | 10696.87 (1.46%) | 1232979.82 (168.21%) | -26207.43 (-3.58%) | 156896.44(146.13%) | 7128.91 (6.64%) | 174177.62 (162.22%) | -24410.09 (-22.73%) | 111250.48(97.54%) | 13311.78 (11.67%) | 168058.76 (147.35%) | -70120.05 (-61.48%) | 1867990.22(90.87%) | 128617.76 (6.26%) | 2980202.35 (144.97%) | -1240829.89 (-60.36%) |
| Oceania | 14959.26(137.04%) | 644.86 (5.91%) | 15725.35 (144.06%) | -1410.95 (-12.93%) | 1637.45(136.06%) | 90.27 (7.5%) | 1731.87 (143.91%) | -184.69 (-15.35%) | 931.99(132.57%) | 140.47 (19.98%) | 1006.27 (143.14%) | -214.75 (-30.55%) | 17980.79(120.27%) | 1494.19 (9.99%) | 20812.17 (139.21%) | -4325.57 (-28.93%) |
| South Asia | 2337397.25(156.14%) | 63459.21 (4.24%) | 2540923.96 (169.74%) | -266985.91 (-17.84%) | 305283.53(137.87%) | 28395.26 (12.82%) | 363497.14 (164.16%) | -86608.86 (-39.11%) | 257059.39(164.5%) | 32565 (20.84%) | 269781.18 (172.64%) | -45286.79 (-28.98%) | 4497580.51(147.16%) | 358084.53 (11.72%) | 5103760.2 (167%) | -964264.22 (-31.55%) |
| Southeast Asia | 2240950.84(176.97%) | 4216.19 (0.33%) | 2213652.28 (174.81%) | 23082.37 (1.82%) | 275062.43(177.16%) | 3865.3 (2.49%) | 271648.67 (174.96%) | -451.53 (-0.29%) | 195699.3(164.49%) | 7333.04 (6.16%) | 203373.03 (170.94%) | -15006.78 (-12.61%) | 3549450.57(159.05%) | 55095.94 (2.47%) | 3775332.25 (169.17%) | -280977.62 (-12.59%) |
| Southern Latin America | 114962.79(43.24%) | 11690.6 (4.4%) | 209838.92 (78.93%) | -106566.73 (-40.08%) | 7458.87(24.17%) | 2995.42 (9.7%) | 23055.71 (74.7%) | -18592.26 (-60.24%) | -3269.18(-14.49%) | 4782.34 (21.19%) | 15140.7 (67.1%) | -23192.22 (-102.78%) | -60326.25(-15.99%) | 45987.36 (12.19%) | 248597.3 (65.89%) | -354910.91 (-94.07%) |
| Southern Sub-Saharan Africa | 185079.23(92.82%) | -6703.65 (-3.36%) | 219505.95 (110.09%) | -27723.07 (-13.9%) | 24903.75(122.13%) | -1040.78 (-5.1%) | 24076.21 (118.07%) | 1868.32 (9.16%) | 14559.52(159.87%) | -1145.98 (-12.58%) | 11701.78 (128.49%) | 4003.72 (43.96%) | 265466.26(153.81%) | -12010.34 (-6.96%) | 218630.44 (126.68%) | 58846.16 (34.1%) |
| Tropical Latin America | 562020.69(122.54%) | 20543.52 (4.48%) | 796835.86 (173.74%) | -255358.69 (-55.68%) | 58982.95(92.17%) | 8995.95 (14.06%) | 105670.05 (165.13%) | -55683.05 (-87.01%) | 17433.18(34.43%) | 13376.94 (26.42%) | 75452.62 (149.02%) | -71396.38 (-141.01%) | 238889.65(27.31%) | 119618.83 (13.68%) | 1263711.05 (144.48%) | -1144440.23 (-130.85%) |
| Western Europe | 1005793.06(28.68%) | 256826.76 (7.32%) | 1768159.87 (50.42%) | -1019193.57 (-29.07%) | -51185.64(-9.93%) | 65279.71 (12.67%) | 227238.6 (44.09%) | -343703.95 (-66.69%) | -161009.74(-42.96%) | 102007.74 (27.22%) | 148614.43 (39.66%) | -411631.92 (-109.84%) | -2394098.51(-43.51%) | 957133.64 (17.39%) | 2134868.21 (38.8%) | -5486100.36 (-99.7%) |
| Western Sub-Saharan Africa | 490855.41(97.45%) | -7489.47 (-1.49%) | 546034.51 (108.4%) | -47689.62 (-9.47%) | 44591.16(90.24%) | -620.03 (-1.25%) | 52622.17 (106.49%) | -7410.98 (-15%) | 38495.25(86.46%) | -269.93 (-0.61%) | 46961.48 (105.48%) | -8196.3 (-18.41%) | 706399.8(84.15%) | -14842.49 (-1.77%) | 880310.66 (104.87%) | -159068.37 (-18.95%) |
| Afghanistan | -1719.11(-5.89%) | 915.98 (3.14%) | -1396.45 (-4.78%) | -1238.63 (-4.24%) | -64.64(-1.33%) | 682.18 (14.04%) | -238.63 (-4.91%) | -508.19 (-10.46%) | 480.11(8.81%) | 1322.44 (24.27%) | -281.47 (-5.17%) | -560.85 (-10.29%) | -1375.32(-1.31%) | 15633.68 (14.92%) | -5140.7 (-4.9%) | -11868.3 (-11.32%) |
| Albania | 9224.68(127.08%) | 475.32 (6.55%) | 10247.98 (141.17%) | -1498.62 (-20.64%) | 1248.61(131.38%) | 82.54 (8.69%) | 1350.81 (142.14%) | -184.74 (-19.44%) | 969.54(118.4%) | 155.98 (19.05%) | 1141.9 (139.45%) | -328.34 (-40.1%) | 13326(96.31%) | 2041.42 (14.75%) | 18342.31 (132.56%) | -7057.73 (-51.01%) |
| Algeria | 126652.74(182.42%) | 388.07 (0.56%) | 128307.05 (184.81%) | -2042.37 (-2.94%) | 16611.43(177.14%) | 898.49 (9.58%) | 17321.43 (184.71%) | -1608.5 (-17.15%) | 10641.42(151.16%) | 4190.33 (59.52%) | 12551.69 (178.3%) | -6100.61 (-86.66%) | 157174.76(125.4%) | 39741.94 (31.71%) | 211650.29 (168.86%) | -94217.47 (-75.17%) |
| American Samoa | 108(98.44%) | 7.97 (7.27%) | 132.17 (120.48%) | -32.15 (-29.3%) | 11.09(97.3%) | 1.12 (9.82%) | 13.7 (120.15%) | -3.72 (-32.67%) | 5.83(104.64%) | 1.5 (27%) | 6.85 (123.02%) | -2.53 (-45.38%) | 104.34(90.55%) | 16.61 (14.41%) | 136.65 (118.59%) | -48.91 (-42.45%) |
| Andorra | 257.9(108.53%) | 25.88 (10.89%) | 345.07 (145.21%) | -113.04 (-47.57%) | 27.02(102.5%) | 6.44 (24.44%) | 37.59 (142.59%) | -17.01 (-64.52%) | 13.09(107.45%) | 9.96 (81.73%) | 18.48 (151.62%) | -15.34 (-125.91%) | 151.96(72.4%) | 88.88 (42.35%) | 288.04 (137.23%) | -224.96 (-107.18%) |
| Angola | 42835.67(184.54%) | 706.02 (3.04%) | 45855.34 (197.55%) | -3725.68 (-16.05%) | 4376.04(173.11%) | 103.52 (4.1%) | 4897.52 (193.74%) | -625 (-24.72%) | 2562.99(197.92%) | 136.5 (10.54%) | 2615.06 (201.94%) | -188.56 (-14.56%) | 50273.14(183.05%) | 1465.75 (5.34%) | 54100.79 (196.98%) | -5293.39 (-19.27%) |
| Antigua and Barbuda | 125.96(53.72%) | -23.58 (-10.06%) | 197.94 (84.41%) | -48.4 (-20.64%) | 9.96(27.96%) | -7.94 (-22.29%) | 27.77 (77.96%) | -9.87 (-27.7%) | -2.82(-8.4%) | -10.95 (-32.62%) | 22.85 (68.09%) | -14.72 (-43.87%) | -2.06(-0.42%) | -117.28 (-23.88%) | 345.65 (70.39%) | -230.44 (-46.93%) |
| Argentina | 64320.51(34.91%) | 9440.93 (5.12%) | 119276.38 (64.73%) | -64396.79 (-34.95%) | 3149.72(14.89%) | 2019.95 (9.55%) | 12861.83 (60.82%) | -11732.06 (-55.47%) | -3903.52(-25.86%) | 3011.91 (19.96%) | 8095.71 (53.64%) | -15011.14 (-99.46%) | -60358.46(-23.84%) | 30330.7 (11.98%) | 134970.5 (53.31%) | -225659.67 (-89.14%) |
| Armenia | 8981.63(63.5%) | 639.46 (4.52%) | 10758.04 (76.06%) | -2415.87 (-17.08%) | 408.71(18.31%) | 196.71 (8.81%) | 1486.31 (66.59%) | -1274.31 (-57.09%) | 568.59(37.15%) | 240.48 (15.71%) | 1083.59 (70.81%) | -755.48 (-49.37%) | 8041.56(29.3%) | 2745.65 (10%) | 18924.77 (68.94%) | -13628.87 (-49.65%) |
| Australia | 85084.23(76.29%) | 9898.57 (8.88%) | 126308.59 (113.25%) | -51122.93 (-45.84%) | 5279.77(37.34%) | 2305.8 (16.31%) | 14587.32 (103.16%) | -11613.35 (-82.13%) | -557.83(-6.92%) | 3078.62 (38.17%) | 7611.89 (94.38%) | -11248.34 (-139.47%) | -13043.12(-10.45%) | 29492.97 (23.62%) | 114291.92 (91.54%) | -156828.02 (-125.61%) |
| Austria | 59051.04(88.52%) | 5149.13 (7.72%) | 35161.77 (52.71%) | 18740.13 (28.09%) | 626.74(6.19%) | 1144.33 (11.29%) | 4095.02 (40.42%) | -4612.6 (-45.53%) | -5203.5(-62.24%) | 1708.82 (20.44%) | 2575.41 (30.81%) | -9487.73 (-113.49%) | -65464.98(-53.9%) | 15744.67 (12.96%) | 38170.71 (31.43%) | -119380.36 (-98.29%) |
| Azerbaijan | 23762.54(119.47%) | -1717.95 (-8.64%) | 23735.84 (119.33%) | 1744.66 (8.77%) | 3269.45(130.48%) | -425.52 (-16.98%) | 3091.65 (123.38%) | 603.31 (24.08%) | 1054.2(65.34%) | -396.25 (-24.56%) | 1682.58 (104.28%) | -232.12 (-14.39%) | 21928.21(71.42%) | -4842.48 (-15.77%) | 32533.15 (105.97%) | -5762.46 (-18.77%) |
| Bahamas | 776.3(136.6%) | -43.22 (-7.6%) | 945.99 (166.46%) | -126.47 (-22.25%) | 81.49(114.62%) | -6.42 (-9.03%) | 113.33 (159.39%) | -25.41 (-35.74%) | 38.21(78.84%) | -5.94 (-12.26%) | 71.42 (147.36%) | -27.26 (-56.25%) | 593.96(73.35%) | -90.16 (-11.13%) | 1179.75 (145.69%) | -495.62 (-61.2%) |
| Bahrain | 1466.64(326.35%) | -56.12 (-12.49%) | 1816.05 (404.1%) | -293.3 (-65.26%) | 136.65(246.61%) | -8.8 (-15.88%) | 205.72 (371.26%) | -60.27 (-108.77%) | 111.63(155.47%) | -20.02 (-27.88%) | 236.6 (329.53%) | -104.96 (-146.18%) | 2028.69(148.03%) | -343.87 (-25.09%) | 4487.4 (327.44%) | -2114.83 (-154.31%) |
| Bangladesh | 327677.84(211.41%) | 3124.63 (2.02%) | 333570.93 (215.21%) | -9017.71 (-5.82%) | 50828.15(220.07%) | 290.13 (1.26%) | 50395.62 (218.2%) | 142.41 (0.62%) | 48844.1(183.25%) | 123.78 (0.46%) | 54840.5 (205.74%) | -6120.19 (-22.96%) | 757175.5(165.39%) | 6480 (1.42%) | 913654.5 (199.57%) | -162959 (-35.59%) |
| Barbados | 686.82(49.87%) | -114.88 (-8.34%) | 1028.72 (74.7%) | -227.03 (-16.49%) | 58.65(27.16%) | -20.73 (-9.6%) | 149.67 (69.32%) | -70.29 (-32.55%) | 2.19(0.98%) | -24.29 (-10.89%) | 141.45 (63.41%) | -114.97 (-51.54%) | 61.86(1.93%) | -374.63 (-11.7%) | 2036.07 (63.58%) | -1599.59 (-49.95%) |
| Belarus | 17491.99(19.75%) | -18.5 (-0.02%) | 24928.34 (28.15%) | -7417.85 (-8.38%) | 794.94(4.72%) | 548.08 (3.25%) | 4452.98 (26.44%) | -4206.13 (-24.97%) | -128.19(-1.08%) | 678.12 (5.69%) | 3077.66 (25.82%) | -3883.96 (-32.58%) | -11787.81(-5.6%) | 5480.3 (2.6%) | 53277.66 (25.29%) | -70545.77 (-33.49%) |
| Belgium | 25702.99(34.76%) | 4858.04 (6.57%) | 32766.99 (44.31%) | -11922.04 (-16.12%) | -1511.06(-12.02%) | 1609.79 (12.8%) | 4705.12 (37.41%) | -7825.97 (-62.23%) | -4269.83(-47.43%) | 2436 (27.06%) | 3002.04 (33.35%) | -9707.87 (-107.83%) | -59404.81(-45.8%) | 22044.23 (17%) | 42570.83 (32.82%) | -124019.86 (-95.62%) |
| Belize | 486.09(173.39%) | -21.83 (-7.79%) | 540.67 (192.86%) | -32.75 (-11.68%) | 54.18(143.37%) | -5.78 (-15.3%) | 68.96 (182.5%) | -9 (-23.83%) | 33.78(132.68%) | -6.46 (-25.37%) | 45.53 (178.82%) | -5.29 (-20.77%) | 530.38(134.21%) | -71.75 (-18.16%) | 708.54 (179.29%) | -106.41 (-26.93%) |
| Benin | 13274.35(102.3%) | -611.52 (-4.71%) | 16874.61 (130.05%) | -2988.74 (-23.03%) | 1237.01(93.37%) | -87.37 (-6.59%) | 1688.34 (127.43%) | -363.96 (-27.47%) | 1268.29(94.99%) | -152.62 (-11.43%) | 1706.21 (127.79%) | -285.3 (-21.37%) | 22683.56(95.32%) | -1936.92 (-8.14%) | 30442.1 (127.93%) | -5821.62 (-24.46%) |
| Bermuda | 224.91(84.05%) | 20.48 (7.65%) | 316.09 (118.12%) | -111.66 (-41.73%) | 20.23(59.25%) | 6.86 (20.1%) | 38.34 (112.27%) | -24.97 (-73.13%) | 5.62(21.57%) | 8.74 (33.53%) | 27.1 (103.93%) | -30.22 (-115.9%) | 45.53(10.96%) | 81.21 (19.54%) | 412.93 (99.37%) | -448.61 (-107.96%) |
| Bhutan | 1236.72(187.98%) | 85.32 (12.97%) | 1173.12 (178.32%) | -21.71 (-3.3%) | 175.12(196.6%) | 28.52 (32.01%) | 161.95 (181.81%) | -15.35 (-17.23%) | 129.1(205.06%) | 36.5 (57.98%) | 116.41 (184.91%) | -23.81 (-37.82%) | 2016.74(161.81%) | 394.9 (31.68%) | 2128.79 (170.8%) | -506.96 (-40.68%) |
| Bolivia (Plurinational State of) | 14118.32(143.52%) | 143.47 (1.46%) | 17770.11 (180.65%) | -3795.26 (-38.58%) | 1616.09(137.13%) | 47.4 (4.02%) | 2104.47 (178.57%) | -535.78 (-45.46%) | 938(86.67%) | 56.55 (5.23%) | 1753.78 (162.04%) | -872.33 (-80.6%) | 15020.66(79.46%) | 585.69 (3.1%) | 30154.31 (159.51%) | -15719.35 (-83.15%) |
| Bosnia and Herzegovina | 19465.58(78.08%) | 1777.88 (7.13%) | 18676.64 (74.91%) | -988.95 (-3.97%) | 2959.84(71.99%) | 753.94 (18.34%) | 3031.48 (73.73%) | -825.58 (-20.08%) | 2169.17(61.14%) | 1069.23 (30.14%) | 2558.18 (72.11%) | -1458.24 (-41.1%) | 28369.11(44.61%) | 12273.44 (19.3%) | 43453.77 (68.33%) | -27358.1 (-43.02%) |
| Botswana | 6750.17(162.15%) | 186.44 (4.48%) | 6492.45 (155.96%) | 71.28 (1.71%) | 912.78(177.89%) | 32.09 (6.25%) | 824.43 (160.68%) | 56.26 (10.96%) | 244.5(89.86%) | 44.19 (16.24%) | 367.52 (135.08%) | -167.21 (-61.46%) | 4729.82(85.49%) | 369.15 (6.67%) | 7368.24 (133.17%) | -3007.57 (-54.36%) |
| Brazil | 549612(122.48%) | 20643.58 (4.6%) | 781584.24 (174.17%) | -252615.82 (-56.29%) | 57491.2(91.74%) | 9048.01 (14.44%) | 103713.75 (165.5%) | -55270.55 (-88.2%) | 16574.96(33.43%) | 13415.03 (27.06%) | 74017.85 (149.3%) | -70857.92 (-142.93%) | 225197.05(26.24%) | 120133.48 (14%) | 1241412.37 (144.65%) | -1136348.8 (-132.4%) |
| Brunei Darussalam | 930.08(95.85%) | -121.63 (-12.53%) | 2011.73 (207.32%) | -960.02 (-98.93%) | 80.62(82.04%) | -10.1 (-10.28%) | 200.84 (204.38%) | -110.12 (-112.06%) | 14.48(29.4%) | -18.92 (-38.41%) | 88.79 (180.29%) | -55.39 (-112.48%) | 376.99(39.87%) | -225.53 (-23.85%) | 1756.62 (185.77%) | -1154.1 (-122.05%) |
| Bulgaria | 13003.88(14.23%) | 5516.86 (6.04%) | 13243.59 (14.49%) | -5756.57 (-6.3%) | 4935.08(31.98%) | 3993.96 (25.88%) | 2396.74 (15.53%) | -1455.62 (-9.43%) | 6363.36(42.96%) | 8010.05 (54.08%) | 2467.22 (16.66%) | -4113.9 (-27.77%) | 59491.4(22.54%) | 80077.02 (30.34%) | 40168.69 (15.22%) | -60754.31 (-23.02%) |
| Burkina Faso | 17139.33(92.77%) | 530.9 (2.87%) | 18751.08 (101.49%) | -2142.66 (-11.6%) | 1612.97(104.04%) | 101.11 (6.52%) | 1617.73 (104.34%) | -105.86 (-6.83%) | 1583.28(126.09%) | 191.69 (15.27%) | 1380.58 (109.95%) | 11.01 (0.88%) | 27836.23(112.32%) | 1818.81 (7.34%) | 26384.39 (106.46%) | -366.97 (-1.48%) |
| Burundi | 6797(40.37%) | -1291.2 (-7.67%) | 14498.45 (86.12%) | -6410.25 (-38.08%) | 671.31(35.28%) | -178.69 (-9.39%) | 1612.58 (84.74%) | -762.59 (-40.07%) | 92.32(6.84%) | -187.25 (-13.87%) | 1040.14 (77.02%) | -760.56 (-56.32%) | 2125.66(8.11%) | -3022.11 (-11.53%) | 20332.65 (77.55%) | -15184.88 (-57.92%) |
| Cabo Verde | 1047.23(74.08%) | -158.54 (-11.21%) | 1136.33 (80.38%) | 69.43 (4.91%) | 106.22(91.54%) | -16.51 (-14.23%) | 98.18 (84.61%) | 24.55 (21.16%) | 135.22(118.67%) | -21.19 (-18.6%) | 103.25 (90.61%) | 53.17 (46.66%) | 2093.09(110.3%) | -349.14 (-18.4%) | 1683.29 (88.7%) | 758.94 (39.99%) |
| Cambodia | 33509.97(189.74%) | 40.24 (0.23%) | 33466.71 (189.5%) | 3.02 (0.02%) | 4993.87(203.85%) | 63.02 (2.57%) | 4755.75 (194.13%) | 175.1 (7.15%) | 3609.14(177.07%) | 127.57 (6.26%) | 3775.11 (185.22%) | -293.54 (-14.4%) | 62382.46(160.99%) | 1198.3 (3.09%) | 69754.12 (180.02%) | -8569.97 (-22.12%) |
| Cameroon | 38339.37(167.86%) | 175.47 (0.77%) | 38993.75 (170.73%) | -829.85 (-3.63%) | 3651.28(179.44%) | 15.49 (0.76%) | 3548.33 (174.38%) | 87.45 (4.3%) | 3537.63(201.7%) | 8.67 (0.49%) | 3181.86 (181.42%) | 347.1 (19.79%) | 67220.23(202.01%) | 200.63 (0.6%) | 60398.23 (181.51%) | 6621.38 (19.9%) |
| Canada | 216985.1(103.02%) | 10124.7 (4.81%) | 253579.92 (120.4%) | -46719.52 (-22.18%) | 6523.63(25.81%) | 2039.52 (8.07%) | 25114.35 (99.36%) | -20630.24 (-81.62%) | 263.17(2.53%) | 2569.81 (24.69%) | 9846.18 (94.61%) | -12152.81 (-116.77%) | 16743.09(10%) | 24161.48 (14.43%) | 160077.31 (95.58%) | -167495.69 (-100.01%) |
| Central African Republic | 3694.4(64.17%) | -62.18 (-1.08%) | 4454.32 (77.37%) | -697.74 (-12.12%) | 425.03(64.24%) | -9.22 (-1.39%) | 512.01 (77.38%) | -77.76 (-11.75%) | 255.03(58.17%) | -2.24 (-0.51%) | 333.14 (75.99%) | -75.87 (-17.31%) | 5101.21(54.93%) | -130.13 (-1.4%) | 6989.88 (75.27%) | -1758.54 (-18.94%) |
| Chad | 11948.38(71.09%) | -1067.05 (-6.35%) | 14241.44 (84.73%) | -1226.01 (-7.29%) | 1093.1(68.74%) | -140.64 (-8.84%) | 1339.03 (84.2%) | -105.29 (-6.62%) | 1366.08(94.4%) | -242.51 (-16.76%) | 1308.73 (90.44%) | 299.86 (20.72%) | 26752.9(99.07%) | -3320.52 (-12.3%) | 24695.74 (91.45%) | 5377.67 (19.91%) |
| Chile | 54503.31(108.16%) | 1675.5 (3.32%) | 76148.52 (151.11%) | -23320.71 (-46.28%) | 4683.48(75.38%) | 608.69 (9.8%) | 8750.45 (140.84%) | -4675.67 (-75.26%) | 1137.17(24.25%) | 1116.97 (23.82%) | 5948.13 (126.85%) | -5927.92 (-126.42%) | 11699.05(14.69%) | 9840.57 (12.36%) | 97729.64 (122.7%) | -95871.16 (-120.37%) |
| China | 11561272.93(316.23%) | 742984.3 (20.32%) | 7797121.83 (213.27%) | 3021166.8 (82.64%) | 1682567.14(318.85%) | 143507.69 (27.2%) | 1131974.4 (214.51%) | 407085.05 (77.14%) | 731728.88(192.05%) | 193064.84 (50.67%) | 671089.35 (176.14%) | -132425.3 (-34.76%) | 12439444.74(165.03%) | 2077970.27 (27.57%) | 12615946.18 (167.37%) | -2254471.71 (-29.91%) |
| Colombia | 101387.86(137.66%) | 8183.72 (11.11%) | 152128.77 (206.56%) | -58924.63 (-80.01%) | 9853.18(110.97%) | 1895.52 (21.35%) | 17596.51 (198.18%) | -9638.85 (-108.56%) | 2873.85(58.06%) | 1494.59 (30.19%) | 9069.14 (183.21%) | -7689.88 (-155.35%) | 41895.89(48.68%) | 13859.66 (16.1%) | 152522.8 (177.21%) | -124486.57 (-144.64%) |
| Comoros | 1666.31(119.73%) | 116.76 (8.39%) | 1942.49 (139.57%) | -392.94 (-28.23%) | 175.49(122.58%) | 16.33 (11.41%) | 200.97 (140.38%) | -41.81 (-29.2%) | 84.85(108.59%) | 20.61 (26.38%) | 107.05 (136.99%) | -42.81 (-54.79%) | 1484.66(92.89%) | 227.84 (14.26%) | 2107.46 (131.86%) | -850.64 (-53.22%) |
| Congo | 7943.8(96.6%) | 40.47 (0.49%) | 9861.98 (119.92%) | -1958.66 (-23.82%) | 850.63(94.98%) | 11.17 (1.25%) | 1069.32 (119.39%) | -229.86 (-25.67%) | 488.82(97.33%) | 45.57 (9.07%) | 603.75 (120.22%) | -160.5 (-31.96%) | 9120.25(87%) | 218.96 (2.09%) | 12286.61 (117.21%) | -3385.33 (-32.3%) |
| Cook Islands | 90.76(151.4%) | 7.42 (12.38%) | 82.28 (137.24%) | 1.06 (1.77%) | 7.99(127.47%) | 0.89 (14.24%) | 8.19 (130.61%) | -1.09 (-17.38%) | 2(54.41%) | 1.14 (31.17%) | 4.11 (112.05%) | -3.26 (-88.81%) | 39.22(55.67%) | 12.68 (18%) | 78.41 (111.29%) | -51.87 (-73.62%) |
| Costa Rica | 11757.31(178.71%) | 145.63 (2.21%) | 14175.27 (215.46%) | -2563.58 (-38.97%) | 1151.83(141.56%) | 65.65 (8.07%) | 1648.93 (202.66%) | -562.75 (-69.16%) | 478.92(120.22%) | 57.8 (14.51%) | 780.76 (195.99%) | -359.65 (-90.28%) | 6848.18(108.64%) | 383.16 (6.08%) | 12055.14 (191.24%) | -5590.12 (-88.68%) |
| Coted'Ivoire | 33314.84(146.65%) | 1190.3 (5.24%) | 39142.84 (172.3%) | -7018.3 (-30.89%) | 3133.74(154.64%) | 175.06 (8.64%) | 3543.69 (174.87%) | -585.01 (-28.87%) | 3169.31(186.6%) | 317.34 (18.68%) | 3147.64 (185.33%) | -295.67 (-17.41%) | 59416.14(169.93%) | 3378.24 (9.66%) | 62886.51 (179.85%) | -6848.6 (-19.59%) |
| Croatia | 17643.76(48.82%) | 5125.82 (14.18%) | 18220.43 (50.41%) | -5702.5 (-15.78%) | 879.32(11.91%) | 1567.91 (21.24%) | 3318.61 (44.96%) | -4007.19 (-54.29%) | -1814.98(-26.79%) | 2272.92 (33.55%) | 2708.52 (39.98%) | -6796.42 (-100.33%) | -33703.66(-31.27%) | 26908.82 (24.96%) | 41740.12 (38.72%) | -102352.6 (-94.96%) |
| Cuba | 29792.6(77.46%) | 439.93 (1.14%) | 33352.44 (86.72%) | -3999.78 (-10.4%) | 4044.36(81.14%) | 617.04 (12.38%) | 4377.09 (87.81%) | -949.77 (-19.05%) | 3190.86(82.59%) | 1078.71 (27.92%) | 3445.08 (89.17%) | -1332.92 (-34.5%) | 43648.55(71.23%) | 8597.9 (14.03%) | 52637.94 (85.9%) | -17587.29 (-28.7%) |
| Cyprus | 774.59(22.89%) | 244.73 (7.23%) | 3897.57 (115.17%) | -3367.7 (-99.51%) | 90.6(15.94%) | 123.61 (21.75%) | 633.49 (111.44%) | -666.51 (-117.25%) | -38.81(-7.1%) | 448.43 (82%) | 675.44 (123.51%) | -1162.68 (-212.61%) | -1208.4(-15.13%) | 4611.87 (57.76%) | 9275.85 (116.16%) | -15096.12 (-189.05%) |
| Czechia | 26681.26(25.4%) | 7866.44 (7.49%) | 50696.3 (48.26%) | -31881.47 (-30.35%) | -4433.96(-20.83%) | 2323.79 (10.92%) | 8681.92 (40.79%) | -15439.68 (-72.53%) | -13323.18(-64.96%) | 3021.87 (14.73%) | 6959.45 (33.93%) | -23304.5 (-113.62%) | -208188.12(-63.51%) | 33568.27 (10.24%) | 110683.56 (33.77%) | -352439.94 (-107.52%) |
| Democratic People's Republic of Korea | 130634.26(137.37%) | 12144.18 (12.77%) | 118291.67 (124.39%) | 198.41 (0.21%) | 18565.92(146.99%) | 1808.06 (14.31%) | 16056.4 (127.12%) | 701.45 (5.55%) | 9842.78(142.03%) | 2093.71 (30.21%) | 8748.5 (126.24%) | -999.43 (-14.42%) | 184867.25(130.12%) | 24192.87 (17.03%) | 174426.82 (122.78%) | -13752.44 (-9.68%) |
| Democratic Republic of the Congo | 83202.95(99.88%) | 2130.56 (2.56%) | 97019.24 (116.46%) | -15946.86 (-19.14%) | 9639.17(101.12%) | 460.32 (4.83%) | 11129.98 (116.76%) | -1951.13 (-20.47%) | 6146.04(122.46%) | 917.68 (18.28%) | 6153.13 (122.6%) | -924.77 (-18.43%) | 110121.38(103.41%) | 7420.31 (6.97%) | 124957.08 (117.34%) | -22256.01 (-20.9%) |
| Denmark | -8233.43(-14.31%) | 748.88 (1.3%) | 20721.44 (36.02%) | -29703.76 (-51.64%) | -2249.19(-30.7%) | 201.59 (2.75%) | 2454.57 (33.5%) | -4905.35 (-66.96%) | -1649.07(-38.17%) | 314.21 (7.27%) | 1406.27 (32.55%) | -3369.55 (-77.99%) | -27197.16(-40.77%) | 2929.43 (4.39%) | 21375.7 (32.04%) | -51502.3 (-77.2%) |
| Djibouti | 2923.6(371.23%) | 24.5 (3.11%) | 3021.47 (383.65%) | -122.37 (-15.54%) | 305.61(383.76%) | 2.98 (3.74%) | 309.44 (388.57%) | -6.81 (-8.55%) | 134.34(380.86%) | 2.72 (7.72%) | 136.72 (387.6%) | -5.1 (-14.46%) | 2846.81(376.26%) | 41.53 (5.49%) | 2918.59 (385.74%) | -113.31 (-14.98%) |
| Dominica | 47.09(21.65%) | -12.3 (-5.65%) | 80.92 (37.2%) | -21.54 (-9.9%) | 6.94(23.6%) | -2.11 (-7.18%) | 11.04 (37.51%) | -1.98 (-6.73%) | 1.72(4.72%) | -3.25 (-8.94%) | 12.64 (34.73%) | -7.67 (-21.07%) | 23.07(4.17%) | -51.53 (-9.31%) | 191.81 (34.64%) | -117.21 (-21.17%) |
| Dominican Republic | 29560.51(228.63%) | 471.31 (3.65%) | 25250.46 (195.29%) | 3838.74 (29.69%) | 3736.67(279.98%) | 251.26 (18.83%) | 2824.72 (211.65%) | 660.69 (49.5%) | 1987.55(191.96%) | 402.53 (38.88%) | 1934.88 (186.87%) | -349.87 (-33.79%) | 32386.09(188.38%) | 3464.43 (20.15%) | 31666.66 (184.2%) | -2745 (-15.97%) |
| Ecuador | 35773.89(176.66%) | 729.28 (3.6%) | 42629.58 (210.52%) | -7584.97 (-37.46%) | 3503.91(165.47%) | 143.21 (6.76%) | 4376.68 (206.68%) | -1015.99 (-47.98%) | 1227.2(85.79%) | 159.38 (11.14%) | 2570.98 (179.74%) | -1503.16 (-105.08%) | 17905.82(74.62%) | 1665.81 (6.94%) | 42114.45 (175.5%) | -25874.43 (-107.82%) |
| Egypt | 205298.43(209.74%) | -387.16 (-0.4%) | 160293.61 (163.76%) | 45391.98 (46.37%) | 26756.7(176.6%) | -643.25 (-4.25%) | 23327.04 (153.96%) | 4072.9 (26.88%) | 21080.62(80.57%) | -2450.58 (-9.37%) | 32801.76 (125.36%) | -9270.56 (-35.43%) | 426720.24(89.15%) | -27396.64 (-5.72%) | 612559.56 (127.97%) | -158442.69 (-33.1%) |
| El Salvador | 8727.14(90.73%) | 816.09 (8.48%) | 10547.85 (109.66%) | -2636.8 (-27.41%) | 993.92(82.29%) | 192.41 (15.93%) | 1298.97 (107.55%) | -497.46 (-41.19%) | 403.33(63.9%) | 130.65 (20.7%) | 650.46 (103.05%) | -377.78 (-59.85%) | 5256.34(49.51%) | 1332.24 (12.55%) | 10498.21 (98.89%) | -6574.11 (-61.93%) |
| Equatorial Guinea | 1453.74(120.73%) | 76.78 (6.38%) | 1659.35 (137.81%) | -282.38 (-23.45%) | 143.93(103.15%) | 10.38 (7.44%) | 185.36 (132.84%) | -51.81 (-37.13%) | 86.81(108.53%) | 13.79 (17.24%) | 107.7 (134.64%) | -34.68 (-43.36%) | 1527.21(93.2%) | 138.54 (8.46%) | 2128.99 (129.93%) | -740.32 (-45.18%) |
| Eritrea | 8628.6(160.1%) | 580.81 (10.78%) | 8909.67 (165.31%) | -861.88 (-15.99%) | 836.95(143.5%) | 86.73 (14.87%) | 935.42 (160.39%) | -185.2 (-31.75%) | 463.77(169.22%) | 96.91 (35.36%) | 461.36 (168.34%) | -94.5 (-34.48%) | 8920.24(144.32%) | 1242.97 (20.11%) | 9913.11 (160.38%) | -2235.83 (-36.17%) |
| Estonia | 138.52(1.25%) | 41.45 (0.37%) | 3151.99 (28.44%) | -3054.92 (-27.56%) | -972.39(-38.49%) | 331.13 (13.11%) | 612.43 (24.24%) | -1915.95 (-75.84%) | -1713.37(-66.45%) | 519.08 (20.13%) | 554 (21.48%) | -2786.45 (-108.06%) | -28979.75(-68.11%) | 4755.54 (11.18%) | 8831.96 (20.76%) | -42567.25 (-100.05%) |
| Eswatini | 1599.04(98.48%) | -81.12 (-5%) | 1643.97 (101.24%) | 36.19 (2.23%) | 221.52(107.08%) | -17.08 (-8.25%) | 214.57 (103.71%) | 24.03 (11.62%) | 114.37(90.81%) | -18.92 (-15.02%) | 124.92 (99.18%) | 8.37 (6.64%) | 2403.65(100.31%) | -195.58 (-8.16%) | 2436.15 (101.66%) | 163.08 (6.81%) |
| Ethiopia | 79251.2(94.86%) | 11782.16 (14.1%) | 93346.03 (111.74%) | -25876.99 (-30.97%) | 9141.28(101.61%) | 1962.46 (21.81%) | 10207 (113.46%) | -3028.17 (-33.66%) | 4382.38(119.31%) | 1723.65 (46.93%) | 4370.53 (118.99%) | -1711.8 (-46.6%) | 70958.48(86.64%) | 18789.53 (22.94%) | 89874.82 (109.74%) | -37705.88 (-46.04%) |
| Fiji | 2162.01(116.58%) | -40.43 (-2.18%) | 2580.51 (139.15%) | -378.07 (-20.39%) | 216.91(107.95%) | -5.83 (-2.9%) | 274.36 (136.55%) | -51.62 (-25.69%) | 97.23(112.46%) | -5.06 (-5.85%) | 119.3 (137.99%) | -17.01 (-19.68%) | 2000.59(107.4%) | -66.46 (-3.57%) | 2541.5 (136.44%) | -474.44 (-25.47%) |
| Finland | 21760.43(36.36%) | 5843.43 (9.76%) | 40493.12 (67.65%) | -24576.12 (-41.06%) | 415.31(5.44%) | 976.93 (12.8%) | 4670.12 (61.19%) | -5231.74 (-68.55%) | -934.51(-22.54%) | 1106.65 (26.69%) | 2348.5 (56.65%) | -4389.66 (-105.88%) | -17989.9(-26.33%) | 11140.3 (16.3%) | 37594.17 (55.02%) | -66724.36 (-97.66%) |
| France | 256868.23(72.6%) | 21643.03 (6.12%) | 232639.11 (65.76%) | 2586.09 (0.73%) | 12755.19(27.87%) | 6872.91 (15.01%) | 26356.55 (57.58%) | -20474.27 (-44.73%) | -12055.92(-29.89%) | 13181.27 (32.68%) | 19441.63 (48.2%) | -44678.83 (-110.78%) | -151653.5(-27.47%) | 117459.01 (21.27%) | 263913.97 (47.8%) | -533026.48 (-96.54%) |
| Gabon | 1994.84(41.67%) | -306.15 (-6.4%) | 3140.98 (65.62%) | -839.99 (-17.55%) | 222.01(45.04%) | -39.66 (-8.05%) | 327.13 (66.37%) | -65.46 (-13.28%) | 103.31(38.81%) | -35.62 (-13.38%) | 172.85 (64.93%) | -33.91 (-12.74%) | 2003.13(39.21%) | -535.5 (-10.48%) | 3322.85 (65.04%) | -784.23 (-15.35%) |
| Gambia | 3563.8(166.63%) | 96.26 (4.5%) | 3763.51 (175.96%) | -295.97 (-13.84%) | 347.88(180.81%) | 17.78 (9.24%) | 347.11 (180.41%) | -17 (-8.84%) | 430.96(247.02%) | 38.35 (21.98%) | 350.81 (201.07%) | 41.81 (23.96%) | 7529.11(217.72%) | 331.18 (9.58%) | 6640.52 (192.03%) | 557.41 (16.12%) |
| Georgia | 4324.59(14.39%) | 485.68 (1.62%) | 39.79 (0.13%) | 3799.11 (12.65%) | 894.29(17.17%) | 770.57 (14.79%) | 7 (0.13%) | 116.72 (2.24%) | 2095.51(51.06%) | 1061.9 (25.88%) | 6.36 (0.16%) | 1027.25 (25.03%) | 25624.3(37.18%) | 9811.79 (14.24%) | 100.83 (0.15%) | 15711.68 (22.8%) |
| Germany | 333313.09(31.92%) | 70374.75 (6.74%) | 497904.37 (47.68%) | -234966.03 (-22.5%) | -6557.45(-4.9%) | 12635.15 (9.43%) | 56082.34 (41.87%) | -75274.94 (-56.2%) | -47050.81(-52.1%) | 18045.67 (19.98%) | 31732.4 (35.14%) | -96828.88 (-107.22%) | -624608.48(-45.87%) | 175290.63 (12.87%) | 484089.89 (35.55%) | -1283988.99 (-94.29%) |
| Ghana | 83494.49(179.26%) | 387.17 (0.83%) | 80892.24 (173.68%) | 2215.08 (4.76%) | 7488.68(184.77%) | 82.81 (2.04%) | 7109.53 (175.41%) | 296.34 (7.31%) | 6692.84(188.91%) | 174.5 (4.93%) | 6258.62 (176.65%) | 259.73 (7.33%) | 128302.7(181.88%) | 1467.54 (2.08%) | 123079.94 (174.48%) | 3755.23 (5.32%) |
| Greece | 25462.16(27.67%) | 13415.04 (14.58%) | 41847.21 (45.48%) | -29800.1 (-32.39%) | 1174.59(7.28%) | 5522.4 (34.21%) | 7003.4 (43.39%) | -11351.21 (-70.32%) | -3456.06(-24.15%) | 8374.18 (58.5%) | 5847.78 (40.85%) | -17678.01 (-123.5%) | -64684.79(-32.1%) | 80681.48 (40.03%) | 77069.34 (38.24%) | -222435.61 (-110.37%) |
| Greenland | 122.86(43.12%) | -0.9 (-0.32%) | 333.71 (117.11%) | -209.94 (-73.67%) | 5.66(17.63%) | 0.5 (1.57%) | 35.24 (109.73%) | -30.08 (-93.67%) | -2.5(-15.1%) | 0.58 (3.53%) | 16.52 (99.89%) | -19.61 (-118.52%) | -37.83(-12.41%) | 2.14 (0.7%) | 306.25 (100.46%) | -346.22 (-113.57%) |
| Grenada | 65.72(17.45%) | -16.01 (-4.25%) | 159.09 (42.25%) | -77.35 (-20.54%) | -3.35(-5.25%) | -16.34 (-25.58%) | 24.8 (38.81%) | -11.81 (-18.48%) | -30.85(-42.02%) | -22.03 (-30.01%) | 23.59 (32.13%) | -32.4 (-44.13%) | -416.61(-37.29%) | -206.05 (-18.44%) | 370.89 (33.2%) | -581.46 (-52.04%) |
| Guam | 892.56(244.49%) | 126.62 (34.68%) | 823.22 (225.49%) | -57.28 (-15.69%) | 70.56(181.65%) | 15.12 (38.93%) | 79.41 (204.45%) | -23.98 (-61.73%) | 6.96(42.68%) | 14.33 (87.8%) | 29.73 (182.22%) | -37.1 (-227.34%) | 277.23(78.11%) | 154.04 (43.4%) | 635.98 (179.2%) | -512.79 (-144.49%) |
| Guatemala | 18330.03(196.27%) | 1403.94 (15.03%) | 20805.86 (222.78%) | -3879.77 (-41.54%) | 2222.77(200.35%) | 370.68 (33.41%) | 2485.48 (224.03%) | -633.39 (-57.09%) | 975.08(163.92%) | 353.55 (59.44%) | 1283.92 (215.84%) | -662.4 (-111.36%) | 14109.23(134.2%) | 3682.76 (35.03%) | 21375.62 (203.32%) | -10949.15 (-104.14%) |
| Guinea | 11341.08(55.75%) | -88.91 (-0.44%) | 11757.18 (57.79%) | -327.18 (-1.61%) | 1104(56.7%) | 8.92 (0.46%) | 1128.54 (57.96%) | -33.46 (-1.72%) | 1411.37(82.05%) | 36.49 (2.12%) | 1077.18 (62.62%) | 297.7 (17.31%) | 25323.79(79.02%) | 56.39 (0.18%) | 19894.75 (62.08%) | 5372.65 (16.77%) |
| Guinea-Bissau | 1359.56(54.77%) | -60.12 (-2.42%) | 1662.57 (66.98%) | -242.88 (-9.78%) | 122.89(55.29%) | -7.3 (-3.28%) | 149.18 (67.11%) | -18.99 (-8.54%) | 135.97(52.51%) | -13.39 (-5.17%) | 172.14 (66.48%) | -22.77 (-8.79%) | 2674.63(50.25%) | -211.03 (-3.96%) | 3514.36 (66.03%) | -628.69 (-11.81%) |
| Guyana | 718.51(37.54%) | -33.5 (-1.75%) | 1320.68 (69.01%) | -568.68 (-29.71%) | 75.28(24.54%) | -12.36 (-4.03%) | 202.71 (66.08%) | -115.07 (-37.51%) | -0.69(-0.22%) | -19.54 (-6.14%) | 192.64 (60.54%) | -173.79 (-54.61%) | -307.91(-5.32%) | -232.8 (-4.02%) | 3444.02 (59.52%) | -3519.14 (-60.82%) |
| Haiti | 10877.97(93.42%) | -58.65 (-0.5%) | 12720.4 (109.24%) | -1783.77 (-15.32%) | 1594.86(94.12%) | 61.71 (3.64%) | 1854.65 (109.45%) | -321.49 (-18.97%) | 1478.42(71.58%) | 132 (6.39%) | 2139.47 (103.58%) | -793.05 (-38.39%) | 24834.28(65.82%) | 945.83 (2.51%) | 38458.14 (101.93%) | -14569.69 (-38.62%) |
| Honduras | 12282.39(207.17%) | -37.15 (-0.63%) | 12772.21 (215.43%) | -452.67 (-7.64%) | 1727.01(246.01%) | -32.51 (-4.63%) | 1607.04 (228.92%) | 152.48 (21.72%) | 1873.87(322.14%) | -45.27 (-7.78%) | 1486.77 (255.6%) | 432.37 (74.33%) | 31038.65(311.37%) | -337.37 (-3.38%) | 25077.85 (251.57%) | 6298.17 (63.18%) |
| Hungary | -18751.77(-14.54%) | 4811.03 (3.73%) | 34885.33 (27.05%) | -58448.13 (-45.31%) | -4387.51(-22.22%) | 2082.23 (10.54%) | 5199.06 (26.32%) | -11668.8 (-59.08%) | -7985.97(-46.92%) | 3246.42 (19.08%) | 4084.74 (24%) | -15317.13 (-90%) | -141594.25(-48.69%) | 30350.76 (10.44%) | 67854.29 (23.33%) | -239799.3 (-82.45%) |
| Iceland | 664.83(35.25%) | 11.42 (0.61%) | 1648.21 (87.38%) | -994.79 (-52.74%) | -5.39(-2.13%) | 6.87 (2.72%) | 196.82 (77.9%) | -209.08 (-82.75%) | -23.19(-19.01%) | 11.25 (9.22%) | 90.3 (74.04%) | -124.73 (-102.28%) | -415.06(-22.03%) | 84.21 (4.47%) | 1373.99 (72.93%) | -1873.26 (-99.43%) |
| India | 1760219.43(161.21%) | 57372.99 (5.25%) | 1906219.87 (174.58%) | -203373.42 (-18.63%) | 224243.28(135.84%) | 26964.9 (16.33%) | 275443.33 (166.85%) | -78164.96 (-47.35%) | 186258.13(174.31%) | 27654.64 (25.88%) | 191461.94 (179.18%) | -32858.44 (-30.75%) | 3322386.24(153.1%) | 305204.5 (14.06%) | 3736657.37 (172.19%) | -719475.62 (-33.16%) |
| Indonesia | 947887.8(174.67%) | -3085.73 (-0.57%) | 888441.31 (163.71%) | 62532.22 (11.52%) | 130925.69(208.01%) | -2474.62 (-3.93%) | 109576.48 (174.09%) | 23823.83 (37.85%) | 93064.4(224.05%) | -4940.72 (-11.89%) | 74599.12 (179.59%) | 23406.01 (56.35%) | 1761970.09(215.48%) | -43266.66 (-5.29%) | 1443726.53 (176.56%) | 361510.22 (44.21%) |
| Iran (Islamic Republic of) | 195245.28(185.75%) | 5710.75 (5.43%) | 202410.98 (192.57%) | -12876.45 (-12.25%) | 22757.45(150.15%) | 4934.82 (32.56%) | 27779.37 (183.29%) | -9956.75 (-65.69%) | 16848.3(119.43%) | 8210.71 (58.2%) | 24940.24 (176.78%) | -16302.65 (-115.56%) | 240176.7(88.84%) | 82850.55 (30.64%) | 443040.72 (163.87%) | -285714.57 (-105.68%) |
| Iraq | 79577.42(206.47%) | -777.37 (-2.02%) | 75865.22 (196.84%) | 4489.57 (11.65%) | 11478.82(175.86%) | -1627.25 (-24.93%) | 12216.24 (187.15%) | 889.83 (13.63%) | 10158.62(130.57%) | -2998.52 (-38.54%) | 13496.46 (173.47%) | -339.32 (-4.36%) | 185590.52(137.21%) | -26833.77 (-19.84%) | 236660.11 (174.96%) | -24235.82 (-17.92%) |
| Ireland | -1906.72(-8.35%) | 255.47 (1.12%) | 15086.93 (66.07%) | -17249.12 (-75.54%) | -1140.87(-31.69%) | 249.34 (6.93%) | 2193.46 (60.92%) | -3583.67 (-99.54%) | -1090.27(-46.4%) | 453.9 (19.32%) | 1389.33 (59.13%) | -2933.5 (-124.85%) | -18955.34(-51.89%) | 3764.8 (10.31%) | 20757.98 (56.83%) | -43478.12 (-119.03%) |
| Israel | 23097.96(68.68%) | 2060.33 (6.13%) | 42175.66 (125.4%) | -21138.04 (-62.85%) | 720.04(19.43%) | 438.31 (11.83%) | 4120.06 (111.19%) | -3838.32 (-103.58%) | 54.45(3.57%) | 582.41 (38.22%) | 1685.09 (110.57%) | -2213.05 (-145.22%) | 141.76(0.53%) | 5257.74 (19.63%) | 28680.69 (107.09%) | -33796.67 (-126.19%) |
| Italy | 79828.49(21.12%) | 45697.49 (12.09%) | 178004.29 (47.09%) | -143873.29 (-38.06%) | -15257.71(-20.34%) | 16305.37 (21.73%) | 30817.84 (41.08%) | -62380.92 (-83.15%) | -15004.51(-26.52%) | 26527.75 (46.88%) | 23733.94 (41.94%) | -65266.2 (-115.34%) | -295685.3(-36.3%) | 252224.25 (30.96%) | 319338.66 (39.2%) | -867248.21 (-106.46%) |
| Jamaica | 3022.29(44.98%) | -339.57 (-5.05%) | 4369.68 (65.03%) | -1007.82 (-15%) | 556.78(46.2%) | 36.68 (3.04%) | 788.65 (65.43%) | -268.56 (-22.28%) | 350.11(28.53%) | 75.23 (6.13%) | 758.58 (61.82%) | -483.69 (-39.42%) | 3541(19.53%) | -196.35 (-1.08%) | 10838.05 (59.77%) | -7100.7 (-39.16%) |
| Japan | 996168.81(92.74%) | 292633.84 (27.24%) | 1082462.57 (100.77%) | -378927.59 (-35.28%) | 49149.93(37.84%) | 51296.63 (39.49%) | 116918.58 (90.02%) | -119065.28 (-91.67%) | 7237.4(8.93%) | 75479.89 (93.16%) | 74088.82 (91.44%) | -142331.3 (-175.67%) | 37119.41(2.88%) | 757766.6 (58.76%) | 1096480.06 (85.02%) | -1817127.24 (-140.9%) |
| Jordan | 38064.54(523.34%) | 377.72 (5.19%) | 36179.19 (497.42%) | 1507.63 (20.73%) | 4326.67(385.57%) | 35.13 (3.13%) | 4942.81 (440.48%) | -651.27 (-58.04%) | 1459.51(169.96%) | -7.87 (-0.92%) | 3027.03 (352.5%) | -1559.66 (-181.62%) | 28302.34(179.47%) | 424.23 (2.69%) | 56206.54 (356.41%) | -28328.43 (-179.63%) |
| Kazakhstan | 21207.98(20.5%) | -172.41 (-0.17%) | 41795.79 (40.4%) | -20415.4 (-19.74%) | 2102.61(14.24%) | -695.14 (-4.71%) | 5818.86 (39.42%) | -3021.12 (-20.47%) | 814.54(7.1%) | -1163.49 (-10.14%) | 4404.78 (38.39%) | -2426.76 (-21.15%) | 11051.95(5.31%) | -10031.18 (-4.82%) | 79263.55 (38.09%) | -58180.42 (-27.96%) |
| Kenya | 70105.48(149.98%) | -3658.89 (-7.83%) | 78639.87 (168.24%) | -4875.5 (-10.43%) | 7620.08(158.1%) | -525.32 (-10.9%) | 8247.92 (171.13%) | -102.52 (-2.13%) | 3452.66(172.41%) | -482.79 (-24.11%) | 3522.53 (175.9%) | 412.92 (20.62%) | 68137.89(175.81%) | -6027.26 (-15.55%) | 68512.66 (176.78%) | 5652.5 (14.58%) |
| Kiribati | 159.38(73.87%) | -9.88 (-4.58%) | 199.72 (92.57%) | -30.46 (-14.12%) | 17.43(72.73%) | -1.55 (-6.45%) | 22.13 (92.32%) | -3.15 (-13.14%) | 9.37(88.32%) | -0.96 (-9.02%) | 10.21 (96.21%) | 0.12 (1.14%) | 197.75(83.57%) | -15.9 (-6.72%) | 224.81 (95%) | -11.15 (-4.71%) |
| Kuwait | 8029.37(352.93%) | 76.97 (3.38%) | 8501.27 (373.68%) | -548.86 (-24.13%) | 855.2(364.41%) | 43.14 (18.38%) | 888.66 (378.67%) | -76.6 (-32.64%) | 336.3(290.89%) | 36.83 (31.86%) | 403.82 (349.28%) | -104.35 (-90.25%) | 5656.41(247.13%) | 290.59 (12.7%) | 7590.46 (331.63%) | -2224.64 (-97.19%) |
| Kyrgyzstan | 1603.01(8.64%) | -305.48 (-1.65%) | 8424.31 (45.41%) | -6515.82 (-35.12%) | -102.39(-3.34%) | -170.23 (-5.55%) | 1324.37 (43.19%) | -1256.53 (-40.97%) | -735.79(-27.45%) | -246.95 (-9.21%) | 1035.39 (38.62%) | -1524.23 (-56.86%) | -7248.86(-15.41%) | -2713.53 (-5.77%) | 19212.63 (40.85%) | -23747.97 (-50.49%) |
| Lao People's Democratic Republic | 11022.09(106.29%) | -146.44 (-1.41%) | 11940.71 (115.15%) | -772.17 (-7.45%) | 1478.01(109.27%) | 68.6 (5.07%) | 1570.08 (116.07%) | -160.68 (-11.88%) | 906.31(67.23%) | 181.49 (13.46%) | 1411.12 (104.68%) | -686.31 (-50.91%) | 14519.38(53.4%) | 1124.1 (4.13%) | 27405.69 (100.79%) | -14010.41 (-51.53%) |
| Latvia | 4666.58(20.4%) | 543.22 (2.37%) | 2897.67 (12.67%) | 1225.69 (5.36%) | -722.52(-12.75%) | 725.93 (12.81%) | 624.52 (11.02%) | -2072.97 (-36.58%) | -734.21(-14.81%) | 1133.55 (22.86%) | 544.53 (10.98%) | -2412.29 (-48.65%) | -17701.42(-22.39%) | 10949.74 (13.85%) | 8298.49 (10.5%) | -36949.65 (-46.73%) |
| Lebanon | 19521.54(218.51%) | 1195.15 (13.38%) | 16997.77 (190.26%) | 1328.62 (14.87%) | 2509.27(180.61%) | 599.08 (43.12%) | 2523.57 (181.63%) | -613.38 (-44.15%) | 709.14(62.95%) | 731.4 (64.93%) | 1685.54 (149.63%) | -1707.81 (-151.61%) | 8464.2(43.57%) | 7593.24 (39.08%) | 27183.63 (139.92%) | -26312.67 (-135.44%) |
| Lesotho | 2055.23(48.43%) | -363.76 (-8.57%) | 1273.09 (30%) | 1145.9 (27%) | 358.41(61.99%) | -78.06 (-13.5%) | 182.99 (31.65%) | 253.48 (43.84%) | 259.22(71.74%) | -88.04 (-24.37%) | 118.49 (32.79%) | 228.77 (63.32%) | 5511.2(84.57%) | -975.37 (-14.97%) | 2219.17 (34.05%) | 4267.4 (65.48%) |
| Liberia | 1981.42(28.38%) | -189.43 (-2.71%) | 3479.38 (49.83%) | -1308.53 (-18.74%) | 146.59(21.65%) | -9 (-1.33%) | 329.22 (48.62%) | -173.63 (-25.64%) | 303.26(48.46%) | 5.3 (0.85%) | 333.84 (53.35%) | -35.88 (-5.73%) | 5007.57(42.2%) | -374.93 (-3.16%) | 6199.75 (52.24%) | -817.25 (-6.89%) |
| Libya | 11437.69(182.76%) | 5.81 (0.09%) | 10211.78 (163.17%) | 1220.09 (19.5%) | 1466.1(178.03%) | -29.67 (-3.6%) | 1332.67 (161.83%) | 163.1 (19.81%) | 1234.48(167.82%) | -38.66 (-5.26%) | 1167.23 (158.68%) | 105.91 (14.4%) | 21839.77(173.85%) | -271.94 (-2.16%) | 20162.17 (160.5%) | 1949.54 (15.52%) |
| Lithuania | 7823.84(33.24%) | 81.87 (0.35%) | 7127.08 (30.28%) | 614.88 (2.61%) | 526.21(7.58%) | 677.83 (9.76%) | 1903.34 (27.41%) | -2054.96 (-29.59%) | 494.02(15.46%) | 715.49 (22.4%) | 906.94 (28.39%) | -1128.41 (-35.32%) | 378.22(0.69%) | 6759.61 (12.31%) | 14632.25 (26.65%) | -21013.64 (-38.27%) |
| Luxembourg | 216.91(7.97%) | 68.9 (2.53%) | 1860.82 (68.41%) | -1712.81 (-62.97%) | -74.05(-17.17%) | 51.4 (11.92%) | 274.09 (63.56%) | -399.54 (-92.66%) | -281.39(-58.32%) | 111.21 (23.05%) | 270.54 (56.07%) | -663.14 (-137.43%) | -4164.45(-60.2%) | 904.42 (13.07%) | 3750.36 (54.21%) | -8819.24 (-127.48%) |
| Madagascar | 33473.72(87.34%) | -2941.86 (-7.68%) | 37761.55 (98.53%) | -1345.98 (-3.51%) | 3339.82(87.88%) | -424.6 (-11.17%) | 3750.38 (98.68%) | 14.04 (0.37%) | 1448.79(58.12%) | -544.05 (-21.83%) | 2269.5 (91.05%) | -276.66 (-11.1%) | 32825.23(67.83%) | -6793.78 (-14.04%) | 45278.01 (93.56%) | -5658.99 (-11.69%) |
| Malawi | 17466.7(75.61%) | 737.93 (3.19%) | 19256.75 (83.36%) | -2527.98 (-10.94%) | 2000.33(80.31%) | 119.3 (4.79%) | 2102.93 (84.43%) | -221.9 (-8.91%) | 1621.95(121.29%) | 155.15 (11.6%) | 1253.83 (93.76%) | 212.97 (15.93%) | 29946.15(110.03%) | 1845.18 (6.78%) | 24828.32 (91.22%) | 3272.65 (12.02%) |
| Malaysia | 114603.19(217.03%) | -1250.44 (-2.37%) | 120976.13 (229.1%) | -5122.5 (-9.7%) | 10285.4(148.27%) | -499.36 (-7.2%) | 14180.18 (204.42%) | -3395.41 (-48.95%) | 5054.65(136.63%) | -658.04 (-17.79%) | 7464.08 (201.76%) | -1751.4 (-47.34%) | 100934.2(140.06%) | -5086.14 (-7.06%) | 146034.97 (202.65%) | -40014.63 (-55.53%) |
| Maldives | 710.36(142.88%) | 61.63 (12.4%) | 1097.22 (220.7%) | -448.49 (-90.21%) | 92.15(168.17%) | 19.65 (35.86%) | 125.99 (229.92%) | -53.48 (-97.61%) | 59.01(164.72%) | 28.42 (79.35%) | 83.89 (234.2%) | -53.31 (-148.82%) | 776.11(102.6%) | 278.95 (36.88%) | 1573.44 (208%) | -1076.28 (-142.28%) |
| Mali | 16414.27(90.82%) | 547.84 (3.03%) | 19843.36 (109.79%) | -3976.93 (-22%) | 1466.94(80.91%) | 89.44 (4.93%) | 1942.56 (107.15%) | -565.06 (-31.17%) | 1573.25(117.89%) | 131.02 (9.82%) | 1562.47 (117.08%) | -120.24 (-9.01%) | 29250.85(110.73%) | 1506.46 (5.7%) | 30414.81 (115.14%) | -2670.42 (-10.11%) |
| Malta | 734.13(38.97%) | 158.38 (8.41%) | 2074.83 (110.14%) | -1499.08 (-79.57%) | 12.19(3.86%) | 50.41 (15.94%) | 318.29 (100.62%) | -356.51 (-112.71%) | -51.63(-22.49%) | 68.8 (29.96%) | 221.37 (96.41%) | -341.8 (-148.86%) | -1110.01(-30.44%) | 702.54 (19.27%) | 3369.42 (92.41%) | -5181.96 (-142.12%) |
| Marshall Islands | 67.12(79.72%) | -11.17 (-13.27%) | 85.65 (101.73%) | -7.36 (-8.74%) | 6.79(69.13%) | -1.9 (-19.36%) | 9.72 (99.03%) | -1.03 (-10.54%) | 2.22(37.1%) | -1.81 (-30.25%) | 5.39 (90.22%) | -1.37 (-22.87%) | 60.71(50.1%) | -24.54 (-20.25%) | 113.71 (93.84%) | -28.46 (-23.49%) |
| Mauritania | 4892.82(65.44%) | -212.96 (-2.85%) | 7856.32 (105.08%) | -2750.55 (-36.79%) | 421.99(60.92%) | -10.83 (-1.56%) | 719.21 (103.83%) | -286.39 (-41.35%) | 482.41(76.72%) | 5.83 (0.93%) | 679.43 (108.06%) | -202.84 (-32.26%) | 7820.13(65.48%) | -388.96 (-3.26%) | 12539.44 (104.99%) | -4330.36 (-36.26%) |
| Mauritius | 3534.87(69.26%) | 176.03 (3.45%) | 7226.94 (141.6%) | -3868.1 (-75.79%) | 349.68(49%) | 70.31 (9.85%) | 970.48 (136%) | -691.12 (-96.85%) | 31.87(6.2%) | 81.73 (15.89%) | 636.26 (123.72%) | -686.12 (-133.42%) | -142.33(-1.39%) | 734.4 (7.15%) | 12327.88 (120.03%) | -13204.61 (-128.56%) |
| Mexico | 257472.68(149.92%) | 7353.42 (4.28%) | 330927.77 (192.69%) | -80808.51 (-47.05%) | 22150.19(107.24%) | 1787.62 (8.65%) | 36902.57 (178.66%) | -16539.99 (-80.08%) | 6361.17(53.17%) | 2107.41 (17.62%) | 19461.34 (162.67%) | -15207.58 (-127.12%) | 115358.86(59.76%) | 18337.22 (9.5%) | 315581.32 (163.47%) | -218559.67 (-113.21%) |
| Micronesia (Federated States of) | 70.53(24.59%) | -33.23 (-11.59%) | 128.85 (44.92%) | -25.08 (-8.74%) | 6.65(19.95%) | -5.21 (-15.64%) | 14.73 (44.19%) | -2.87 (-8.6%) | -0.03(-0.16%) | -4.28 (-20.03%) | 8.69 (40.64%) | -4.44 (-20.78%) | 25.1(5.96%) | -63.13 (-14.98%) | 175.86 (41.73%) | -87.62 (-20.79%) |
| Monaco | -45.18(-8.74%) | 2.01 (0.39%) | 151.71 (29.34%) | -198.9 (-38.46%) | -19.13(-26.08%) | 2.63 (3.58%) | 19.94 (27.18%) | -41.69 (-56.84%) | -25.82(-38.4%) | 6.14 (9.13%) | 17.36 (25.82%) | -49.31 (-73.35%) | -373.82(-40.43%) | 46.49 (5.03%) | 234.91 (25.41%) | -655.22 (-70.87%) |
| Mongolia | 4793.68(128.9%) | -336.55 (-9.05%) | 4161.42 (111.9%) | 968.81 (26.05%) | 420.69(123.33%) | -37.91 (-11.11%) | 377.03 (110.53%) | 81.57 (23.91%) | 141.35(80.16%) | -31.94 (-18.11%) | 174.91 (99.19%) | -1.62 (-0.92%) | 3329.49(89.12%) | -457.89 (-12.26%) | 3793.87 (101.54%) | -6.49 (-0.17%) |
| Montenegro | 1716.25(72.76%) | 42.86 (1.82%) | 1708.81 (72.44%) | -35.43 (-1.5%) | 252.17(74.33%) | -17.17 (-5.06%) | 247.35 (72.91%) | 21.98 (6.48%) | 305.74(147.81%) | -45.06 (-21.78%) | 185.17 (89.52%) | 165.64 (80.08%) | 4485.18(133.07%) | -321.69 (-9.54%) | 2892.3 (85.81%) | 1914.57 (56.8%) |
| Morocco | 108173.06(171.11%) | -1233.16 (-1.95%) | 98267.6 (155.44%) | 11138.62 (17.62%) | 16121.67(161.34%) | -421.8 (-4.22%) | 15238.41 (152.5%) | 1305.06 (13.06%) | 13136.75(128.66%) | -660.78 (-6.47%) | 14571.74 (142.71%) | -774.2 (-7.58%) | 226818.61(127.29%) | -8143.55 (-4.57%) | 253540.08 (142.29%) | -18577.92 (-10.43%) |
| Mozambique | 36403.67(101.85%) | -315.55 (-0.88%) | 30492.61 (85.31%) | 6226.62 (17.42%) | 4181.15(99.94%) | -49.11 (-1.17%) | 3551.75 (84.89%) | 678.52 (16.22%) | 3263.8(118.59%) | -70.66 (-2.57%) | 2452.08 (89.09%) | 882.38 (32.06%) | 67183.06(121.84%) | -926.88 (-1.68%) | 49512.56 (89.79%) | 18597.37 (33.73%) |
| Myanmar | 112006.35(98.87%) | 1900.49 (1.68%) | 126261.66 (111.45%) | -16155.79 (-14.26%) | 15127.89(101.1%) | 1179.63 (7.88%) | 16797.36 (112.25%) | -2849.09 (-19.04%) | 10724.71(73.65%) | 2428.81 (16.68%) | 15282.37 (104.95%) | -6986.47 (-47.98%) | 165719.25(57.6%) | 21265.72 (7.39%) | 289082.16 (100.48%) | -144628.64 (-50.27%) |
| Namibia | 3898.96(82.11%) | 374.51 (7.89%) | 4547.93 (95.77%) | -1023.47 (-21.55%) | 587.3(96.71%) | 59.83 (9.85%) | 603.36 (99.35%) | -75.88 (-12.5%) | 363.5(107.02%) | 76.42 (22.5%) | 346.67 (102.06%) | -59.59 (-17.54%) | 6196.56(90.25%) | 882.36 (12.85%) | 6715.95 (97.81%) | -1401.76 (-20.41%) |
| Nauru | 6.13(21.46%) | 1.01 (3.52%) | 7.66 (26.84%) | -2.54 (-8.91%) | 0.01(0.46%) | 0.12 (4.33%) | 0.66 (24.73%) | -0.76 (-28.6%) | 0.47(24.22%) | 0.23 (11.72%) | 0.53 (27.15%) | -0.29 (-14.66%) | 7.87(18.21%) | 2.71 (6.28%) | 11.44 (26.49%) | -6.29 (-14.55%) |
| Nepal | 36136.13(153.95%) | 1316 (5.61%) | 38927.55 (165.85%) | -4107.41 (-17.5%) | 5434.07(165.44%) | 411.48 (12.53%) | 5565.64 (169.45%) | -543.05 (-16.53%) | 3900.83(124.25%) | 606.6 (19.32%) | 4932.19 (157.1%) | -1637.96 (-52.17%) | 66206.4(109.03%) | 7662.24 (12.62%) | 92323.24 (152.05%) | -33779.08 (-55.63%) |
| Netherlands | 4653.56(3.07%) | 3995.08 (2.63%) | 93574.1 (61.67%) | -92915.62 (-61.24%) | -1999.53(-11.11%) | 897.33 (4.99%) | 10550.2 (58.63%) | -13447.07 (-74.73%) | -1049.1(-10.97%) | 1264.43 (13.22%) | 5654.18 (59.12%) | -7967.71 (-83.31%) | -26840.24(-18.05%) | 12089.95 (8.13%) | 85270.88 (57.33%) | -124201.07 (-83.51%) |
| New Zealand | 13260.98(65.25%) | 1086.61 (5.35%) | 20560.64 (101.16%) | -8386.27 (-41.26%) | 757.69(27.2%) | 265.7 (9.54%) | 2545.04 (91.38%) | -2053.05 (-73.71%) | 144.3(8.63%) | 399.57 (23.91%) | 1457.93 (87.23%) | -1713.21 (-102.5%) | -661.93(-2.52%) | 3645.58 (13.89%) | 21966.83 (83.7%) | -26274.33 (-100.12%) |
| Nicaragua | 9765.41(164.6%) | 160.13 (2.7%) | 12352.01 (208.2%) | -2746.74 (-46.3%) | 1069.18(135.19%) | 32.38 (4.09%) | 1567.75 (198.23%) | -530.95 (-67.14%) | 308.25(93.02%) | 17.1 (5.16%) | 613.06 (185%) | -321.91 (-97.14%) | 5497.95(98.29%) | 197.47 (3.53%) | 10405.09 (186.02%) | -5104.6 (-91.26%) |
| Niger | 22173.59(157.81%) | 411.14 (2.93%) | 26305.55 (187.21%) | -4543.1 (-32.33%) | 2111.26(156.15%) | 71.53 (5.29%) | 2523.64 (186.65%) | -483.9 (-35.79%) | 1881.12(211.77%) | 106.98 (12.04%) | 1821.67 (205.08%) | -47.53 (-5.35%) | 35044.08(194.63%) | 1378.24 (7.65%) | 35906.34 (199.42%) | -2240.5 (-12.44%) |
| Nigeria | 188453.67(76.29%) | -5931.39 (-2.4%) | 216934.71 (87.82%) | -22549.66 (-9.13%) | 16855.61(65.22%) | -612.09 (-2.37%) | 22011.96 (85.17%) | -4544.27 (-17.58%) | 10352.61(43.31%) | -370.88 (-1.55%) | 19101.78 (79.91%) | -8378.29 (-35.05%) | 183234.72(41.52%) | -12108.94 (-2.74%) | 350738.56 (79.47%) | -155394.9 (-35.21%) |
| Niue | -3.06(-18.13%) | -1.58 (-9.35%) | 0.16 (0.92%) | -1.64 (-9.7%) | -0.47(-24.98%) | -0.21 (-11.28%) | 0.02 (0.88%) | -0.27 (-14.58%) | -0.53(-36.98%) | -0.3 (-21.08%) | 0.01 (0.82%) | -0.24 (-16.72%) | -7.22(-31.06%) | -3.44 (-14.81%) | 0.2 (0.85%) | -3.98 (-17.1%) |
| North Macedonia | 9013.71(63.39%) | 617.29 (4.34%) | 12223.26 (85.96%) | -3826.84 (-26.91%) | 1861.73(62.84%) | -350.97 (-11.85%) | 2566.52 (86.63%) | -353.82 (-11.94%) | 1947.07(68.52%) | -473.37 (-16.66%) | 2546.66 (89.62%) | -126.22 (-4.44%) | 28796.2(60.97%) | -1855.32 (-3.93%) | 40963.69 (86.73%) | -10312.17 (-21.83%) |
| Northern Mariana Islands | 156.79(276.04%) | 0.69 (1.22%) | 172.08 (302.97%) | -15.99 (-28.15%) | 16.28(290.33%) | 0.02 (0.3%) | 17.3 (308.4%) | -1.03 (-18.38%) | 6.18(192.72%) | 0.37 (11.65%) | 8.74 (272.51%) | -2.93 (-91.43%) | 131.98(197.56%) | 3.53 (5.28%) | 182.82 (273.67%) | -54.37 (-81.39%) |
| Norway | 2342.76(3.93%) | 18.84 (0.03%) | 22007.91 (36.93%) | -19683.99 (-33.03%) | -1318.13(-16.7%) | 92.23 (1.17%) | 2675.24 (33.89%) | -4085.61 (-51.76%) | -2539.46(-58.05%) | 245.82 (5.62%) | 1219.14 (27.87%) | -4004.42 (-91.53%) | -36673.52(-55%) | 1533.12 (2.3%) | 18787.54 (28.18%) | -56994.18 (-85.48%) |
| Oman | 4556.9(167.06%) | -127.33 (-4.67%) | 4774.75 (175.04%) | -90.52 (-3.32%) | 594.04(150.22%) | -46.24 (-11.69%) | 671.59 (169.84%) | -31.31 (-7.92%) | 229.63(67.23%) | -49.95 (-14.62%) | 486.99 (142.57%) | -207.41 (-60.72%) | 4183.27(63.25%) | -631.05 (-9.54%) | 9349.99 (141.38%) | -4535.67 (-68.58%) |
| Pakistan | 212127.12(93.87%) | -8825.5 (-3.91%) | 229815.91 (101.7%) | -8863.29 (-3.92%) | 24602.91(82.34%) | -2256.4 (-7.55%) | 29494.49 (98.71%) | -2635.18 (-8.82%) | 17927.23(91.69%) | -2440.23 (-12.48%) | 19784.91 (101.19%) | 582.55 (2.98%) | 349795.63(95.47%) | -34129.85 (-9.32%) | 374268.77 (102.15%) | 9656.71 (2.64%) |
| Palau | 73(114.59%) | -6.66 (-10.46%) | 80.77 (126.79%) | -1.11 (-1.74%) | 6.6(99.06%) | -0.91 (-13.59%) | 8.15 (122.33%) | -0.64 (-9.68%) | 2.68(64.18%) | -0.79 (-18.91%) | 4.67 (111.99%) | -1.2 (-28.9%) | 61.05(71.81%) | -11.43 (-13.45%) | 97.22 (114.35%) | -24.74 (-29.1%) |
| Palestine | 4946.01(151.95%) | -65.19 (-2%) | 5359.91 (164.66%) | -348.71 (-10.71%) | 997.2(149.14%) | -88.39 (-13.22%) | 1096.4 (163.98%) | -10.82 (-1.62%) | 421.49(47.48%) | -134.9 (-15.2%) | 1159.66 (130.64%) | -603.28 (-67.96%) | 7547.19(52.38%) | -1458.25 (-10.12%) | 19094.32 (132.52%) | -10088.89 (-70.02%) |
| Panama | 9736.6(170.31%) | 437.01 (7.64%) | 11203.39 (195.96%) | -1903.8 (-33.3%) | 1109.54(135.82%) | 132.89 (16.27%) | 1511.02 (184.97%) | -534.38 (-65.42%) | 583(126.96%) | 122.81 (26.74%) | 839.52 (182.82%) | -379.33 (-82.61%) | 7850.79(108.1%) | 1105.3 (15.22%) | 12763.55 (175.75%) | -6018.06 (-82.87%) |
| Papua New Guinea | 8520.04(162.72%) | 433.42 (8.28%) | 8510.91 (162.55%) | -424.28 (-8.1%) | 972.47(167.63%) | 57.01 (9.83%) | 952.82 (164.25%) | -37.36 (-6.44%) | 653.12(165.74%) | 105.91 (26.88%) | 645.05 (163.69%) | -97.83 (-24.83%) | 12301.45(145.82%) | 1157.69 (13.72%) | 13276.42 (157.38%) | -2132.66 (-25.28%) |
| Paraguay | 12408.69(125.32%) | -183.58 (-1.85%) | 15420.7 (155.74%) | -2828.43 (-28.57%) | 1491.75(112.47%) | -6.85 (-0.52%) | 2013.51 (151.8%) | -514.92 (-38.82%) | 858.22(81.26%) | 5.76 (0.54%) | 1501.18 (142.14%) | -648.72 (-61.42%) | 13692.6(83.52%) | -194.61 (-1.19%) | 23392.13 (142.68%) | -9504.92 (-57.97%) |
| Peru | 58189(159.81%) | 1999.64 (5.49%) | 66381.78 (182.31%) | -10192.43 (-27.99%) | 4560.63(102.06%) | 474.46 (10.62%) | 7324.92 (163.92%) | -3238.76 (-72.48%) | 2102.73(84.23%) | 409.86 (16.42%) | 3969.54 (159%) | -2276.68 (-91.19%) | 33928.19(84.08%) | 4103.89 (10.17%) | 63871.21 (158.28%) | -34046.91 (-84.37%) |
| Philippines | 250458.44(230.86%) | -3840.53 (-3.54%) | 223742.65 (206.23%) | 30556.32 (28.16%) | 28699.46(254.84%) | -169.86 (-1.51%) | 24114.91 (214.13%) | 4754.41 (42.22%) | 16109.23(174.72%) | 715.95 (7.77%) | 17508.2 (189.89%) | -2114.92 (-22.94%) | 322403.24(191.11%) | 325.13 (0.19%) | 327578.5 (194.18%) | -5500.39 (-3.26%) |
| Poland | 138592.39(76.08%) | 9188.2 (5.04%) | 130986.85 (71.9%) | -1582.66 (-0.87%) | 15389.23(43.55%) | 4559.74 (12.9%) | 23232.63 (65.75%) | -12403.14 (-35.1%) | -13608.35(-28.7%) | 8662.63 (18.27%) | 24517.54 (51.71%) | -46788.52 (-98.68%) | -231120.42(-30.88%) | 76613.02 (10.24%) | 379186.96 (50.67%) | -686920.39 (-91.78%) |
| Portugal | -33407.03(-31.41%) | 8579.9 (8.07%) | 51213.61 (48.16%) | -93200.54 (-87.64%) | -6613.93(-36.27%) | 4878.21 (26.75%) | 8909.74 (48.85%) | -20401.89 (-111.87%) | -9914.62(-51.12%) | 9876.8 (50.92%) | 9620.78 (49.6%) | -29412.21 (-151.64%) | -168389.03(-58.7%) | 93806.73 (32.7%) | 131753.86 (45.93%) | -393949.63 (-137.33%) |
| Puerto Rico | 11718.33(99.28%) | 1545.63 (13.09%) | 12019.23 (101.83%) | -1846.53 (-15.64%) | 865.91(60.85%) | 516.35 (36.28%) | 1344.98 (94.51%) | -995.42 (-69.95%) | -72.1(-7.47%) | 532.51 (55.15%) | 803.95 (83.26%) | -1408.56 (-145.88%) | -927.07(-6.46%) | 4805.19 (33.47%) | 11496.29 (80.07%) | -17228.55 (-119.99%) |
| Qatar | 1708.35(371.28%) | -79 (-17.17%) | 2843.58 (618%) | -1056.22 (-229.55%) | 161.47(361.05%) | -13.92 (-31.11%) | 275.48 (615.95%) | -100.09 (-223.79%) | 58.35(181%) | -16.73 (-51.89%) | 168.53 (522.74%) | -93.44 (-289.85%) | 1177.03(192.1%) | -225.42 (-36.79%) | 3258.54 (531.81%) | -1856.09 (-302.92%) |
| Republic of Korea | 263132.05(86.85%) | 22805.73 (7.53%) | 627560.67 (207.12%) | -387234.35 (-127.81%) | 13620.06(39.04%) | 5493.33 (15.75%) | 66746.35 (191.31%) | -58619.62 (-168.02%) | 2701.34(13.45%) | 10674.67 (53.16%) | 38652.45 (192.49%) | -46625.78 (-232.19%) | 6005.53(1.58%) | 104797.96 (27.64%) | 688504.73 (181.61%) | -787297.16 (-207.67%) |
| Republic of Moldova | 9212.08(46.17%) | -388.83 (-1.95%) | 8860.78 (44.41%) | 740.13 (3.71%) | 1259.32(33.48%) | 497.55 (13.23%) | 1614.89 (42.94%) | -853.13 (-22.68%) | 367.34(13.96%) | 755.72 (28.72%) | 1094.19 (41.59%) | -1482.57 (-56.35%) | 10108.6(22.16%) | 6488 (14.22%) | 19103.84 (41.88%) | -15483.24 (-33.94%) |
| Romania | 57520.88(28.7%) | 13648.07 (6.81%) | 73295.22 (36.57%) | -29422.41 (-14.68%) | 5880.44(15.76%) | 7638.97 (20.47%) | 13162.58 (35.27%) | -14921.12 (-39.98%) | 4664.17(14.14%) | 14079.51 (42.69%) | 11880.25 (36.02%) | -21295.6 (-64.57%) | 15593.01(2.86%) | 138539.62 (25.39%) | 184858.15 (33.88%) | -307804.76 (-56.41%) |
| Russian Federation | 360918.41(35.28%) | -2104.08 (-0.21%) | 403113.77 (39.4%) | -40091.28 (-3.92%) | 30517.82(12.58%) | 26279.43 (10.84%) | 88283.97 (36.41%) | -84045.57 (-34.66%) | -33895.65(-12.8%) | 40122.15 (15.15%) | 87865.4 (33.18%) | -161883.2 (-61.14%) | -780178.6(-17.6%) | 316742.42 (7.15%) | 1426982.71 (32.19%) | -2523903.73 (-56.94%) |
| Rwanda | 12199.07(63.53%) | 186.08 (0.97%) | 20673.38 (107.67%) | -8660.39 (-45.1%) | 1360.91(62.73%) | 47.9 (2.21%) | 2330.84 (107.44%) | -1017.83 (-46.92%) | 354.8(25.1%) | 99.42 (7.03%) | 1375.62 (97.3%) | -1120.24 (-79.24%) | 5378.56(18.67%) | 719.19 (2.5%) | 27455.17 (95.29%) | -22795.79 (-79.12%) |
| Saint Kitts and Nevis | 4.64(1.6%) | -36.84 (-12.72%) | 160.2 (55.32%) | -118.72 (-40.99%) | -2.03(-4.9%) | -7.39 (-17.84%) | 22.22 (53.61%) | -16.86 (-40.67%) | -14.15(-30.53%) | -11.63 (-25.09%) | 22.15 (47.77%) | -24.67 (-53.21%) | -209.38(-28.14%) | -171.79 (-23.08%) | 360.08 (48.39%) | -397.68 (-53.44%) |
| Saint Lucia | 448.77(96.76%) | -8.93 (-1.92%) | 672.9 (145.08%) | -215.2 (-46.4%) | 53.12(76.47%) | 9.16 (13.18%) | 97.54 (140.41%) | -53.58 (-77.12%) | 36.76(47.24%) | 25.75 (33.08%) | 104.19 (133.89%) | -93.18 (-119.73%) | 408.17(34.38%) | 203.59 (17.15%) | 1518.55 (127.91%) | -1313.98 (-110.68%) |
| Saint Vincent and the Grenadines | 193.25(58.28%) | -12.63 (-3.81%) | 298.51 (90.02%) | -92.63 (-27.93%) | 22.03(45.79%) | -0.72 (-1.5%) | 41.83 (86.92%) | -19.07 (-39.63%) | 10.5(22.28%) | 0.16 (0.35%) | 38.08 (80.85%) | -27.75 (-58.91%) | 124.52(16.83%) | -18.67 (-2.52%) | 587.21 (79.38%) | -444.02 (-60.02%) |
| Samoa | 312.62(65.15%) | 18.15 (3.78%) | 321.55 (67.01%) | -27.08 (-5.64%) | 27.44(52.6%) | 2.39 (4.57%) | 33.67 (64.54%) | -8.61 (-16.51%) | 14.65(49.56%) | 3.42 (11.56%) | 18.92 (64.04%) | -7.69 (-26.04%) | 270.81(47.47%) | 40.1 (7.03%) | 362.53 (63.54%) | -131.81 (-23.1%) |
| San Marino | 106.43(51.19%) | 21.17 (10.18%) | 183.97 (88.49%) | -98.71 (-47.47%) | 8.83(33.36%) | 4.91 (18.55%) | 22.41 (84.7%) | -18.5 (-69.89%) | -1.67(-9%) | 8.57 (46.26%) | 14.61 (78.9%) | -24.84 (-134.16%) | -27.86(-10.54%) | 77.93 (29.49%) | 201.58 (76.28%) | -307.37 (-116.32%) |
| Sao Tome and Principe | 249.89(48.38%) | -14.25 (-2.76%) | 265.09 (51.32%) | -0.95 (-0.18%) | 23.1(50.49%) | -2 (-4.36%) | 23.65 (51.7%) | 1.44 (3.15%) | 20.72(61.14%) | -2.73 (-8.05%) | 18.17 (53.61%) | 5.28 (15.58%) | 393.78(63.68%) | -34.22 (-5.53%) | 333.94 (54%) | 94.06 (15.21%) |
| Saudi Arabia | 34956.04(175.23%) | -2280.43 (-11.43%) | 37001.03 (185.48%) | 235.44 (1.18%) | 3972.5(115.19%) | -856.38 (-24.83%) | 5650.36 (163.85%) | -821.49 (-23.82%) | 1558.55(43.36%) | -1322.15 (-36.78%) | 4999.12 (139.07%) | -2118.42 (-58.93%) | 37796.45(60.19%) | -14668.83 (-23.36%) | 91321.17 (145.43%) | -38855.89 (-61.88%) |
| Senegal | 22622.38(105.3%) | 12.53 (0.06%) | 27624.3 (128.58%) | -5014.45 (-23.34%) | 1987.74(103.15%) | 24.35 (1.26%) | 2465.43 (127.94%) | -502.03 (-26.05%) | 2341.86(126.45%) | 75.97 (4.1%) | 2493.09 (134.62%) | -227.21 (-12.27%) | 40618.77(114.79%) | 335.36 (0.95%) | 46449.67 (131.27%) | -6166.26 (-17.43%) |
| Serbia | 24107.47(30.77%) | 10711.74 (13.67%) | 37096.42 (47.34%) | -23700.69 (-30.25%) | 8038.98(54.15%) | 4827.06 (32.51%) | 7612.23 (51.27%) | -4400.3 (-29.64%) | 4651.17(26.73%) | 9234.47 (53.07%) | 8622.25 (49.55%) | -13205.55 (-75.88%) | 49191.92(17.75%) | 109974.46 (39.68%) | 131404.72 (47.41%) | -192187.26 (-69.34%) |
| Seychelles | 210.51(60.05%) | -29.16 (-8.32%) | 308.27 (87.93%) | -68.6 (-19.57%) | 18.24(43.31%) | -5.17 (-12.27%) | 35.25 (83.71%) | -11.85 (-28.14%) | 4.77(14.59%) | -6.31 (-19.31%) | 24.94 (76.25%) | -13.85 (-42.36%) | 100.65(17.61%) | -77.46 (-13.56%) | 441.25 (77.22%) | -263.14 (-46.05%) |
| Sierra Leone | 7230.08(51.8%) | -390.39 (-2.8%) | 9432.23 (67.58%) | -1811.76 (-12.98%) | 600.5(45.48%) | -46.64 (-3.53%) | 874.71 (66.25%) | -227.57 (-17.24%) | 697.52(52.29%) | -73.86 (-5.54%) | 902.78 (67.68%) | -131.4 (-9.85%) | 12961.36(52.25%) | -1083.06 (-4.37%) | 16785.86 (67.67%) | -2741.45 (-11.05%) |
| Singapore | 18784.47(97.46%) | 825.97 (4.29%) | 47365.62 (245.74%) | -29407.12 (-152.57%) | 857.7(41.91%) | 161.67 (7.9%) | 4599.87 (224.75%) | -3903.84 (-190.74%) | -346.43(-38.48%) | 161.75 (17.96%) | 1784.16 (198.16%) | -2292.33 (-254.61%) | -4561.01(-24.82%) | 1463.41 (7.96%) | 36778.77 (200.16%) | -42803.18 (-232.95%) |
| Slovakia | 11266.03(17.52%) | 1243.34 (1.93%) | 35103.93 (54.58%) | -25081.24 (-38.99%) | 852.92(10.55%) | 203.17 (2.51%) | 4302.06 (53.21%) | -3652.31 (-45.17%) | -783.04(-14.65%) | 282.49 (5.29%) | 2590.24 (48.48%) | -3655.77 (-68.42%) | -13798.41(-14.49%) | 2982.09 (3.13%) | 46128.77 (48.43%) | -62909.27 (-66.04%) |
| Slovenia | 4839.98(41.8%) | 839.62 (7.25%) | 8630.12 (74.53%) | -4629.76 (-39.98%) | -285.29(-10.59%) | 409.41 (15.2%) | 1715.46 (63.68%) | -2410.16 (-89.47%) | -543.8(-27.54%) | 412.11 (20.87%) | 1183.56 (59.94%) | -2139.46 (-108.35%) | -11404.56(-35.99%) | 4033.01 (12.73%) | 18218.7 (57.5%) | -33656.26 (-106.22%) |
| Solomon Islands | 1097.51(151.1%) | 68.89 (9.48%) | 1044.73 (143.84%) | -16.11 (-2.22%) | 144.04(165.66%) | 13.58 (15.62%) | 128.63 (147.94%) | 1.83 (2.1%) | 63.56(170.55%) | 13.45 (36.08%) | 55.8 (149.72%) | -5.68 (-15.25%) | 1220.17(148.42%) | 160.26 (19.49%) | 1176.81 (143.15%) | -116.91 (-14.22%) |
| Somalia | 15783.79(153.55%) | -291.93 (-2.84%) | 18203.29 (177.08%) | -2127.57 (-20.7%) | 1627.12(145.85%) | -77.69 (-6.96%) | 1949.3 (174.73%) | -244.49 (-21.92%) | 622.19(104.59%) | -125.91 (-21.17%) | 955.38 (160.6%) | -207.28 (-34.84%) | 14838.1(121.42%) | -1172.84 (-9.6%) | 20342.19 (166.46%) | -4331.25 (-35.44%) |
| South Africa | 153669.06(94.22%) | -6275.94 (-3.85%) | 195479.87 (119.86%) | -35534.87 (-21.79%) | 20843.55(129.91%) | -949.36 (-5.92%) | 20867.89 (130.06%) | 925.02 (5.77%) | 12125.75(180.7%) | -1106.79 (-16.49%) | 9722.41 (144.89%) | 3510.13 (52.31%) | 215974.69(171.23%) | -11262.66 (-8.93%) | 178984.95 (141.91%) | 48252.4 (38.26%) |
| South Sudan | 1437.79(9.15%) | -785.4 (-5%) | 4426.57 (28.18%) | -2203.39 (-14.03%) | 220.39(13.49%) | -91.06 (-5.57%) | 469.03 (28.71%) | -157.57 (-9.65%) | 26.1(2.78%) | -58.82 (-6.28%) | 256.77 (27.4%) | -171.86 (-18.34%) | 466.34(2.52%) | -1258.35 (-6.8%) | 5065.39 (27.37%) | -3340.71 (-18.05%) |
| Spain | 164648.63(55.34%) | 35211.02 (11.83%) | 191176.97 (64.26%) | -61739.37 (-20.75%) | -7868.53(-15.34%) | 9807.92 (19.13%) | 26510.24 (51.7%) | -44186.69 (-86.17%) | -16657.12(-45.88%) | 16983.69 (46.78%) | 17774.26 (48.96%) | -51415.06 (-141.62%) | -231566.46(-44.73%) | 160316.28 (30.97%) | 245261.06 (47.38%) | -637143.8 (-123.08%) |
| Sri Lanka | 82842.47(157.45%) | 2198.56 (4.18%) | 93362.79 (177.44%) | -12718.88 (-24.17%) | 9660.13(143%) | 694.87 (10.29%) | 11686.17 (172.99%) | -2720.91 (-40.28%) | 7895.45(115.51%) | 1334.88 (19.53%) | 11264.09 (164.79%) | -4703.53 (-68.81%) | 123514.15(108.4%) | 13713.88 (12.04%) | 184698.17 (162.1%) | -74897.9 (-65.74%) |
| Sudan | 42950.99(112.83%) | -1538.62 (-4.04%) | 38280.29 (100.56%) | 6209.32 (16.31%) | 6196.7(102%) | 151.59 (2.5%) | 5949.03 (97.92%) | 96.08 (1.58%) | 3751.53(57.62%) | 361.46 (5.55%) | 5662.81 (86.97%) | -2272.74 (-34.91%) | 61810.45(51.2%) | -205.76 (-0.17%) | 102993.3 (85.31%) | -40977.09 (-33.94%) |
| Suriname | 1399.8(141.28%) | 26.88 (2.71%) | 1523.94 (153.8%) | -151.01 (-15.24%) | 189.61(138.41%) | 2.44 (1.78%) | 209.44 (152.88%) | -22.27 (-16.25%) | 107.46(92.75%) | 3.49 (3.01%) | 161.43 (139.34%) | -57.46 (-49.6%) | 1745.49(92.42%) | 71.19 (3.77%) | 2629.03 (139.2%) | -954.73 (-50.55%) |
| Sweden | 7619.72(7.23%) | 5127.87 (4.87%) | 35659.05 (33.84%) | -33167.2 (-31.47%) | -1427.89(-10.23%) | 963.54 (6.9%) | 4395.96 (31.5%) | -6787.39 (-48.63%) | -3549.3(-43.64%) | 1207.72 (14.85%) | 2233.91 (27.47%) | -6990.94 (-85.96%) | -52684.63(-43.35%) | 11384.06 (9.37%) | 33104.2 (27.24%) | -97172.9 (-79.96%) |
| Switzerland | 18354.24(40.78%) | 1059.05 (2.35%) | 29099.45 (64.65%) | -11804.27 (-26.22%) | -181.4(-2.72%) | 487.66 (7.3%) | 3741.8 (56.01%) | -4410.86 (-66.03%) | -2106.41(-41.91%) | 902.2 (17.95%) | 2464.57 (49.03%) | -5473.18 (-108.89%) | -28090.46(-40.32%) | 7591.71 (10.9%) | 33955.74 (48.74%) | -69637.92 (-99.95%) |
| Syrian Arab Republic | 26150.51(130.41%) | 379.47 (1.89%) | 31177.87 (155.48%) | -5406.83 (-26.96%) | 3226.62(109.62%) | -369.12 (-12.54%) | 4375.11 (148.64%) | -779.37 (-26.48%) | 2571.56(89.09%) | -579.42 (-20.07%) | 4114.42 (142.55%) | -963.44 (-33.38%) | 48419.3(97.49%) | -4122.61 (-8.3%) | 72123.37 (145.22%) | -19581.47 (-39.43%) |
| Taiwan (Province of China) | 160027.84(139.81%) | 35992.4 (31.44%) | 196015.89 (171.25%) | -71980.45 (-62.88%) | 13370.77(91.77%) | 3755.13 (25.77%) | 22669.53 (155.59%) | -13053.9 (-89.6%) | 39.54(0.65%) | 3025.51 (49.51%) | 8155.97 (133.47%) | -11141.95 (-182.34%) | 8914.92(6.96%) | 35525.57 (27.72%) | 167719 (130.85%) | -194329.65 (-151.61%) |
| Tajikistan | 11929.02(98.77%) | -730.18 (-6.05%) | 12373.6 (102.45%) | 285.6 (2.36%) | 2425.01(131.66%) | -418.81 (-22.74%) | 2066.94 (112.22%) | 776.88 (42.18%) | 708.28(39.99%) | -519.4 (-29.33%) | 1543.58 (87.16%) | -315.9 (-17.84%) | 15551.62(49.07%) | -5658.05 (-17.85%) | 28403.4 (89.63%) | -7193.74 (-22.7%) |
| Thailand | 346729.44(211.68%) | 19882.89 (12.14%) | 392764.95 (239.79%) | -65918.4 (-40.24%) | 30189.77(167.4%) | 3442.87 (19.09%) | 40540.3 (224.79%) | -13793.41 (-76.48%) | 16234.1(155.47%) | 4137.32 (39.62%) | 23283.78 (222.98%) | -11187 (-107.13%) | 288201.98(139.74%) | 41823.49 (20.28%) | 444481.39 (215.51%) | -198102.9 (-96.05%) |
| Timor-Leste | 3134.88(359.96%) | 209.89 (24.1%) | 2775.72 (318.72%) | 149.28 (17.14%) | 441.23(390.68%) | 28.31 (25.07%) | 371.86 (329.26%) | 41.06 (36.36%) | 335.27(375.5%) | 25.59 (28.66%) | 290.15 (324.97%) | 19.53 (21.87%) | 6172.31(373.26%) | 519 (31.39%) | 5354.58 (323.81%) | 298.74 (18.07%) |
| Togo | 12019.89(163.15%) | -296.09 (-4.02%) | 14105.45 (191.46%) | -1789.47 (-24.29%) | 1080.2(157.95%) | -46.01 (-6.73%) | 1297.47 (189.72%) | -171.26 (-25.04%) | 1112.05(180.11%) | -87.86 (-14.23%) | 1218.04 (197.28%) | -18.13 (-2.94%) | 22245.83(185.82%) | -976.65 (-8.16%) | 23843.33 (199.17%) | -620.84 (-5.19%) |
| Tokelau | 0.51(6.64%) | 0.7 (9.25%) | 0.09 (1.13%) | -0.28 (-3.74%) | -0.08(-9.08%) | 0.11 (12.32%) | 0.01 (1.05%) | -0.19 (-22.46%) | -0.09(-12.84%) | 0.16 (22.95%) | 0.01 (1.04%) | -0.26 (-36.82%) | -2.34(-18.36%) | 1.76 (13.77%) | 0.13 (1%) | -4.23 (-33.14%) |
| Tonga | 125.4(52.09%) | 27.43 (11.39%) | 112.09 (46.56%) | -14.12 (-5.86%) | 14.81(59.47%) | 3.54 (14.23%) | 11.88 (47.68%) | -0.61 (-2.44%) | 9.17(76.2%) | 4.12 (34.19%) | 6.05 (50.28%) | -0.99 (-8.27%) | 133.62(55.25%) | 46.08 (19.05%) | 113.7 (47.02%) | -26.17 (-10.82%) |
| Trinidad and Tobago | 3576.88(79.5%) | -142.01 (-3.16%) | 5767.68 (128.19%) | -2048.79 (-45.53%) | 380.94(62.14%) | 18.61 (3.04%) | 756.22 (123.37%) | -393.89 (-64.26%) | 210.02(39.67%) | 70.14 (13.25%) | 629.29 (118.86%) | -489.42 (-92.44%) | 2803.73(31.66%) | 333.78 (3.77%) | 10194.77 (115.1%) | -7724.82 (-87.22%) |
| Tunisia | 36416.1(200.14%) | -316.49 (-1.74%) | 33709.22 (185.26%) | 3023.37 (16.62%) | 5305.65(196.75%) | 425.05 (15.76%) | 4973.25 (184.42%) | -92.65 (-3.44%) | 3779.09(145.29%) | 860.63 (33.09%) | 4389.62 (168.76%) | -1471.16 (-56.56%) | 58013.16(126.47%) | 7056.96 (15.38%) | 74388.18 (162.17%) | -23431.98 (-51.08%) |
| Turkey | 226291.43(124.48%) | 12741.04 (7.01%) | 307158.63 (168.97%) | -93608.24 (-51.49%) | 26657.63(114.84%) | 2503.52 (10.78%) | 38544.97 (166.04%) | -14390.86 (-61.99%) | 16194.61(81.03%) | 3175.51 (15.89%) | 31166.32 (155.94%) | -18147.21 (-90.8%) | 230983.03(67.53%) | 39897.56 (11.66%) | 517204.11 (151.21%) | -326118.64 (-95.34%) |
| Turkmenistan | 15182.3(151.57%) | -312.74 (-3.12%) | 12107.42 (120.87%) | 3387.62 (33.82%) | 1866.47(130.83%) | -26.35 (-1.85%) | 1647.66 (115.49%) | 245.15 (17.18%) | 1506.06(148.05%) | -36.65 (-3.6%) | 1226.38 (120.55%) | 316.33 (31.1%) | 31023.89(150.49%) | -856.79 (-4.16%) | 24938.42 (120.97%) | 6942.26 (33.67%) |
| Tuvalu | 21.17(62.87%) | 2.58 (7.67%) | 19.7 (58.5%) | -1.11 (-3.3%) | 2.34(62.63%) | 0.45 (11.94%) | 2.18 (58.47%) | -0.29 (-7.78%) | 1.29(48.61%) | 0.72 (27.21%) | 1.49 (56.31%) | -0.92 (-34.91%) | 20.25(36.23%) | 8.06 (14.42%) | 30.1 (53.85%) | -17.91 (-32.05%) |
| Uganda | 41207.3(94.99%) | -340.91 (-0.79%) | 48036.06 (110.73%) | -6487.85 (-14.96%) | 4612.17(103.14%) | -32.5 (-0.73%) | 5050.84 (112.95%) | -406.17 (-9.08%) | 1400.65(65.66%) | 57.61 (2.7%) | 2195.57 (102.93%) | -852.54 (-39.97%) | 27681.88(65.51%) | -221.72 (-0.52%) | 43439.24 (102.8%) | -15535.64 (-36.76%) |
| Ukraine | -53431.4(-10.17%) | 1425.33 (0.27%) | 50406.85 (9.6%) | -105263.58 (-20.04%) | -24591.04(-22.86%) | 7608.63 (7.07%) | 9742.39 (9.06%) | -41942.06 (-38.98%) | -32496.86(-34.52%) | 11836.15 (12.57%) | 8067.71 (8.57%) | -52400.72 (-55.66%) | -560684.19(-35.7%) | 105591.69 (6.72%) | 132678.49 (8.45%) | -798954.37 (-50.88%) |
| United Arab Emirates | 13814.69(656.5%) | -1145.44 (-54.43%) | 16546.02 (786.3%) | -1585.89 (-75.36%) | 1189.32(423.96%) | -328.88 (-117.24%) | 1880.67 (670.4%) | -362.46 (-129.21%) | 304.38(172.06%) | -372.43 (-210.53%) | 1002.99 (566.98%) | -326.18 (-184.39%) | 8153.27(229.92%) | -4903.49 (-138.28%) | 21322.05 (601.28%) | -8265.29 (-233.08%) |
| United Kingdom | 22836.34(4.19%) | 20710.25 (3.8%) | 181411.56 (33.3%) | -179285.48 (-32.91%) | -20777(-25.54%) | 6032.63 (7.42%) | 23929.46 (29.42%) | -50739.09 (-62.37%) | -34004.49(-57.72%) | 8752.1 (14.85%) | 15052.29 (25.55%) | -57808.88 (-98.12%) | -515418.47(-58.19%) | 78416.09 (8.85%) | 222765.26 (25.15%) | -816599.82 (-92.19%) |
| United Republic of Tanzania | 100448.42(166.27%) | 2455.56 (4.06%) | 81386.53 (134.71%) | 16606.33 (27.49%) | 11284.96(175.88%) | 404.04 (6.3%) | 8812.07 (137.34%) | 2068.86 (32.24%) | 6151.26(218.53%) | 710.72 (25.25%) | 4178.01 (148.43%) | 1262.52 (44.85%) | 109898.35(190.77%) | 6291.26 (10.92%) | 81373.56 (141.26%) | 22233.53 (38.6%) |
| United States of America | 1577526.74(72.9%) | -34643.3 (-1.6%) | 1814516.93 (83.85%) | -202346.88 (-9.35%) | 20577.96(9.18%) | -1625.75 (-0.72%) | 154622.21 (68.95%) | -132418.51 (-59.05%) | 15869(16.28%) | 4532.21 (4.65%) | 68848.7 (70.62%) | -57511.91 (-58.99%) | 293043.87(18.09%) | 14108.79 (0.87%) | 1149844.5 (70.99%) | -870909.42 (-53.77%) |
| United States Virgin Islands | 416.11(173.48%) | 30.16 (12.57%) | 389.12 (162.23%) | -3.16 (-1.32%) | 53.95(189.61%) | 7.6 (26.7%) | 47.57 (167.2%) | -1.22 (-4.29%) | 9.84(42.61%) | 9.05 (39.21%) | 29.26 (126.7%) | -28.47 (-123.3%) | 140.97(36.4%) | 99.63 (25.73%) | 477.57 (123.33%) | -436.23 (-112.65%) |
| Uruguay | -3869.43(-12.4%) | 1002.28 (3.21%) | 9560.85 (30.64%) | -14432.56 (-46.25%) | -374.97(-10.71%) | 438.35 (12.52%) | 1088.73 (31.09%) | -1902.05 (-54.31%) | -502.83(-18.07%) | 782.75 (28.12%) | 859.32 (30.87%) | -2144.9 (-77.06%) | -11666.42(-26.24%) | 7133.88 (16.05%) | 13028.79 (29.31%) | -31829.09 (-71.6%) |
| Uzbekistan | 85100.9(128.42%) | -1232 (-1.86%) | 82396.12 (124.34%) | 3936.77 (5.94%) | 12774.98(143.01%) | -1666.68 (-18.66%) | 11570.03 (129.52%) | 2871.63 (32.15%) | 4980.87(91.68%) | -1635.81 (-30.11%) | 6239.6 (114.84%) | 377.08 (6.94%) | 105925.91(98.98%) | -15993.59 (-14.94%) | 124750.42 (116.57%) | -2830.92 (-2.65%) |
| Vanuatu | 633.47(179.24%) | 13.06 (3.69%) | 631.53 (178.69%) | -11.13 (-3.15%) | 65.81(179.77%) | 2.08 (5.68%) | 65.47 (178.85%) | -1.74 (-4.76%) | 29.35(138.84%) | 2.97 (14.06%) | 35.11 (166.08%) | -8.73 (-41.3%) | 613.47(134.23%) | 32.02 (7.01%) | 751.33 (164.39%) | -169.89 (-37.17%) |
| Venezuela (Bolivarian Republic of) | 62535.5(172.08%) | 333.56 (0.92%) | 78517.18 (216.06%) | -16315.24 (-44.9%) | 7392.27(162.39%) | 220.65 (4.85%) | 9692.49 (212.92%) | -2520.87 (-55.38%) | 4629.27(192.62%) | 186.29 (7.75%) | 5410.04 (225.1%) | -967.06 (-40.24%) | 69744.53(174.93%) | 781.11 (1.96%) | 86895.77 (217.95%) | -17932.36 (-44.98%) |
| Viet Nam | 331240.41(169.05%) | -9828.63 (-5.02%) | 295648.66 (150.89%) | 45420.37 (23.18%) | 42425.29(144.2%) | -1579.44 (-5.37%) | 42192.94 (143.41%) | 1811.79 (6.16%) | 41403.61(145.55%) | -2332.22 (-8.2%) | 40941.34 (143.93%) | 2794.49 (9.82%) | 698063.96(144.55%) | -37992.1 (-7.87%) | 693700.45 (143.65%) | 42355.61 (8.77%) |
| Yemen | 32512.4(186.56%) | 16.61 (0.1%) | 32034.47 (183.82%) | 461.32 (2.65%) | 5452.26(191.17%) | 276.2 (9.68%) | 5285.4 (185.32%) | -109.35 (-3.83%) | 6036.66(181.98%) | 567.95 (17.12%) | 6048.63 (182.34%) | -579.93 (-17.48%) | 105975.07(165.03%) | 5536.23 (8.62%) | 113576.47 (176.87%) | -13137.62 (-20.46%) |
| Zambia | 24571.96(148.46%) | -97.56 (-0.59%) | 22740.51 (137.39%) | 1929.01 (11.65%) | 3095.09(175.45%) | -12.89 (-0.73%) | 2559.88 (145.11%) | 548.09 (31.07%) | 1608.71(174.21%) | -14.14 (-1.53%) | 1337.25 (144.81%) | 285.6 (30.93%) | 30568.85(169.64%) | -176.91 (-0.98%) | 25853.5 (143.47%) | 4892.26 (27.15%) |
| Zimbabwe | 17106.76(79.5%) | -982.39 (-4.57%) | 14433.8 (67.08%) | 3655.36 (16.99%) | 1980.19(81.1%) | -177.1 (-7.25%) | 1647.76 (67.49%) | 509.52 (20.87%) | 1452.18(111.91%) | -201.4 (-15.52%) | 955.98 (73.67%) | 697.6 (53.76%) | 30650.34(121.87%) | -2377.12 (-9.45%) | 18993.69 (75.52%) | 14033.78 (55.8%) |
